# Novel drug targets for personalized precision medicine in relapsed/refractory diffuse large B-cell lymphoma: a comprehensive review

**DOI:** 10.1186/s12943-015-0474-2

**Published:** 2015-12-11

**Authors:** Rosalba Camicia, Hans C. Winkler, Paul O. Hassa

**Affiliations:** Institute of Veterinary Biochemistry and Molecular Biology, University of Zurich, Winterthurerstrasse 190, 8057 Zurich, Switzerland; Stem Cell Research Laboratory, NHS Blood and Transplant, Nuffield Division of Clinical, Laboratory Sciences, Radcliffe Department of Medicine, University of Oxford, Oxford, OX3 9DU UK; MRC-UCL Laboratory for Molecular Cell Biology Unit, University College London, Gower Street, London, WC1E6BT UK; Institute of Pharmacology and Toxicology, Vetsuisse Faculty, University of Zurich, Winterthurerstrasse 260, 8057 Zurich, Switzerland

**Keywords:** Macrodomain, DLBCL, ARTD, PARP, DTX3L, NF-κB, STAT1, STAT3, Chemoresistance, ADP-ribosylation, Antibody drug conjugate, ABC-DLBCL, GCB-DLBCL, BCR, CAR-T cells

## Abstract

Diffuse large B-cell lymphoma (DLBCL) is a clinically heterogeneous lymphoid malignancy and the most common subtype of non-Hodgkin’s lymphoma in adults, with one of the highest mortality rates in most developed areas of the world. More than half of DLBLC patients can be cured with standard R-CHOP regimens, however approximately 30 to 40 % of patients will develop relapsed/refractory disease that remains a major cause of morbidity and mortality due to the limited therapeutic options.

Recent advances in gene expression profiling have led to the identification of at least three distinct molecular subtypes of DLBCL: a germinal center B cell-like subtype, an activated B cell-like subtype, and a primary mediastinal B-cell lymphoma subtype. Moreover, recent findings have not only increased our understanding of the molecular basis of chemotherapy resistance but have also helped identify molecular subsets of DLBCL and rational targets for drug interventions that may allow for subtype/subset-specific molecularly targeted precision medicine and personalized combinations to both prevent and treat relapsed/refractory DLBCL. Novel agents such as lenalidomide, ibrutinib, bortezomib, CC-122, epratuzumab or pidilizumab used as single-agent or in combination with (rituximab-based) chemotherapy have already demonstrated promising activity in patients with relapsed/refractory DLBCL. Several novel potential drug targets have been recently identified such as the BET bromodomain protein (BRD)-4, phosphoribosyl-pyrophosphate synthetase (PRPS)-2, macrodomain-containing mono-ADP-ribosyltransferase (ARTD)-9 (also known as PARP9), deltex-3-like E3 ubiquitin ligase (DTX3L) (also known as BBAP), NF-kappaB inducing kinase (NIK) and transforming growth factor beta receptor (TGFβR).

This review highlights the new insights into the molecular basis of relapsed/refractory DLBCL and summarizes the most promising drug targets and experimental treatments for relapsed/refractory DLBCL, including the use of novel agents such as lenalidomide, ibrutinib, bortezomib, pidilizumab, epratuzumab, brentuximab-vedotin or CAR T cells, dual inhibitors, as well as mechanism-based combinatorial experimental therapies. We also provide a comprehensive and updated list of current drugs, drug targets and preclinical and clinical experimental studies in DLBCL. A special focus is given on STAT1, ARTD9, DTX3L and ARTD8 (also known as PARP14) as novel potential drug targets in distinct molecular subsets of DLBCL.

**Electronic supplementary material:**

The online version of this article (doi:10.1186/s12943-015-0474-2) contains supplementary material, which is available to authorized users.

## Introduction

Diffuse large B-cell lymphoma (DLBCL) is a clinically and genetically heterogeneous lymphoid malignancy with molecular subtypes and subsets defined by distinct molecular signatures and clinical outcomes. Many subtypes and subsets are at high-risk for treatment failure with standard immuno-chemotherapy [1–4]. DLBCL is by far the most common category and disease entity of B-cell non-Hodgkin lymphoma (NHL) in adults, with one of the highest mortality rates of B-cell NHL in most developed areas of the world [1–3]. In Europe and USA, the current annual incidence of NHL is estimated to be 15–20 cases/100,000 [5]. DLBCL accounts for approximately 30–40 % of all newly diagnosed B-cell NHL cases in Western countries, and for an even higher percentage in developing countries [6–9]. The median age of DLBCL at diagnosis falls between the sixth and seventh decade [1, 9]. DLBCL corresponds to a group of lymphoid malignancies composed of large cells with vesicular nuclei, prominent nucleoli, basophilic cytoplasm and a high proliferation rate [9]. DLBCL is usually aggressive, characterized by the appearance of rapidly growing tumors in lymph nodes, spleen, liver, bone marrow or other organs [10]. Nearly 90 % of aggressive mature B-cell NHL tumors in the Western world are identified as DLBCL [6, 7]. More than half of DLBLC patients can be cured with current multi-agent chemo-, radio- and/or immunotherapeutic regimes, combined with or without autologous stem cell transplantation, representing one of the successes of modern cancer therapy. However, approximately 30 to 40 % of patients will develop relapsed or refractory disease that remains a major cause of morbidity and mortality in most developed areas of the world [2, 3, 6, 7].

Gene expression and genome sequencing analyses have not only increased our understanding of DLBCL subtypes and the molecular basis of chemotherapy resistance but also led to the identification of novel molecular DLBCL subsets and rational targets for drug interventions that may allow for subtype/subset-specific molecularly targeted precision medicine and personalized combinations to both prevent and treat relapsed/refractory DLBCL. Recent studies identified several novel potential drug targets such as, the BET bromodomain protein 4, phosphoribosyl-pyrophosphate synthetase 2, macrodomain-containing mono-ADP-ribosyltransferase 9, deltex-3-like E3 ubiquitin ligase, NF-kappaB inducing kinase, programmed cell death 1 and transforming growth factor beta receptor.

In the present review we give a systematic overview of the current drug targets and experimental treatments for newly diagnosed and relapsed/refractory DLBCL. We also provide comprehensive and updated lists of current drug targets and preclinical and clinical experimental studies in DLBCL, including mechanism based combinatorial studies. The use of novel approaches for relapsed/refractory DLBCL, such as antibody drug conjugates and chimeric antigen receptor-modified autologous T cells will also be discussed. A special focus is given on STAT1, ARTD9, DTX3L and ARTD8 as novel potential drug targets in distinct molecular subsets of DLBCL, respectively.

### Oncogenic pathways in DLBCL

The majority of DLBCLs are thought to arise from normal antigen-exposed B cells that are at separate stages of differentiation and are undergoing clonal expansion in the germinal center (GCs) of peripheral lymphoid organs [1–3]. DLBCLs are considered clonal malignancies that evolve and progress through a range of multistep transformation processes, i.e., chromosomal translocations through errors in the *Ig* gene remodeling processes during normal B cell differentiation [11–13].

Progression of DLBCLs to a more aggressive state either evolves slowly over time as a consequence of clonal evolution (selective growth and survival benefits of subclones) or alternatively, through the rapid outgrowth after catastrophic intracellular events that result in subclones characterized by extensive DNA rearrangements that have occurred simultaneously and that confer a significant survival advantage [3, 11, 12, 14]. Consistent with their clinical and genetic (clonal) heterogeneity, several diverse genetic abnormalities have been identified in DLBCL including aberrant somatic hypermutations, nonrandom chromosomal deletions, balanced reciprocal translocations deregulating the expression of proto-oncogene products such as BCL6, REL, BCL2 or c-MYC, and often associated with dysregulated apoptosis or defective DNA repair [2, 3, 12, 13, 15–17].

Several recent whole-genome/exome sequencing studies identified over 300 DLBCL cancer genes that are recurrently mutated in primary DLBCLs [12, 13, 15–22]. These recurrent mutations are located both in genes that are well known to be functionally relevant in DLBCL and in genes for which a functional role in DLBCL has not been previously suspected [12, 16, 17, 22]. It is thought that the primary or early oncogenic events are chromosomal translocations involving oncogenes such as *BCL6, BCL2, REL* or *c-MYC* whereas the secondary or late oncogenic events consist of clonally represented recurrent mutations/gene alterations including *BCL2, PRDM1, CARD11, MyD88, TNFAIP3, CREBBP, TP53, EZH2, MLL2, MYOM2, PIM1, LYN, CD36, B2M, CD79B, MEF2B, ANKLE2, KDM2B, HNF1B, NOTCH1/2, DTX1 and MYCCD58* [12, 13, 15–22]. Moreover, alterations in a variety of DNA repair and DNA damage signaling genes, such as *ARTEMIS, DNA-PKS, KU80, KU70, CHECK2 or ARTD1/PARP1* that affect the MMR and/or NHEJ DNA repair pathways have been recently identified in DLBCL tumors and most likely also constitute intermediate cancer driver events in lymphomagenesis [23, 24]. Overexpression of proto-oncogene products through mutation or translocation of *BCL6, BCL2, REL,* or *c-MYC,* constitutive activation of canonical and/or non-canonical nuclear factor kappa B (NF-κB) pathways through genetic lesions and mutations in *TNFAIP3, CARD11, CD79A/B, MyD88* or *TRAF2* and *TRAF3* genes, respectively [15–18, 25–27], and/or epigenetic reprogramming, triggered by mutations in genes such as *TET1, MLL2, EZH2, MEF2B, EP300* and *CREBBP* [15–17, 19, 20, 28–30], account for some of the most frequent cancer driver events in DLBCL [2]. The alterations in gene expression of proto-oncogene products and/or tumor suppressors provide tumor cells with gene expression plasticity, escape from apoptosis and enhanced growth through constitutive survival and proliferative signals. See next sections. For a detailed description of oncogenic pathways in DLBCL, the readers are referred to the recent excellent reviews [2, 3, 31–36].

### Distinct disease entities and molecular subtypes of DLBCL

Based on the morphological, biological pathological, and/or clinical grounds, DLBCL has been subdivided into four distinct categories and disease entities within the 4th. Edition of the World Health Organization (WHO) Classification of Tumors of Hematopoietic and Lymphoid Tissues (2008) [1, 9, 37, 38]: 1.) DLBCL with a predominant extranodal location, including primary mediastinal (thymic) large B-cell lymphoma (PMLBCL), 2.) Large cell lymphomas of terminally differentiated B-cells, 3.) B-cell neoplasms with features intermediate between DLBCL and other lymphoid tumors, including B-cell neoplasm with features intermediate between DLBCL and Burkitt lymphoma (DLBCL/BL) and 4.) The biologically and clinically heterogeneous and therefore collectively termed DLBCL, not otherwise specified (DLBCL-NOS) [37, 39–41].

**Fig. 1:**
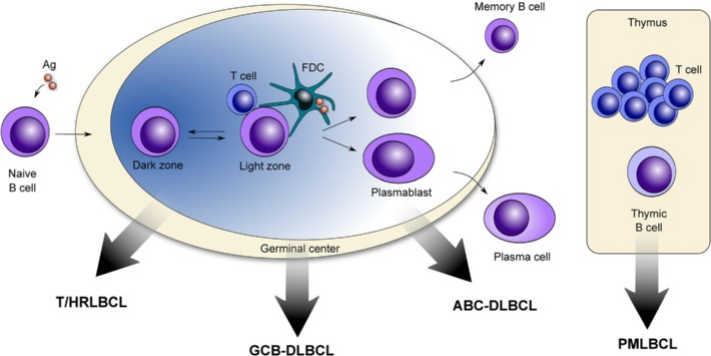
Origins of germinal center (GC)-derived and non-GC derived molecular subtypes of DLBCL. Germinal center (GC)-derived molecular subtypes of B-cell lymphoma originate from GC B cells that are blocked at different stages of development and have distinctive mechanisms of oncogenic activation and different clinical outcomes. T cell/histiocyte-rich large B-cell lymphoma. (T/HRLBCL) is thought to originate from a progenitor cell of germinal center origin. T/HRLBCLs are characterized by scattered large B cells immersed in a T-cell rich background with frequent presence of histiocytes. GC B cell (GCB)-like diffuse large B-cell lymphoma (DLBCL) originate from light zone B cells. Activated B cell-like (ABC) DLBCL shows characteristics of the late post–germinal center plasmablasts, a normally transient state that is committed to terminal plasmacytic differentiation. Primary mediastinal (thymic) large B-cell lymphomas (PMLBCL) shows characteristics of late post-germinal center thymic B cells and are thought to originate from thymic asteroid medulla B cells. Adapted from REF [3, 70, 86]. Information for this figure was gleaned from the following references: references: T/HRLBCL [37, 38, 42, 43, 45–47, 111, 112, 116], GCB-DLBCL [2, 3, 13, 35, 39, 40, 42, 43, 65–74, 86], ABC-DLBCL [2, 3, 13, 35, 39, 40, 42, 43, 65, 66, 70, 86, 87, 96] and PMLBCL [1, 3, 9, 35, 48, 49, 51, 52, 62, 63, 70, 86]. Abbreviations: FDC follicular dendritic cell, Ag antigen

Gene-expression profiling (GEP) and cell-of-origin (COO) studies (cell-of-origin signatures) have confirmed the physiological heterogeneity of the disease and defined at least three molecular subtypes of DLBCL: Primary mediastinal (thymic) large B-cell lymphoma (PMLBCL), belonging to the WHO category DLBCL with a predominant extranodal location [37] and two subtypes, belonging to the WHO category DLBCL-NOS [9]. The DLBCL-NOS category has been subdivided based on the origin and gene expression signature (COO signature) into at least two/(three) molecular, biologically and clinically distinct subtypes: the two fully classified subtypes germinal center B-cell-like (GCB)-DLBCL and activated B-cell-like (ABC)-DLBCL, the not yet fully classified T cell/histiocyte-rich large B-cell lymphoma (T/HRLBCL) and cases, that remain non-classified, termed type-3 or NC-DLBLCL [35, 39, 40, 42–44]. These DLBCL-NOS subtypes originate from B cells at different stages of development and have distinctive mechanisms of oncogenic activation and different clinical outcomes (Fig. 1) [2, 3, 9, 35, 39, 40, 42, 43]. A summary of clinical, pathological, and molecular characteristics of the molecular subtypes of DLBCL is shown in Table 1. GCB and ABC subgroups represent up to 45 % and 35 %, respectively, of DLBCL-NOS cases [1, 35]. The not yet fully classified T/HRLBCLs account for roughly 1–5 % of all DLBCL cases [1, 45–47] whereas the residual 15–20 % of DLBCL are unclassifiable DLBCLs, including type-3-DLBCL [1, 35].

**Table 1:** Summary of clinical, pathological, and molecular characteristics of the molecular subtypes of DLBCL

Molecular subtype	Immuno phenotype	Presume d cell of origin	Diagnostic molecular features	Etiology	Frequency^a^	Clinical features
T/HRLBCL	CD20, CD45,k (CD79A/B), CD15^—^, (CD30^—^),	Progenitor cell of germinal center B- cell	Gene expression profiling	Unknown	~1–4 %	Median age: 30–50
						Gender dominance: male
						Extranodal (vague nodularity is rare)
	BOB1, PU.1^+/—^, CT2, PAX5, (BCL2), BCL6, IRF4^—^					Diffuse pattern with predominance of reactive T cells
						Bone marrow involvement: ~ 30 %
						Survival ~ 50 % at 5 years
						Curable ~ 45 %
GCB-	CD20, CD10, CD22, (CD30), (BCL2), (MYC), BCL6, GCET, HGAL, LMO2, PTEN^+/—^, IRF4^—^, FOX ^—^,	Germinal center B-cell	Gene expression profiling	Unknown	~17 %	Median age 61
DLBCL						Gender dominance: male ≥ female
						Nodal and extranodal
						Bone marrow involvement: ~ 15 %
						Survival ~ 60 % at 5 years
						Curable ~ 50 %
ABC-	CD20, CD22, (CD30), (CD79A/B), IRF4, FOXP1, (BCL2), (MYC), BCL6^+/—^, GCET^—^, LMO2^—^,	Post-germinal center B- cell	Gene expression profiling	Unknown	~15 %	Median age: 66
DLBCL						Gender dominance: male ≥ female
						Nodal and extranodal
						Bone marrow involvement: ~ 15 %
						Survival ~ 40 % at 5 years
						Curable ~30 %
PMLBCL	CD19, CD20, CD22, CD30, CD45, CD79A, CD3^—^, CD10^—^, CD21^—^ BOB1, PU.1, OCT2, PAX5, BCL6, IRF4, c-REL, TRAF1	Post- thymic B- cell	Gene expression profiling	Unknown	~6 %	Median age: 33
						Gender dominance: female Mediastinal, thoracic > nodal Bone marrow involvement: rare Survival: > 60 % at 5 years
						Curable: > 60 %

The PMLBCL, GCB and ABC subtypes are histologically indistinguishable and require gene expression profiling (GEP) to be discerned. T/HRLBCL also shows overlapping histological features of other distinct entities and may appear similar to other lymphoid diseases, such as nodular lymphocyte-predominant Hodgkin lymphoma, classical Hodgkin lymphoma, and peripheral T-cell lymphoma. Diagnosis of this entity is occasionally difficult and requires careful immunohistochemical analysis of the tumor cells. GEP can greatly help to classify T/HRLBCL.

^a^Approximate percentage of the particular DLBCL subtype among all patients with B-cell lymphoma. Adapted from REF: [3, 70, 86]. Information for this table was gleaned from the following references: [1–3, 15, 19, 20, 29, 35, 37–40, 45–48, 50, 51, 56, 62, 63, 65–70, 75, 86, 87, 107, 108, 111, 112, 116].

*DLBCL* diffuse large B cell lymphoma, *T/HRLBCL* T cell/histiocyte-rich large B-cell lymphoma, *GCB* Germinal center B cell-like, *ABC* Activated B cell-like, *PMLBCL* primary mediastinal (thymic) large B-cell lymphomas

### Primary mediastinal (thymic) large B-cell lymphoma (PMLBCL)

PMLBCL constitutes roughly 2–4 % of B-cell NHL and 8 % of DLBCLs, respectively [1, 48]. It shares morphologic features with DLBCL and some features of classical Hodgkin’s lymphoma (cHL) [1, 9, 35, 48–50]. PMLBCL is characterized by a diffuse proliferation of medium-to-large B cells associated with sclerosis [51]. The presence of somatic hypermutations suggests a germinal or post-germinal center origin for PMLBCL [1, 48, 49, 52]. The similar pattern of mutations of *IGVH* and *BCL6* genes found in thymic B cells and PMLBCLs further supports the theory that PMLBCL originates from thymic B cells [52]. Thus, PMLBCLs is a distinct entity and thought to stem from thymic asteroid medulla B cells of the thymus [51]. PMLBCL normally affects young women with the mediastinum being the predominant site of lymphoma manifestation [48]. PMLBCLs have a gene expression signature that is distinct from other forms of DLBCL but closely resemble that of cHL [53–55]. Over 30 % of all PMLBCL signature genes were also more highly expressed in cHL [53–55]. This is characterized by constitutive activation of the NF-κB signaling pathway [53–55], which in part, acts through c-REL containing NF-κB transcriptional complexes [56]. The *SOCS1* gene, a suppressor of Janus kinase (JAK)-2 signaling is recurrently deleted in PMLBCL [57, 58].

The expression of interleukin (IL)-13-receptor and downstream effectors JAK2 and signal transducer and activator of transcription (STAT)-1 as well as their activities are both up-regulated in PMLBCL [53–55]. Proliferation and survival of PMLBCL cells relies on JAK/STAT and NF-κB signaling [59, 60]. Like cHL, PMLBCLs show low levels of expression of multiple components of the B cell (antigen) receptor (BCR)-signaling cascade [53–55]. Moreover, constitutive STAT6 activation has been identified as a characteristic feature of PMLBCL compared with other DLBCL subgroups [3, 53–55, 61]. A summary of the characteristic molecular features of PMLBCL is shown in Table 2. For a detailed description of the biology and pathology of PMLBCL as well as diagnosis and treatment options, the readers are referred to the recent excellent reviews [1, 48, 50, 51, 62–64].

**Table 2:** Summary of the major characteristic molecular features of PMLBCL

Genetic Aberration: Gain of function	Genetic Aberration: Loss of function	Pathways: Upregulated/Gain	Pathways: Inhibited/Loss	Study
JAK2 (9p24.1 Ampl)	SOCS1 (Mut)	JAK/STAT (**STAT1**, *STAT3*, STAT6) **c-MYC** PD-1/PD-L1/2		[57, 60, 603–605]
STAT6 (Mut)	PTPN1 (Mut)	STAT6	PTPN1	[558, 606]
JMJD2C (9p24.1 Ampl)		**c-MYC**		[603]
CD274 (PD-L1) (Tx/ 9p24.1Ampl) PDCD1LG2 (PD-L2) (Tx/Ampl)		PD-1		[607, 608]
	*TNFAIP3 (A20) (Mut)*	*NF-κB* (c-REL), TRAF1	*A20*	[56, 609]
	CIITA (Tx)	PD-1	HLA II complex	[64, 610]
	**TP53** (Mut)		TP53	[611]

Overlapping molecular features of PMLBCL and ABC-DLBCL are marked as italic. Overlapping molecular features of PMLBCL, GCB-DLBCL and ABC-DLBCL are marked as bold. Normal letters refer to genetic aberrations that contribute to DLBCL pathogenesis, regardless of subtype.

*DLBCL* diffuse large B cell lymphoma, *ABC* activated B cell-like, *GCB* germinal center B cell-like, *PMLBCL* primary mediastinal (thymic) large B-cell lymphomas, *Del* deleted/deletions, *Mut* mutated/mutations, *Ampl* amplified/amplification, *Tx* translocations, *PRDM1/BLIMP1* B lymphocyte-induced maturation protein 1, *PD-L1/L2* programmed cell death ligand 1/2, *JMJD2C* jumonji domain containing 2c, *PTPN1* phosphotyrosine phosphatase N1, *SOCS1* suppressor of cytokine signaling 1, *TNFAIP3* tumor necrosis factor, alpha-induced protein, 3, *NF-κB* nuclear factor-kappa B, *TRAF* TNF receptor-associated factor, *STAT* signal transducer and activator of transcription, *HLA* human leukocyte antigens

### Germinal center B cell-like (GCB)-DLBCL-NOS

**Fig. 2:**
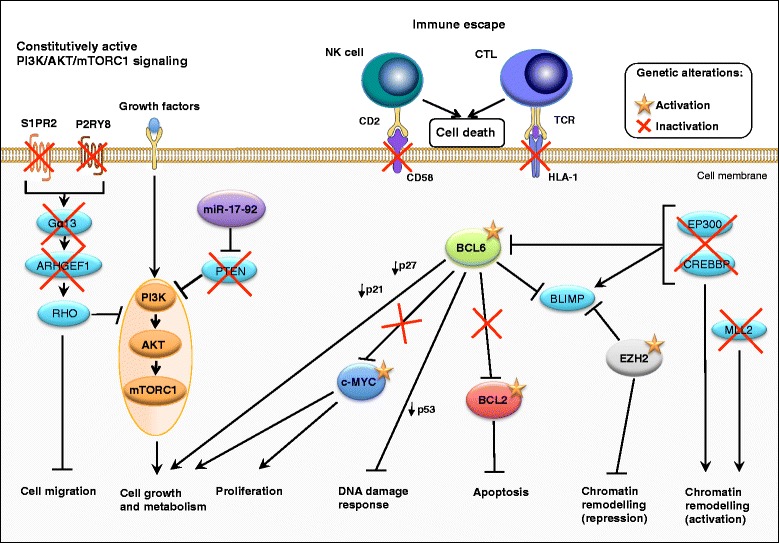
Oncogenic (deregulated) intracellular signaling pathways in germinal center B cell-like (GCB) diffuse large B-cell lymphoma (DLBCL). Germinal center B cell-like diffuse large B-cell lymphoma (GCB-DLBCL) are characterized by the inactivation or loss of the phosphatase and tensin homologue (PTEN) and S1PR2-Gα13-ARHGEF1 signaling pathways, which both negatively modulate GC B cell migration and phosphatidylinositol-3-kinase (PI3K) signaling [13, 82–84, 324, 593]. In addition, activating mutations of the histone methyltransferase EZH2 (enhancer of zeste homologue 2) and master regulator of the GCB phenotype promote epigenetic changes that, at least partly, cooperate with the B-cell lymphoma 2 (BCL2) and BCL6 to mediate lymphomagenesis in GCB-DLBCL [77, 79]. A subset of GCB-DLBCL is characterized by translocations affecting the *c-MYC* and/or the *BCL2* loci [80, 81, 87]. These translocations lead to constitutive activation of c-MYC and the anti-apoptotic BCL2 protein and to a malignant transformation by preventing terminal differentiation or blocking apoptosis. The different colors that are used in the figure indicate molecules that belong to a specific pathway and/or lead to a specific outcome. Adapted from REF: [3, 70, 86, 109]. Information for this figure was gleaned from the following references: [2, 3, 13, 15–18, 20, 29, 39, 66, 75–84, 86, 87, 93, 109, 123, 148, 247–251, 259, 324, 325, 352, 354–356, 360–365, 367, 374, 375, 377, 380–382, 384–386, 593, 612]. Abbreviations: S1PR2 sphingosine-1-phosphate receptor-2, Gα13 (GNA13) heterotrimeric G protein alpha 13, ARHGEF1 Rho guanine nucleotide exchange factor (GEF) 1, PI3K phosphoinositide 3-kinase, mTORC1 mammalian target of rapamycin (mTOR) complex 1, BCL6 B cell lymphoma protein 6, BCL2 B cell lymphoma protein 2, EZH2 enhancer of zeste homologue 2

GCB-DLBCLs are thought to arise from normal germinal center B cells [65] and show features that are consistent with germinal center B cell derivation [13, 66–70]. GCB-DLBCLs largely express gene products, such as BCL6, HGAL and LMO2 [13, 65–69] that define normal germinal center B cells within the germinal center light zone [71]. Malignant GCB-DLBCL clones continue to undergo somatic hypermutation of their variable immunoglobulin heavy chain gene and have often switched IgH classes that are mediated by AID, an enzyme that is characteristically expressed at high levels in germinal center B cells [72–74]. The GCB-DLBCL subtype is characterized by low level of NF-κB activation and its survival is not dependent on NF-κB [13, 18]. Various oncogenic pathways are deregulated in GCB-DLBCL and contribute to its molecular pathogenesis. Oncogenic (deregulated) intracellular signaling pathways in GCB-DLBCL are summarized in Fig. 2.

Translocations of *BCL2* and/or c-*MYC* genes are commonly observed in GCB-DLBCLs [13, 66, 75]. These translocations lead to constitutive activation of c-MYC and the anti-apoptotic BCL2 protein [76] and to a malignant transformation by preventing terminal differentiation or blocking apoptosis [2, 3]. 20 % have gain of function mutations of the histone methyltransferase EZH2, which is a master regulator of the GCB phenotype and cooperates, at least partly, with BCL2 and BCL6 to mediate lymphomagenesis in GCB-DLBCL [20, 29, 77–79]. GCB-DLBCL is furthermore characterized by downregulation of the phosphatase and tensin homologue (PTEN) and concomitant upregulation of phosphatidylinositol-3-kinase (PI3K) signaling pathway (Fig. 2) [13]. Upon loss of the *PTEN* gene or repression of the *PTEN* promoter through the mir-17-92 microRNA cluster, phosphatidylinositol-3 phosphate accumulates and AKT and mTORC1 are activated, further promoting cell survival, proliferation, and growth [13, 80–83]. GCB-DLBCL is also associated with loss of sphingosine-1-phosphate receptor-2 (S1PR2) - G-protein alpha 13 (Gα13) signaling, which negatively modulates GC B-cell migration and PI3K signaling [15–17, 84].

The GCB-DLBCL subtype also frequently carries additional mutations affecting epigenetic modification such as mutations in genes encoding the histone acetyltransferases CREB-binding protein (CREBBP) and E1A-binding protein p300 (EP300) as well as the histone-lysine N-methyltransferase and myeloid/lymphoid or mixed-lineage leukemia protein 2 (MLL2) [15, 19, 20, 29]. Other characteristic features of GCB-DLBCLs are the amplification of MDM2, a negative regulator of the tumor suppressor TP53, as well as deletions of the tumor suppressor gene TP73 [13]. A summary of the characteristic molecular features of GCB-DLBCL is shown in Table 3. The GCB subtype has a cure rate of about 70 to 80 % with currently available therapies [13, 85]. For a more detailed description of the biology and pathology of GCB-DLBCL, the readers are referred to the recent excellent reviews [2, 35, 39, 70, 86].

**Table 3:** Summary of the major characteristic molecular features of GCB-DLBCL-NOS

Genetic Aberration: Gain of function	Genetic Aberration: Loss of function	Pathways: Upregulated/Gain	Pathways: Inhibited/Loss	Study
***BCL2 (Tx/Ampl/Methyl)***		***BCL2***	***Pro-apoptotic signaling***	[75, 362, 365]
EZH2 (Mut)		EZH, ***BCL2***, ***BCL6***	***BLIMP1***, ***CREBBP***; ***EP300***	[16, 17, 77–79]
***BCL6 (Tx/Ampl/Methyl)***		***BCL6***	***BLIMP1***, ***CREBBP***; ***EP300***	[87, 93, 148, 386, 612]
***MEF2B/(MEF2C) (Mut)***		***BCL6***	***BLIMP1***,*** CREBBP***; ***EP300***	[16, 17, 28]
MDM2 (Ampl)	**TP53 (Del/Mut),** ***TP73 (Del)***		**TP53** ***and TP73Pro-apoptotic signaling***	[16, 17, 155, 613, 614]
***c-MYC (Tx/Ampl/Methyl)***		**c-MYC**, miR-17-92, BCR, PI3K/AKT/mTORC1	ISGs/IFITs, PTEN	[80, 81, 87]
miR-17-92		BCR, PI3K/AKT/mTORC1	ISGs/IFITs, PTEN	[13, 80, 81]
	P2RY8 (Mut)	PI3K/AKT/mTORC1	(Mut)	[15–17, 84]
	S1PR2 (Mut)	PI3K/AKT/mTORC1	(Mut)	[15–17, 84]
	GNA13 (Mut)	PI3K/AKT/mTORC1	Gα13	[15–17, 84]
	ARHGEF1 (Del/Mut)	PI3K/AKT/mTORC1	ARHGEF1	[15–17, 84]
	PTEN (Del/Methyl/miR)	PI3K/AKT/mTORC1	PTEN	[13, 82, 83, 593]
	***B2M (Del/Mut)***		***B2M***, ***HLA-I complex***	[16, 17, 615, 616]
	***CD58 (Del/Mut)***		***CD58***, ***HLA-I complex***	[16, 17, 615, 616]
	***MLL2***, ***MLL3 (Mut)***	***BCL6***	***MLL2/3***, ***CREBBP***; ***EP300***	[12, 16, 17, 20]
	***CREBBP***; ***EP300 (Del/Mut)***	***BCL6***, ***HSP90***,	***CREBBP***; ***EP300***, ***BLIMP1***, **TP53**	[16, 17, 19, 386, 388]
	TNFRSF14 (Mut)		HVEM/FAS	[16, 17, 617]
	FAS (Mut)		FAS/ Pro-apoptotic signaling	[16, 618]
	***TRAF3 (Del/Mut)***	***Non-canonical NF-κB signaling (p52/RELB)***,	??	[26, 27]
??	??	**STAT1**		[470]

Overlapping molecular features of GCB- and ABC-DLBCL are marked as bold italic. Overlapping molecular features of PMLBCL, GCB-DLBCL and ABC-DLBCL are marked as bold. Normal letters refer to genetic aberrations that contribute to DLBCL pathogenesis, regardless of subtype.

*DLBCL* diffuse large B cell lymphoma, *ABC* activated B cell-like, *GCB* germinal center B cell-like, *PMLBCL* primary mediastinal (thymic) large B-cell lymphomas, *Del* deleted/deletions, *Mut* mutated/mutations, Ampl amplified/amplification, *Tx* translocations, *BCL2* B-cell lymphoma protein 2, *BCL6* B-cell lymphoma protein 6, *CREBBP* CREB-binding protein, *EP300* E1A-binding protein p300, *EZH2* enhancer of zeste homologue 2, *NF-κB* nuclear factor- kappa B, *PRDM1/BLIMP1* B-lymphocyte-induced maturation protein 1, *TNFAIP3* tumor necrosis factor, alpha-induced protein 3, *TRAF* TNF-receptor-associated factor, *MLL* myeloid/lymphoid or mixed-lineage leukemia, *TNFRSF14* tumor necrosis factor receptor superfamily, member 14, *MDM2* murine double minute E3 ubiquitin protein ligase 2, *STAT* signal transducer and activator of transcription*, P2RY8* purinergic receptor P2Y, G-protein coupled 8, *S1PR2* sphingosine-1-phosphate receptor 2, *MEF2B* myocyte enhancer factor 2B, *PTEN* phosphatase and tensin homolog, *FAS* TNF receptor superfamily, member 6, *HLA* human leukocyte antigens, *ISGs/IFITs* interferon stimulated genes/interferon inducible transcription factors

### Activated B cell-like (ABC)-DLBCL-NOS

The second subtype, ABC-DLBCL has experienced the germinal center and is thought to arise from post-germinal center B cells that are arrested during plasmacytic differentiation [13, 65, 70, 87]. ABC-DLBCLs have a COO signature that is reminiscent of the plasmablast stage of B cell development [65]. ABC-DLBCLs largely express genes normally induced during *in vitro* activation of peripheral blood B cells, including genes that define the end stage memory B cells or plasma cells, such as the transcription factors IRF4 and XBP1 [13, 65, 66, 87]. IRF4 is transiently expressed during normal lymphocyte activation and is essential for the proliferation of B cells in response to signals from the activated B cell (antigen) receptor [88, 89]. Thus, constitutive expression of interferon response factor (IRF)-4 in ABC-DLBCLs contributes to unchecked proliferation of DLBCL tumors [90, 91]. X-box binding protein 1 (XBP1) is the master regulator of immunoglobulin secretion [35, 92]. On the other hand, ABC-DLBCLs acquire genetic alterations that repress BLIMP1 expression and function, thereby blocking the differentiation into end stage plasma cells [92–94]. ABC-DLBCL is characterized by genetic abnormalities that play an important role in its pathogenesis. For instance, the p16INK4A/p14ARF tumor suppressor locus is deleted in approximately 30 % of ABC-DLBCLs and is associated with inferior outcome within this subtype [13, 95]. A summary of characteristic molecular features of ABC-DLBCL is shown in Table 4.

**Table 4:** Summary of the major characteristic molecular features of ABC-DLBCL-NOS

Genetic Aberration: Gain of function	Genetic Aberration: Loss of function	Pathways: Upregulated/Gain	Pathways: Inhibited/Loss	Study
MyD88 L265P (Mut) *MyD88* (other)		TLR/MyD88 *NF-κB*, IRF4	IRF7/IFNβ (Type 1 interferon)	[16–18]
CD79A, CD79B (Mut)		B-cell receptor *NF-κB*, IRF4,	IRF7/IFNβ (Type 1 interferon)	[16, 17]
CARD11 (Mut)		*NF-κB*, IRF4	IRF7/IFNβ (Type 1 interferon)	[16–18]
	***TRAF3 (Del/Mut)***	*Canonical NF-κB (p65/p50) and non-canonical NF-κB (p52/RELB) signaling*, IRF4	IRF7/IFNβ (Type 1 interferon)	[16–18, 26, 27, 160]
	TRAF2 (Del/Mut)			
SPIB (Tx/Mut)		CARD11/*NF-κB*, IRF4	IRF7/IFNβ (Type 1 interferon)	[13, 16, 17, 87, 90]
REL (Ampl)		_	_	[619]
***BCL2 (Tx/Ampl/Methyl)***		***BCL2***	***Pro-apoptotic signaling***	[362]
***MYC (Tx/Ampl/Methyl)***		**c-MYC**		[87, 107]
***BCL6 (Tx/Ampl/Methyl)***		***BCL6***	***BLIMP1***, ***CREBBP***; ***EP300***	[87, 93, 148, 386, 612, 620]
***MEF2B (Mut)***		***BCL6***	***BLIMP1***, ***CREBBP***; ***EP300***	[13, 16, 17, 28]
STAT3 (Mut)		IL6/10, *JAK/STAT3*		[307, 316]
??	??	**STAT1**		[470]
FOXP1 (Ampl)		Wnt/β-catenin	**TP53/Pro-apoptotic signaling**	[13, 621, 622]
	**TP53 (Del/Mut)**		**TP53** ***and TP73Pro-apoptotic signaling***	[16, 17, 155, 614
	***TP73 (Del)***			
	***B2M (Del/Mut)***		***B2M***, ***HLA-I complex***	[16, 17, 615, 616]
	***CD58 (Del/Mut)***		***CD58***, ***HLA-I complex***	[16, 17, 615, 616]
	***MLL2***, ***MLL3 (Mut)***		***MLL2***, ***CREBBP***; ***EP300***	[12, 16, 17, 20]
	***CREBBP***; ***EP300 (Del/Mut)***	***BCL6***, *STAT3*, ***HSP90***	***CREBBP***; ***EP300***, ***BLIMP1***, ***TP53***	[17–19, 386, 388, 392]
	CDKN2A/B (Del/Methyl)		P16INK4a/pRB p14ARF/TP53	[13, 120]
	TNFAIP3 (Del/Mut)	*NF-κB*, IRF4	*A20*	[13, 16–18]
	PRDM1 (Del/Mut)		BLIMP1	[13, 16, 17]

Overlapping molecular features of ABC-DLBCL and PMLBCL are marked as italic and those of GCB- and ABC-DLBCL are marked as bolditalic. Overlapping molecular features of PMLBCL, GCB-DLBCL and ABC-DLBCL are marked as bold. Normal letters refer to genetic aberrations that contribute to DLBCL pathogenesis, regardless of subtype.

*DLBCL* diffuse large B cell lymphoma, *ABC* activated B cell-like, *GCB* germinal center B cell-like, *PMLBCL* primary mediastinal (thymic) large B-cell lymphomas, *Del* deleted/deletions, *Mut* mutated/mutations, *Ampl* amplified/amplification, *Tx* translocations, *BCL2* B-cell lymphoma protein 2, *BCL6* B-cell lymphoma protein 6, *NF-κB* nuclear factor-kappa B, *TLR* Toll like receptor, *MyD88* myeloid differentiation primary response 88, *CARD11* caspase recruitment domain family, member 11, *IRF4* interferon-regulatory factor 4, *CREBBP* CREB-binding protein, *EP300* E1A-binding protein p300, *EZH2* enhancer of zeste homologue 2, *PRDM1/ BLIMP1* B-lymphocyte-induced maturation protein 1, *TNFAIP3* tumor necrosis factor, alpha-induced protein 3, *TRAF* TNF-receptor-associated factor, *MLL* myeloid/lymphoid or mixed-lineage leukemia, *CDKN1A* cyclin-dependent kinase inhibitor 1A, *MDM2* murine double minute E3 ubiquitin protein ligase 2, *STAT* signal transducer and activator of transcription, *FOXP1* forkhead box P1, *IRF7* interferon-regulatory factor 7, *IFNβ* interferon beta, *MEF2B* myocyte enhancer factor 2B, *FAS* TNF receptor superfamily, member 6, *HLA* Histocompatibility antigen, domains alpha

**Fig. 3:**
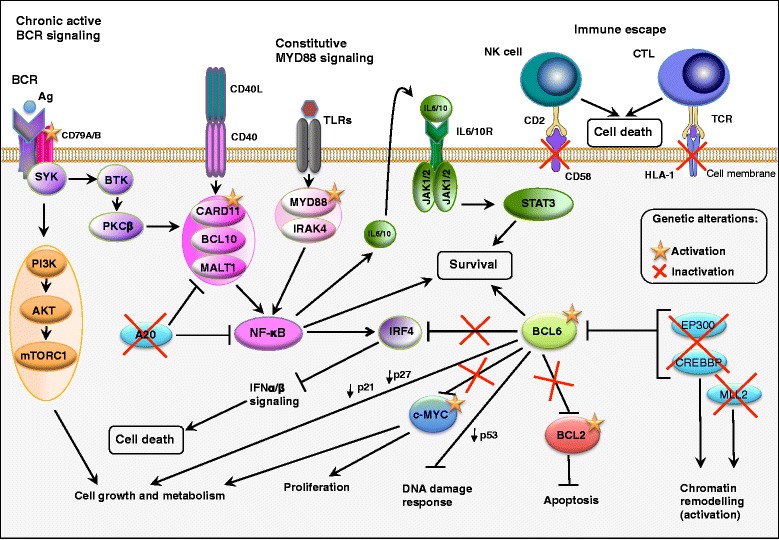
Oncogenic (deregulated) intracellular signaling pathways in activated B cell-like (ABC) diffuse large B-cell lymphoma (DLBCL). The oncogenic constitutive activation of canonical nuclear factor-kappa B (NF-κB) family members in activated B cell-like diffuse large B-cell lymphoma (ABC-DLBCL), together with a blockade in terminal B cell differentiation, which is in part mediated by BCL6, represent the hallmarks of ABC-DLBCL pathogenesis [13, 16–18, 102]. The different colors that are used in the figure indicate molecules that belong to a specific pathway and/or lead to a specific outcome. Adapted from REF: [3, 70, 86, 109]. Information for this figure was gleaned from the following references: [2, 3, 12, 13, 15–20, 27, 29, 54, 60, 70, 75–81, 86, 87, 90, 91, 93, 96–100, 103, 104, 107, 109, 123, 133, 148, 160, 161, 171, 232, 233, 247–251, 254–257, 259, 271, 285, 286, 307–311, 313–317, 360–365, 367, 374, 375, 377, 380–382, 384–386, 572, 612, 620]. Abbreviations: BCR B cell receptor, BTK Bruton’s tyrosine kinase, SYK spleen tyrosine kinase, PI3K phosphoinositide 3-kinase, mTORC1 mammalian target of rapamycin (mTOR) complex 1, CD40L CD40 ligand, JAK Janus kinase, IRF4 interferon-regulatory factor 4, MALT1 mucosa-associated lymphoid tissue lymphoma translocation protein 1, BCL10 B cell lymphoma protein 10, TLR Toll-like receptor, MyD88 myeloid differentiation primary response 88, CARD11 caspase recruitment domain family, member 11, PKCβ protein kinase Cβ, STAT3 signal transducer and activator of transcription 3, BCL6 B cell lymphoma protein 6, BCL2 B cell lymphoma protein 2

ABC-DLBCLs express genes that are upregulated in B cells with activated BCR signaling [13, 96] see also next sections. BCR-mediated NF-κB-dependent survival signaling plays important roles in certain B-cell malignancies [97–99]. Similar to PMLBCL, a key feature of the more aggressive ABC-DLBCL subtype is the constitutive activation of NF-κB-dependent gene expression and its dependency on the activity of NF-κB family members for proliferation and survival [18, 54, 66, 76, 100]. NF-κB is a family of inducible transcription factors consisting of five members, REL-A (p65), REL-B, c-REL, NF-κB1 (p50 and its precursor p105), and NF-κB2 (p52 and its precursor p100) [101]. Inhibition of this pathway using either a dominant active form of NF-κB inhibitory protein inhibitor of kappa B (IκB)-α or a specific IκB kinase inhibitor is toxic to ABC- but not to GCB-DLBCL cell lines [59, 100]. The NF-κB family members REL-A, REL-B and c-REL have the capacity to regulate transcription of various subsets of genes involved in cell proliferation and resistance to apoptosis [102]. ABC-DLBCLs shows a more restricted, potentially developmentally regulated NF-κB target gene signature [54, 76, 100]. Activation of NF-κB has been identified as a key driver in apoptosis resistance in ABC-DLBCL and PMLBCL leading to poor outcomes in patients with ABC-DLBCL [18, 54, 100]. In 39 % of ABC-DLBCL cases, constitutive activation of NF-κB-dependent gene expression is activated by somatic, gain-of-function mutations in the myeloid differentiation primary response gene (88) (*MyD88*) [103]. The most common MyD88 mutant, L265P, spontaneously coordinates a signaling complex in which interleukin-1 receptor-associated kinase (IRAK)-4 phosphorylates IRAK1, leading to inhibitor of kappa B (IκB) kinase (IKK) and NF-κB activation [103]. In addition, 10 % of ABC-DLBCL patients harbor activating mutations in the gene encoding caspase recruitment domain-containing protein 11 (CARD11) leading to constitutively active NF-κB family members [104]. CARD11 is a key signaling adaptor that coordinates a BCR and CD40-mediated signaling complex that activates NF-κB pathways [105, 106]. ABC-DLBCLs with wild-type CARD11 depend on constitutively active BCR signaling [104]. Both wild-type and mutant CARD11 are essential for chronic active BCR signaling and survival in ABC-DLBCL [103]. Approximately, 20 % of ABC-DLBCLs have mutations in the BCR signaling molecules CD79B and CD79A [66, 96, 104], which lead to constitutively active BCR signaling [66, 96]. Oncogenic (deregulated) intracellular signaling pathways in ABC-DLBCL are summarized in Fig. 3. Translocations of both *BCL2* and c-*MYC* genes also occur in ABC-DLBCLs and contribute to the inferior survival of the ABC subtype of DLBCL [107]. Both GCB-DLBCL and ABC-DLBCL share genetic lesions that lead to inactivation of chromatin modifiers, owing to mutations in CREBBP, EP300 and MLL2 [15, 19, 20, 29], as well as to immune escape, owing to inactivation of β2 microglobulin (B2M), CD58 and genes encoding human leukocyte antigens (HLA-A, HLA-B and HLA-C) [15, 19, 20, 29, 108]. The ABC subtype has a cure rate of about 40 to 50 % with currently available therapies [13, 85]. For a detailed description of the biology and pathology of ABC-DLBCL, the readers are referred to the recent excellent reviews [2, 3, 35, 39, 70, 86, 102, 109, 110].

### T cell/histiocyte-rich large B-cell lymphoma (T/HRLBCL)

T cell/histiocyte-rich large B-cell lymphoma (T/HRLBCL) is an aggressive DLBCL with a poor clinical outcome, similar to ABC-DLBCL [45, 47]. T/HRLBCL accounts for approximately 5 % of DLBCL [111, 112]. Many T/HRLBCL tumors, especially cases containing numerous histiocytes behave aggressively and show resistance to current therapies for DLBCL [45]. T/HRLBCL occurs in younger patients, predominantly affects men, and involves liver, spleen, and bone marrow with greater frequency than classical DLBCL [45, 111]. T/HRLBCL is thought to stem from a progenitor cell of germinal center origin [111]. T/HRLBCL is an uncommon morphologic subtype characterized by a minor population of scattered large neoplastic B cells existing in a background of predominant reactive T-lymphocytes with frequent presence of histiocytes [37, 45, 111]. No recurrent genetic rearrangements and/or mutations directly affecting the biology and pathology of T/HRLBCL have been identified yet [111–115]. According to the WHO classification 2008, T/HRLBCL is considered a separate clinically heterogeneous not yet fully classified entity [38, 45–47]. However, several recent morphological and GEP studies [42, 43, 46, 116] indicate that T/HRLBCL and the T/HRLBCL-like variant nodular lymphocyte predominant Hodgkin lymphoma (NLPHL), initially defined as distinct entities, may represent a spectrum of the same disease [42, 43]. T/HRLBCL and NLPHL were found to share rare imbalances on chromosomes 4q and 19p suggesting a similar precursor for both disorders [113, 114]. The different clinical behavior of these lymphomas may be strongly influenced by differences in the lymphoma microenvironment [42].

The T/HRLBCL associated diffuse T cell- and histiocyte-rich infiltrates are important for a tolerogenic host immune response and for escaping the T cell-mediated immune surveillance [45, 46]. Genes found to be upregulated in T/HRLBCL overlapped significantly with genes found to be related to an unfavorable immune response in a subset of follicular lymphomas and DLBCL [85, 117], particularly with genes found to be related to host inflammatory response in the host response subtype DLBCL, HR-DLBCL (see next sections) [116]. T/HRLBCL and subsets of NLPHL have therefore been suggested to represent together a distinct clinical and molecular subtype of DLBCL-NOS [42, 43]. The gene expression signature of T/HRLBCL is dominated by interferon gamma (IFNγ) and STAT1-dependent pathways and suggests a macrophage/histiocyte-activated status that is required for the tolerogenic host immune response [45, 46, 116, 118]. For a detailed description of the biology and pathology of T/HRLBCL, as well as diagnosis and treatment options, the readers are referred to the recent excellent reviews [45, 47, 111]. In the following sections we will mainly focus on the two molecular subtypes of DLBCL-NOS; GCB- and ABC-DLBCL.

### Molecular signatures associated with poor prognosis

More recent GEP studies using multiple clustering methods revealed the existence of at least seven distinct DLBCL-NOS subsets with poor prognosis. The first study identified three discrete DLBCL-NOS subsets defined by their unique transcriptional profiles (consensus cluster (CC) signature): Oxidative phosphorylation (OxPhos)-DLBCL, B-cell receptor (BCR)/proliferation (BCR)-DLBCL, and host-response (HR)-DLBCL [116]. Several other recent studies provided evidence for at least four additional molecular subsets associated with poor prognosis: c-MYC-driven (MD)-DLBCL [119], stromal-II signature-subtype DLBCL [85], *CDKN2A/2B* (9p21)-deletion signature subtype DLBCL [120] and *RCOR1-*(*TRAF3*)*-*deletion signature subtype DLBCL [121]. An overview of molecular signatures in DLBCL-NOS associated with poor prognosis is presented in Table 5.

**Table 5:** Overview of molecular signatures in DLBCL-NOS associated with poor prognosis

Molecular signature	Cell of origin (COO)	Prognosis	Molecular features	Potential drug targets	Study
Oxidative phosphorylation	GCB (46 %) ABC (18 %) type-3 (36 %) (n.c.i.)	5y OS: 53 %	Oxidative phosphorylation (↑) TCA cycle (↑) Lipogenesis (↑) Glycolytic flux (↓) Proteosomal activity (↑) BCR signaling (↓)	Fatty acid metabolism, PPARγ, γ-glutamyl cysteine synthase,	[97, 116, 122, 123, 134]
BCR/proliferation	GCB (23.4 %) ABC (53.2 %) type-3 (23.4 %) (n.c.i.)	5y OS: 60 %	BCR signaling (↑) NF-κB signaling (↑) BCL6 (↑) Proliferation genes (↑)	BTK, PI3K, mTORC1/2, BCL6, BCL2, STAT3, NF-κB	[98, 116, 133, 134, 312, 314]
Host response	GCB (46 %) ABC (18 %) type-3 (36 %) (n.c.i.)	5y OS: 54 %	TCR signaling (↑) STAT1 signaling (↑) NF-κB signaling (↑)	PD-1, STAT1, DTX3L, ARTD9 NF-κB, BCL2	[116, 134, 340, 344–346, 469, 470, 472]
C-MYC-driven	GCB (35-50 %) ABC (45-55 %) type-3 (5-10 %) (n.c.i.)	5y OS: ≤40 % (ABC!) (n.c.i.)	c-MYC (↑) Proliferation genes (↑) BCL2 (↑) (BCL6) (↑) Anti-apoptotic signaling (↑)	BRD2 and 4, PRPS2 BCL2, (BCL6)	[107, 119, 138–144, 150, 399, 414]
Stromal-II	Independent of COO(n.c.i.)	n.c.i.	Endothelial markers (↑) Key regulators of angiogenesis (↑)	CXCR4/CXCL12 axis, VEGFR2	[85]
*CDKN2A/2B* (9p21) deletion	GCB (4 %) ABC (30 %) (n.c.i.)	n.c.i.	Loss of p15INK4B, p16INK4A, p14ARF, Loss of RB1/E2F regulation, Proliferation genes (↑) Cellular metabolism (↑) Immune and inflammatory response (↓)		[13, 120, 623]
*RCOR1-*(*TRAF3*)*-*deletion	GCB (15 %) ABC (15 %) (n.c.i.)	5y OS: 55 %	Non-canonical NF-κB signaling (↑) Loss of RCOR1 signaling HDAC class II signaling (↓)	NF-κB, NIK	[27, 121, 160]

*DLBCL* diffuse large B cell lymphoma, *ABC* activated B cell-like, *GCB* germinal center B cell-like, *n.c.i*. not completely investigated, *TCA* tricarboxylic acid, *PPARγ* peroxisome proliferators activated receptor-gamma, *BCR* B cell receptor, *NF-κB* nuclear factor-kappa B, *STAT* signal transducer and activator of transcription, *BCL2* B-cell lymphoma protein 2, *BCL6* B-cell lymphoma protein 6, *PI3K* phosphoinositide 3-kinase, *BTK* Bruton’s tyrosine kinase, *NIK* NF-κB inducing kinase, *RB1* Retina blastoma protein 1, *mTORC1/2*, mammalian target of rapamycin (mTOR) complex 1 and 2, *DTX3L* deltex (DTX)-3-like E3 ubiquitin ligase, *ARTD9* ADP- ribosyltransferase-9 diphteria toxin like, *PRPS2* phosphoribosyl-pyrophosphate synthetase 2, *BRD2 and 4* bromodomain and extra terminal (BET) protein-2 and 4, *CXCL12* CXC chemokine ligand 12 (also called stromal-cell-derived factor 1, or SDF-1), *CXCR4* CXCL12 receptor, *VEGFR2* vascular endothelial growth factor receptor 2; *RCOR1* REST corepressor 1, *HDAC* histone deacetylase

### OxPhos-signature-subtype DLBCLs

The first signature identified by Monti S. et al., termed oxidative phosphorylation (OxPhos) signature, is characterized by overexpression of genes that regulate oxidative phosphorylation, mitochondrial function and the electron transport chain, such as the nicotinamide adenine dinucleotide dehydrogenase (NADH) complex and cytochrome c/cytochrome c oxidase (COX) complex as well as adenosine triphosphate (ATP) synthase components [40, 116, 122]. OxPhos-DLBCL tumors have also higher levels of the antiapoptotic BCL2 related family member, BFL-1/A1 and exhibit genetic lesions affecting the intrinsic and extrinsic apoptotic pathways [40, 116, 122]. The OxPhos-DLBCL subset does not display any chronic or active/functional BCR signaling [97] and is insensitive to inhibition of BCR survival signaling [122, 123]. OxPhos-DLBCL display enhanced mitochondrial energy transduction, greater incorporation of nutrient-derived carbons into the tricarboxylic acid cycle, and increased glutathione levels [122]. Although the exact nature of survival pathways in this group of tumors is not known, - based on findings in other cancer models- it has been suggested that the increased fatty acid metabolism observed in OxPhos-DLBCL may serve as an alternative survival pathway that is triggered by glucose deprivation or lack of glucose uptake [122]. Indeed, disturbing the fatty acid oxidation program and glutathione synthesis is selectively toxic to the OxPhos-DLBCL tumor subset [122]. 46 % of OxPhos-DLBCL tumors analyzed in theses studies were classified as GCB-, 18 % as ABC- and the remainder 36 % were designated type-3-DLBCL-NOS [116].

### BCR/proliferation-signature-subtype DLBCLs

The second subtype signature identified by Monti S. et al., termed BCR/proliferation has increased expression levels of many components of the BCR signaling cascade (CD19, Ig, CD79A, BLK, SYK, PLCγ2, and MAPK4) and enhanced BCR signaling activity [97, 98, 116]. BCR/proliferation-DLBCL tumors also show increased expression levels of cell-cycle regulatory factors, (including CDK2 and MCM family members), DNA damage response signaling factors (such as PMS2 family members, H2AX, PTIP, and TP53) as well as higher levels of various essential B cell-specific transcription factors (such as BCL6, MYC, STAT6, PAX5, OBF1 and E2A). 53.2 % of BCR-DLBCL tumors analyzed in theses studies were classified as ABC-, 23.4 % as GCB- and 23.4 % as type-3-DLBCL-NOS [116].

In normal untransformed B cells, BCR signaling is activated in an active antigen-dependent manner that initiates the germinal center response [96, 124–128]. Antigen-induced aggregation of the BCR activates BCR signaling through receptor oligomerization and phosphorylation of immunoreceptor tyrosine-based activation motifs (ITAMS) by SRC family kinases, including FYN and B lymphocyte kinase (BLK) [129, 130]. Following ITAM phosphorylation, the spleen tyrosine kinase (SYK) is then recruited to the dually phosphorylated ITAMs through its tandem SRC homology 2 (SH2) domains, resulting in SYK phosphorylation and recruitment of additional adaptor proteins and initiating downstream signaling through phosphatidylinositol-3-kinase (PI3K) [124], Brutons’s tyrosine kinase (BTK) and finally the activation of protein kinase C (PKC)-β, which in turn phosphorylates many substrates including CARD11 [105, 106]. Active antigen-dependent BCR signaling engages multiple downstream pathways, [105, 106, 129, 130].

Normal untransformed B cells also exhibit tonic, ligand (antigen)-independent, ITAM-transmitted BCR signaling, that promotes subsequent development and survival of mature B cells in the periphery [96, 124–128]. Tonic active BCR signaling engages the phosphoinositide 3-kinase (PI3K) pathway only and is also relevant to B-cell malignancies [96, 124–128, 131]. A role for tonic BCR signaling has been postulated for GCB-DLBCL based on the sensitivity of certain cell lines of this lymphoma subtype to R406, a broad range small molecule inhibitor of SYK [97]. However, genetic knockdown of proximal BCR subunits (IgM, Ig-kappa, CD79A and CD79B) killed only ABC-DLBCL with wild-type CARD11 but did not kill other lymphomas including various GCB-DLBCL cell lines [96]. Moreover, GCB-DLBCL tumors do not acquire highly recurrent mutations in the BCR signaling or canonical NF-κB pathways [96]. Thus it remains to be elucidated whether SYK is indeed essential in GCB-DLBCL or other receptors might be required for activation of the observed BCR-like signaling in GCB-DLBCL [31].

A third type of BCR signaling, termed chronic active BCR signaling, have been characterized in B-cell malignancies, which can involve mutations of BCR pathway components or be triggered by (auto-) antigens present in the tissue microenvironment [96, 124–128, 131]. Similar to the antigen-activated active BCR signaling in normal B cells chronic active BCR signaling, which typifies ABC-DLBCL, engages multiple downstream pathways, including PI3K/AKT/mTORC1 and canonical NF-κB signaling pathways [31, 96, 105, 106, 110, 124–131]. Chronic active BCR signaling is distinct from tonic BCR signaling, which stimulates the PI3K pathway but not the NF-κB signaling pathways [31]. Up to 20 % of ABC-DLBCLs have somatic gain of function mutations in the immunoreceptor tyrosine-based activation motifs (ITAM) of the BCR subunits CD79B and CD79A, which lead to chronic BCR signaling [66, 96, 104]. However, CD79A/B mutants do not initiate BCR signaling de novo when introduced into heterologous cells, but rather increase the amplitude of ongoing BCR signaling through increased BCR surface expression and attenuation of LYN kinase activity, a negative regulator of BCR signaling [66, 96]. LYN functions as a feedback inhibitor of BCR-stimulated signaling [132]. LYN is frequently mutated and inactivated in ABC-DLBCL [21]. This new pathogenetic mechanism in ABC-DLBCL was therefore termed chronic active BCR signaling [96]. Certain BCR-dependent ABC-DLBCLs also exhibit constitutive PI3K activation, which modulates downstream NF-κB signaling [98, 133].

### HR-signature-subtype DLBCLs

Several studies have shown that differences in the tumor microenvironment of DLBCL affect survival after treatment with rituximab-based chemotherapeutic regimens [85, 116, 134, 135]. HR-DLBCL was identified by a microenvironment gene expression signature and is associated with increased expression of inflammatory mediators, such as multiple components of the T-cell receptor (TCR), molecules associated with T/NK-cell activation and the complement cascade, downstream targets of IFNγ and/or IFNγ/STAT1 signaling and upregulation of NF-κB pathways [116, 134]. The robust NF-κB target gene signature of HR-DLBCL partially overlaps with that of PMLBCLs, implicating the NF-κB survival pathway in this subtype [40, 54, 116]. HR-DLBCL lack most of the common cytogenetic abnormalities seen in OxPhos-DLBCL or BCR-DLBCL and occur in younger patients who often have splenomegaly and bone marrow involvement [116]. The molecular profiles and clinicopathologic features of HR-DLBCL tumors resemble those of T/HRLBCL [46, 116]. In contrast to OxPhos-DLBCL and BCR-DLBCLs, HR-DLBCL tumors are associated with a brisk, but ineffective host immune/inflammatory response and with a prominent T-cell/dendritic cell infiltrate as previously described for T/HRLBCL [46, 47, 116, 134]. 30.6 % of HR-DLBCL tumors analyzed in theses studies were classified as GCB- and 16.8 % as ABC-DLBCL. However the large majority (53 %) was sub-classified as type-3-DLBCL-NOS [116]. Remarkably, the clinical outcome of HR-DLBCLs upon R-CHOP treatment is not improved despite their increased host immune/inflammatory response [116, 134]. Thus, it has been suggested that similar to T/HRLBCL, either their immune responses are inhibited by counter-regulatory mechanisms or HR-DLBCL tumors were resistant towards R-CHOP-based chemotherapy, or a combination of both [116, 134].

The clinical outcome of tumor microenvironment/host inflammatory HR-DLBCL is quite similar to those of BCR/proliferation-DLBCL and OxPhos-DLBCL, with a 5-year survival of 54 to 60 % [40, 116, 134].

### MD-subtype DLBCLs

The c-MYC overexpressing subsets of DLBCL-NOS have been recently suggested to be sub-classified as c-MYC-driven (MD) subtype of GCB-, ABC and type-3-DLBCL-NOS [119]. C-MYC is overexpressed in up to 15 % of DLBCL-NOS and in up to 58 % of DLBCL, unclassifiable, with features intermediate between DLBCL and Burkitt lymphoma (DLBCL/BL) as a result of the t(8,14) translocation (5–14 %), gain/amplification (8q24) (21–38 %) or other, epigenetic mechanisms (28–41 %) [136, 137]. In accordance with the concept of *c–MYC* translocations arising in the GC microenvironment, most of the DLBCL-NOS cases harboring a *c-MYC* gene translocation show a GCB-type gene expression profile and/or a GCB phenotype [107, 138–147]. Large fractions of DLBCL (58–83 %) with the t(8,14) translocation contain concurrent rearrangements of the anti-apoptotic *BCL2* oncogene and are referred to as double-hit lymphomas [107, 137, 144]. Double-hit cases harboring a *c-MYC* gene translocation and *BCL6* rearrangements have also been reported, although at a much lower frequency than with *BCL2* [148]. In addition, in some cases with the t(8,14) translocation, there is a concurrent rearrangement of both anti-apoptotic *BCL2* and *BCL6* oncogene(s), which are referred to as triple-hit lymphomas [107, 144]. A recently published comprehensive study of double-hit and triple-hit lymphomas showed that 62 % of double hit lymphomas involve BCL2 and 18 % involved BCL6, the remaining cases were triple-hit lymphomas [149].

DLBCLs with high co-expression of c-MYC and BCL2 proteins have an aggressive clinical course and an inferior overall survival when treated with R-CHOP [143, 144, 150]. The aggressive nature of double-hit and triple-hit lymphomas is likely due to the concurrent rearrangement of both the pro-proliferative *c-MYC* oncogene and the anti-apoptotic *BCL2* oncogene [107, 144]. C-MYC/BCL2 co-overexpression in DLBCL is more common in the ABC subtype and contributes to the overall inferior prognosis of patients with ABC-DLBCL [107, 144]. Several studies indicate that the overexpression of the c-MYC protein might be a prognostic marker for poor survival in DLBCL, independent of BCL2 [138–142]. Thus, MD-DLBCLs, composed of the c-MYC-driven subsets of DLBCL-NOS and DLBCL/BL, including subsets of the newly defined categories of double-hit and triple-hit DLBCL have been suggested to represent an independent clinically highly relevant diagnostic molecular subtype [119]. However, it is still controversial whether the high expression of c-MYC has prognostic significance as a sole marker, independent of BCL2 co-overexpression [107, 144–147]. Indeed, it has been recently suggested that only high co-expression of both c-MYC and BCL2 proteins may serve as an independent predictor of very poor survival in DLBCL [107, 144–147]. The significantly worse outcome in patients with double-hit and triple-hit DLBCL may include both synergistic action of c-MYC and BCL2/BCL6 as well as other molecular features originating from the more numerous genetic aberrations in double-hit and triple-hit DLBCL [151, 152]. For instance, *TP53* inactivating mutations/deletions have been detected in a large number of high-risk subgroup of relapsed/refractory double-hit DLBCLs with *c-MYC* and *BCL2* [152–154] but not *c-MYC* and *BCL6* gene translocations [152]. *TP53* mutations constitute an early recurrent event in lymphomagenesis of c-MYC/BCL2 double-hit DLBCLs, leading to BCL2 driven evasion of apoptosis free from TP53-mediated control and contribute to the highly aggressive morphological and clinical phenotype of this entity [152–157].

### Stromal-II signature-subtype DLBCL

A large GEP study performed by Lenz G. et al. identified an additional microenvironment (stromal) gene expression signatures associated with superior or inferior outcomes, respectively after treatment with rituximab-based chemotherapeutic regimens [85]. Three gene-expression signatures, - termed germinal-center B cell, stromal-I and stromal-II signatures - were identified that predicted survival in patients who received CHOP or R-CHOP, respectively [85]. The stromal-I-signature, related to extracellular matrix deposition and histiocytic infiltration was associated with a good outcome [85, 158]. By contrast the angiogenesis-related stromal-II signature reflected tumor blood-vessel density and was found to be highly associated with a poor outcome [85]. This stromal-II signature includes genes encoding key regulators of angiogenesis such as vascular endothelial growth factor (VEGF) receptor 2, growth factor receptor-bound protein (GRB)-10, which mediates VGFR2 signaling; integrin alpha 9, which enhances VEGF signaling and the endothelial receptor tyrosine kinase TEK, the receptor kinase for angiopoietin signaling [85]. DLBCLs with overexpression of the stromal-II gene expression are associated with increased tumor blood-vessel density [85]. The stromal-II gene expression signature has been therefore suggested to represent an angiogenic switch in which the progression of a hyperplastic lesion to a fully malignant tumor is accompanied by new blood-vessel formation [85].

### *CDKN2A/2B* (9p21) deletion signature-subtype DLBCL

Jardin F. et al. recently defined an additional signature-subtype of DLBCL characterized by deletions of the cyclin-dependent kinase inhibitor genes *CDKN2A* and/or *CDKN2B* [120]. DLBCL with *CDKN2A/2B* (9p21) deletions have a specific gene expression profile and a poor prognosis under R-CHOP treatment [120]. The *CDKN2A/2B* locus encodes 2 different proteins: p16INK4A and p14ARF. Analysis of the 9p21 genomic region indicated that transcripts encoding p14ARF and p16INK4A were both disrupted in most patients with *CDKN2A/2B* deletion [120]. *CDKN2A/2B* deletion impairs both p14ARF/p53 and p16INK4A/pRB pathways. Loss of *CDKN2A/2B* is observed in up to 35 % of DLBCL-NOS patients and is significantly associated with a poor prognosis after R-CHOP treatment, independently of the international prognostic index (IPI) and COO [120].

*CDKN2A/2B* deletion is predominantly observed in ABC-DLBCL [13, 120]. *CDKN2A/2B* is deleted in up to 30 % of ABC-DLBCL and in up to 4 % of GCB-DLBCL [13, 120]. Patients with *CDKN2A/2B* deletion predominantly show an activated B cell profile and a specific gene expression signature that combines direct and indirect effects of the deletion, characterized by dysregulation of the RB/E2F pathway, activation of cellular metabolism, increase of anti-apoptotic mechanisms and decreased immune and inflammatory responses, including downregulation of FAS (TNFRSF6), IL1R1, AIM1, ARTD12 (PARP12) and TNFRSF1A [120].

### *RCOR1-*(*TRAF3*)*-*deletion signature-subtype DLBCL

A systematic integrative study of high-resolution genotyping arrays and RNA sequencing data of two independent large cohorts of homogenously R-CHOP-treated DLBCL patients identified novel focal and recurrent deletions in the chromatin regulator and transcriptional corepressor gene *RCOR1* (encoding CoREST1) that are associated with a novel prognostically significant risk-associated gene expression signature [121]. *RCOR1* deletions define a subgroup of DLBCL patients with unfavorable progression-free survival [121]. The chromatin regulator and transcriptional corepressor RCoR1/CoREST1 has been linked biochemically to hematopoiesis and is part of the BRAF35-histone deacetylase/LSD1-CoREST histone demethylase and chromatin remodeling complex, where it associates with the C-terminal domain of REST, the histone deacetylases 1 and 2 (HDAC1/2), and the histone demethylase LSD1/KDM1A [159]. The established *Rcor1* loss-associated prognostic gene signature was independent of the cell of origin classification [121]. This risk-associated gene expression signature comprises 233 genes and is enriched for biological processes that includes upregulation of the proteasome, processing of capped intron-containing pre-mRNA as well as downregulation of signaling events mediated by HDAC class II [121]. Interestingly, loss of *RCOR1* was associated with deletions of the *TRAF3* gene, which is located in close vicinity. TRAF3 is a negative regulator of the alternative non-canonical NF-κB signaling pathways in DLBCL, acting as a negative regulator of NF-κB-inducing kinase NIK [27, 160, 161]. Thus, it is very likely that the combination of transcriptional pattern changes mediated by *RCOR1* loss and the downstream effects on constitutive NF-κB signaling may cooperate and contribute to the malignant phenotype of this subgroup of DLBCL [121].

### Chemotherapeutical strategies and clinical outcome

Until 1997, the standard treatment for DLBCL was the anthracycline-based chemotherapy regimen of cyclophosphamide, hydroxyldaunorubicin, vincristine, and prednisone (CHOP). The majority of DLBCL patients initially respond favorably when treated with CHOP alone, but around 60 % eventually relapse [1, 109, 162]. Relapsed CHOP-resistant lymphomas disseminate and are highly lethal without autologous stem cell transplantation [1, 109, 162]. DLBCL subtypes differently respond to the standard CHOP chemotherapy. The ABC-DLBCL subtype is associated with a very poor prognosis when treated with CHOP only, the majority of ABC-DLBCL patients treated with CHOP alone will succumb to their disease [1, 109, 162]. In contrast, CHOP only treated patients with GCB-DLBCL and PMLBCL have a significantly better outcome with relatively favorable 5-year overall survival rates [1, 109, 162]. The constitutive activation of the NF-κB and BCR pathways has been suggested to be required for the anti-apoptotic phenotype and chemotherapy-resistance in ABC-DLBCL [35, 109, 163].

Since 1997 the co-administration of the chimeric anti-CD20 monoclonal antibody rituximab and CHOP chemotherapy significantly improved the survival of DLBCL patients, with a cure rate of 60 to 70 % [1, 109, 162]. The humanized chimeric anti-CD20 monoclonal antibody rituximab binds to the CD20 antigen on B-lymphoma cells and leads to the inhibition of five main pathways NF-κB, PI3K/AKT/mTORC1, STAT3, MEK/ERK and p38-MAPK pathways, resulting in downregulation of the expression of BCL2 family members and direct activation of FAS-mediated apoptosis [164–173]. This benefit is observed in both ABC- and GCB-DLBCL patients, though GCB-DLBCL is still associated with a much higher overall survival [40, 85, 163] and a relapse rate of only ∼15 % to 20 % [85, 174]. The 3-year progression free survival (PFS) rate and overall survival (OS) rate for R-CHOP treated patients with ABC-DLBCL are approximately 40 % and 45 %, respectively, while the corresponding PFS and OS rate for R-CHOP treated patients with GCB-DLBCL are approximately 74 % and 80 %, respectively [85, 163]. Approximately 15 to 30 % of relapsing GCB- and ABC-DLBCL patients have c-MYC/BCL2 or c-MYC/BCL6 double-hit DLBCLs with high co-expression of c-MYC and BCL2 or BCL6 proteins [85, 174]. These groups of patients pose a particular urgent clinical need because of a very aggressive clinical course, high chemorefractoriness and inferior overall survival when treated with R-CHOP [143, 144, 150].

The combination of rituximab with R-CHOP [175–177] or dose dense CHOP chemotherapy, every 14 or 21 days [178, 179], is now the current standard treatment for most patients with newly diagnosed DLBCL and improves the outcome of DLBCL patients of all ages and risk groups [1]. The complete remission (CR) rate of newly diagnosed DLBCL is approximately 65–75 % with R-CHOP [175]. An alternative standard regimen for first-line treatments is DA-EPOCH-R (dose-adjusted etoposide, prednisone, vincristine, cyclophosphamide, doxorubicin, rituximab) [180, 181]. Preliminary data from ongoing clinical studies suggest that DA-EPOCH-R therapy might have a superior outcome in some subtypes of DLBCL when compared to R-CHOP therapy [180, 182, 183]. For a detailed description of chemotherapeutic regimens used for the first-line treatment of DLBCL, the readers are referred to the recent excellent reviews [1, 109, 184–186]. Unfortunately, specific molecular subsets of DLBCLs at high risk for treatment failure with rituximab and/or anthracycline-based (immuno-)chemotherapy regimes are be coming more frequent [187]. Even though more than half of DLBLC patients achieve and maintain complete remission after first-line therapy with empiric combination multi-agent chemo-, radio- and/or immunotherapeutic regimes; 30 to 40 % of patients with specific molecular subsets of DLBCLs undergo relapse and approximately 10 to 15 % have refractory disease [188, 189]. Relapsed or refractory DLBCL is difficult to treat, with limited therapeutic options. Patients with relapsed/refractory DLBCL have a poor prognosis, with only ~10 % ultimately achieving complete cure [188].

After finding a significant improvement of survival outcomes in an international randomized phase III trial (PARMA study), high-dose chemotherapy (HDC) followed by autologous stem cell transplantation (ASCT) has been suggested as the standard therapy for patients with relapsed or refractory DLBCL [1, 190]. In addition, a recently performed prospective randomized trial by the Dutch Belgian Hemato-Oncology Cooperative Group established the benefits of rituximab combined with salvage chemotherapy, demonstrating clear survival benefits of combining rituximab with HDC prior to ASCT (HOVON-44 study) [191]. Rituximab-based HDC/ASCT therapy regimens have become the mainstay of therapy for patients with chemotherapy-sensitive relapsed DLBCL, still sensitive to conventional dose salvage chemotherapy [1, 192]. On the other hand, rituximab maintenance appears to have no defined role in relapsed DLBCL after ASCT [193].

Various salvage regimens are available such as R-DHAP (rituximab, dexamethasone, high-dose cytarabine, and cisplatin) R-DHAP-VIM-DHAP (rituximab-cisplatin, cytarabine, dexamethasone, etoposide - ifosfamide, methotrexate - cisplatin, cytarabine, dexamethasone), R-ESHAP (rituximab, etoposide, steroids, ara-C, and cisplatin), R-DHAX (rituximab, dexamethasone, cytarabine, and oxaliplatin), R-ICE (rituximab, ifosfamide, carboplatin, etoposide), DA-EPOCH-R (etoposide, doxorubicin, and cyclophosphamide with vincristine, prednisone, and rituximab), R-GIFOX (rituximab, gemcitabine, ifosfamide, oxaliplatin), R-GEMOX (rituximab emcitabine, oxaliplatin), R-GDP (rituximab plus gemcitabine, cisplatin, and dexamethasone), R-MINE (rituximab mesna, ifosfamide, mitoxantrone, etoposide) or R-BEAM (rituximab plus carmustine, etoposide, cytarabine, and melphala) [191, 193–206]. However, salvage therapy and transplant conditioning regimens are still suboptimal, as are therapeutic options for patients who relapse following ASCT [1, 192]. Thus, the optimal salvage chemotherapy regimen still needs to be determined. A summary of randomized, phase III trial studies of rituximab combined chemotherapy regimens in relapsed/refractory DLBCL is presented in Table 6. R-ICE or R-DHAP followed by high-dose therapy and autologous stem cell transplantation are the two most widely used regimens worldwide for the treatment of patients with relapsed/refractory DLBCL [1, 192, 193]. Recently, the results of the randomized collaborative trial, which compared R-DHAP to R-ICE followed by HDC/ASCT in relapsed aggressive B-cell lymphoma, including relapsed/refractory DLBCL, have been reported (CORAL study) [193]. There were no significant differences reported between R-ICE and R-DHAP for ORR, 3-year EFS, or OS [193]. However, a subsequent subgroup analysis performed on the CORAL database showed that salvage treatment with R-DHAP was superior to R-ICE in the GCB subtype and may improve outcome in patients with GCB-type DLBCL [174, 207].

**Table 6:** Randomized, phase III trial studies of rituximab combined chemotherapy regimens in relapsed/refractory DLBCL

Design/regimen	Patient characteristics	Results	Status	Study
R- DHAP-VIM-DHAP (A) vs. DHAP-VIM-DHAP (B) Prior to BEAM/ASCT	Patient No.: 239 Age: 18 to 65 y;	Survival benefits of combining rituximab with HDC/ASCT: 2-year FFS: 50 (A) vs 24 % (B) 2-year PFS: 52 (A) vs 31 % (B)	Completed phase III	[191] Trial-No: NCT00012051 (HOVON-44 study)
R-ICE (A) vs. R-DHAP (B) After HDC/ASCT	Patient No.: 477 Age: 18 to 65 y;	No survival differences between R-ICE and R-DHAP Rituximab maintenance has no defined role in relapsed DLBCL after ASCT	Completed phase III	[193, 624] Trial-No: NCT00137995 (CORAL study)
R-GDP vs. R-DHAP As salvage chemotherapy prior to ASCT	Patient No.: 619 Age: 16 to 65 y	No survival differences between R-GDP and R-DHAP GDP is associated with less toxicity and hospitalization, and superior quality of life.	Completed phase III	[195] Trial-No: NCT00078949
R-BEAM vs. Bexxar/BEAM prior to ASCT	Patient No.: 224 Age: 18 to 80 y	B-BEAM and R-BEAM regimens produced similar 2-year PFS and OS rates	Completed phase III	[194] Trial-No: NCT00329030
Ofatumumab + DHAP vs. R-DHAP As salvage chemoimmunotherapy followed by ASCT	Patient No.: 445 Age: 18 to 65	NA	Completed phase III	Trial-No:NCT01014208 (ORCHARRD study)
R-DHAP +/− Bortezomib As induction therapy before HDC/BEAM/ASCT	Patient No.: 108 (estimated) Age: 18 to 65 (estimated)	NA	Ongoing Phase III	Trial-No: NCT01805557 (FILVERAL12 study)
90-Yttrium Ibritumomab Tiuxetan (Zevalin) +/− R-BEAM	Patient No.: 158 (estimated) Age: 18 to 70 (estimated)	NA	Ongoing Phase III	Trial-No: NCT02366663 (SPINOZA study)

*DLBCL* diffuse large B cell lymphoma, *BEAM* carmustine, etoposide, cytarabine, and melphala, *BEAM/ASCT* carmustine, etoposide, cytarabine, and melphala with autologous stem cell transplantation, *PFS* progression-free survival, *FFS* failure-free survival, *OS* overall survival, OS overall survival, *HDC* high-dose chemotherapy, *HDC/ASCT* high-dose chemotherapy and autologous stem cell transplantation, *R-DHAP* rituximab plus dexamethasone, cytarabine, and cisplatinum, *R-GDP* rituximab plus gemcitabine, cisplatin, and dexamethasone, *DHAP-VIM-DHAP* cisplatin-cytarabine-dexamethasone -- etoposide-ifosfamide-methotrexate -- cisplatin-cytarabine-dexamethasone, *R-ICE* ifosfamide, carboplatin and etoposide, *Bexxar* iodine-131 tositumomab

Unfortunately, not all patients are fit or eligible for the HDC-ASCT. Patients with aggressive non-GCB-DLBCL (ABC-subtype or c-MYC-driven type-3-DLBCL-NOS) respond poorly to treatment with classical R-ICE or R-DHAP based salvage therapy [207–209]. Relapsed DLBCLs resistant to rituximab alone or R-CHOP treatments are refractory to subsequent treatments with the initial chemotherapy regimen and may even exhibit cross-resistance to multiple chemotherapeutic anticancer drugs [169, 210]. Many patients ultimately succumb to these aggressive tumors despite second-line chemotherapy and autologous stem cell transplantation, resulting in disappointing 3-year overall survival rates of approximately 30 % [4, 188, 207]. Patients with relapsed/refractory DLBCL who relapse after HDC-ASCT and develop refractory disease have an extremely poor prognosis with only a few, if any, curative therapy options and a median survival of approximately 3 to 4 months [188, 207]. The management of patients ineligible for HDC-ASCT or with relapsed/refractory DLBCL relapsing after HDC-ASCT is very difficult and the only remaining treatment option for these patients includes participation in phase 1/2 clinical trials with novel experimental agents or palliative therapy [188, 207]. Thus, for the remainder of these patients with relapsed/refractory and high-risk biological subtypes of DLBCL, further improvements in therapy and novel therapeutic strategies are urgently needed. For a detailed description of drug resistance pathways in DLBCL and chemotherapeutic regimens used for the treatment of relapsed/refractory DLBCL, the readers are referred to the recent excellent reviews [187–189, 192, 211, 212].

### Selected novel drug targets under study for relapsed/chemo-refractory DLBCLS

Most patients with relapsed or refractory DLBCLs resistant to rituximab and/or R-CHOP chemotherapy are thought to have ABC-DLBCL or type 3-DLBCLs [35]. Three different types of drug resistance have been defined and are associated with adverse clinical outcomes: Drug resistance of DLBCLs can be inherent from the beginning (innate, due to the genetic heterogeneity of DLBCL tumor cells) [213, 214]. This type is called intrinsic genetic resistance, which is associated with recurrent translocations and the presence of specific genetic abnormalities [12, 18, 213, 214]. Inherited genetic variations can contribute to the risk of therapy-induced side effects [12, 18, 215]. A second type, termed treatment acquired resistance, develops from prior exposure to chemotherapy [33, 213, 214]. Acquisition of chemotherapy resistance in DLBCL is due to the genetic and epigenetic instability of DLBCL tumor cells and emergence of subclonal populations of drug-resistant tumor clones, which eventually leads to the failure of standard rituximab and/or anthracycline-based chemotherapy regimens [33, 213, 214]. A third resistance mechanism, known as tumor microenvironment (TME)/cell adhesion–mediated drug resistance, arises from the interaction of DLBCL tumor cells with the normal stromal tissue [216–219].

Due to the extensive heterogeneous genetic nature of DLBCL, multiple drug-resistant molecular mechanisms are required for the intrinsic genetic resistance and acquisition of chemotherapy resistance in DLBCL. Only a small number of high-risk subsets of DLBCL are associated with increased expression of multidrug pumps (i.e., P-glycoprotein; MDR1/ABCB1) [189, 220, 221] and overexpression does not appear to be consistently associated with chemoresistance in DLBCL [221, 222]. Moreover, CD20 mutations involving the rituximab epitope are rare in both *de novo* and relapsed/refractory DLBCL tumors, and do not represent a significant cause of R-CHOP resistance [223]. CD20 protein-negative relapses can occur in up to 20 % DLBCL cases after rituximab-containing combination chemotherapies [223–226]. Downregulation of CD20 protein expression strongly correlates with rituximab resistance *in vitro* [227]. However its clinical relevance is not yet fully understood [223–226]. Chemotherapy resistance has been mainly associated with down-regulation of intrinsic apoptosis pathways and activation of survival pathways in DLBCL [165, 166, 169–171, 189, 228]. For instance, repeated exposure to rituximab can generate a therapy-resistant phenotype by the upregulation of the anti-apoptotic BCL2 family proteins [169] or downregulation of the pro-apoptotic BAK and BAX proteins [229]. A summary of observed and postulated (immuno)-chemotherapy resistance mechanisms in DLBCL is presented in Table 7.

**Table 7:** Summary of observed mechanisms underlying drug resistance in DLBCL

Resistance mechanisms	Resistance type	Examples of molecules involved in	Examples of drugs affected	Study
Upregulation or constitutive activation of pro-survival pathways	Intrinsic genetic resistance Treatment acquired resistance	BCL2 family/c-MYC (↑)	Proteasome inhibitors	[209, 625]
		BCR signaling (↑), NF-κB (↑) JAK/STAT3 signaling (↑)	(Bortezomib), CHOP, Rituximab	[107, 138, 164, 169, 171, 218, 366, 398, 407, 626]
		PAK1 (MEK/ERK, PI3K/AKT, WNT/β-catenin signaling) (↑)	PI3K, mTORC1/2 inhibitors	
Downregulation or inactivation of pro- apoptotic pathways	Intrinsic genetic resistance Treatment acquired resistance	TP53 (↓/Mutated)	Cisplatin, cytarabine, doxorubicin, etoposide, (−> CHOP, ICE, DHAP etc.)	[627–629]
Metabolic detoxification of drugs	Intrinsic genetic resistance Treatment acquired resistance	ALDH1A1, GPX1	Doxorubicin (−> CHOP)	[630]
Upregulation or constitutive activation of DNA damage signaling pathways	Intrinsic genetic resistance Treatment acquired resistance	CHK1/2 (↑) DTX3L, ARTD9 (↑)	Doxorubicin (−> CHOP)	[470, 547, 548, 631, 632]
Upregulation of multi-drug export machines	Intrinsic genetic resistance Treatment acquired resistance	ABCB1, ABCG2 (↑)	Doxorubicin (−>CHOP), methotrexate, fludarabine, adriamycin, vinblastine	[220, 630, 633]
Upregulation or (constitutive) activation of alternative pro-survival pathways	Intrinsic genetic resistance Treatment acquired resistance	MCL1, BFL1 (↑)	BCL2 inhibitors (ABT-737)	[363, 364]
		HSP27, HSP70, HSP90 (↑) MCL1, BCL-XL (↑) HDAC3 (↑)	Proteasome inhibitors (Bortezomib) Rituximab	[166, 268, 371, 560, 561]
		CARD11 mutant or MyD88 mutant mediated signaling -> NF-κB (↑)	BTK inhibitors (Ibrutinib)	[104, 259, 634]
Loss of key negative regulators of pro-survival signaling pathways	Intrinsic genetic resistance Treatment acquired resistance	4EBP1 (↓) - > mTORC1 pathway (↑)	mTORC1 inhibitors	[635]
Mutation(s) in target molecules or target pathways	Treatment acquired resistance	Ibrutinib binding site mutations in BTK. Gain-of-function mutation in PLCγ2 -> BCR signaling (↑)	BTK inhibitors (Ibrutinib)	[465]
Downregulation of target molecules		CD20 (↓)	Rituximab	[223–227]
TME induced up regulation of pro-survival signaling pathways	Treatment acquired resistance TME mediated resistance	BCR signaling (↑) ABCG2 (↑) BCL2, BCL-XL, BCL2A1 (↑)	CHOP	[217, 218] [633]

*CHOP* cyclophosphamide, doxorubicin, vincristine, and prednisone, *DHAP* cisplatin, cytarabine, dexamethasone, etoposide, *VIM* ifosfamide, methotrexate, *ESHAP* etoposide, steroids, ara-C, and cisplatin, *ICE* ifosfamide, carboplatin, etoposide, *GDP* gemcitabine, cisplatin, and dexamethasone, *BEAM* carmustine, etoposide, cytarabine, melphala, *MDR* multi drug resistance, *BCR* B cell receptor, *NF-κB* nuclear factor-kappa B, *JAK* Janus kinase, *STAT* signal transducer and activator of transcription, *PAK1* p21-activated kinase 1, *CHK1/2* Chk1 protein kinase 1/2, *DTX3L* deltex (DTX)-3-like E3 ubiquitin ligase, *ARTD9* ADP-ribosyltransferase-9 diphteria toxin like, *BCL* B-cell lymphoma protein, *MCL1* myeloid cell leukemia 1, *HSPs* heat shock proteins, *HDAC* histone deacetylase, *CARD11* caspase recruitment domain family, member 11, *MyD88* myeloid differentiation primary response 88, *4EBP1* eukaryotic translation initiation factor 4E binding protein 1, *mTORC1*, mammalian target of rapamycin (mTOR) complex 1, *ALDH1A1* aldehyde dehydrogenase 1A1, *GPX1* glutathione peroxidase 1, *ABCG2* ATP-binding cassette, sub-family G, member 2, *ABCB1* ATP-binding cassette, sub-family B (MDR/TAP), member 1*, PLCγ2* phospholipase C gamma 2, *BTK* Bruton’s tyrosine kinase, *TME* tumor microenvironment

Multiple driver mutations and aberrant signaling pathways suggested to be required for drug resistance in DLBCL have been recently identified in specific molecular subsets of DLBCLs through gene expression profiling (GEP), transcriptome sequencing, RNA interference screens, and DNA sequencing and have increased our understanding of (chemotherapy-) resistance. A detailed list of novel potential drug targets and agents in DLBCL-NOS, including relapsed/refractory DLBCL is shown in Additional file 1: Table S1. Numerous small molecule inhibitors acting as GCB- and ABC-DLBCL subtype-specific pathway inhibitors are now in various stages of investigation, including clinical trial phase III studies. Only a few of them have been already approved for the treatment of B-cell malignancies, none of them for the treatment of DLBCL (Table 8). At least seven are currently evaluated in clinical phase III studies in patients with newly diagnosed or relapsed/refractory DLBCL (Table 9). These drugs are mainly targeting oncogenic factors regulating cell metabolism, proliferation, cell cycle, growth, migration, survival and angiogenesis in a subtype-specific manner. A detailed list of novel single experimental agents in clinical trials in relapsed/refractory DLBCL-NOS is shown in Additional file 1: Table S2. Completed and ongoing experimental clinical studies combining novel experimental agents with conventional (immuno-)chemotherapy (i.e., R-CHOP, DHAP) in first-line treatment of newly diagnosed DLBCL and/or second-line treatment of relapsed/refractory DLBCL are summarized in Additional file 1: Tables S3–S5.

**Table 8:** Approved novel drugs for treatment of B-lymphoid malignancies that are currently in clinical evaluation for the treatment of DLBCL

Drug name	Target/Pathway	Manufacturer	Approved for the treatment of
Ofatumumab (Arzerra) Second-generation anti-CD20 mAb	(↓) CD20-pro-survival signaling	GSK Genmab	B-cell chronic lymphocytic leukemia
Obinutuzumab (Gazyva/GA101) Third-generation anti-CD20 mAb	(↓) CD20-pro-survival signaling	Roche Pharmaceuticals	B-cell chronic lymphocytic leukemia
Ibrutinib (Imbruvica/PCI-32765) BTK inhibitor	(↓) BTK, BCR/NF-κB-pro-survival signaling	Pharmacyclics/Janssen Pharmaceutica	Mantle cell lymphoma B-cell chronic lymphocytic leukemia
Lenalidomide (Revlimid) Immunomodulatory drug	(↓) IRF4, BCR/NF-κB-pro-survival signaling (↑) pro-apoptotic STAT2- IFNα/β axis	Celgene	Multiple myeloma Mantle cell lymphoma
Bortezomib (Velcade) Proteasome Inhibitor	(↓) NF-κB-pro-survival signaling	Millennium Pharmaceuticals	Multiple myeloma
Carfilzomib (Kyprolis) Proteasome Inhibitor	(↓) NF-κB-pro-survival signaling		Multiple myeloma
Idelalisib (Zydelig/CAL-101, GS 1101) PI3Kδ inhibitor	(↓) PI3Kδ PI3K/AKT/mTORC1 pathway	Gilead Sciences Inc.	B-cell chronic lymphocytic leukemia Small lymphocytic lymphoma Follicular lymphoma
Dasatinib SRC kinase inhibitor	(↓) SRC family BCR/NF-κB-pro-survival signaling	Bristol-Myers Squibb	Chronic myeloid leukemia
Brentuximab vedotin (Adcetris/SGN-35)	(↓) Tubulin (↑) pro-apoptotic signaling	Seattle Genetics Inc.	Hodgkin’s lymphoma B and T-acute lymphoblastic leukemia
Antibody-drug conjugate (ADC) (anti-CD30 mAb conjugated to cytotoxin MMAE)	(MMAE is a microtubule disrupting agent)		Anaplastic lymphoma kinase-positive large B-cell lymphoma
Panobinostat (LBH589) HDAC inhibitor	(↓) HDAC, c-MYC pro-survival signaling	Novartis Pharmaceuticals	Multiple myeloma
Alemtuzumab (Campath-1H/LDP-03) Anti-CD52 mAB	(↓) CD52-pro-survival signaling	Sanofi	B-cell chronic lymphocytic leukemia

*mAB* monoclonal antibody, *BTK* Bruton’s tyrosine kinase, *BCR* B cell receptor, *NF-κB* nuclear factor-kappa B, *IRF4* interferon-regulatory factor 4, *STAT2* signal transducer and activator of transcription 2, *IFNα/β* interferon alpha/beta, *PI3K* phosphoinositide 3-kinase, *mTORC1*, mammalian target of rapamycin (mTOR) complex 1, *HDAC* histone deacetylase

**Table 9:** Novel agents in clinical trial phase III studies for first-line and second-line treatment of DLBCL-NOS

Drug	Agent mechanism or target	Pathway	Manufacturer	Status	Studies
Enzastaurin (LY317615)	PKCβ inhibitor	(↓) BCR/ NF-κB pro-survival signaling	Denovo Biopharma LLC	Completed phase III	[258] Trial-No: NCT00332202 (PRELUDE)
Ofatumumab (Arzerra)	CD20 Second- generation anti-CD20 mAb	(↓) CD20 pro-survival signaling	GSK Genmab	Completed phase III	Trial-No:NCT01014208 (ORCHARRD)
Bortezomib (Velcade)	Proteasome Inhibitor	(↓) NF-κB pro-survival signaling	Millennium Pharmaceuticals	Ongoing phase III	Trial-No: NCT01805557 (FILVERAL12) NCT01324596 (REMoDL-B)
Lenalidomide (Revlimid)	IRF4 Immuno modulatory drug	(↓) BCR/ NF-κB pro-survival signaling (↑) pro-apoptotic STAT2-IFNα/β axis	Celgene	Ongoing phase III	[294] Trial-No: NCT01122472 (REMARC) NCT02285062 (ROBUST) NCT02128061 NCT01197560
Ibrutinib (PCI-32765)	BTK inhibitor	(↓) BCR/ NF-κB pro-survival signaling	Pharmacyclics Janssen Pharmaceutica	Ongoing phase III	Trial-No: NCT01804686 NCT01855750
Everolimus (RAD001/Afinitor)	mTORC1 inhibitor	(↓) PIK/AKT/ mTORC1 pro-survival signaling	Novartis Pharmaceuticals	Ongoing phase III	Trial-No: NCT00790036
Obinutuzumab (Gazyva, Gazyvaro GA101/RO5072759)	CD20 Third- generation anti-CD20 mAb	(↓) CD20 pro-survival signaling	Roche Pharmaceuticals	Ongoing phase III	Trial-No: NCT01287741 (GOYA)

*mAB* monoclonal antibody, *PKCβ* protein kinase Cβ, *BCR* B cell receptor, *NF-κB*, nuclear factor-kappa B, *IRF4* interferon-regulatory factor 4, *STAT2* signal transducer and activator, of transcription 2, *IFNα/β* interferon alpha/beta, *BTK* Bruton’s tyrosine kinase, *mTORC1*, mammalian target of rapamycin (mTOR) complex 1, *PI3K* phosphoinositide 3-kinase

Most of these drugs are targeting the DLBCL subtypes according to their COO status (GCB- or ABC-specific) and CC status (OxPhos-, BCR- or MD-specific). The most important of these novel drug targets currently under study are discussed below.

### Inhibition of BTK and SYK kinases in the BCR-subtype of ABC-DLBCL

As mentioned above chronic active BCR-mediated signaling was recently identified as a critically important pathway in the pathogenesis of ABC-DLBCL [96, 116, 122, 230]. Chronic active BCR signaling in DLBCL is mainly dependent on the BTK, SYK and PI3K kinases [231]. The BTK, SYK and PI3K kinases have significant activities in patients with relapsed/refractory ABC-DLBCL [96, 116, 230]. Thus, the BCR-associated kinases SYK and BTK have emerged as promising therapeutic targets for relapsed/refractory ABC-DLBCL. SiRNA-mediated depletion of SYK or BTK as well as inhibition of SYK or BTK by small molecule inhibitors selectively decreased BCR signaling and induced apoptosis of BCR-dependent DLBCL cell lines [90, 98, 232–234]. In a phase I/II clinical trial study that involved 80 subjects with relapsed or refractory DLBCL, ibrutinib (PCI-32765), a BTK inhibitor, have shown a promising activity of ibrutinib as a single agent in the BCR- subtype of ABC-DLBCL; a 37 % ORR and a 16 % CR in relapsed/refractory DLBCL was reported for patients with the ABC- subtype compared with only 5 % of those with the germinal GCB subtype [235–238]. Thus, ibrutinib efficacy is limited to ABC-DLBCL patients with a constitutively active BCR signaling pathway [235, 239, 240]. Consistent with the cooperation between the BCR and MyD88 pathways observed *in vitro*, ABC tumors with concomitant *BCR* and *MyD88* mutations responded to ibrutinib frequently [235]. However, the highest number of responses occurred in ABC tumors that lacked BCR mutations, as 67 % of ibrutinib responders had wild-type CD79A and CD79B [235]. Remarkably, ibrutinib does not inhibit the growth and survival of BCR wild-type tumors with *MyD88-* and/or *CARD11* mutations [235, 239, 240] indicating that ibrutinib specifically targets ABC-DLBCL tumors that rely on chronic active BCR signaling [235].

Ibrutinib is a selective and irreversible BTK inhibitor that binds covalently to a C481 residue in the BTK active site, preventing Y223 phosphorylation required for activation [241]. Ibrutinib is very well tolerable both as single agent and in combination with R-CHOP [235–237, 242]. Ongoing clinical phase II studies and several randomized clinical phase III studies are evaluating ibrutinib with or without R-CHOP in patients with newly diagnosed and relapsed/refractory DLBCL 30 (NCT01325701, NCT01197560, NCT01804686, NCT01855750) [235, 236, 243–245] (Table 9 and Additional file 1: Tables S2–S5). Fostamatinib (R406, FosD), a selective oral small molecule inhibitor of SYK, also showed significant activity in relapsed/refractory DLBCL in a phase I/II study [246] (Additional file 1: Table S2). More than 20 % of the patients with multiple relapsed/refractory DLBCL responded to therapy with fostamatinib, though the CR was only 5 % [246]. A multicenter phase II trial of fostamatinib has completed and is pending announcement of the results (NCT01499303) (Additional file 1: Table S2). Additional phase II trials have been proposed to determine whether fostamatinib may improve the response to rituximab [246].

### Inhibition of PKCβ in the BCR-subtype of ABC-DLBCL

Another potential therapeutic target in relapsed/refractory BCR-subtypes of ABC-DLBCL is the protein kinase C (PKC) isoform PKCβ II. PKCβ II is a serine/threonine kinase isoform amplified through the BCR signaling pathway and is highly expressed in refractory DLBCL [247–250]. PKCβ-II overexpression is an adverse prognostic factor in DLBCL and associated with poor prognosis in BCR-subtypes of ABC-DLBCL and GCB-DLBCL deficient of phosphatase and tensin homolog (PTEN) [247–251]. Preclinical studies demonstrated that sotrastaurin (AEB071) and enzastaurin, two adenosine triphosphate-competitive selective inhibitors of PKCβ, induce apoptosis and inhibit the proliferation of BCR-subtypes of ABC-DLBCL *in vitro* and *in vivo* [252, 253].

Sotrastaurin selectively inhibited the growth of CD79 mutant BCR-subtypes of ABC-DLBCL *in vitro* and *in vivo* whereas the presence of CARD11 mutations resulted in resistance to the inhibitor [253]. Sotrastaurin is currently being tested in an international multi-institutional phase I/II trial study in patients with DLBCL that harbor either CD79A or CD79B mutations (NCT01854606) [109]. Treatment with enzastaurin is well tolerated in patients with newly diagnosed DLBCL as well as in patients with relapsed/refractory DLBCL [254–257]. However a recent clinical phase I/II trial study showed that the improved outcome upon treatment using enzastaurin alone was only modest in relapsed/refractory DLBCL (NCT00042666, Additional file 1: Table S2), indicating that enzastaurin is not very effective as single-agent [254–256]. Moreover, a randomized phase III study of enzastaurin as single-agent in patients with newly diagnosed DLBCL did not meet its primary endpoint to improve progression-free survival (NCT00332202) [258] (Table 9). Two clinical phase II studies evaluating the efficacy and safety of enzastaurin in combination with R-CHOP or R-Gemox have been recently completed and are pending announcement of the results (NCT00436280, NCT00451178) (Additional file 1: Table S3).

### Inhibition of canonical NF-κB signaling pathways in ABC-DLBCL

As already discussed in previous sections recent studies validated the NF-κB signaling pathways as an important therapeutic target in ABC-DLBCL. Receptor-mediated constitutive activation of NF-κB in B-cell malignancies can occur through two distinct signaling pathways: the canonical pathway or classical pathway mediated by the action of RelA/p50 heterodimers and the non-canonical or alternative pathway mediated by the action of p52/p50, RelB/p50 or RelB/p52 heterodimers [26, 27, 160, 161]. The canonical pathway is the major NF-κB signaling pathway in DLBCLs and activated through BCR, CD40 and/or TLR signaling and by the inhibitor of κB kinase complex (IKKα/β/γ), which in turn, phosphorylates IκB and causes its degradation [27, 160, 161].

The major fraction of oncogenic NF-κB activating mutations in DLBCL is predominantly related to the canonical NF-κB pathway [12, 16, 18, 19, 96, 104, 259]. The less well-studied non-canonical NF-κB pathway is not yet fully established as a drug target in DLBCL, see next section. ABC-DLBCL and PMLBCL cell lines and primary tumors, including drug-resistant cases, can be sensitized *in vitro* and *in vivo* to chemotherapy by treatment with drugs, which can inhibit the canonical NF-κB pathway. For instance, small molecule inhibitors of the IKK complex were found to be selectively toxic for ABC-DLBCL and PMLBCL cell lines, but had no effect on GCB-DLBCL cell lines [59]. Proteasome inhibitors such as bortezomib or carfilzomib, block the degradation of negative regulators of cell cycle progression as well as of NF-κB inhibitory protein IκBα thereby inducing cell cycle arrest and mitochondrial dependent apoptosis in ABC-DLBCL [260–262]. A preclinical/clinical phase II study demonstrated that targeting the canonical NF-κB pathway through inhibition of the 26S proteasome complex with bortezomib can selectively sensitize patients with relapsed/refractory ABC-DLBCL, but not patients with relapsed/refractory GCB-DLBCL, to chemotherapy (including (R-)CHOP and DA-EPOCH) [263]. Bortezomib alone as single agent had no significant activity in relapsed/refractory DLBCL [263–265], but when combined with chemotherapy, it showed a significantly higher response (83 % vs. 13 %) and median overall survival (10.8 vs. 3.4 months) in ABC compared with GCB-DLBCL, respectively [263] (Additional file 1: Table S5). A subsequent phase I/II study of patients with previously untreated DLBCL investigating bortezomib in combination with R-CHOP demonstrated that the efficacy of bortezomib plus R-CHOP was similar in patients with non-GCB- and GCB-DLBCL, consistent with the concept that the unfavorable non-GCB-DLBCL subgroup with constitutive NF-κB overexpression derives benefit from bortezomib sensitization with chemotherapy [266] (Additional file 1: Table S3). A phase II study and a randomized phase III study evaluating the use of R-CHOP with or without bortezomib in unselected patients with newly diagnosed DLBCL (Additional file 1: Table S4) as well as a randomized phase II/III study of R-DHAP with and without bortezomib in patients with relapsed/refractory DLBCL are currently ongoing (NCT00931918, NCT01324596 and NCT01805557) (Table 9). Unfortunately, not all ABC-DLBCL are bortezomib-sensitive, and patients may eventually develop bortezomib-resistant disease. Preclinical studies showed that proteasome inhibitors not only trigger the accumulation of proapoptotic proteins, but can also up-regulate antiapoptotic proteins, particularly MCL1 [267] and HSP90 [268], which are implicated in bortezomib resistance [267–269].

Surprisingly, a recent preclinical study uncovered an unexpected profound regulatory role for the bromodomain and extraterminal domain (BET) proteins BRD2 and BRD4 in cytoplasmic signaling through IKK in ABC-DLBCL [239]. Inhibition BET proteins by small molecules inhibitors CPI203 and JQ1 as well as siRNA-mediated depletion of BRD2 and BRD4 expression, attenuated oncogenic IKKβ signaling, thereby inhibiting downstream oncogenic NF-κB-driven transcriptional programs and killing ABC-DLBCL cells *in vitro* and in an ABC-DLBCL xenograft model [239].

### Inhibition of MALT1 in the BCR-subtype of ABC-DLBCL

Recent studies demonstrated that the proteolytic activity of mucosa-associated lymphoid tissue lymphoma translocation (MALT) protein-1 is required for the survival and pathogenesis of ABC- DLBCL with chronic active BCR signaling [270, 271] and therefore, represents another new potential therapeutic target in relapsed/refractory cases of the BCR-subtype of ABC-DLBCL [270]. MALT1 is an enzymatically active signaling component essential for upstream activation of NF-κB upon antigen stimulation of BCR [270]. MALT1-dependent cleavage of the non-canonical and tumor suppressive NF-κB family member RELB promotes canonical NF-κB activation in DLBCL [272]. Recent preclinical studies demonstrated that selective inhibition of the proteolytic activity of MALT1 with small-molecule inhibitors blocks the anti-apoptotic NF-κB signaling pathway and elicits toxic effects selectively on MALT1-dependent subsets of ABC-DLBCL cells *in vitro* and *in vivo* with very little toxicity towards primary B cells [273, 274].

### Inhibition of TLR-mediated canonical NF-κB signaling pathways in ABC-DLBCL

As mentioned above, a recent clinical phase II study demonstrated that ibrutinib does not inhibit the growth and survival of BCR wild-type ABC-DLBCL tumors with *MyD88* mutations [235, 239, 240]. MyD88 is an initial adapter linker protein in the canonical NF-κB signaling pathway activated by Toll-like receptors (TLRs), including the endosomal TLRs 7, 8, and 9 [275]. In the presence of the most common MyD88 mutant L265P, ligand activation of those TLRs results in markedly increased signaling with subsequent increased cell activation, cell survival, and cell proliferation in DLBCL [103]. Thus, direct inhibition of TLR/MyD88 signaling pathways by TLR antagonists might circumvent the resistance of BCR wild-type ABC-DLBCL tumors with *MyD88* mutations against ibrutinib. A dose escalation phase I/II study evaluating the efficacy and safety of the TLR antagonist IMO-8400 in patients with relapsed or refractory DLBCL bearing a MyD88-L265P mutation has been recently started (NCT02252146). IMO-8400 is an antagonistic oligonucleotide specifically designed to inhibit ligand activation of TLRs 7, 8 and 9 [276]. The scientific rationale for assessing the use of IMO-8400 to treat patients with DLBCL and the L265P mutation is based on the observations that IMO-8400 inhibits ligand-based activation of DLBCL cell lines with the L265P mutation and decreases the survival and proliferation of DLBCL cells (unpublished data of preclinical studies performed by Idera Pharmaceuticals, Inc.).

### Inhibition of non-canonical NF-κB signaling pathways in *TRAF3*-negative ABC-DLBCL

Several recent studies provided strong evidence for an important role of the non-canonical NF-κB signaling pathway in DLBCL, particularly in ABC-DLBCL [160]. Non-canonical NF-κB signaling appears to be activated by a restricted number of ligands in DLBCL, such as CD30 ligand (CD30L), CD40 ligand (CD40L) and B cell activating factor BAFF, belonging to the TNF superfamily. Binding to the corresponding receptors (CD30, CD40 or BAFF-R/BR3) results in activation and accumulation NF-κB-inducing kinase (NIK), which in turn activates IKKα, the kinase that promotes the processing of p100 into the active p52 isoform, thereby resulting in continuous activation of p52/p50, RelB/p50 or RelB/p52 heterodimers in the nucleus [27, 160, 161]. Pham L.V. et al. recently reported that NIK kinase is overexpressed and accumulates in both GCB-like and ABC-like DLBCL cell lines [160]. NIK activation is usually tightly controlled through negative feedback mechanisms involving negative regulation by the adaptor/regulator proteins TNF receptor-associated factor 2 and 3 (TRAF2/3) and the cellular inhibitors of apoptosis ubiquitin ligases (c-IAP1/2) [27, 160, 161]. CD30, CD40 and BR3 receptors have been suggested to form a multimeric complex with TRAF3, TRAF2, TRAF5 c-IAP1, and c-IAP2 in DLBCL cells [27, 160, 161]. Both TRAF2 and TRAF3 serve as negative regulators of non-canonical NF-κB signaling pathways and target NIK for constant ubiquitination and degradation [161]. Loss of this quaternary inhibitory complex can lead to increased NIK protein accumulation and constitutive activation of the non-canonical NF-κB signaling pathway [161]. Indeed, 10 % of DLBCL cases demonstrate nuclear NF-κB activity exclusively for the non-canonical pathway (indicated by nuclear staining of p52 but not p50) while 20 % of DLBCLs display nuclear staining for both p50 and p52 [18], strongly indicating that the concurrent activation of both canonical and non-canonical NF-κB pathways occurs in a large fraction of DLBCL [18, 27, 160]. In line with these observation, recurrent loss of function mutations and deletions were recently revealed in genes encoding TRAF2 and/or TRAF3 in DLBCL [12, 15, 16, 19, 26, 27, 161]. Zhang B. et al. demonstrated that biallelic or monoallelic deletions/mutations of TRAF3 occur recurrently in roughly 15 % of ABC-DLBCL and GCB-DLBCL (in similar fractions) and correlate with non-canonical NF-κB activity in ABC-DLBCL cases [27]. Modeling these genetic events in mice, Zhang B. et al. demonstrated a key oncogenic role for the non-canonical NF-κB pathways in DLBCL pathogenesis [27]. Most DLBCL tumors developed in their mice model resembled ABC-DLBCL [27]. Thus, NIK appears to be an attractive new therapeutic target for ABC-DLBCL treatment, particularly for patients with ABC-DLBCL that are refractory to bortezomib or to the BCR pathway inhibitor ibrutinib. Of interest, proteasome inhibitors such as bortezomib or carfilzomib, can also block the constant ubiquitination and degradation of NIK, thereby upregulating the non-canonical NF-κB signaling pathways. In addition, targeting both arms of NF-κB signaling may also improve the therapeutic outcome in patients with newly diagnosed high-risk DLBCL displaying mutations in both canonical and non-canonical NF-κB pathways [12, 18, 19, 27, 161]. Dual targeting of NF-κB pathways has been successfully demonstrated for multiple myeloma *in vitro* and in a xenograft model [277, 278].

Combination therapy simultaneously targeting NIK and IKKβ (as a main kinase of the canonical NF-κB pathway), either using the selective NIK inhibitors (AM-0216 or AM-0561) and a small molecule IKKβ inhibitor (MLX) [278] or the promising dual inhibitor of NIK and IKKβ, PBS-1086 [277], showed significant anti-multiple myeloma activity, associated with apoptosis and inhibition of both NF-κB pathways in tumor cells *in vitro* [277, 278] and in a mouse xenograft model of human multiple myeloma [277]. To our best knowledge, there are no clinical trial studies ongoing, which evaluate the safety and efficacy of NIK inhibitors in patients with newly diagnosed or relapsed/refractory DLBCL.

### Inhibition of NF-κB signaling and reactivation of a lethal type I interferon response in the BCR-subtype of GCB and ABC-DLBCL by targeting cereblon

Recent preclinical study demonstrated that the thalidomide-like drug lenalidomide is preferentially suppressing the proliferation and survival of ABC-DLBCL subtypes with minimal effects on non- ABC-DLBCL [90, 91]. Thalidomide-like immunomodulatory agents such as lenalidomide or pomalidomide, are clinically important drugs for multiple myeloma and other B-cell malignancies [279–281]. The activity of lenalidomide in ABC-DLBCL has at least two postulated mechanisms: inhibition of BCR-mediated NF-κB-dependent pro-survival signaling pathways in and expression of trancription factor IRF4, which in turn leads to the upregulation of the STAT2/type I interferon death pathway [90, 91]. IRF4 overexpression has been shown to enhance NF-κB activation and BCR signaling [90, 91]. The lenalidomide-mediated reduction of IRF4 requires the E3 ubiquitin ligase complex coreceptor protein cereblon (CRBN) [90, 91]. CRBN a substrate receptor of the Cul4-Rbx1-DDB1-CRBN E3 ubiquitin ligase complex, is a direct target of the immunomodulatory drugs thalidomide, lenalidomide and pomalidomide [282, 283]. Thalidomide-like drugs directly bind to CRBN and promote the recruitment of its common substrates such as transcription factors Aiolos and Ikaros to the E3 ubiquitin ligase complex, thus leading to substrate ubiquitinylation and degradation [284] and subsequent repression of IRF4 and SPIB [90, 91]. Repression of IRF4 and SPIB by lenalidomide induces a lethal type I interferon response in ABC-DLBCL by augmenting interferon β (IFNβ) production [90]. IRF4 and its regulatory partner SPIB prevent IFNβ production by repressing IRF7 in ABC-DLBCLs [90]. The STAT2/type I interferon (IFNα/β) axis is well known to have a tumor suppressor function in B-cell lymphoma *in vitro* [285, 286] and inhibits tumor growth *in vivo* [287]. Loss of the STAT2-IFNα/β axis confers resistance to apoptosis induced by chemotherapeutic drugs in B-cell lymphoma cell lines [285, 286]. However, due to their high toxicities, IFNα and -β have not yet been accepted as clinically useful agents in patients with aggressive B-cell lymphoma.

Multiple phase I/II and phase II clinical trial studies revealed that lenalidomide is well tolerated and produces already as single agent durable responses in patients with aggressive relapsed/refractory ABC-DLBCL, leading to an overall response rate (ORR) of up to 53 % and a complete response rate (CR) of up to 23 % in non-GCB-DLBCL (including ABC-DLBCL cases) (Additional file 1: Table S2) [288–293]. In line with the reported efficacy of bortezomide and ibrutinib for non-GCB-DLBCL, patients with non-GCB-DLBCL showed a higher response to lenalidomide in relapsed/refractory DLBCL (ORR 52.9 %) when compared to patients with GCB-DLBCL (ORR 8.7 %) (Additional file 1: Table S2) [288]. In an additional separate ongoing multicenter, randomized clinical phase II/III study, patients with refractory ABC-DLBCL treated with lenalidomide, when compared to GCB patients, showed greater improvements in ORR (45.5 % vs. 21.4 %), PFS (82 weeks vs. 13.2 weeks) and OS (108.4 weeks vs. 30 weeks) [294] (Additional file 1: Table S2). Recent phase I/II clinical trial studies have demonstrated that the combination of lenalidomide with R-CHOP is safe and efficacious, particularly in elderly patients [291, 295–299] (Additional file 1: Table S5). Several randomized phase III studies evaluating the use of lenalidomide with or without R-CHOP in patients with newly diagnosed DLBCL as well as in patients with relapsed/refractory DLBCL are currently ongoing (NCT01122472 (REMARC), NCT02285062 (ROBUST), NCT02128061, NCT01197560) [281] (Table 9 and Additional file 1: Table S2). 

A recent study performed by Hagner P.R. et al. demonstrated that CC-122, a novel immunomodulatory agent-like thalidomide analog, directly binds to CRBN and promotes ubiquitinylation and degradation of Aiolos and Ikaros in DLBCL *in vitro*, *in vivo* and in patients with relapsed/refractory DLBCL, resulting in a mimicry of lethal IFN type I signaling through direct de-repression of interferon-stimulated gene (ISGs) transcription and induction of interferon inducible proteins, and ultimately leading to apoptosis in DLBCL [300]. Surprisingly, CC-122 emerges with features that differentiate it from family member of thalidomide analogs. The anti-lymphoma activity of CC-122 was independent of the cell of origin and observed in both ABC- and GCB-DLBCL cell lines, in contrast to the ABC-subtype selective activity of lenalidomide [300]. CC-122 has therefore been suggested to belong to a new class of drugs: pleiotropic pathway modifiers [300, 301]. These novel properties make CC-122 potentially clinically active in the GCB- subtype of DLBCL in which its predecessor, lenalidomide, has only limited or even no activity [300].

At least three possibilities have been suggested to explain the differential activity of CC-122 and lenalidomide [300, 301]. First, CC-122 may promote the recruitment, ubiquitination and degradation of specific and unique substrates to mediate some of its biological effects distinct from lenalidomide [300]. Secondly, Aiolos and Ikarus, both known co-repressors of ISG transcription may act independently of IRF4 and interferon secretion in GCB- and type-3 DLBCL [300]. Thirdly, CC-122 may promote the upregulation of the STAT2/type I interferon death pathway independently of IRF4 in GCB-DLBCL [301]. Moreover, other potential immunomodulatory mechanisms for its activity in (GCB)-DLBCL likely do exist and may impact the nonimmune environment *in vivo*, in patients as well [301]. CC-122 has already demonstrated clinical activity as single-agent in DLBCL [300–302, 303]. CC-122 is currently in phase I trials in patients with newly diagnosed DLBCL or relapsed/refractory DLBCL, either as a single-agent (NCT01421524) [300–302, 303] (Additional file 1: Table S2) or in combination with the novel dual mTORC1/mTORC2 inhibitor CC-223 [304], the novel BTK inhibitor CC-293 [305] and/or rituximab (NCT02031419) as well as in combination with obinutuzumab, (NCT02417285) [303] (Additional file 1: Tables S5 and S14).

Of interest, Shi C.X. et al. recently demonstrated that proteasome inhibitors such as bortezomib and carfilzomib can block Ikaros degradation by lenalidomide in multiple myeloma, when concomitantly added to the lenalidomide treatment [306]. These data suggest that administration of thalidomide-like agents concurrent with or shortly after proteasome inhibitor administration might be ineffective or at least strongly reduce the efficacy of thalidomide-like agents in DLBCL.

### Inhibition of JAK2/STAT3 in ABC-DLBCL

The JAK/STAT3 signaling pathway represents another potential therapeutic drug target for relapsed or refractory ABC-DLBCL. Constitutive STAT3 activation has been recently correlated with poor overall survival in patients with ABC-DLBCL subtype treated with R-CHOP [307–310]. Inhibition of constitutive STAT3 activity sensitizes resistant B-cell NHL cells to chemotherapeutic cytotoxic drugs, including CHOP, cisplatin, fludarabine, adriamycin, and vinblastine [164, 171]. STAT3 is persistently phosphorylated (pSTAT3-Y705) in most ABC-DLBCL in an autocrine and paracrine manner (from the tumor microenvironment) [311–313]. Inactivating STAT3 in ABC-DLBCL cells inhibits cell proliferation and triggers apoptosis *in vitro* [312–314]. Small molecule inhibitors of STAT3 signaling (inhibitors of nuclear translocation or JAKs) are currently under investigation whether they could serve as drugs for relapsed and/or refractory ABC-DLBCL [171, 311, 315] (Additional file 1: Table S6). A phase I/II is currently being performed in patients with advanced tumors, including refractory DLBCL, evaluating the clinical efficacy and safety of ISIS-STAT3Rx, an antisense oligonucleotide, which inhibits nuclear translocation of STAT3 (NCT01563302). Clinical grade oral inhibitors of JAK1 and JAK2, such as fedratinib (SAR302503/TG101348) or ruxolitinib, which blocks phosphorylation of STAT1 and STAT3 [60] have been recently proposed for clinical evaluation for the treatment of ABC-DLBCL [60, 316, 317]. A multicentre phase II study of ruxolitinib in patients with relapsed/refractory DLBCL is ongoing (NCT01431209) [109].

Moreover, a phase I study evaluating the safety and efficacy of the novel JAK1 inhibitor INCB039110 [318] has been recently started in relapsed/refractory B-cell lymphoma, including DLBCL (NCT01905813) (Additional file 1: Table S6). In addition, the novel macrocyclic pyrimidine-based selective oral inhibitor of JAK2/JAK2(V617F) and FLT3 kinases, pacritinib (SB1518), which blocks phosphorylation of STAT1, STAT3 and STAT5 has also been suggested for further investigations in clinical studies of patients with relapsed/refractory DLBCL [317, 319, 320] (Additional file 1: Table S6). A phase I dose-finding and pharmacokinetic/pharmacodynamic study of pacritinib (SB1518) has shown safety and early clinical activity in patients with relapsed B-cell lymphoma, providing the first proof of principle of the potential clinical value of targeting JAK/STAT pathway in B-cell lymphoma [317, 321]. Unfortunately, no responses were observed in the five patients with relapsed/refractory DLBCL, which was explained by the small number of patients [317, 321] (Additional file 1: Table S6). To date, pacritinib (SB1518) is the first and only JAK2 inhibitor that has been already evaluated in patients with relapsed/refractory DLBCL [317].

HDAC inhibitors can also enhance dephosphorylation of STAT3 and are evaluated in ongoing clinical trials in DLBCL, including relapsed/refractory DLBCL (see next sections). Moreover, a recent study provided evidence that STAT3 mediated proliferation and survival of DLBCLs also depends on IL10/IL10-receptor signaling [322, 323], suggesting IL10-receptor (IL10R) as a novel JAK2/STAT3 pathway-specific therapeutic target in DLBCLs [322]. Inhibition of IL10R signaling with an anti-IL10R-blocking antibody induced dose-dependent cell death in all tested ABC-DLBCL cell lines and primary DLBCLs [322, 323]. Anti-IL10R treatment resulted in interruption of the IL10/IL10R-STAT3 auto-stimulatory loop [322].

### Inhibition of PI3K/AKT/mTORC1 and PI3K/mTORC2/PKC/AKT pathways in GCB-DLBCL and BCR-subtype of ABC-DLBCL

The constitutive activation of the PI3K/AKT/mTORC1 pathway in GCB-DLBCL plays a central role in promoting survival and chemotherapy-resistance and represents a rational therapeutic target in relapsed or refractory GCB-DLBCL [324]. Deregulation of the PI3K/AKT/mTORC1 pathway by the inactivation of PTEN, an inhibitor of the PI3K/AKT/mTORC1 pathway, is found in 55 % of GCB-DLBCL cases, but only in 14 % of non-GCB-DLBCL and worsens prognosis [324]. In preclinical *in vitro* studies, inhibitors of PI3K, such as LY294002 selectively targeted PTEN- deficient GCB-DLBCL cells [324, 325]. In addition, inhibition of target of rapamycin complex 1 (mTORC1) or PI3K blocks proliferation and induces cell death in BCR-subtype of ABC-DLBCL [133, 232, 233]. Idelalisib (formerly GS-1101 and CAL-101), a selective oral reversible inhibitor of the p110δ isoform of PI3K, is currently evaluated in an ongoing early phase I study as a single-agent in patients with relapsed/refractory DLBCL. Moreover, an ongoing early dose escalation and dose expansion phase I study is evaluating the safety and efficacy of idelalisib in combination with the novel BTK inhibitor ONO*-*4059 in patients with relapsed/refractory ABC-DLBCL (Additional file 1: Table S14). In addition, MK-2206, an AKT inhibitor and BKM-120, a pan-class I PI3K inhibitor, are currently being tested in early clinical phase I trials in DLBCL, including relapsed/refractory DLBCLs (Additional file 1: Table S2).

However, new drugs targeting the PI3K/AKT/mTORC1 pathway appears to have only modest activities as a single-agent in treating patients with relapsed/refractory DLBCL and many patients experience severe side effects [326–328]. For instance, recent phase II trial studies of the oral mTORC1 inhibitors everolimus (RAD001) and temsirolimus, two analogues of the parental compound rapamycin, showed a very modest ORR of 30 to 38 % and CR of only 12 % for DLBCL [326–328] (Additional file 1: Tables S2 and S5). An ongoing randomized clinical phase III study is further evaluating everolimus in patients with relapsed/refractory DLBCL (NCT00790036) (Table 9). The modest activity observed in these clinical studies has been explained in two ways: First, blockade of PI3K/AKT/mTORC1 pathway induces autophagy [329, 330], which can serve as a protective mechanism to mitigate the cytotoxicity of drugs targeting the PI3K/AKT/mTORC1 pathway in DLBCL cells [329]. Autophagy can also serve as a protective mechanism to survive from chemotherapeutic-induced genotoxic stress [331]. Inhibition of autophagy has been shown to enhance chemotherapy-induced cell death [331] and enhance the toxic activity of drugs targeting the PI3K/AKT/mTORC1 pathway in DLBCL cells [329]. Secondly, the weak activity of rapamycin analogues can also be explained by their mTORC1-selective inhibitor activity. Both everolimus and temsirolimus target only the mTORC1 but not mTORC2. mTORC2 is generally considered to be unaffected by rapamycin and produces resistance at least partly via the induction of upstream receptor tyrosine kinase signaling and phosphorylation of AKT on S473, a critical regulatory site that stimulates maximal activity of this important survival kinase [332–334]. Thus, it is hypothesized that dual mTOR kinase inhibitors, blocking both PI3K/AKT/mTORC1 and PI3K/TORC2/PKC/AKT signaling pathways to prevent feedback loops, may have expanded therapeutic potential [304]. Pre-clinical studies have already demonstrated that dual inhibition of mTORC1/2 can overcome rapamycin and/or temsirolimus resistance in solid cancer types [335]. An ongoing phase I/II, multi-center study evaluating the safety and efficacy of the novel dual mTORC1/mTORC2 inhibitor CC-223 [304] in patients with relapsed/refractory tumors, including DLBCLs, is currently testing this hypothesis (NCT01177397) [304]. A preclinical study performed by Mortensen D.S. et al. provided preliminary evidence that CC-223 can strongly inhibit the growth of GCB-, ABC- and type-3 DLBCL cell lines associated with high mTORC1 and mTORC2 activity *in vitro* [304]. Of interest, these data suggest that ABC-DLBCL with high IRF4 tend to be less sensitive towards CC-223 [304]. In addition, a phase I study evaluating the efficacy and safety of the dual mTORC1/2 inhibitor OSI-027 in patients with solid cancer and B-cell lymphoma, including DLBCL has been recently completed and is pending announcement of the results (NCT00698243) [336]. OSI-027 is a novel, highly selective, dual mTORC1/2 inhibitor that inhibits the phosphorylation of mTORC1 and mTORC2 [336]. A previous preclinical study showed that OSI-027 markedly diminished proliferation and induced apoptosis in a variety of lymphoid cell lines and induced tumor regressions in B-cell lymphoma xenograft models [336]. Moreover, using *in vitro* screening, Ezell S.A. et al. recently demonstrated that the combination of ibrutinib and AZD2014, a novel dual catalytic inhibitor of mTORC1/mTORC2, is highly synergistic in killing ABC-subtype DLBCL cell lines, weakly expressing IRF4 [232]. Simultaneous inhibition of BTK and mTORC1/2 caused apoptosis both *in vitro* and *in vivo* and resulted in tumor regression in a xenograft model [232].

Taken together, despite their modest activity as single-agents both everolimus and temsirolimus might be targeted as a clinical strategy for re-sensitization to (R-)CHOP based chemotherapy [329, 337, 338]. Ongoing clinical phase I/II studies are evaluating the safety and activity of salvage therapy consisting of the oral mTORC1 inhibitors everolimus and temsirolimus, added to standard therapy of rituximab or R-CHOP, respectively, in patients with newly diagnosed or relapsed/refractory DLBCL (Additional file 1: Tables S4 and S5).

### Inhibition of programmed death-1 (PD-1) signaling in (HR-like)-subtypes of ABC-DLBCL

Programmed death 1 (PD-1) is an inhibitory receptor expressed on the surface of T cells that functions in conjunction with receptor ligands, PD-L1 and PD-L2 to physiologically limit T-cell activation and proliferation [339]. Its ligands, PD-L1 and PD-L2, are expressed on antigen-presenting cells [339]. Binding of PD-L1 or PD-L2 to its receptor inhibits T-cell activation and counterbalances T-cell stimulatory signals, thus primarily limits the T-cell response in peripheral tissues [339]. The sustained expression of PD-1 and the receptor ligands result in T-cell exhaustion and immune escape [339, 340]. This mechanism has been adopted by tumors to prevent antitumor activity in tumor-infiltrating lymphocytes that are present in the tumor microenvironment [340].

The PD-1/PD-L1 pathway plays an important role in tumor immune evasion [340]. PD-L1 expression is either driven by direct oncogenic signaling or upregulated on the tumor cell surface via induction by IFNγ or other inflammatory cytokines, as occurs in the course of the normal immune response [340]. IFNγ/cytokine induced expression of PD-L1 is mainly mediated through JAK2-STAT1 and/or STAT3 respectively [341–343]. PD-L1 is aberrantly overexpressed in subsets of aggressive GCB- and ABC-DLBCL (10–14 % of DLBCL-NOS) with worse prognosis, most likely belonging to the HR-subtype of ABC-DLBCL [344–348]. A recent phase II study of the anti-PD-1 antibody pidilizumab (NCT00532259), administered to patients after ASCT for relapsed/refractory DLBCL demonstrated an overall response rate of 51 % in patients with DLBCL [349] (Additional file 1: Table S2). 34 % of patients who had residual disease after ASCT experienced a complete response [349]. The 16-month progression-free survival was 72 % in the population as a whole and 70 % in high-risk patients [349]. These data suggest that PD-1 is a very promising target in the treatment and management of relapsed/refractory DLBCL. A phase II clinical trial is currently ongoing to evaluate the role of the anti-PD-1 antibody nivolumab in relapsed/refractory DLBCL [344] (Additional file 1: Table S2). There are also efforts to combine anti-PD1 agents with other drugs [344] (Additional file 1: Table S2). For instance, a phase I/II clinical trial evaluating the efficacy and safety of the anti-PD-1 antibody MEDI4736 in combination with ibrutinib in patients with relapsed/refractory DLBCL is currently ongoing (Additional file 1: Table S2).

### Inhibition of EZH2 in GCB-DLBCL

The polycomb-group oncogene product enhancer of zeste homologue 2 (EZH2) is a histone methyltransferase and plays a key role in transcriptional repression through chromatin remodeling [350]. The EZH2Y641F has now been reported as a gain-of-function mutation in > 21 % of GCB-DLBCL and is essentially absent from ABC-DLBCL [29]. The Y641F mutation in EZH2 results in altered histone-lysine methyltransferase activity [351, 352]. The EZH2Y641F mutation can cooperate with c-MYC to accelerate lymphomagenesis in animal models and is implicated in drug resistance [353]. In addition, EZH2 cooperates with BCL2 and BCL6 to create the GCB phenotype and induce B-cell lymphomas through formation and repression of bivalent chromatin domains [77, 79]. Several recent preclinical studies demonstrated that potent and selective S-adenosyl-methionine-competitive small molecule inhibitors of EZH2 such as E7438 (EPZ-6438), GSK-126 or CPI-360 eliminate tumor growth in GCB-DLBCL models with activating EZH2 mutations [77, 354–356]. GSK-126 is selectively targeting the activating oncogenic mutant form of EHZ2 [355]. GSK-126 affected the viability of mutant EZH2-containing GCB-DLBCL cells *in vitro* and in mouse xenograft models with EZH2 mutations *in vivo* but not of wild-type (WT) EZH2-containing GCB-DLBCL cells or in mouse WT-EZH2 xenograft models [355]. On the other hand inhibitors such as CPI-360 are broadly efficacious also in GCB-DLBCL models with wild-type EZH2 [356]. Thus pharmacological inhibition of EZH2 activity may provide a promising treatment for relapsed or refractory GCB-DLBCL overexpressing EZH2 wild-type and/or mutants. Moreover, a recent preclinical study provided evidence for synergistic anti-tumor activity of the EZH2 inhibitor E7438 (EPZ-6438) and glucocorticoid receptor agonists in models of GCB-DLBCL *in vitro* [357]. A phase I/II clinical trial study of E7438 (EPZ-6438) in NHL including newly diagnosed DLBCL is currently ongoing (NCT01897571) [354, 357].

### Inhibition of BCL2, BFL-1 and MCL1 in GCB- and ABC-DLBCL

Deregulation of members of the B-cell lymphoma (BCL)-2 family of pro- and anti-apoptotic proteins are associated with rituximab and/or chemotherapy resistance in DLBCL-NOS [358–363]. BCL2, BCL2-related factor (BFL)-1 and myeloid leukemia cell differentiation protein (MCL)-1 represent novel therapeutic targets for relapsed or refractory GCB- and/or ABC-DLBCL, respectively [360–364]. BCL2 is overexpressed in approximately 40–65 % of DLBCL tumors, owing to t(14;18)(q32;q21) translocations found in 15–30 % of cases, and through additional mechanisms that are not well defined [75, 107, 144, 145, 360, 362, 365, 366]. BCL2 is frequently overexpressed in both GCB- and ABC-DLBCL, albeit the mechanisms of BCL2 upregulation are different between GCB- and ABC-DLBCL [75, 362, 365]. ABC-DLBCLs overexpress MCL1 at significantly higher levels compared with GCB-DLBCL, showing IHC positivity in 50 % of ABC and 30 % of GCB tumors [363]. Recent studies have suggested that overexpression of BCL2 remains a negative predictor of outcome after rituximab-based chemotherapy mainly in GCB-DLBCL [360, 361] while MCL1 mainly contributes to chemotherapy resistance in ABC-DLBCL [363]. On the other hand, in presence of c-MYC overexpression, BCL2 overexpression also contributes to a decreased survival of ABC-DLBCL after rituximab-based chemotherapy [107].

Structure-based design was used for the development of small-molecule BH3 mimetics that bind to the BH3 hydrophobic-binding groove of the anti-apoptotic proteins BCL2, B-cell lymphoma-extra large (BCL-XL), BCL2-related protein A1 (BCL2A1/A1) and MCL1 and promote apoptosis [367]. Small-molecule BH3 mimetics include the clinically relevant agents ABT-737, ABT-263 (navitoclax), ABT-199 and GX15-070 (obatoclax) [367–370]. ABT-737 and its oral derivative A263 bind to BCL2, BCL-W and BCL-XL, but not to MCL1, BFL1 or A1 [367, 369, 370], whereas GX15-070, a pan-BCL2 inhibitor also binds to and inactivates MCL1 [367, 368]. Preclinical studies indicate that ABT-737, ABT-263 and GX15-070 are effective against GCB- and/or ABC-DLBCL cells *in vitro*, including bortezomib-resistant DLBCL cells and substantially suppressed tumor growth *in vivo* [363, 371–373]. ABT-263 and GX15-070 have been suggested to be effective in patients with relapsed/refractory DLBCL [363, 371]. However, on-target BCL-XL inhibition by ABT-263 and GX15-070 led to dose-dependent thrombocytopenia and posed a barrier to maximizing the activity of these agents [374]. Moreover, a recent preclinical study suggests that patients may eventually develop ABT-737-resistant disease by up-regulating the expression of MCL1 and BFL1 [364]. ABT-199 (venetoclax), a second-generation orally available derivative of ABT-737 that selectively targets BCL2 is currently under evaluation in clinical trials of B-cell NHL [367, 374, 375]. ABT-199 has greater than 100-fold selectivity for BCL2 over BCL-XL [375, 376].

Preclinical and early clinical studies demonstrated that ABT-199 inhibits the growth of aggressive c-MYC-driven mouse B-cell lymphomas and human BCL2-dependent B-cell lymphoma tumors *in vivo* without causing thrombocytopenia [375, 377]. Of interest, a recent study provided preliminary evidence that normal, untransformed mature B cells may also be sensitive to ABT-199, both *in vitro* and *in vivo* [378]. A preclinical study showed that single-agent ABT-199 had only modest antitumor activity against most DLBCL lines and resulted in compensatory upregulation of MCL1 expression [379]. Moreover, in a phase I clinical trial study, used as a single-agent, ABT-199 has shown a modest overall response rate of up to 33 % in DLBCL, with a complete response rate of only up to 11 % [380–382]. On the other hand, treatment of high-risk DLBCL with ABT-199 combined with the potent new cyclin-dependent kinase inhibitor dinaciclib [383], which knocks down MCL1 by inhibiting CDK9 [379, 383], synergistically induced tumor regression, in xenografts and in a genetically accurate murine model of c-MYC/BCL2 double-hit B-cell lymphoma [379]. A phase II study is currently ongoing combining ABT-199 with bendamustine and rituximab in relapsed/refractory NHL including relapsed/refractory DLBCL (NCT01594229) (Additional file 1: Table S5).

### Inhibition of BCL6 in the BCR-subtype of GCB- and ABC-DLBCL

B-cell lymphoma protein BCL6 overexpression inhibits apoptosis induced by chemotherapeutic agents in DLBCL [32]. BCL6 is overexpressed in both GCB- and ABC-DLBCL, albeit through different mechanisms [32]. Recent studies demonstrated that HSP90 forms a complex with BCL6 and inhibition of HSP90 with the drug PU-H71, a purine scaffold HSP90 inhibitor destabilizes BCL6 and selectively kills BCL6-positive DLBCL cells *in vitro* and *in vivo* [384]. Subsequent studies demonstrated that small molecule inhibitors, including the retro-inverted BCL6 peptide inhibitor (RI-BPI, 79–6) that directly antagonize BLC6 function by disrupting the BCL6-corepressor complexes via binding in the lateral groove of the BCL6 BTB domain and thereby selectively inhibiting the interaction with nuclear receptor co-repressor BCOR, NCOR1 and NCOR2 proteins [123, 385, 386]. The small-molecule inhibitor mediated disruption of the activity of BCL6, can be selectively toxic towards high-risk BCL6-dependent BCR-subtypes of GCB and ABC-DLBCL *in vitro* and potently suppressed GCB-DLBCL tumors in a DLBCL xenograft mouse model *in vivo* through reactivating pro-apoptotic genes repressed by BCL6 [123, 385, 386]. RI-BPI mediated inhibition of BCL6 also induces the expression of EP300, resulting in acetylation and activation of TP53 and concomitant acetylation and inactivation of HSP90 [386]. Moreover the BCL6 activity can also be indirectly blocked in DLBCLs by pharmacologic inhibition of both HDACs and SIRT1/2 [387–389], see next sections. Together, BCL6 represents a novel promising therapeutic target in relapsed/refractory BCR-subtypes of GCB- and ABC-DLBCL. To our best knowledge, there are no clinical trial studies ongoing, which evaluate the safety and efficacy of HSP90 or BCL6 inhibitors in patients with newly diagnosed or relapsed/refractory DLBCL.

### Inhibition of HDACs and Sirtuins in GCB- and ABC-DLBCL

Several recent studies identified small inhibitory molecules targeting histone deacetylases (HDACs) and sirtuins as promising potential therapeutic agents in relapsed/refractory GCB- and ABC-DLBCLs [387]. SIRT1 expression is associated with poor prognosis in DLBCL [390]. Several HDAC inhibitors (HDACi) are already approved for clinical use or in clinical trials [391]. HDACi and sirtuin inhibitors can target both GCB- and ABC-DLBCL, albeit through different mechanisms [387, 392]. Various HDACi and sirtuin inhibitors can repress GCB-DLBCL as a result of their inhibition of the BCL6 oncogene [386–389]. Inhibition of both HDACs and SIRT1 results in the accumulation of acetylated BCL6 [388]. Acetylation of BCL6 inhibits the ability of BCL6 to recruit HDAC-containing SMRT co-repressor complexes [388]. Thus, inhibition of HDACs and Sirtuins in BCL6-positive GCB-DLBCLs (and to a minor extend in ABC-DLBCL) results in the accumulation of inactive acetylated BCL6 and eventually in cell cycle arrest and apoptosis [386, 388]. Moreover a recent preclinical and clinical study demonstrated that combined sirtuin and pan-HDAC inhibition synergistically kills DLBCLs with a preference for GCB-DLBCL [389]. Combined treatment of DLBCL cells with HDACi such as vorinostat in combination with the Sirtuin inhibitor niacinamide produced synergistic cytotoxicity *in vitro* and *in vivo* by inhibiting BCL6 and activating TP53 [389]. Acetylation of p53 strongly stimulates its pro-apoptotic activity [393]. A subsequent proof-of-principle phase I clinical pilot trial in patients with relapsed or refractory NHL, including DLBCL led to an ORR of 18 % and CR of 18 % [389]. 46 % of the patients experienced stabilization of their aggressive disease [389]. In addition, a study performed by Gupta M. et al. demonstrated that HDACi such as LBH589 can effectively suppress STAT3 in ABC-DLBCL [392]. Inhibition of HDACs leads to increased acetylation of STAT3, dephosphorylation of pSTAT3(Y705), nuclear export of STAT3 to the cytoplasm and blocks survival of ABC-DLBCL cells [392]. Inhibition of SIRT1 has also been shown to induce dephosphorylation of pSTAT3(Y705), nuclear export of STAT3 to the cytoplasm and thereby inactivation of STAT3 [394]. Preliminary data from a clinical phase II trial study indicate that HDACi might not work as single-agents in relapsed/refractory GCB and ABC-DLBCL [395]. Despite an encouraging activity of the HDACi vorinostat in DLBCL was noted in a phase I trial study [396], subsequent clinical phase II trial studies of oral vorinostat in relapsed DLBCL showed only limited activity against relapsed DLBCL [395]. Although vorinostat seems not to be very effective as a single-agent, other more potent pan-HDACi such as romidepsin, panobinostat, MGCD-0103, LBH589 have been suggested as rational therapeutic options for clinical trials in relapsed/refractory DLBCL when combined with other agents [1, 397].

### Inhibition of BRD4 (and BRD2) in the c-MYC-driven subtype of DLBCL-NOS

High c-MYC expression correlates with inferior clinical outcome in R-CHOP-treated DLBCL patients [107, 138, 366]. C-MYC-driven gene (over)expression has been suggested to confer resistance to rituximab immunotherapy [107, 138, 366, 398]. Recent preclinical studies demonstrated that the transcriptional coactivator and bromodomain and extra terminal (BET) protein BRD4 is required for transcriptional amplification of the *c-MYC* oncogene [398–402]. BRD4 occupies a small set of exceptionally large super-enhancers associated with genes including the *c-MYC* oncogene [399, 403]. Inhibition of BRD4 (and other BRD family members) by small molecule inhibitors of the BET family, such as the BET-bromodomain inhibitor JQ1, leads to preferential inhibition of *c-MYC* oncogene expression, growth arrest and extensive apoptosis in a variety of B-cell leukemia and lymphoma cell lines, including c-MYC/BCL2-double-hit DLBCLs and AML cells that overexpress c-MYC in association with BCL2 [398–401, 404]. I-BET151, another novel highly selective inhibitor of both BRD2 and BRD4 causes transcriptional silencing of the *c-MYC* gene and c-MYC-dependent programs in myeloma cells by inhibition of BRD2 and BRD4 binding to the promoter of the *c-MYC* gene and failure to recruit P-TEFb and PAFc1 complexes to the *IGH/c-MYC* enhancer by BRD2 and BRD4, and lack of subsequent RNA-Pol-II recruitment [405]. Both JQ1 and PFI-1, another highly selective inhibitor of BRD4 and BRD2, also displayed significant antitumor activities *in vivo* in xenograft models of DLBCL, Burkitt’s lymphoma or acute myeloid leukemia and thereby improved survival of engrafted mice [399–402, 406]. Another preclinical study recently demonstrated that the new BET bromodomain inhibitor OTX015 affects pathogenetic pathways in preclinical B-cell tumor models, including GCB-, ABC-and type-3 DLBCL-NOS [407]. OTX015 showed strong anti-proliferative activity in a large panel of cell lines derived from mature B-cell lymphoid tumors [407]. Interestingly, in addition to its broad cytostatic activity, OTX015 selectively induced apoptosis in a genetically defined subgroup of cells, derived from ABC-DLBCL, bearing wild-type TP53 and mutations in MYD88, CD79B and/or CARD11 [407]. OTX015 targets and inhibits canonical NF-κB, TLR and JAK/STAT3 signaling pathways, as well as c-MYC- and E2F1-regulated genes in DLBCL *in vitro* and *in vivo* [407]. As already mentioned above, inhibition of BRD2 and BRD4 by CPI203 and JQ1 could also inhibit the oncogenic NF-κB activity and kill ABC-DLBCL cells [239]. Surprisingly, this effect seems to be independent of c-MYC since ectopic provision of c-MYC did not rescue ABC-DLBCL cells from JQ1 toxicity [239]. Remarkably, Eμ-*Brd2* transgenic mice developed predominately ABC-like DLBCLs [408]. Confirming previous preclinical studies [398, 399], Boi M. et al. also showed that BET bromodomain inhibition by OTX015 increases rituximab sensitivity in DLBCL cells [407]. At least four small-molecule bromodomain inhibitors of BET are in ongoing oncology/hematology early phase I clinical studies (CPI-0610, NCT01949883; GSK525762, NCT01587703; OTX015, NCT01713582 and TEN-010, NCT01987362), which include relapsed/refractory DLBCL (CPI-0610; NCT01949883) (Additional file 1: Table S2). The first results from a phase I study with the orally available BET bromodomain inhibitor OTX015 have been reported with clinical responses in both leukemia and B-cell lymphoma patients in the absence of major toxicities [409, 410].

Of interest, a recent study performed by Lu J. et al. showed that JQ1 and OTX015 can lead to BRD4 protein accumulation over time in Burkitt lymphoma (BL) cells and incomplete c-MYC suppression *in vitro* [411]. Whether these effects may also occur in DLBCL remain to be investigated. Remarkably, three recent studies provided a novel strategy, which can circumvent these limitations by generating bifunctional phthalimide or VHL-ligand-conjugated versions of JQ1 and OTX015 [411–413]. Lu J. and colleagues designed a hetero-bifunctional proteolysis targeting chimera (PROTAC), ARV-825, by connecting the small-molecule BRD4 binding moiety OTX015 to the E3 ligase cereblon binding moiety of pomalidomide that recruits BRD4 and BRD2 to the E3 ubiquitin ligase cereblon, leading to fast, efficient, and prolonged degradation of BRD4 and BRD2, effective suppression of c-MYC signaling, inhibition of cell proliferation and apoptosis induction in BL *in vitro* [411]. In the second study, Winter G.E. et al. generated a bifunctional thalidomide-conjugated version of JQ1, termed dBET [412]. Treatment of acute myeloid leukemia (AML) cells with dBET1 induced highly selective cereblon-dependent protein degradation of BET family members *in vitro* and *in vivo*, resulted in transcriptional downregulation of MYC, induction of antiproliferative responses in leukemia cells *in vitro* and delayed proliferation and leukemia progression in mice, without toxicity, thus underscoring the potential clinical utility of this approach [412]. Moreover, treatment with dBET1 or ARV-825 induced a superior apoptotic response compared with JQ1 or OTX015, respectively in primary AML or BL cells [412]. Pharmacologic destabilization of BRD4 *in vivo* also resulted in improved anti-tumor efficacy in a human leukemia xenograft compared to JQ1, highlighting the potential superiority of BET degradation over BET bromodomain inhibition [412]. Moreover a third study using a BET-specific PROTAC, termed MZ1, that tether JQ1 to a ligand for the E3 ubiquitin ligase VHL, demonstrated preferential degradation of BRD4 over BRD2 and BRD3 at low concentrations and did not observe any degradation of JQ1-specific off-targets by MZ1, thus point to a more BRD4-selective pharmacological profile of BET specific PROTACs compared with pan-selective BET inhibitors JQ1 or OTX015 [413]. Taken together, cereblon-based PROTACs may therefore provide a better and more efficient strategy in targeting BRD4 and BRD2 than traditional small-molecule inhibitors [411, 412]. This chemical strategy for controlling target protein stability of BRD proteins may also have broad implications for therapeutically targeting previously intractable proteins in DLBCL [412].

### Inhibition of PRPS2 in the c-MYC-driven subtype of DLBCL-NOS

Using a mouse model of c-MYC-driven B-cell lymphomagenesis, a recent study uncovered the mechanism by which c-MYC coordinates the nexus between nucleotide metabolism and protein biosynthesis. The authors found that the single rate-limiting enzyme, phosphoribosyl-pyrophosphate synthetase 2 (PRPS2), promotes increased nucleotide biosynthesis in c-MYC-transformed cells [414]. Remarkably the levels of PRPS2 are specifically increased in c-MYC-driven lymphomagenesis and this upregulation tightly correlates with eukaryotic translation initiation factor 4E (eIF4E) expression, which was also directly induced by c-MYC [414]. Moreover, the authors also demonstrated that loss of PRPS2 in the *Prps2* knockout mouse background is synthetically lethal in c-MYC-transformed human and mouse cell lines, but knockout of *Prps2* did not affect wild-type cells or mice [414]. Depletion of PRPS2 levels by siRNA in c-MYC-driven B-cell lymphomas improved survival and induced complete tumor regression in 30 % of mice, strongly indicating that protein and nucleotide biosynthesis controlled by PRPS2 is crucial for c-MYC-driven tumorigenesis [414]. Together, PRPS2 might be a promising and effective druggable target in c-MYC-driven subtype of DLBCL-NOS.

### Reactivation of the pro-apoptotic TGF-β-SMAD pathway through inhibition of DMNTs in relapsed/refractory GCB and ABC-DLBCL-NOS

Several recent studies identified the transforming growth factor-β (TGF-β)-SMAD pathway as a critical target in relapsed/refractory DLBCL [415–418]. By profiling genome-wide DNA methylation at single-base pair resolution in thirteen DLBCL diagnosis-relapse sample pairs and in a GCB-DLBCL cell line, Pan H. et al. identified a relapse-associated DNA methylation signature based on consistently differentially methylated regulatory elements between diagnosis and relapsed pairs [418]. This signature was linked with specific genes and pathways, including the pro-apoptotic TGF-β-receptor-SMAD pathway suggested to play an important role in relapse of DLBCL [418].

These data confirm a previous study indicating that methylation aberrations of TGF-β receptor activity pathway-associated genes might be involved in relapse and chemoresistance in DLBCL [417]. Pan H. et al. demonstrated that prolonged exposure to low-dose DNMT inhibitors (DNMTi) reprogrammed chemoresistant GCB- and ABC-DLBCL cells to become doxorubicin sensitive *in vivo* [417]. Recurrent hypermethylation of the promoter region and reactivation of SMAD1 was a critical contributor and required for chemosensitization [417]. A subsequent phase I clinical pilot study evaluating the priming by DNMT inhibitor azacytidine (AZA) followed by standard R-CHOP in high-risk patients with DLBCL showed a very high rate of complete remission (92 %) and confirmed SMAD1 demethylation and chemosensitization [417]. AZA and 5-aza-2′-deoxycytidine/decitabine (5-AZA/DAC) mainly inhibit DNA methyltransferases DMNT1 and DMNT3 resulting in the re-expression of tumor-suppressor genes [419, 420]. Both studies are in line with a previous report demonstrating that escaping from TGF-β-SMAD5-mediated growth inhibition through microRNA-155-mediated inhibition of SMAD5 is critical to relapse of DLBCL [415, 416]. Together these studies delineate a personalized therapeutic strategy for the clinical use of DNMT inhibitors as activator of the pro-apoptotic TGF-β receptor-SMAD pathway in relapsed/refractory GCB- and ABC-DLBCL. Two recent phase I studies evaluating the efficacy and safety of decitabine in combination with the HDACi vorinostat or valproic acid in patients with relapsed/refractory B-cell lymphoma, including DLBCL has been recently completed and are pending announcement of the results (NCT00109824, NCT00275080) (Additional file 1: Table S14).

### Treatment of relapsed/refractory DLBCL-NOS with antibody drug-conjugates targeting CD19, CD22, CD30 or CD79B positve tumors

Antibody drug-conjugates (ADCs), in which cytotoxic drugs are linked to antibodies targeting antigens on tumor cells, represent promising novel agents for the treatment of malignant B-cell lymphomas. ADCs use antibodies to deliver a potent cytotoxic compound selectively to specific antigen expressing malignant cells, thereby maximizing drug delivery while limiting bystander effects of traditional cytotoxic agents, thus improving the specificity and efficacy of chemotherapeutic agents. Over the past several years, the use of ADCs as targeted chemotherapies has successfully entered clinical practice. Most of ADC developed for B-cell malignancies target CD19 or CD22 [421, 422]. CD19 is the earliest differentiation antigen of the B-cell lineage and uniformly expressed on all types of B-lymphocytes and the vast majority of B-cell malignancies but not on other normal cells, thereby representing an attractive target in B-cell malignancies, including DLBCL [423, 424]. CD19 is a transmembrane protein that forms a signaling complex together with CD21, CD81 and CD225, which decreases the threshold for the activation of B cells mediated by the BCR [425]. There are already at least seven distinct ADCs in preclinical and clinical studies, evaluating their efficacy in DLBCL, including relapsed/refractory DLBCL: SAR3419 [426, 427], MEDI-551 [421], inotuzumab-ozogamicin (INO/CMC-544) [428–430], moxetumomab-pasudotox (HA22, CAT-8015) [421, 431], brentuximab-vedotin (ADCETRIS) [432], pinatuzumab-vedotin (DCDT2980S) [432] and polatuzumab-vedotin (DCDS4501A) [433] (Additional file 1: Table S7).

SAR3419 is an anti-CD19 antibody conjugated to the cytotoxine Maytansine DM4, a potent inhibitor of tubulin polymerization and microtubule assembly [421, 422]. Two recent early phase I multi-dose-escalation safety and pharmacokinetic studies evaluating SAR3419 in patients with relapsed/refractory DLBCL provided preliminary evidence for promising clinical activity in relapsed/refractory DLBCL and demonstrated that SAR3419 can be safely administered to patients with relapsed/refractory DLBCL (Additional file 1: Table S7) [426, 427]. Another CD19-specific ADC currently evaluated in an ongoing phase I study in relapsed/refractory DLBCL is SGN-CD19A (NCT01786135, Additional file 1: Table S7). SGN-CD19A is an affinity-optimized monoclonal anti-CD19 antibody linked to the microtubule disrupting agent monomethyl auristatin E (MMAE) [434]. An interim analysis of this phase I study showed promising activities in patients with relapsed or refractory DLBCL; with an ORR of 35 % a CR of 20 % and a PR of 16 % [434, 435]. Antitumor activity appeared to be higher in relapsed DLBCL with an ORR of 55 %, a CR of 32 % and a PR of 23 % [434, 435]. MEDI-551, an affinity-optimized and afucosylated monoclonal anti-CD19 antibody with enhanced antibody-dependent cellular cytotoxicity (Additional file 1: Table S7). Inotuzumab-ozogamicin is an affinity-optimized monoclonal anti-CD22 antibody linked to the DNA damaging toxin N-acetyl-γ-calicheamicin dimethyl hydrazide (CalichDMH) [430]. Calicheamicin binds to DNA in the minor groove and introduces double-strand DNA breaks, leading to G2/M arrest and subsequent cell death [430]. Promising anti-tumor responses were observed in early stage clinical trials, where inotuzumab ozogamicin was administered either as single-agent or in combination with rituximab [430]. However, subsequent phase II studies evaluating inotuzumab-ozogamicin with rituximab in patients with high-risk relapsed/refractory DLBCL showed mixed results in patients with high-risk relapsed/refractory DLBCL [428, 429]. The observed activities were in one study less than that seen with other standard salvage regimens for transplant eligible patients with DLBCL [428, 429]. In the first phase II study inotuzumab-ozogamicin has shown an ORR of up to 74 % and a CR of up to 50 % [429], while in the second phase II study, the observed activity of inotuzumab-ozogamicin was rather modest with an ORR of 40 % and CR of 21 % (Additional file 1: Table S7) [428]. Ongoing phase II trials in CD22 expressing DLBCLs are examining inotuzumab-ozogamicin as part of chemotherapy combination regimens (Additional file 1: Table S7). An additional CD22-specific ADC under investigation is moxetumomab-pasudotox (HA22/BL22), an affinity-optimized monoclonal anti-CD22 antibody linked to a synthetic ADP-ribosylating Pseudomonas exotoxin (PE38) [421, 422]. PE38 exerts its cytotoxic effect on cells by mono-ADP-ribosylating elongation factor 2, thereby inhibiting protein synthesis and leading to cell death [422]. A recent phase II study evaluating the efficacy and safety of moxetumomab pasudotox in patients with relapsed/refractory B-cell lymphoma, including DLBCL has been recently completed and is pending announcement of the results (NCT01030536). Another promising ADC currently being tested in clinical trials in relapsed/refractory B-cell lymphoma, including DLBCL is brentuximab-vedotin (ADCETRIS). Brentuximab-vedotin is a human CD30-specific antibody-drug conjugate, which consists of the chimeric monoclonal anti-CD30 antibody SGN-30 conjugated to the synthetic microtubule disrupting agent monomethyl auristatin E (MMAE) [421]. After binding to CD30 on the tumor cell surface, brentuximab-vedotin internalizes leading to release of MMAE via proteolytic cleavage and induction of cell-cycle arrest and apoptosis [421]. CD30, part of the tumor necrosis factor (TNF) receptor family, is an ideal target for ADC-based therapy in DLBCL. CD30 is variably expressed in approximately 20-30 % of DLBCL, with a higher frequency in non-GCB-DLBCL [436–438]. A phase II study, evaluating the efficacy of brentuximab-vedotin in relapsed/refractory CD30- expressing NHL, showed significant activity as single-agent in relapsed/refractory DLBCL with an ORR of 44 %, CR of 17 % and with a median duration of 16.6 months thus far [432] (Additional file 1: Table S7). Ongoing phase I and phase II trials in CD30 expressing DLBCLs are examining brentuximab-vedotin after autologous stem cell transplantation, as part of chemotherapy combination regimens (Additional file 1: Table S7).

In two recent clinical phase I studies, Pfeifer M. et al. demonstrated that pinatuzumab-vedotin and polatuzumab-vedotin are active agents for the treatment of patients with GCB, ABC and/or type-3 subtypes of relapsed/refractory DLBCL, respectively (Additional file 1: Table S7) [433]. Pinatuzumab-vedotin is an anti-CD22 ADC and polatuzumab-vedotin an anti-CD79B ADC that are both conjugated to the microtubule-disrupting agent MMAE [421]. Preclinical experiments unexpectedly showed that both pinatuzumab-vedotin and polatuzumab-vedotin are highly active and induced cell death in the vast majority of ABC- and GCB-DLBCL cell lines [433], suggesting that both can be used effectively in DLBCL subtypes without the need for sophisticated molecular testing [433, 439]. In a subsequent phase Ib study evaluating escalating doses of polatuzumab-vedotin in combination with rituximab in patients with relapsed/refractory DLBCL, Palanca-Wessels M.C. et al. demonstrated that polatuzumab-vedotin has an acceptable safety and tolerability profile in patients with relapsed/refractory DLBCL [439]. Polatuzumab-vedotin showed significant activity in relapsed/refractory DLBCL with an ORR of 56 % and CR of 14.8 % [433, 439] (Additional file 1: Table S7). Polatuzumab-vedotin is currently being studied in a phase I trial as a replacement for vincristine in combination with immuno-chemotherapy (R-CHOP) in patients with newly diagnosed DLBCL (NCT01992653) [439] as well as in a phase II study in combination with rituximab or obinutuzumab plus bendamustine in patients with relapsed/refractory DLBCL NCT02257567 (Additional file 1: Table S7).

### Treatment of relapsed/refractory DLBCL-NOS with anti-CD19 or anti-CD20 chimeric antigen receptor T cells

Another potential approach to target chemotherapy refractory DLBCLs are chimeric antigen receptor-modified autologous T cells (CAR T cells) targeted specifically to antigens expressed by B-cell malignancies. T cells that are genetically modified to express chimeric antigen receptors (CARs) recognizing the B cell-associated CD19 or CD20 molecules have emerged as a clinically feasible, potentially potent therapeutic modality and appears to be safe [440–446]. CARs are fusion proteins made up of antigen recognition moieties and T-cell activation domains [440, 447–450]. T cells expressing anti-CD19 CARs are activated in a CD19-specific manner and recognize and kill CD19^+^ primary malignant B cells [440, 441, 443, 451–453]. The CAR T cell based immunotherapy approach serves as a form of adoptive T-cell immunotherapy [440, 454]. For a detailed description of CAR T-cell based therapies, the readers are referred to the recent excellent reviews [440, 441, 443].

Studies of CAR T cells have mainly been performed in multiple myeloma, chronic lymphocytic leukemia and acute lymphocytic leukemias [440–446]. Initial studies on patients with relapsed DLBCL treated with anti-CD20 or anti-CD19 CAR T cells were not very successful, most likely due to a cellular anti-transgene immune response in some of the patients [443, 455]. Moreover previous studies of anti-CD19 CAR T cells showed that multiple patients with indolent B-cell malignancies had specific depletion of normal B cells and lengthy remissions [456–458]. However, interim results of an ongoing study performed by Kochenderfer J.N. et al. on heavily pre-treated patients showed the first patients, which obtained complete remissions (CRs) in chemotherapy-refractory DLBCL after receiving anti-CD19 CAR T cells [459, 460]. Using a significantly changed anti-CD19 CAR T cell production process and modified clinical treatment protocol four of the seven evaluable patients with DLBCL obtained CRs, two obtained PRs, and one had stable disease (SD) after infusion of CAR T cells [460] (Additional file 1: Table S8). Infusion of anti-CD19 CAR T cells was associated with significant but only transient toxicity [460]. In addition, preliminary findings from an ongoing phase IIb study (NCT02030834) evaluating the safety and efficacy of the anti-CD19 CAR-T cells in patients with relapsed or refractory DLBCL and follicular lymphoma showed an ORR of 50 %, a CR of 45 % and a PFS of 37 % at median follow up 6 months with acceptable toxicity in the 12 evaluable patients with relapsed or refractory DLBCL (Additional file 1: Table S8) [461].

Several additional phase I/II studies with CAR T cells directed against CD19^+^ B cells are currently being performed in patients with advanced relapsed/refractory DLBCL after autologous stem cell transplant (NCT01865617, NCT01475058, NCT02431988, NCT02348216, NCT01840566 and NCT02445248) (Additional file 1: Table S8). Moreover, a preliminary report of an ongoing pilot study (NCT01735604) evaluating the efficacy and safety of anti-CD20 CAR T cells in patients with chemotherapy refractory advanced DLBCL showed that five out of six evaluable patients experienced objective responses in this pilot trial [446] (Additional file 1: Table S8). Thus, CAR T cell based immunotherapy approaches represent a promising novel strategy in the treatment of patients with relapsed/refractory DLBCL.

### Acquired (secondary) resistance to novel experimental drugs

Unfortunately, several reports already demonstrated that a small proportion of patients with B-cell malignancies had a relapse during therapies with novel experimental agents, such as ibrutinib, lenalidomide or bortezomib [280, 462–464]. However, the molecular mechanisms of resistance are poorly understood. For instance, recent studies showed that up to 10 to 15 % of patients with chronic lymphocytic leukemia (CLL) [462] or mantle cell lymphoma (MCL) [463] treated with ibrutinib have had a partial response (relapse) due to acquired (secondary) drug resistance to ibrutinib during therapy. A subsequent study demonstrated that the observed acquired resistance to ibrutinib in CLL was due at least in part to recurrent mutations in *BTK* and *phospholipase Cγ2 (PLCγ2)* genes [465].

A cysteine-to-serine mutation in BTK at the binding site of ibrutinib was identified in five patients and three distinct mutations in PLCγ2 were identified in two patients [465]. Functional analysis showed that the C481S mutation of BTK confers resistance to ibrutinib by preventing irreversible drug binding. The R665W, and L845F mutations in PLCγ2 are all potentially gain-of-function mutations that allow autonomous B-cell-receptor activity that is independent of BTK [465]. PLCγ2 is one of the key regulators of the B-cell receptor signaling pathway [110]. Thus, the investigation of the molecular mechanisms underlying the observed secondary resistance to novel agents is of great importance. The identification of novel drug targets that may lead to novel alternative therapeutic strategies for patients with drug resistant relapsed/refractory DLBCL is urgently needed to overcome the arising resistance to novel experimental agents. Molecular profiling of advanced cancer patients participating in targeted therapy trials will be important to identify mutational signatures that may predict for drug sensitivity and guide rational patient specific drug combinations.

### Tumor microenvironment (TME)/cell adhesion–mediated drug resistance

It is increasingly apparent that differences in the local tumor microenvironment (TME) affect survival of patients with DLBCL after treatment with chemotherapeutic regimens [217, 218, 466]. The local TME seems to be an essential player for the development and disease progression of DLBCL and to dictate lymphoma cell growth, response to therapy, as well as resistance of residual lymphoma cells to chemotherapeutic agents [217, 466]. It is thought that specific niches within the DLBCL tumor microenvironment provide sanctuary for subpopulations of DLBCL cells through dynamic stromal cell-tumor cell interactions [217, 466]. Adaptive, reciprocal signaling circuits between tumor cells and supportive fibroblast-like stromal cells of TME via positive feedback/feed-forward loops have been suggested to be essential for the observed TME or environmental-mediated drug resistance (EMDR) [217, 218]. EMDR in resident DLBCL cells has been suggested to result in small foci of residual disease that subsequently develop complex genetic or epigenetic means of acquired resistance in response to the selective pressure of therapy [217, 466]. However, the exact molecular mechanisms involved in EMDR are not yet fully understood in DLBCL and remain to be elucidated.

The dynamic interplay between tumor cells and supportive fibroblast-like stromal cells, mediated through consists of extrinsic signals, which are generated by the lymphoma microenvironment and intrinsic factors encompassing signaling determinants of cell cycle and pro-survival pathways [217, 218, 466]. For instance, it has been recently suggested that chronic and tonic BCR signaling is a central hub for the integration between the extrinsic B cell microenvironment and the intrinsic signaling pathways in B-cell lymphomas [218]. For a detailed review about TME induced chronic BCR signaling in B-cell lymphomas the readers are referred to the recent excellent review [218]. In addition, autocrine and/or paracrine IL6 and IL10, two putative DLBCL survival cytokines, have also been proposed to play an important role in EMDR [217, 218]. Several studies documented that higher levels of serum IL6 and/or IL10 in patients with more aggressive disease correlated with unfavorable prognostic factors included in the international prognostic index (IPI) [323, 467]. As already discussed, IL10 has been recently shown to enhance survival of primary DLBCL cell lines *in vitro* [322, 323]. Signaling downstream of IL6 and IL10 in B-cell lymphoma involves primarily activation of JAK1/JAK2-STAT3 axis and to a lesser extend the JAK1/JAK2-STAT1 or TYK2-MAPK-STAT1 axis [313, 322, 323, 468]. Activation of the JAK1/JAK2-STAT3 and JAK1/JAK2-STAT1 signaling cascades often results in upregulation of survival factors such as BCL2 family members [313, 322, 323, 468], IFN/NF-κB signaling-related immune modulatory factors such as PD-1/PD-L1 [341–343], and/or drug resistance related factors such as DTX3L or ARTD9 [116, 469–471]. Both DTX3L and ARTD9 have been shown to be involved in drug resistance in HR- DLBCL associated with a R-CHOP chemotherapy-induced microenvironment gene expression signature [469, 470, 472], see next sections. For a detailed review about TME or environmental-mediated drug resistance the readers are referred to the recent excellent reviews [217, 218, 466].

### Novel potential drug targets in relapsed and/or chemo-refractory HR-subtypes of GCB-, ABC- and type-3-DLBCL-NOS

Except for the antibodies pidilizumab and nivolumab, both targeting PD-1, no small molecule inhibitors, which specifically target relapsed/refractory HR-subtypes of DLBCLs are yet under investigations. However, several recent studies from different labs identified several candidates, including STAT1, the E3 ubiquitin ligase DTX3L (also known as B-lymphoma and BAL-associated protein (BBAP)) and the B-aggressive lymphoma protein and mono-ADP-ribosyltransferase ARTD9 (also known as BAL1 or PARP9), which may be essentially required for the observed chemotherapy resistance in HR-DLBCL and also play a role in editing or inhibiting the host immune response against HR-DLBCL [469, 470, 473]. These studies strongly suggest that STAT1, ARTD9 and DTX3L might serve as novel druggable targets in HR-subtype DLBCL [469, 470, 473]. Moreover, a recent study using an Eμ-*c-Myc* driven B-cell lymphoma tumor mice model, demonstrated that the ARTD9-related ARTD8 (also known as B-aggressive lymphoma protein BAL2 or PARP14) is overexpressed in mouse B-lymphoma cells and can facilitate c-MYC driven B-lymphoid oncogenesis [474].

**Fig. 4:**
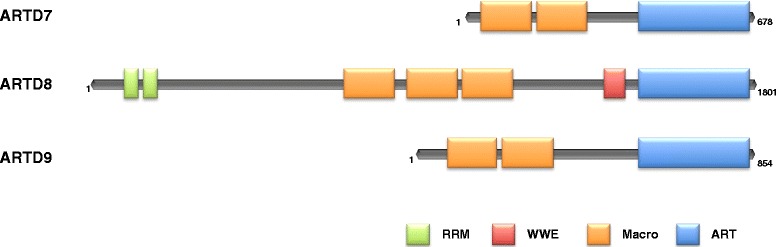
Schematic comparison of the domain architecture of the macrodomain-containing ARTD family members ARTD7, ARTD8 and ARTD9 Domain architecture of the human diptheria toxin-like macro-domain containing mono-ADP-ribosyltransferases and ARTD family members ARTD7, ARTD8 and ARTD9. The following domains are indicated: The diptheria toxin-like ART(D) domain is the catalytic core required for basal (mono-)ADP-ribosyltransferae (ART) activity. The WWE domain is named after three conserved residues (W-W-E) and is predicted to mediate specific protein-protein interactions in ubiquitin- and ADP-ribose conjugation systems [555, 636, 637]. The macro or A1pp domains are structurally related to the catalytic domain of enzymes that process ADP-ribose-1-phosphate, a reaction product derived from ADP-ribose 1-2′cyclic phosphate generated by TpT [638]. The macrodomain can serve as ADP-ribose or O-acetyl-ADP-ribose binding module [638]. RRM is an RNA and poly-ADP-ribose-binding/recognition motif [639]

Both ARTD9 and the closely related ARTD8 belong to the intracellular Diphteria toxin-like glutamate/aspartate-specific mono- and polymerizing-ADP-ribosyltransferase (ARTD) superfamily (also known as PARPs) [475–477] that also includes the ARTD9-related B-aggressive lymphoma protein and active mono-ADP-ribosyltransferase ARTD7 (also known as BAL3 or PARP15) [475, 476, 478–480], ARTD7, ARTD8 and ARTD9 contain several evolutionary conserved macrodomains [475, 476, 478–480], which have been recently shown to act as binding modules for free and protein-linked mono- or poly-ADP-ribose [475, 481–483]. A schematic comparison of the domain architecture of the macrodomain containing ARTD (PARP) family members ARTD7-9 is presented in Fig. 4. Both, ARTD8 and ARTD9 have been shown to interact with ARTD1 [471].

ARTD1, also known as PARP1, is the founding member of the ARTD/PARP family and has been suggested to act as a crucial oncogenic factor in various solid cancer types [484–488]. Numerous preclinical and clinical studies provided preliminary evidence that potent ARTD/PARP inhibitors with a high specificity towards ARTD1 and ARTD2, such as olaparib and veliparib (ABT-888) are promising drugs in the treatment of various solid cancer types [484, 485]. ARTD1/2-specific inhibitors are not only effective as single-agents to selectively kill DNA repair deficient solid cancer tumors (i.e., BRCAness tumors) but also as radio- and chemosensitizers of DNA repair proficient solid cancer tumors [484, 485]. Recent preclinical studies provided preliminary evidence that the ARTD1/2 inhibitors may also target DNA DSB repair deficient B- and T-lymphoid cells [489]. Several clinical trial phase I/II studies investigating the roles of ARDT1 and ARTD1/2 inhibitors in DLBCL are currently ongoing (Additional file 1: Table S9). For a detailed description of the oncogenic functions of ARTD1 and ARTD2, the readers are referred to the recent excellent reviews [488, 490, 491].

### Inhibition of STAT1 in the HR-subtype of GCB-, ABC- and type-3-DLBCL

STAT1 signaling is mainly activated by IFNγ and mediated through activation of IFNγ receptor and receptor-associated kinases JAK1 and JAK2 [492]. However, STAT1 can also be activated by other stimuli such as IL6 and IL10, which play crucial roles in tumor survival and progression [493–496].

Ligand-mediated activation of JAK1, JAK2 or tyrosine kinase 2 (Tyk2) lead to tyrosine phosphorylation of STAT1 on Y701, homodimerization and translocation of STAT1 to the nucleus [497–501]. JAK1/2-mediated phosphorylation of STAT1 phosphorylation on Y701 enhances the nuclear shuttling by triggering the nuclear retention of the shuttling STAT1α and STAT1β, which are kept in the nucleus until tyrosine dephosphorylation occurs [498, 501, 502]. STAT1 exists in two mayor isoforms, the full-length isoform STAT1α, which mainly acts as a sequence specific transcriptional activator of gene expression and STAT1β, lacking a complete transactivation domain [503]. STAT1β can act both as a transcriptional activator and repressor or antagonist of STAT1α, depending on the stimuli and cell type [504–508]. Subsequent nuclear phosphorylation on S727 in the transactivation domain of STAT1α can also occur independent of STAT1 tyrosine phosphorylation [509]. For instance, BCR signaling enhances S727 phosphorylation through calcium mobilization and activation of multiple serine kinase pathways, resulting in increased transcriptional activation of STAT1 target genes [510].

STAT1 has been initially considered to act exclusively as tumor suppressor and key regulator of the surveillance of developing tumors, primarily by activating growth-inhibitory and pro-apoptotic signaling in tumor cells, mainly mediated by IRF1 [511–514]. However, recent studies identified STAT1 as a proto-oncogene product in a variety of cancers [468, 470, 471, 515–518]. The oncogenic activity strongly correlated with abnormal expression of STAT1 and pSTAT1(pY701 and/or pS727) in these tumors [468, 470, 471, 515–518]. Constitutively active STAT1 promotes cell survival, invasion, metastasis, chemotherapy and/or radiation resistance in multiple types of solid cancers [468, 470, 471, 515–523]. Several studies have demonstrated that chemotherapeutic agents, such as doxorubicin or other anthracyclines enhance the expression of STAT1, its activation and the nuclear localization of phosphorylated STAT1 in chemotherapy-resistant solid cancer cells [516–518, 520, 524]. STAT1 has therefore been suggested as a potential target for radio- and chemosensitization of aggressive solid tumors that constitutively overexpress IFNγ/STAT1-dependent pathways [518]. Previous reports provided first evidence that a subset of STAT1- dependent antiviral gene products can simultaneously have radio- and chemo-protective pro-survival functions and are associated with oncogenesis in solid cancers [518].

Recent studies indicate that STAT1 can also act as a proto-oncogene product in hematopoietic cells and accelerate the development of T- and B-lymphoid malignancies [468, 470, 525]. Constitutive activation of STAT1 has been demonstrated in both acute and chronic leukemia [468, 470, 525]. For instance STAT1 promotes B-lymphoid leukemia development, at least in part by maintaining high MHC class I expression independently of IFN signaling [525]. Upregulation of MHC class I molecules has been suggested to represent a general mechanism to escape tumor surveillance [525]. A subsequent study identified an oncogenic TYK2-STAT1 pathway in T-cell acute lymphoblastic leukemia (T-ALL) cell lines, mediated through gain-of-function TYK2 mutations or activation of IL10 receptor signaling, that leads to the upregulation of BCL2 and T-ALL cell survival [468]. IFNγ/STAT1 signaling has therefore been suggested to exhibit both pro- and antitumor properties, depending on the context and cancer type [497]. Initially, IFNγ/STAT1 signaling helps protecting the host from tumor formation and development (immune surveillance), but subsequently IFNγ can also promote the tumors to resist attack (immunoediting) and escape by Darwinian evolution [473, 497, 526].

Our own recent study implies that STAT1 might also acts as an oncogene in DLBCLs and be required for proliferation, survival and chemoresistance of HR-subtypes of GCB- and ABC-DLBCL [470]. We observed increased constitutive phosphorylation of STAT1 on Y701 and S727 in the HR-like GCB-DLBCL cell line SUDHL7 and to a minor extend in the pSTAT3(Y705) positive BCR-subtype ABC-DLBCL cell lines OCI-Ly3 and OCI-Ly10 [470]. SUDHL7 has been suggested to represent a *bona fide* model cell line for HR-subtype GCB-DLBCL [470]. Analysis of primary DLBCL samples revealed positive nuclear co-staining for the oncogene product IRF2, STAT1-pY701 and STAT1-pS727 in 18 % of the primary DLBCL tumor samples analyzed, while benign tissue did not show this pattern in any case [470]. Our siRNA-*STAT1*-knockdown studies revealed that STAT1 is required for growth, survival and chemoresistance of HR-subtype GCB-DLBCL cells *in vitro*. STAT1β together with ARTD9 synergistically down regulated the transcriptional expression of tumor suppressor IRF1, while STAT1α together with ARTD9 up-regulated the expression of the proto-oncogene IRF2 [470]. IRF2, an antagonist of IRF1 is known to act as an oncogene in various types of cancer [527]. Recent studies showed that overexpression of STAT1β, the antagonistic isoform of STAT1α increases the growth rate of cells and their resistance to drug-induced apoptosis and cell cycle arrest by repressing STAT1α target genes such as p21 and IRF1 in human B cells [505]. Our study provides first evidence that STAT1β, together with ARTD9 may negatively regulate a tumor suppressor network (see also next section). Moreover, in line with the observed activity in B-cell leukemia STAT1 might also promote the outgrowth of DLBCL tumors with reduced immunogenicity (immunoediting). Future knockdown or ectopic (co)-overexpression xenograft studies need to be carried out in order to determine whether absence or overexpression of STAT1α and/or STAT1β negatively or positively affects chemoresistance and tumor growth of HR-DLBCL *in vivo*.

**Fig. 5:**
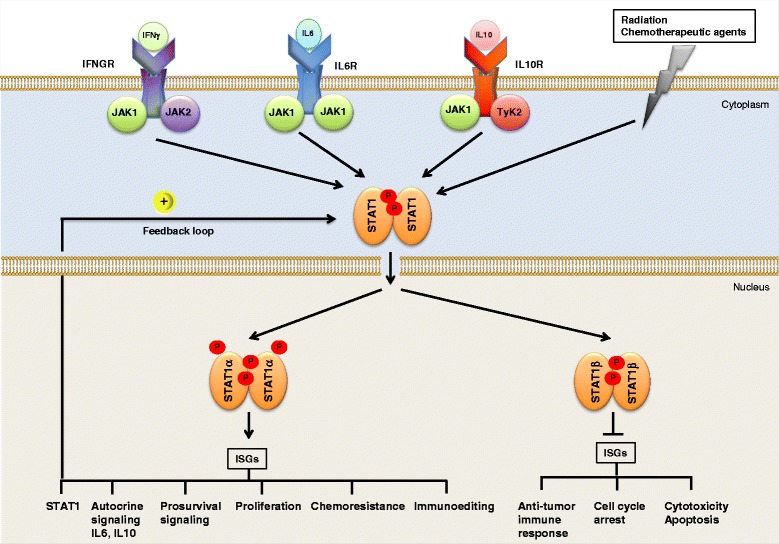
Proposed model for the postulated functions of STAT1 in HR-DLBCL-NOS. Chronic exposure to IFNs and chemotherapeutic agents such as alkylating agents, topoisomerase inhibitors, HDAC inhibitors, or proteasome inhibitors, can lead to constitutive oncogenic upregulation of STAT1 and interferon signaling genes (ISGs) during tumor development [515–518, 524, 640–643] and select tumor clones resistant to a cytotoxic microenvironment and concurrently to genotoxic therapy [518]. The overall population of tumor cells is sensitive to cytotoxic factors of microenvironment and committed to death because of the secretion of cytotoxic ligands by host cells (T lymphocytes, B lymphocytes, tumor-associated macrophages, dendritic cells etc.) [518]. The majority of tumor cells are relatively sensitive to radio- and/or chemotherapy. However, a small fraction of tumor cells (HR-DLBCL cells) constitutively express STAT1/ISGs and are resistant to the cytotoxic microenvironment and genotoxic therapy. Oncogenic constitutive expression of STAT1 can activate the prosurvival secretome, including the interleukines IL6 and IL10 [322, 468, 644] and in turn, can enhance selective advantages of surrounding tumor cells [518]. Moreover, STAT1 pathway can be also activated in the context of IL6 and IL10 [322, 645], thereby generating auto-stimulatory feedback loops (IL6-IL16R-STAT1 and IL10-IL10R-STAT1). Thus, IFN/JAK pathway and chemotherapeutic agents may act in concert with other HR-DLBCL specific intrinsic signaling pathways such as IL10/IL10R-JAK1-TYK2 and or IL6/IL6R-gp130-JAK1/2-TYK2 to induce chemotherapy resistance. In addition, oncogenic JAK/STAT1α-autocrine signaling in HR-DLBCL causes enhanced expression of STAT1α dependent proto-oncogenes (i.e., DTX3L ARTD9, IRF2, MCL1, BCL2, BCL6), thereby stimulating proliferation and survival pathways [470]. In addition, oncogenic STAT1α signaling helps the tumors to escape the attack by the immune system (immunoediting) [473, 497, 526]. Conversely, STAT1β repress the transcriptional activation of anti-proliferative and pro-apoptotic genes (including tumor the suppressors IRF1 and TP53) [470, 471] as well as genes involved in immune surveillance [473, 497, 526]. Adapted from REF: [470, 471, 518]. Abbreviations: HR-DLBCL host response DLBCL, ARTD ADP-ribosyltransferase Diphteria toxin-like, STAT signal transducer and activator of transcription, ISG interferon stimulated gene, IL interleukin, JAK Janus kinase, TYK2 tyrosine kinase 2, IRF interferon response factor, BCL6 B cell lymphoma protein 6, BCL2 B cell lymphoma protein 2

Together, STAT1 may serve as a novel potential drug target for treatment of high-risk relapsed and/or refractory DLBCLs with a brisk, but ineffective, host immune/inflammatory response and IFNγ signature. It remains to be investigated whether constitutive overexpression and/or phosphorylation of STAT1 alone, independent of ARTD9 or DTX3L, may also serve as novel useful prognostic biomarker for poor survival of fatal high-risk relapsed/refractory DLBCL. A model for the postulated functions of STAT1 in DLBCLs is shown in Fig. 5. Several clinical phase I/II studies, evaluating the safety and efficacy of JAK1 and JAK2 inhibitors in patients with relapsed/refractory B-cell lymphoma, including DLBCL are ongoing (NCT01905813, NCT01431209) (Additional file 1: Table S6) or in planning [109, 317, 318].

### Inhibition of ARTD9 in HR- and BCR-subtype of GCB-, ABC- and type-3-DLBCL-NOS

The inactive mono-ADP-ribosyltransferase ARTD9 is a nucleocytoplasmic shuttling protein that has been identified as a potential risk-related gene product in fatal high-risk DLBCLs [469, 472]. ARTD9 is constitutively overexpressed in high-risk chemoresistant subsets of DLBCL tumors associated with a brisk, but ineffective IFNγ-mediated host inflammatory response [469, 472, 473]. The large majority of ARTD9-positive HR-subtypes were classified as ABC-DLBCL [469, 472]. Recent studies demonstrated that overexpression of ARTD9 is mainly mediated by (IFNγ)-JAK1/STAT1 and/or autocrine (IL6)-JAK1/STAT3 signaling [469–471, 528]. In line with these observations and with the expression pattern in DLBCL tumors *in vivo*, ARTD9 is mainly overexpressed in pSTAT1(Y701/S727)-positive HR-subtype GCB-DLBCL cells and pSTAT3(Y705/S727)-positive BCR-subtype ABC-DLBCL cells weekly expressing pSTAT1(Y701/S727) [470]. Thus, overexpression of ARTD9 in high-risk tumors might also be modulated by differences in tumor microenvironment or relevant signaling pathways. Interestingly, overexpression of ARTD9 could not be observed so far in other pSTAT1(Y701/S727)-positive or pSTAT3(Y705/S727)-positive subgroups of DLBCLs such as T/HRLBCL or PMLBCL, which molecular profiles resemble (at least in part) those of HR-subtype DLBCL tumors [46, 53–55, 116].

ARTD9 has been proposed to be associated with lymphocyte migration and may promote the dissemination of malignant B cells in high-risk DLBCL *in vivo* [469]. Moreover, doxocyclin-induced overexpression of ARTD9 in an ARTD9-negative low-risk GCB-DLBCL cell line led to the induction of a small subset of IFN(γ)-related genes [469], thus provided preliminary evidence that ARTD9 might play a direct role in IFN signaling pathways [469]. Indeed, our own subsequent study identified ARTD9 as a novel oncogenic survival factor, which acts as a crucial negative and positive co-regulator of IFNγ/STAT1 signaling and mediates proliferation, survival and chemoresistance in HR-subtype DLBCL cells [470]. Inactivation of ARTD9 through siRNA- mediated depletion of ARTD9 not only inhibited cell proliferation and survival but also reversed chemoresistance in high-risk HR-subtype DLBCL cells [470]. Our study showed that ARTD9 can inhibit the IFNγ/STAT1-dependent anti-proliferative and pro-apoptotic activities of tumor suppressor IRF1 while simultaneously activating the STAT1/IRF2-mediated anti-apoptotic-pro-survival pathways in HR-subtype DLBCL cells [470]. ARTD9 acts as a repressor of the transcriptional activation of IRF1 and TP53 expression and thereby counteracts the IFNγ-dependent anti-proliferative and pro-apoptotic IFNγ-STAT1-IRF1-TP53 axis and induces an oncogenic switch in STAT1 from a tumor suppressor to an oncogene [470]. This may explain why constitutive IFNγ-STAT1 signaling does not lead to apoptosis but rather to survival and chemoresistance in HR-subtype DLBCL cells. The observed ARTD9-mediated down-regulation of tumor-suppressors IRF1 and TP53 may also represent a novel molecular mechanism inactivating the TP53/IRF1 pro- apoptotic pathway in high-risk DLBCL expressing wild-type TP53 [470]. In addition, ARTD9 simultaneously up-regulates the expression of BCL2 and BCL6 and thereby activates additional anti-apoptotic-pro-survival pathways [470]. The observed concomitant overexpression of BCL2 and BCL6 in the ARTD9 positive HR-subtype DLBCL cells also indicates that ARTD9 blocks the BCL6-mediated repression of BCL2 [470], which is frequently disrupted in DLBCL [365]. Thus, our study provided first evidence that ARTD9 together with STAT1β may negatively regulate a tumor suppressor network, while ARTD9 may concomitantly positively regulate a STAT1- dependent and independent proto-oncogene network in HR-subtype DLBCL [470]. It will be important to confirm these *in vitro* observations *in vivo* through ectopic overexpression of ARTD9 in xenograft DLBCL tumors, alone or in combination with STAT1α and/or STAT1β, respectively.

ARTD9 physically interacts with both isoforms of STAT1, STAT1α and STAT1β through its macrodomains in an ADP-ribosylation dependent manner [470, 471]. Co-immunoprecipitation of endogenous BAL1 and STAT1 in presence of increasing concentration of ADP-ribose and subsequent immunoblot analysis for BAL1 and STAT1 revealed that free ADP-ribose could disrupt the interaction between ARTD9 and STAT1 in a dose dependent manner [470, 471]. Thus, the observed interaction of ARTD9 with STAT1α or STAT1β, respectively, might be modulated *in vivo* through the levels of mono-ADP-ribosylation on the corresponding STAT1 isoforms. Our ADP-ribosylation analyses revealed that both STAT1α and STAT1β isoforms could be mono-ADP-ribosylated *in vitro* by ARTD8 and ARTD10 (unpublished observations). However, we cannot exclude the possibility that the observed interaction might be indirect and mediated through a mono-ADP-ribosylated bridging factor. Thus, the exact molecular mechanisms underlying the observed interaction between STAT1 and ARTD9 as well as the exact functional roles of the proposed mono-ADP-ribosylation activity remain to be investigated. The recent development of distinct ARDT7-, ARTD8- and ARTD10-specific ARTD inhibitors may help us in future to elucidate the ARDT enzyme(s) required for the exact functional roles of the proposed mono-ADP-ribosylation activity in DLBCL [529, 530]. In this regard, it will also be important to investigate whether ectopic overexpression of ARTD9 wild-type or ADP-ribose-binding mutant forms of ARTD9 in xenograft DLBCL tumors may positively or negatively affect tumor growth and metastasis *in vivo*. ARTD9 also interacted with the IFNGR-receptor complex and thereby stimulated directly or indirectly the kinase activity of JAK1/2 and phosphorylation of STAT1 on Y701 [470, 471]. However, the exact molecular mechanisms underlying these observations remain to be investigated.

**Fig. 6:**
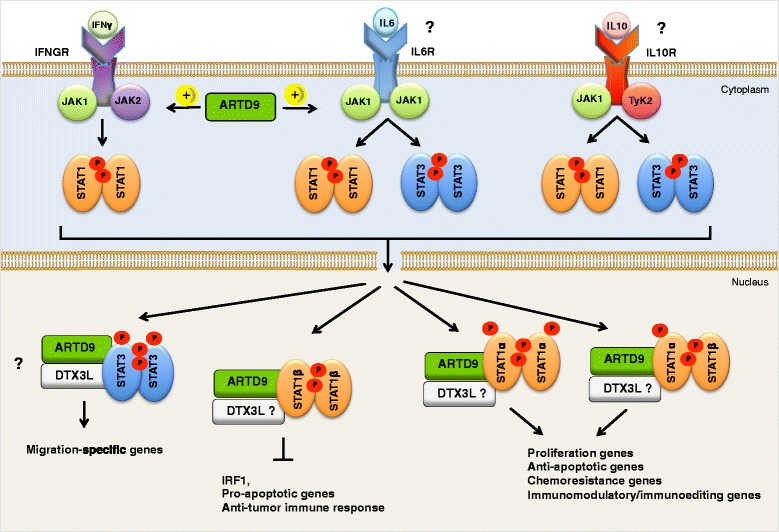
Proposed model for the postulated functions of ARTD9 and DTX3L in DLBCLs. ARTD9 and DTX3L act as novel oncogenic survival factors in relapsed or refractory DLBCL. Constitutively active IL6/IL6R-JAK1-STAT3, oncogenic IFN/IFNGR-JAK1/2-STAT1 and/or IL10/IL10R-JAK1-TYK2-autocrine signaling in HR-DLBCL causes overexpression of ARTD9 and DTX3L, which in turn, further stimulates the phosphorylation of STAT1 on Y701 and subsequently the enhanced expression of STAT1α dependent proto-oncogenes (i.e., ARTD8, ARTD9, DTX3L, IRF2, BCL6, BCL2). Both ARTD9 and DTX3L might also enhance the transcription of DNA damage repair/response genes upon treatment of tumors with genotoxic chemotherapeutic agents [548]. Conversely, ARTD9 and DTX3L repress together with STAT1β the transcriptional activation of tumor suppressor IRF1 and TP53 [470, 471]. Overexpression of ARTD9 inhibits intrinsic and extrinsic IFNγ-STAT1-IRF1/p53-mediated cell death pathways while simultaneously enhancing the STAT1-dependent IRF2-mediated proliferation and BCL6-BCL2 mediated survival pathways. As a consequence ARTD9 induces an oncogenic switch in STAT1 from a tumor suppressor to an oncogene and mediates proliferation, survival and chemo-resistance in HR-DLBCL. Pharmaceutical inhibition of ARTD9 in HR-DLBCL associated with constitutively active STAT1 signaling might therefore reactivate the anti-proliferative and pro-apoptotic IFNγ-STAT1-IRF1/p53 axes and reverses chemo-resistance in HR-DLBCL. In addition, endogenous overexpression of DTX3L and/or ARTD9 might also be associated with lymphocytes migration and may promote the dissemination of malignant B cells in high-risk DLBCL *in vivo*, potentially dependent on STAT3 or PI3K/AKT/mTORC1 signaling [471]. DTX3L and/or ARTD9 might form specific complexes with non-canonical STAT1:STAT3 heterodimers and activate a migration-specific signaling and gene expression program in HR-DLBCL. Finally, oncogenic. ARTD9/DTX3L-STAT1α signaling might upregulate immunomodulatory genes and may help the tumors to escape the attack by the immune system (immunoediting) [470, 471, 473]. Conversely, ARTD9 and DTX3L together with STAT1β might repress the transcriptional activation of genes involved in immune surveillance [470, 471, 473]. Adapted from REF: [470, 471]. Abbreviations: ARTD ADP-ribosyltransferase Diphteria toxin-like, DTX3L deltex (DTX)-3-like E3 ubiquitin ligase, STAT signal transducer and activator of transcription, ISG interferon stimulated gene, IL interleukin, JAK Janus kinase, TYK2 tyrosine kinase 2, IRF interferon-regulatory factor, BCL6 B cell lymphoma protein 6, BCL2 B cell lymphoma protein 2

ARTD9 may also be directly involved in editing or inhibiting the IFNγ-dependent host immune response against HR-subtype DLBCL either through its transcriptional co-repressor activity and termination of IFNγ-mediated gene expression and inhibition of the extrinsic IFNγ-induced anti-proliferative and pro-apoptotic STAT1-IRF1-TP53 axes or through its transcriptional co-activator activity and induction of expression of the NK/T-cell inhibitory molecules i.e., PD-1L and increasing the resistance of IFNγ-treated tumor cells towards NK cell- or lymphokine activated killer-mediated lysis [470]. IFNγ is typically secreted by activated, tumor-infiltrating cytotoxic T lymphocytes and NK and NK/T cells [531, 532]. A proposed model for the postulated functions of ARTD9 in DLBCL is presented in Fig. 6. Remarkably, our study strongly suggests that ARTD9 (may be together with DTX3L) acts both as a negative and positive co-factor in transcription in HR-DLBCL. The observed antagonistic transcriptional activities might be explained by the formation of different ARTD9-containing promoter-specific co-repressor and co-activator complexes. In analogy to other chromatin remodeling and transcriptional cofactors such as the ARTD members ARTD1 or ARTD8, respectively [533–536], and depending on the methylation status of the corresponding promoters and/or enhancers, ARTD9 might differentially interact either with STAT1α/β- or other unknown factor-X-containing transcriptional coactivator complexes (i.e., containing the co-activators CBP/EP300, NCOA and/or BRG1 [537–539]) or with STAT1β-homodimer- or other unknown factor-Y-containing corepressor complexes (i.e., containing the co-repressors MeCP, and NCORs [540, 541]). Promoters and/or enhancers of genes involved in survival, proliferation, chemoresistance, autocrine signaling as well as immunoediting are considered as being hypomethylated in HR-DLBCL under these conditions while the corresponding promoters and/or enhancers of pro-apoptotic and anti-proliferative genes are considered as being hypermethylated.

Future studies are required to decipher the composition of these distinct ARTD9-STAT1 or ARTD9-X-factor co-repressor and co-activator complexes.

Taken together, aberrant constitutive overexpression of ARTD9 may not only serve as a novel useful prognostic biomarker for poor survival of fatal high-risk relapsed/refractory DLBCL but also as a novel rational future drug target for treatment of high-risk relapsed/refractory HR- (and BCR-) subtypes of DLBCL-NOS. The observed macrodomain and mono-ADP-ribosylation-mediated interaction between ARTD9 and STAT1 not only indicates a regulatory cross-talk between ARTD9 and other active members of the ARTD family but also provide the rational base for the development of macrodomain-specific small molecules that can selectively disrupt ARTD9 complex formations in ARTD9-positive relapsed/refractory HR-subtype DLBCLs.

### Inhibition of DTX3L in HR- and BCR-subtype of GCB-, ABC- and type-3-DLBCL-NOS

**Fig. 7:**
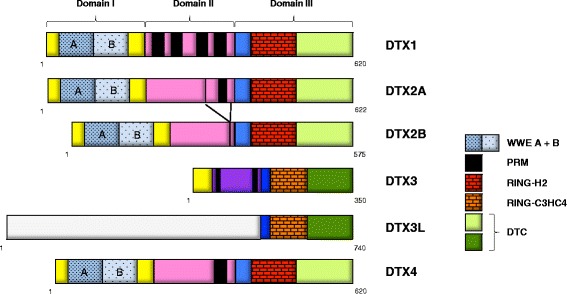
Schematic comparison of the domain architecture of the DTX family. Domain architecture of the human Deltex-like E3 ubiquitin ligases and DTX family members DTX1, DTX2A, DTX2B, DTX3, DTX3L and DTX4. The domain organization is based on analogous functional motifs in murine DTX family members DTX1, DTX2, DTX2deltaE and DTX3 [589], human DTX1 [544, 646], and human DTX3L [542, 543]. DTX1, DTX2 and DTX4 have well-conserved domains I, II and III. DTX1, DTX2A, DTX2B and DTX4 contain two conserved tandem WWE modules in their domain I [646]. The N-terminal domains I of DTX1, DTX2A and DTX2B bind through their tandem WWE modules to the ankyrin repeat region of intracellular NOTCH1 [646]. The WWE domain is named after three conserved residues (W-W-E)and is predicted to mediate specific protein-protein interactions in ubiquitin- and ADP-ribose conjugation systems [555, 636, 637]. DTX3 lacks most of the site corresponding to the binding site for the ankyrin repeats of human NOTCH1. Of interest, the WWE domain of DTX1 serves also as a *bona fide* poly-ADP-ribose binding module [555]. Domains II of DTX1, DTX2A, DTX3 and DTX4 contain a proline-rich region (black boxes) that is not found in DTX3L. DTX2B is an alternatively spliced form of DTX2A, DTX2B lack a proline-rich motif (PRM), the fourth of nine predicted DTX2 exons. The most conserved region of all human DTX family members (DTX1, DTX2A, DTX2B, DTX3, DTX3L and DTX4) is their C-terminal part, domain III. The C-terminal domain III of DTX1, DTX2A, DTX2B and DTX4 contain a classic RING finger E3 ubiquitin ligase domain (RING-H2 finger motif; red box); while domain III of DTX3 and DTX3L contain a RING-C3HC4 finger motif found in many E3 ubiquitin ligases (indicated by an orange box). Human DTX3 has a short unique N-terminal domain (yellow box), and DTX3L contains a longer unique N-terminal region (grey box). Abbreviations: RING really interesting new gene, DTX deltex, DTC Deltex C-terminal domain

The Deltex (DTX)-3-like E3 ubiquitin ligase DTX3L is an active E3 ubiquitin ligase with C-terminal identity to the Deltex (DTX) family member DTX3 [542, 543]. The human DTX family of E3 ubiquitin ligases encompasses six E3 ubiquitin ligases [542–546]. A schematic comparison of the domain architecture of the DTX family is shown in Fig. 7. DTX3L was originally identified as a binding partner of ARTD9 and as a novel risk-related gene product in high-risk chemotherapy-resistant aggressive forms of DLBCL [469, 542]. DTX3L is also overexpressed in HR- and BCR- subtype DLBCLs and at least in part associated with intrinsic IFNγ signaling and constitutive activity of STAT1 [469, 470]. Similar to ARTD9, no overexpression of DTX3L could be observed so far in T/HRLBCL or PMLBCL [46, 53–55, 116]. In line with the observation that the *DTX3L* and *ARTD9* genes are located in a head-to-head orientation on chromosome 3q21, sharing the same bidirectional STAT1/IRF1 and STAT3-responsive promoter [469–471], DTX3L is mainly overexpressed in pSTAT1(Y701/S727)-positive HR-subtype GCB-DLBCL cells and pSTAT3(Y705/S727)-positive BCR-subtype ABC-DLBCL cells weekly expressing pSTAT1(Y701/S727) [470].

Several recent studies provided evidence that DTX3L acts tightly together with ARTD9 in proliferation, chemoresistance and survival of solid tumor cells, albeit through different mechanisms [471, 547, 548]. For instance, we could recently demonstrate that DTX3L together with ARTD9 forms a complex with STAT1 and mediates together with ARTD8 and ARTD9 proliferation, chemoresistance and survival of CRPC-like metastatic prostate cancer (mPCa) cells [471]. DTX3L and ARTD9 act together as repressors of the tumor suppressor IRF1 in mPCa cells [471]. Remarkably, DTX3L together with STAT1 and STAT3 enhanced cell migration of mPCa cells in an ARTD9-independent manner [471] and has been suggested to function in a non-canonical STAT1:STAT3 heterodimer-mediated signaling pathway in migration of mPCa cells *in vitro* [471]. Thus, it is very likely that DTX3L act also together with ARTD9 in the same proliferation, cell survival and chemotherapy resistance pathways in DLBCL. In analogy to the suggested roles of ARTD9, DTX3L may also be directly involved in editing or inhibiting the IFNγ-dependent host immune response against HR-subtype DLBCL. It will therefore be important to investigate whether ectopic overexpression of DTX3L xenograft tumors (alone or simultaneously in combination with ARTD9 and/or STAT1) may positively or negatively affect chemoresistance, enhanced growth and/or metastasis of HR-DLBCL tumors *in vivo*.

Several DTX3L studies in solid cancer cell lines strongly indicate that DTX3L might also act in a STAT1/IFNγ independent manner [547, 548]. The first studies performed by Yan Q. et al. suggested that DTX3L is implicated in the DNA damage response pathway via monoubiquitinylation of histone H4K91 [547, 548]. Ectopically overexpressed GFP-tagged DTX3L and ARTD9 were recruited to sites of DNA damage in solid cancer cells [547, 548]. This activity was dependent on ARTD1/PARP1-mediated (n)ADP-ribosylation [548], see also next sections.

DTX3L together with ARTD9 protected solid cancer cells exposed to DNA-damaging agents and have been therefore suggested to limit the efficacy of chemotherapy-induced DNA damage responses [547, 548]. More importantly, a recent *in vivo* study performed by Thang N.D. et al. provided first evidence that DTX3L mediates invasion and metastasis in melanoma through stimulation of the FAK/PI3K/AKT signaling pathway [549]. Depletion of DTX3L in murine and human melanoma cells resulted in decreased activity of FAK/PI3K/AKT signaling and reduced invasion and metastasis of murine and human melanoma cells [549]. It is therefore important to investigate whether DTX3L may cooperate with the PI3K/AKT/mTORC1 or PI3K/AKT/NF-κB signaling pathways in DLBCL.

Besides forming homodimers, DTX3L can also form heterodimers with DTX1 via their respective N- termini in a manner that enhance the E3 ubiquitin ligase activity of DTX3L [542]. DTX1 is thought to act as a signal repressor or as a mediator of negative feedback for canonical NOTCH1 signaling pathways [550]. On the other hand, DTX1 can also positively regulate cell context-dependent non-canonical NOTCH1/2 signaling pathways and NOTCH1/2-independent signaling pathways in cancer [551, 552]. DTX1 has been shown to enhance the aggressiveness and survival of cancer cells through the activation of the RTK/PI3K/AKT and the MAPK/ERK mitotic pathways and induction of anti-apoptotic MCL1 [551]. DTX1 is frequently mutated in GCB- and ABC-DLBCL [21, 22]. A recent exome sequencing study found DTX1 mutated at high frequency (9–12 %) in a DLBCL cohort derived from Chinese patients [21]. The Non-silent (gain or loss of function) mutations in DTX1 have been postulated to impair its function as a negative regulator of Notch pathway [21]. Functionally relevant gain-of-function mutations of *NOTCH1* and *NOTCH2* were identified in up to 8 % of DLBCL patients and postulated to act as likely drivers of DLBCL pathogenesis [16, 553, 554]. *NOTCH2* mutations mainly occur in ABC- and type-3-DLBCL-NOS [16]. HR-subtype-DLBCL has been shown to overexpress NOTCH2 [116]. Future studies are required to investigate whether DTX3L might also act independently of ARTD9 together with DTX1 in non-canonical NOTCH1/2-Deltex1-dependent PI3K/AKT signaling pathways in HR subtype DLBCL. Of interest, the WWE domain of DTX1 (Fig. 7) serves also as a *bona fide* poly-ADP-ribose-binding module [555] and might therefore be also involved in the observed ARTD1 dependent recruitment of DTX3L to sites of DNA damage in solid cancer cells.

Taken together, DTX3L might serve as a novel useful prognostic biomarker for poor survival and as a rational drug target for treatment of high-risk relapsed/refractory HR- (and BCR-) subtypes of DLBCL-NOS.

### ARTD8, a novel drug target in HR-, BCR- and MYC-driven DLBCL subtypes?

We have recently shown that both DTX3L and ARTD9 can form nuclear complexes with the closely related macrodomain-containing and enzymatically active ARTD8 [471]. Although no functional or clinical studies providing evidence for a direct role of ARTD8 in DLBCL have yet been published, we recently showed that ARTD8 is overexpressed in HR-subtype GCB-DLBCL and BCR-subtype ABC-DLBCL cell lines [470]. We recently demonstrated that ARTD8 mediates in cooperation with DTX3L and ARTD9 survival and proliferation of high-risk metastatic castration resistant prostate cancer (CRPC) cells [471]. Our study also provided first evidence that the enzymatic activity of ARTD8 is required for survival of metastatic CRPC cells [471]. These observations imply that ARDT8 might also cooperate with DTX3L and ARTD9 in DLBCL.

Several recent studies by the Boothby lab demonstrated that ARTD8 interacts with STAT6 and amplifies STAT6-mediated gene expression pathways in B and T cells [479, 480]. ARTD8 has been suggested to functions as STAT6-specific coregulator of IL4-mediated gene expression and to enhance IL4-induced proliferation and protection of B cells against apoptosis following irradiation or growth factor withdrawal [479, 480, 556]. However, STAT6 may only play a significant role in a small subset of DLBCL-NOS. For instance, STAT6 has been suggested to be inactive in ABC-DLBCL cells due to the overexpression of protein tyrosine phosphatase 1B (PTP1B) in this subtype [557, 558]. Thus other DLBCL related factors might be a target of ARTD8. Indeed, it has been suggested that ARTD8 could influence c-MYC-induced B-lymphoid oncogenesis by increasing the cellular metabolic rates [474]. A recent study using an Eμ-*c-Myc* driven B-cell lymphoma mice model, demonstrated that ARTD8 is overexpressed in mouse B-cell lymphoma and can facilitate B-lymphoid oncogenesis and alterations in developmental progression driven by c-MYC [474]. Conversely, ARTD8-deficient mice showed a reduced susceptibility to c-MYC-induced B-cell lymphoma [474]. Interestingly, ARTD8 also enhance the expression of MCL1 or PIM1, which are both implicated in chemotherapy resistance in DLBCL [556].

A recent study showed that ARTD8 was overexpressed in over 80 % of primary multiple myeloma (MM) samples tested in this study [559]. More importantly, this study demonstrated that ARTD8 promotes c-Jun N-terminal kinase (JNK)-2-dependent survival in MM *in vitro* by binding and inhibiting the kinase activity of JNK1 [559]. Depletion of ARTD8, or inhibition of the catalytic activities of ARTD8 enhanced the sensitization of MM cells to anti-myeloma agents [559]. Preclinical studies in DLBCL and MM indicate that obatoclax/bortezomib- or obatoclax/carfilzomib- as well as ricolinostat/carfilzomib mediated lethality in DLBCL cells *in vitro* and *in vivo* includes activation of the stress-related JNK1 signaling pathway [371, 560–562]. It is therefore important to test whether ARTD8 might also function as a druggable oncogenic factor in relapsed or refractory c-MYC-driven double-hit or triple-hit DLBCL, HR- and/or BCR-subtype DLBCLs.

### Are the proposed ARTD9-DTX3L-dependent pathways involved in chemotherapy-resistance in DLBCL connected to ARTD1?

Recent studies provided first evidence that ARTD9 and DTX3L might act in a (poly or) mono-ADP-ribosylation-dependent DNA damage response pathway and together with ARTD1 enhance tumor cell survival [547, 548]. As already mentioned above, both DTX3L and ARTD9 were recruited to sites of DNA damage in solid cancer cells [547, 548]. Moreover, both ARTD9-related macrodomain-containing ARTD family members and ARTD7 and ARTD8 were also localized to sites of DNA damage in a (poly-)ADP-ribosylation and macrodomain-dependent manner [548, 563]. Both ARTD8 and ARTD9 have been suggested to act as novel modulators of homologous recombination (HR) required for mitigating replication stress and promoting and genomic stability [548, 563], albeit their exact functions in this process remain to be defined [548, 563]. These studies indicate that combined inhibition of the enzymatic activities of ARTD1 and ARTD8 inhibitors might potentiate genotoxic chemotherapy by blocking HR-DNA repair and rendering solid cancer cells hypersensitive to DNA damage. We have recently demonstrated that DTX3L forms complexes with ARTD8 and ARTD9 independent of mono-ADP-ribosylation, where as ARTD9 interacts with ARTD8 and other ARTD family members, such as ARTD1 in a (mono-)ADP-ribosylation dependent manner [471]. Thus ARTD1 might indeed function together with DTX3L and macro-domain-containing macroARTD7-9 in chemotherapy resistance in solid cancer.

However, it remains to be further investigated whether this proposed ARTD1 connected macroARTD(7–9)-DTX3L pathways and mechanisms do also exists in DNA-DSB repair proficient DLBCL, or may be restricted to DNA-DSB repair deficient solid cancer types and certain DNA-DSB repair deficient B and T lymphoid tumor types. Indeed, a recent study provided preliminary evidence that the ARTD1/2 inhibitor olaparib selectively targets ATM mutant (DNA-DSB repair deficient) but not wild-type (DNA-DSB repair proficient) B-lymphoid cells, including proliferating primary CLL cells *in vitro* and *in vivo* [489]. Moreover, one has to be also very cautious because several recent studies provided preliminary evidence that ARTD1 might not act as an oncogenic factor but rather as a tumor suppressor in DNA DSB repair proficient DLBCL and other B-cell lymphoma [23, 564, 565]. For instance, a recent study demonstrated that endogenously overexpressed BCL2 localizes in the nucleus in B-lymphoid tumors and suppresses ARTD1 function and non-apoptotic ARTD1 dependent necrotic cell death in ATM wild-type DLBCL cells [564]. BCL2 directly interacts with ARTD1 and blocks the enzymatic activity of ARDT1 in CLL and DLBCL cells [564]. Remarkably, ABT-737 can disrupt the BCL2-ARTD1 interaction and promotes ARTD1 and poly-ADP-ribosylation dependent necrotic cell death in primary ATM wild-type CLL cells [564]. Thus, ARTD1 might therefore act as a tumor suppressor in absence of BCL2 in ATM wild-type DLBCL. Along this line, ARTD1 activation has been recently shown to be associated with NOTCH1 ligand-mediated apoptosis in B-cell acute lymphoblastic leukemia (B-ALL) where NOTCH1 activation leads to growth arrest and cell death [566]. The NOTCH1 target Hairy/Enhancer of Split1 (HES1) interacts with ARTD1, activates the enzymatic activity of ARTD1, which then in turn results in ARTD1 overactivation, NAD depletion and necrotic cell death [566]. Another cautionary study provided preliminary evidence that ARTD1 acts as a transcriptional co-repressor of the *BCL6* gene in DLBCL [565]. ARTD1 binds to the enhancer/promoter region of the *BCL6* gene and inhibits the transcriptional activation of BCL6 in a poly-ADP-ribosylation-dependent manner [565]. In line with these observations, it has been recently shown that a subset of primary DLBCL samples presents loss-of-function mutations in ARDT1 and/or lower expression levels of ARDT1 [23]. Targeting and inactivation of ARTRD1 by some DLBCL tumors has been suggested to represent a survival adaptation strategy to high degrees of genomic instability in B-cell lymphoma [23]. These observations strongly indicate that the proposed cancer promoting ARTD1-DTX3L/ARTD9 connection might only exist in solid cancers and support the idea that ARTD1 acts as an ARTD9 and DTX3L independent tumor suppressor in DLBCL. Interestingly, it has been recently shown that macrodomain-containing macroH2A1.1 can inhibit the enzymatic activity of ARTD1 [567, 568]. Thus one cannot exclude the possibility that the macrodomain-containing ARTD9, ARTD8 or ARTD7 may indeed functionally interact with ARTD1 in DLBCL and even block the enzymatic and potential tumor suppressor activity of ARTD1 through their macrodomains.

Several preclinical and clinical trial phase I/II studies, which include B-cell lymphoma patients, are currently ongoing to test the efficacy of ARTD/PARP inhibitors as therapeutic agents 7 (NCT00810966, NCT01326702, NCT00740805) [488, 569, 570] (Additional file 1: Table S9). Thus the ongoing preclinical and clinical trial phase I/II studies and future phase II studies investigating the role of ARDT1/2 specific inhibitors in ARTD9 or BCL2-positive and -negative DLBCL-NOS subtypes will certainly clarify whether the proposed functional crosstalk of ARTD1 and ARTD7-9 may also exist in DLBCL.

### Status quo of drugs targeting the STAT1, ARTD8, ARTD9 and DTX3L-mediated pathways

Although the combination of classical therapeutic drugs with highly ARTD8 or DTX3L-specific inhibitors and drugs specifically targeting STAT1 or the macrodomains of ARTD9 might provide a novel therapeutic strategy to increase the sensitivity of relapsed/refractory DLBCL tumors towards classical therapy, the specificity and toxicity of novel agents selectively targeting STAT1, DTX3l, ARTD9 or ARTD8 have to be carefully analyzed to avoid toxic off target effects in the surrounding benign cells/tissues and counter activity of drug-target related proteins (i.e., antagonistic activities of ARTD or DTX family members). Highly selective and potent inhibitors specifically targeting STAT1, DTX3L or ARTD9, respectively are not yet available.

### Selective STAT1 inhibitors

Selective STAT1 inhibitors are not yet available due to the observed STAT cross-binding specificity of SH2-domain-based competitive small inhibitors of STAT1 and STAT3 [571]. STAT1/3 selective SH2-domain-based competitive small inhibitors act through the prevention of the recruitment of STAT1 or STAT3 to the receptor complex and inhibition of their homo- (or hetero-) dimerization, which is critical for nuclear translocation and DNA binding [572, 573]. Thus, the SH2-domain-based competitive small inhibitors of STAT3 might therefore not only target the pSTAT3 positive HR-subtype ABC-DLBCL but also pSTAT3 negative HR-subtype GCB-DLBCL. In addition, inhibition of JAK2 does not only block the activation of STAT3 but also the activity of STAT1 in B-cell lymphoma [60]. Despite clear anti-cancer efficacy *in vitro* and *in vivo* [574–577], SH2-domain-based competitive small inhibitors of STAT3 have not yet reached the clinic.

JAK1/JAK2 inhibitors, such as fedratinib (TG101348) [60, 317], which strongly reduce the phosphorylation of both STAT1 and STAT3 [60], have been shown to inhibit ABC-DLBCL growth *in vitro* and *in vivo* [313, 316]. JAK1/JAK2 inhibitors, such as INCB039110 [318] or ruxolitinib (INCB18424), are currently in clinical phase I and II trials in B-cell lymphoma, including relapsed/refractory DLBCL (NCT01905813, NCT01431209), or have been suggested for further clinical evaluation in DLBCL (pacritinib, SB1518), including relapsed or refractory DLBCL [109, 317, 318] (Additional file 1: Table S6).

Drugs targeting the activity of JAK1/2 mainly block the phosphorylation of STAT1 on Y701 but do only affect the phosphorylation of STAT1 on S727 to a minor extend. Moreover, the activity of uSTAT1, an unphosphorylated form of STAT1 also involved in oncogenesis [578, 579], is not inhibited by JAK1/2 inhibitors. In contrast, SH2-domain-based competitive small inhibitors selectively targeting STAT1 will inhibit all isoforms and phosphorylated forms of STAT1.

However partial inhibition of STAT1 or selective inhibition of STAT1:STAT3 signaling pathways could be more beneficial than complete inhibition of STAT1. A recent mice study provided evidence that complete inhibition of STAT1 might affect B-lymphoid development before differentiation to pre-B cells, and particularly blocks the recovery phase from doxorubicin-(chemotherapy) induced hematopoietic toxicity [580]. STAT1 is required for the efficient B lymphocyte repopulation in the recovery phase in mice and thus particularly the B cell immune function might be disturbed or delayed after hematopoietic reconstitution in a situation of chemotherapy, combined with complete STAT1 inhibition [580]. On the other hand it is not yet clear whether all STAT1 isoforms (STAT1α and STAT1β) or phosphorylated forms (uSTAT1, pSTAT1(Y701), pSTAT1(S727) or pSTAT1(Y701/S727)) are involved in these process. Thus, small molecule inhibitors that only partially inhibit STAT1, such as of JAK1/JAK2 inhibitors, or indirectly inhibit STAT1 by targeting a subset of STAT1-dependent oncogenic signaling pathways, such as small molecule inhibitors of ARTD9 and DTX3L, might be the more suitable drugs for targeting the oncogenic functions of STAT1 in DLBCL.

Remarkably, recent studies provided evidence that pan-HDACi may not only inhibit the phosphorylation of STAT3 (on Y705) but also the phosphorylation of STAT1 (on Y701). For instance, HDAC4 positively regulates STAT1 activation and mediates STAT1-dependent platinum resistance in ovarian cancer [522]. HDAC4 deacetylates STAT1, thereby enhancing phosphorylation and nuclear translocation of STAT1 upon cisplatin-treatment in platinum resistant ovarian cancer cells (exposure to chemotherapy) [522]. Conversely silencing of HDAC4 increased acetyl-STAT1 levels and dephosphorylation of STAT1, thereby prevented platinum induced STAT1 activation and restored cisplatin sensitivity [522].

### Inhibitors targeting the macro-domains of ARTD8 and ARTD9

Since ARTD9 is an inactive enzyme ARTD inhibitors targeting the enzymatic activity of ARTD family members cannot be applied for blocking the function of ARTD9. On the other hand the selective mono-ADP-ribose-specific ADP-ribose-binding activity of the macro-domains of ARTD7, ARTD8 and ARTD9 [482] presents rational drug targets for the selective inhibition of the oncogenic functions of macro-domain-containing ARTDs. A recent study provided first evidence that the ARTD macrodomains have highly selective binding activities [482], which makes them even more attractive as druggable targets. The high-resolution crystal structure of the macro-domains of ARTD7 and 8 have already been recently published [481, 482, 581] or deposited in the protein data bank PDBe, allowing now the design of small molecule inhibitors selectively targeting each single macro-domain of ARTD7: http://www.ebi.ac.uk/pdbe/entry/pdb/3v2b, ARTD8: http://www.ebi.ac.uk/pdbe/entry/pdb/3q6z, http://www.ebi.ac.uk/pdbe/entry/pdb/3q71, http://www.ebi.ac.uk/pdbe/entry/pdb/4d86, http://www.ebi.ac.uk/pdbe/entry/pdb/3vfq, http://www.ebi.ac.uk/pdbe/entry/pdb/4abl, http://www.ebi.ac.uk/pdbe/entry/pdb/4abk, ARTD9: http://www.ebi.ac.uk/pdbe/entry/pdb/5ail.

### ARTD inhibitors selectively targeting the enzymatic activities of ARTD8

Although developed as selective ARTD1/PARP1 inhibitors, family-wide chemical profiling and structural analysis of ARTD/PARP inhibitors demonstrated that many of the best-known inhibitor compounds, which are widely used as so called ARTD1/2-specific research tools as well as in experimental clinical studies, including olaparib, veliparib (ABT-888) and rucaparib, lack clear selectivity towards ARTD1 and ARTD2 and have additional low promiscuous inhibitory activity across the ARTD family, thus exhibiting polypharmacology [582]. Moreover, recent studies found diverse ARTD-independent mechanisms of action associated with the ARTD1/2-specific clinically relevant inhibitors such as rucaparib, veliparib, and olaparib [583–585]. They have both “beneficial” and detrimental off target effects: For instance rucaparib and to a lesser extend veliparib inhibit PIM1/2 and CDK9 [584], which are important kinases and signaling factors regulating cell survival and can mediate chemotherapy resistance to available agents, such as rapamycin or BCL2 inhibitors [379, 586, 587]. In addition rucaparib has the capacity to inhibit the phosphorylation of STAT3 most-likely through its inhibition of the STAT3 phosphorylating kinases DYRK1A and/or CDK1 [584]. Moreover, treatment of BRCA1-deficient cancer cells with olaparib leads to the induction of an IFN-related gene expression signature and activation of IFN-dependent pro-apoptotic signaling pathways in solid cancer cells [588].

Thus, the development of 4^th^ and 5^th^ generation of ARTD inhibitors selectively targeting single ARTD family members without having off target activity is of extraordinary importance for the therapeutic use of ARTD inhibitors. The high-resolution crystal structure of the catalytic domains of ARTD7 and 8 have been recently published [529, 582] or deposited in the protein data bank PDBe, allowing now the design of small molecule inhibitors selectively targeting the enzymatic activity of ARTD8: http://www.ebi.ac.uk/pdbe/entry/pdb/4f1q, http://www.ebi.ac.uk/pdbe/entry/pdb/4f1l, http://www.ebi.ac.uk/pdbe/entry/pdb/4py4. Indeed, using a virtual screening approach, including structural modeling, a set of more than 16 small molecule inhibitors has been recently discovered that bind to ARTD7, ARTD8 and/or ARTD10 [529, 530]. The molecules belonged to eight different structural classes, and two of the molecules with good water solubility displayed a promising selectivity for ARTD8 in the low micromolar range [529].

### Inhibitors targeting the DTX-like E3 ubiquitin ligase activity of DTX3L

Drugs selectively inhibiting the E3 ubiquitin ligase activity of the Deltex family members are not yet available. Unfortunately, there are no high-resolution crystal structures of the C-terminal catalytic RING E3 ligase domain of DTX family members published so far. The only reported high-resolution structure of the conserved C-terminal region of DTX proteins is the so called Deltex C-terminal (DTC) domain of human DTX3L [543]. DTC is of unknown function but has been suggested to be involved in the catalytic process or required for the substrate specificity of DTX family members [542, 543, 589]. The design of small molecule inhibitors selectively targeting the E3 ubiquitin ligase of DTX3L remains therefore dependent on the published RING E3 structures of other E3 ligases. RING E3 ligases do not possess intrinsic catalytic activity and act by simultaneously binding to the charged E2, ubiquitin and the protein substrate [279, 590]. Thus the most advanced ligase-based drug discovery strategy to date for targeting RING E3 ligases has been the development of allosteric antagonists or protein-protein inhibitors of E3-substrate binding, that is considered more difficult to target than a catalytic site [279, 590]. In contrast to the ARTD inhibitors, the selectivity might not be a crucial prerequisite for drugs blocking DTX3 functions.

Non-selective drugs simultaneously targeting DTX3 and DTX1 might be even more efficient and beneficial for DLBCL patients since DTX1 may also have oncogenic functions in DLBCL and forms distinct heterodimers with DTX3L.

### Multi-targeted mechanism-based combinatorial experimental therapies for relapsed/refractory DLBCLs

**Fig. 8:**
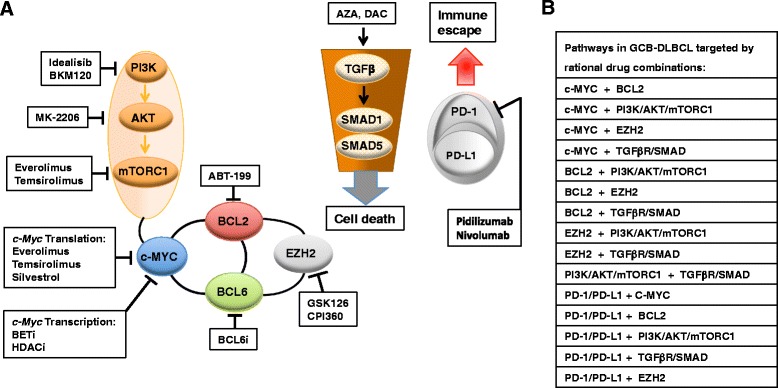
Mechanism-based combinatorial experimental regimens aimed at disrupting known oncogenic cooperation pathways in GCB-DLBCLs. Targets for rational mechanism-based combinatorial experimental regimens are shown for GCB-DLBCL (**a** and **b**). The black thick lines connecting the different drug targets pathways depict established biological interactions and/or dependencies. The single drugs listed in (**a**) have either proven clinical activity in patients with DLBCL or are currently in clinical trials for patients with B-cell lymphoma, including DLBCL. Pathways in GCB-DLBCL targeted by rational drug combinations using drugs tested as single agent in preclinical studies or clinical trials are shown in (**b**). Silvestrol inhibits RNA helicase and translation initiationfactor eIF4A thereby blocking translation (i.e., of oncogenic factors) [647]. Azacytidine (AZA) and decitabine/5-aza-2′-deoxycytidine (DAC/5-AZA) inhibit DNA methyltransferases (mainly DMNT1 and DMNT3) resulting in the re-expression of tumor-suppressor genes [419, 420]. Adapted from REF: [601, 602]. Information for this figure was gleaned from the following references: [2, 3, 13, 15–17, 20, 29, 31, 66, 75–84, 87, 93, 107, 123, 148, 233, 255, 257, 324, 325, 352, 354–356, 360–367, 374, 375, 377, 380–382, 384–386, 388, 393, 407, 415–417, 594, 595, 601, 602, 612, 648–650]. See also additional tables 2 and 5–9. Abbreviations: Gα13 (GNA13) heterotrimeric G protein alpha 13, ARHGEF1 Rho guanine nucleotide exchange factor (GEF) 1, PI3K phosphoinositide 3-kinase, mTORC1 mammalian target of rapamycin (mTOR) complex 1, BCL2 B-cell lymphoma protein 2, BCL6 B-cell lymphoma protein 6, EZH2 enhancer of zeste homologue 2, PD-1 programmed cell death, PD-L1 programmed cell death ligand, TGFβR transforming growth factor beta (TGFβ) receptor, HDACi inhibitors of histone deacetylases, BCL6i inhibitors of BCL6, AZA azacytidine, DAC decitabin

**Fig. 9:**
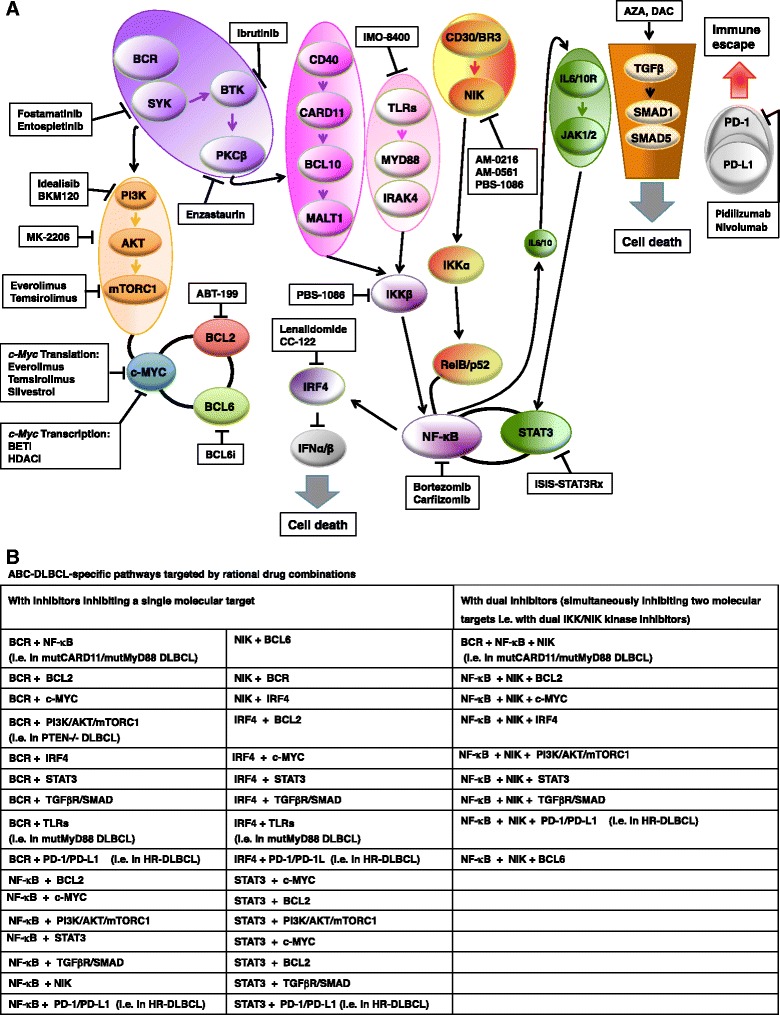
Mechanism-based combinatorial experimental regimens aimed at disrupting known oncogenic cooperation pathways in ABC-DLBCLs. Targets for rational mechanism-based combinatorial experimental regimens are shown for ABC-DLBCL (**a** and **b**). The black thick lines connecting the different drug targets pathways depict established biological interactions and/or dependencies. The single drugs listed in (**a**) have either proven clinical activity in patients with DLBCL or are currently in clinical trials for patients with B-cell lymphoma, including DLBCL. Pathways in ABC-DLBCL targeted by rational drug combinations using drugs tested as single agent in preclinical studies or clinical trials are shown in (**b**). Bortezomib and carfilzomib inhibit the 26S proteasome complex thereby blocking the degradation of negative regulators of cell cycle progression as well as of NF-κB inhibitory protein IκBα. Lenalidomide is an immunomodulatory agent directly binding to the E3 ubiquitin ligase substrate receptor CRBN and promoting the recruitment of its common substrates to the E3 ubiquitin ligase complex, thus leading to substrate ubiquitinylation and degradation [284] and subsequent repression of IRF4, a hallmark of ABC-DLBCL cells, thereby inhibiting the BCR-mediated canonical NF-κB-dependent pro-survival signaling pathways [90, 91]. Silvestrol inhibits RNA helicase and translation initiationfactor eIF4A thereby blocking translation (i.e., of oncogenic factors) [647]. Azacytidine (AZA) and decitabine/5-aza-2′-deoxycytidine (DAC/5-AZA) inhibit DNA methyltransferases (mainly DMNT1 and DMNT3) resulting in the re-expression of tumor-suppressor genes [419, 420]. Mechanism-based drug combinations targeting the biological interaction between MYC and BCL2 as well as MYC and the PI3K/AKT/mTORC1 pathway are aimed for both GCB- and ABC-DLBCL. Adapted from REF: [590, 591]. Information for this figure was gleaned from the following references: [2, 3, 13, 16–18, 20, 29, 31, 54, 57, 60, 65, 66, 75–81, 87, 90, 91, 93, 96–100, 103, 104, 107, 123, 133, 148, 171, 232, 233, 242, 247–250, 255, 257, 266, 270, 271, 285, 286, 289, 296, 297, 307–311, 313–317, 319, 323, 324, 326, 327, 360–365, 367, 374, 375, 377, 380–382, 384–386, 388, 393, 394, 398–402, 407, 415–417, 601, 602, 612, 620, 648–652]. See also Additional file 1: Table S2 and S5–S9. Abbreviations: BCR B cell receptor, BTK Bruton’s tyrosine kinase, CD40L CD40 ligand, JAK Janus kinase, CRBN cereblon, IRF4 interferon-regulatory factor 4, MALT1 mucosa-associated lymphoid tissue lymphoma translocation protein 1, BCL10 B cell lymphoma protein 10, TLR Toll-like receptor, MyD88 myeloid differentiation primary response 88, CARD11 caspase recruitment domain family, member 11, PKCβ protein kinase Cβ, IKK inhibitor kappa B (IκB) kinase, NF-κB nuclear factor-kappa B, NIK NF-κB inducing kinase, STAT signal transducer and activator of transcription, PI3K phosphoinositide 3-kinase, mTORC1 mammalian target of rapamycin (mTOR) complex 1, BCL2 B-cell lymphoma protein 2, BCL6 B-cell lymphoma protein 6, TGFβR transforming growth factor beta (TGFβ) receptor, HDACi inhibitors of histone deacetylases, BCL6i inhibitors of BCL6, PD-1 programmed cell death, PD-L1 programmed cell death ligand, AZA azacytidine, DAC decitabine

Since most patients with relapsed/refractory DLBCL show activation of multiple pathways including activation of feedback signaling pathways that can circumvent the drug target, most targeted therapies may not succeed as molecularly targeted single-agent monotherapy. Given the genetic diversity of relapsed/refractory DLBCL subtypes, and given that cells in different functional or anatomical compartments will have differing phenotypes, overcoming drug resistance to conventional and novel experimental immuno-chemotherapeutic regimens is highly reliant on multi-targeted mechanism-based subtype- and signature-specific combinatorial therapies concomitantly targeting multiple signaling pathways to restore apoptotic pathways in tumor cells and to inhibit pro-survival signaling from the stroma environment. Next-generation of multi-targeted combinatorial experimental therapies composed of new targets and conventional (immuno-)chemotherapeutic regimens may thus provide more immediate hope for relapsed/refractory patients who have failed current (immuno-)chemotherapeutic regimens. Ideally, one hopes to exploit synthetic lethality with multi-targeted mechanism-based combinatorial therapies that target the oncogenic rewiring of malignant cells [591, 592]. For instance, there is strong evidence for an oncogenic cooperation of the c-MYC and BCL2 signaling pathways in c-MYC-driven c-MYC/BCL2-double hit DLBCL subtypes *in vivo* [107, 366, 375, 377]. In mouse models, inhibiting BCL2 using ABT-199 prolongs the survival of mice with c-MYC-driven c-MYC/BCL2-double hit DLBCL tumors [375, 377]. In addition, several recent genetic and pharmacological studies *in vitro* and/or *in vivo* provided evidence for a functional cooperation between EZH2, BCL2 and BCL6 [77, 79], c-MYC and the PI3K/AKT/mTORC1 pathway [324, 593, 594], between NF-κB, STAT3, PI3K and c-MYC [233, 594, 595] as well as between non-canonical NF-κB signaling and BCL6 [27]. A scheme of mechanism-based combinatorial experimental regimens aimed at disrupting known oncogenic cooperation pathways in GCB-DLBCL and ABC-DLBCL are presented in Figs. 8 and 9.

Various mechanism-based experimental drug combinations are currently investigated in preclinical models and are already demonstrating promising activities *in vitro* and *in vivo*. For instance, combination studies *in vitro* suggest that the biological crosstalks between the c-MYC, PI3K/AKT/mTORC1, STAT3, IRF4 and/or BCR/NF-κB-dependent signaling pathways represent promising targets in a subset of ABC-DLBCL that may be exploited for a combination therapy of relapsed/refractory DLBCL together with cytotoxic (immuno-)chemotherapeutic agents, that are currently used to treat ABC-DLBCL [313, 595, 596]. Preclinical studies demonstrated that co-administration of the BCR inhibitor ibrutinib with the proteasome inhibitor bortezomib synergistically increased mitochondrial injury and apoptosis in highly bortezomib-resistant GCB and ABC-DLBCL *in vitro* [561]. Another recent preclinical study showed that the combination of ibrutinib and lenalidomide is synthetic lethal in blocking IRF4 expression, increasing IFNβ production, and killing ABC-DLBCL cells *in vitro* and *in vivo* [90]. Preclinical studies have also documented synergistic interactions between pan-HDACi (i.e., vorinostat, SNDX-275, or SBHA) and the novel proteasome inhibitor carfilzomib (CFZ) in GCB- and ABC-DLBCL cells sensitive or resistant to bortezomib [560]. A recent preclinical study showed that combinatorial multilevel inhibition of PI3K/AKT/mTORC1 and CDK1 cell-cycle pathways is very effective in inhibiting DLBCL proliferation and overcoming drug resistance and has been suggested as an effective strategy in treating drug resistant DLBCLs with overactive AKT and CDK1 [338]. Moreover, a large matrix-based high-throughput screening platform for the rapid and systematic identification of synergistic, additive, and antagonistic drug combinations has been recently established for DLBCL [233]. This matrix-based strategy was used to screen ABC-DLBCL cell lines for sensitivity to the BCR inhibitor ibrutinib in combination with nearly 500 established or investigational anti-cancer compounds [233]. This study identified a wide range of compounds, including inhibitors of the PI3K/AKT/mTORC1 signaling pathway (i.e., idelalisib, everolimus, BKM-120), BCL2 family inhibitors (i.e., ABT-199) and other BCR pathway inhibitors that cooperate with ibrutinib to kill ABC-DLBCL cell lines [233]. In addition, using a similar high-throughput screening platform, a more recent report demonstrated that ibrutinib strongly synergized with BET inhibitors CPI203 and JQ1 in killing ABC-DLBCL cells *in vitro* and in a xenograft mouse model *in vivo* [239]. Moreover, another *in vitro* screen using a panel of different drugs and DLBCL cell lines showed synergistic or additive effects of the BET inhibitor OTX015 when combined with several anti-lymphoma agents [407]. In line with previous studies, the combination of OTX015 and ibrutinib was strongly synergistic in ABC-DLBCL cells [407]. Besides ibrutinib, OTX015 acted also synergistically when combined with clinically achievable doses of other anti-lymphoma drugs such as rituximab, lenalidomide, the PI3Kδ inhibitor idelalisib, the mTORC1 inhibitor everolimus, the demethylating agent decitabine and the HDACi vorinostat [407]. The strongest synergism was obtained with the mTORC1 inhibitor everolimus [407]. The combination OTX015/rituximab appeared more active in ABC-DLBCL cell lines [407], which was explained by the common targeting of the IL10 and STAT3 pathway by both OTX015 and rituximab [165, 407]. A detailed list of preclinical multi-targeted combinatorial experimental treatments for DLBCL-NOS is shown in Additional file 1: Table S10.

Another elegant and efficient way to further circumvent chemotherapy resistance might be the application of rationally designed highly selective dual inhibitors simultaneously blocking two or more molecular targets in DLBCL. The combination of highly selective dual inhibitors may strongly increase the number of concomitantly targeted signaling pathways without leading to severe side effects. As already mentioned before, the dual mTORC1/2 catalytic inhibitor AZD2014 acts highly synergistically in combination with the BTK inhibitor ibrutinib and causes apoptosis both in vitro and in vivo and resulted in tumor regression in an ABC-type DLBCL xenograft model [232]. A recent high-throughput screening study performed by Griner M. et al. confirmed these results demonstrating that the combination of NVP-BEZ235 and the BTK inhibitor ibrutinib is highly synergistic in killing ABC-subtype DLBCL cell lines in vitro [233]. NVP-BEZ235 is an ATP-competitive dual panPI3K/mTORC1/2 with retained activity against cells harboring PI3K-activating mutations [597–599]. Moreover, Rahmani and colleagues, recently demonstrated that NVP-BEZ235 in combination with the HDACi panobinostat markedly potentiates HDAC inhibitor activity in DLBCL cells, including in poor-prognosis ABC- and double-hit subtype in vitro and induced tumor regressions in DLBCL xenograft models [597]. When combined, BEZ235 and panobinostat appeared well tolerated by mice in in vivo studies [597]. A list of dual inhibitors currently evaluated in preclinical and/or clinical studies in DLBCL is shown in Additional file 1: Table S11.

Remarkably, several recent clinical studies of multi-targeted combinatorial experimental therapies composed of new targets and conventional (immuno-)chemotherapeutic regimens showed already promising effects and at least in part, could confirm the preclinical observations. For instance, novel agents such as lenalidomide in combination with rituximab were very effective in relapsed/refractory DLBCL, showing ORR of up to 70 % and CR of up to 60 % [289, 429] whereas combinations with other novel agents such as mTORC inhibitors were less effective [328]. Clinical trials with ibrutinib combinations are still ongoing. Moreover, combinations of R-CHOP with lenalidomide or epratuzumab in experimental clinical studies showed promising effects in first-line treatment of DLBCL [296, 297, 600]. A detailed list of ongoing experimental clinical multi-targeted studies combining novel experimental agents with or without conventional (immuno-)chemotherapy in newly diagnosed or relapsed/refractory DLBCL is shown in Additional file 1: Tables S12-14.

Mechanism based multi-targeted combinatorial therapies combining multiple novel agents with rituximab and/or conventional chemotherapy might represent much more effective treatment regimens with less toxicity for individualized molecular precision therapy in relapsed/refractory DLBCL. These novel multi-targeted drug combinations may be eventually already incorporated as part of a personalized first-line precision therapy, where the impact on preventing relapse will hopefully lead to a significantly improved overall survival for patients with aggressive subtypes of DLBCL. However it is important to note that one has to be cautious regarding the side effects of multi-targeted mechanism-based combinatorial therapies. As more targeted agents are developed, the timing of administration with other agents and dose of each single drug in clinical trials will become increasingly important to define mechanism-based synergistic combinations associated with minimal toxicities rather than simply adding new precision medicines to existing chemotherapeutic regimens, thus ensure maximal efficacy while minimizing side effects.

Unexpected and/or synergistic toxicities can represent a practical limitation to clinical development. Given the synergy between drugs such as ibrutinib or lenalidomide and multiple agents uncovered thus far, it may eventually be possible to combine in a personalized manner conventional immuno-chemotherapeutic regimes with three, four or more novel single-agent drugs simultaneously inhibiting the most important oncogenic signaling pathways to overcome the aggressive nature of HR-and BCR-subtype ABC-DLBCL or other subtypes of relapsed/refractory DLBCLs and thereby ensure maximal efficacy while minimizing side effects. Thus, subtype and subset-specific personalized multi-targeted combinations of novel highly selective therapeutic drugs (i.e., BCL2-, BCL6-, NF-κB- or STAT3-specific small molecule inhibitors) together with or without ADC and/or standard (immuno-)chemotherapy (i.e., R-CHOP or DA-EPOCH-R) might provide a novel therapeutic strategy to increase the sensitivity of relapsed/refractory DLBCLs towards conventional immuno-chemotherapeutic regimens and thus pave the way to develop novel personalized therapeutic strategies for patients suffering from highly aggressive relapsed/refractory DLBCLs.

## Concluding remarks

Although the clinical outcome in DLBCL has tremendously improved over the last decades and will likely improve further with the introduction of novel specific anti-cancer agents and therapeutic approaches, relapsed/refractory DLBCL remains a major cause of morbidity and mortality.

Unfortunately, the ongoing efforts exploring novel conditioning regimens and maintenance approaches for relapsed/refractory DLBCL will have limited impact for most of the current patients with relapsed/refractory DLBCL, because only a minority of patients proceed to this second line treatment. On the other hand the improved understanding of DLBCL subtypes and etiology of relapsed/refractory DLBCL is leading to the identification of targeted drugs that not only may allow for subtype-specific and molecularly targeted combinational therapy in the management of patients with relapsed/refractory DLBCL who are not cured in the rituximab era but also to development of very efficient personalized first-line therapeutic regimens. Due to the extensive intra-patient and intra-tumor genetic heterogeneity in DLBCL and the efficiency of single-agent therapies it is evident that rational personalized therapeutic regimens using novel agents directed at distinct cellular pathways and properly tested in well-designed (personalized) clinical trials are needed for optimal therapy for relapsed or refractory DLBCL. Such rational personalized therapeutic regimens will also require both, positive selection strategies to identify patients most likely to respond to therapy as well as a negative selection approach to determine which patients may be resistant to a given therapy [601, 602], thus, taking the molecular subtypes and molecular signatures of DLBCL into account. The most suitable strategy to achieve this is to identify the patient specific mutational and gene expression profiles either via a combinatorial analysis using mass-spectrometry (protein expression analysis), next generation sequencing (RNA expression analysis) and candidate gene sequencing (mutation analysis) or alternatively, using combinations of thoroughly validated clinical biomarkers highly specific for each molecular subtype and gene expression signature.

There is preliminary evidence that novel agents such as lenalidomide or epratuzumab could be very effective in relapsed/refractory DLBCL when used as novel agent in combination with rituximab or R-CHOP. Conversely, many other novel agents such as enzastaurin alisertib, or everolimus were less effective when used as novel agent in combinations with rituximab or R-CHOP. Thus, large multicenter non-randomized and personalized long term clinical phase III trials combining 2, 3 or more novel agents with or without standard immunochemotherapy (i.e., R-CHOP21 or DA-ECHOP-R) are required to investigate whether (personalized) multi-targeted combinations of novel experimental agents together with rituximab or CHOP21 regimens have a superior efficacy when compared with optimized R-CHOP21 or DA-ECHOP-R regimens in relapsed/refractory DLBCL or as first line treatments for newly diagnosed high-risk DLBCL.

A better understanding of the functions of novel potential drug targets such as PLCγ2, PRPS2, NOTCH1/2, STAT1, DTX3L, or ARTD9 and the generation of novel highly selective small molecule inhibitors with negligible off target activity are also required to give us the opportunity to develop novel personalized therapeutic strategies targeting novel subtype-specific pathways, thereby also minimizing toxicities by limiting exposure of patients to agents to which their disease would be unlikely to respond. Moreover, acquired drug resistance is very likely mediated by a finite set of pathways whose relative contributions will vary in individual patients which would allow genomic and proteomic analyses to be performed on primary samples from patients with relapsed/refractory DLBCL to determine which targets need to be suppressed or activated to restore sensitivity to drugs that were used successfully in a prior line of therapy, or to optimize the efficiency of the available therapeutic personalized regimens. In addition the interactions between relapsed/refractory DLBCL tumors and their microenvironment are biologically and clinically crucial. Thus, an understanding of how novel potential drug targets such as STAT1, ARTD9 and/or DTX3L may modulate the interaction between (relapsed or refractory) DLBCL tumors and their microenvironment should also provide new therapeutic options.

Together, multi-targeted combinatorial personalized precision therapies composed of multiple novel agents highly selectively targeting at distinct cellular pathways and DLBCL subtypes (i.e., ABC, GCB) or subsets (i.e., BCR, OxPhos, MD or HR) combined with ADC or conventional rituximab based chemotherapy will most likely represent the future mainstay of treatment for patients with newly diagnosed high-risk DLBCL or relapsed/refractory DLBCL and thus may provide hope for relapsed patients who have failed current chemotherapies in the rituximab era.

## Additional file

Additional file 1:
**Table S1–S14. **(PDF 747 kb)
