# Supplementary material for: Novel drug targets for personalized precision medicine in relapsed/refractory diffuse large B-cell lymphoma: a comprehensive review
Source: Mol Cancer. 2015 Dec 11;14:207. doi: 10.1186/s12943-015-0474-2 (PMC4676894; doi:10.1186/s12943-015-0474-2)
Supplement: Additional file 1: — Table S1–S14. (PDF 747 kb) [file 12943_2015_474_MOESM1_ESM.pdf]

Tables S1-S14

Table S1

Novel potential Drug Targets in DLBCL-NOS, including relapsed/refractory DLBCL

Target	Molecular Subtype	Molecular signature	Target in refractory DLBCL?	Inhibitor, Antibody, SiRNA	Study
**Signaling Receptors:**					
PD-1	HR?<br>NA	NA	Yes	Pidilizumab, Nivolumab, MPDL3280A (anti-PD-1 antibodies)	[1-4]
CD19	NA	NA	Yes	Anti-CD19 CAR T cells (T cells expressing anti-CD19 CARs recognize and kill CD19+ target cells)	[5, 6]
CD22	NA	NA	Yes	Epratuzumab (anti-CD22 mAb)	[7]
CD43	ABC, type-3	NA	Yes	NA	[8, 9]
CD52	NA	NA	Yes	Alemtuzumab (anti-CD52mAB)	[10]
**Cytoskeleton:**					
Tubulin<br>CD19 (indirect drug target)	NA	NA	Yes	SAR3419, an anti-CD19 antibody conjugated to the cytotoxine Maytansine DM4, a potent inhibitor of tubulin polymerization and microtubule assembly	[11-14]
Tubulin<br>CD22 (indirect drug target)	ABC, GCB,	NA	NA	Pinatuzumab-Vedotin CD22 antibody linked to the microtubule disrupting agent monomethyl auristatin E (MMAE).	[15]
Tubulin<br>CD30 (indirect drug target)	ABC, GCB,	“CD30”	Yes	Brentuximab vedotin (anti-CD30 antibody linked to the microtubule disrupting agent monomethyl auristatin E (MMAE).	[16-18]
Tubulin<br>CD79B (indirect drug target)	ABC, GCB,	NA	NA	Polatuzumab-Vedotin (anti CD79B antibody linked to the microtubule disrupting agent monomethyl auristatin E (MMAE).	[15]
Ezrin-Radixin-Moesin (ERM) family	ABC, GCB, type-3 (n.c.i.)	BCR (n.c.i.)	NA	Ezrin inhibitors NSC668394, NSC305787 (Interference with ERM function impairs BCR survival signaling)	[19]

**DNA (Genome):**					
DNA<br>CD22 (indirect drug target)	NA	NA	Yes	Inotuzumab ozogamicin (INO)<br>Anti-CD22 antibody linked to the DNA damaging toxin N-acetyl-γ-calicheamicin dimethyl hydrazide (CalichDMH)<br>(induces DNA double strand breaks)	[14, 20]
**mRNA Translation Machinery:**<br>**(i.e. Oncogene translation)**					
Elongation Factor 2	NA	NA	Yes	Moxetumomab Pasudotox (HA22/ BL22)<br>Anti-CD22 antibody linked to a to an ADP-ribosylating Pseudomonas Exotoxin<br>(Inhibits protein synthesis)	[13, 14, 21]
Translation initiation complex eIF4F<br>(eIF4E, eIF4G and eIF4A)	ABC, GCB, type-3<br>(n.c.i.)	BCR<br>(n.c.i.)	NA	eIF4A inhibitor Silvestrol<br>mTORC1 inhibitor CC214-1	[22, 23]
**NF-κB Pathway:**					
26S proteasome	ABC,<br>(GCB?, type-3)	BCR, HR,<br>(n.c.i.)	Yes	Bortezomib, carfilzomib<br>(26S inhibitors)	[24-27]
NF-κB/RELA/p65,<br>NF-κB1/p50,<br>NF-κB2/p52<br>RELB	ABC<br>(GCB?, type-3)	BCR, HR,<br>(n.c.i.)	Yes	BAY-11-7082 (NF-κB inhibitor)	[28-33]
IκB kinase (IKK) complex	ABC<br>(GCB?, type-3)	BCR, (n.c.i.)	NA	*IKKα* siRNA,<br>MLX, MLN120B<br>(IKKβ inhibitors)	[34, 35]
NIK	ABC<br>(GCB?, type-3)<br>(n.c.i.)	BCR<br>Non-BCR	NA	Mouse model for DLBCL<br>NIK inhibitors AM-0216, AM-0561, PBS-1086 tested in multiple myeloma	[31-33, 36, 37]
IκB-ζ	ABC<br>(GCB?, type-3)	BCR, (n.c.i.)	NA	*IκB-ζ* siRNA	[38]
MALT1	ABC	BCR, (n.c.i.)	NA	MI-2, mepazine, thioridazine, and promazine<br>(MALT inhibitors)	[39-42]
CARD11	ABC	BCR, (n.c.i.)	NA	zVRPR-fmk (CARD11 inhibitor)	[43, 44]
MYD88	ABC	BCR/	NA	*MYD88* siRNA	[45]

		MYD88 mutation, (n.c.i.)			
**Ubiquitinylation/ Neddylation**					
CSN5<br>COP9-signalosome (CSN) deneddylating activity	ABC, GCB	(n.c.i.)	NA	Doxocycline	[46]
UBC13/UEV1A	ABC, GCB	BCR, (n.c.i.)	NA	NSC697923	[47]
RNF31	ABC	BCR/ RNF31 mutations, (n.c.i.)	NA	Stapled RNF31 N-Q622L peptide	[48]
NEDD8	ABC, GCB	NA	NA	MLN4924, a NEDD8-activating enzyme inhibitor	[49, 50]
**IFNα/β Signaling:**					
IRF4	ABC	BCR, (n.c.i.)	Yes	Lenalidomide	[51, 52]
Cereblon	ABC, GCB, type-3 (n.c.i.)	BCR, (n.c.i.)	Yes	Lenalidomide, CC-122<br>*Cereblon*-siRNA	[51-56]
**BCR Signaling:**					
CD79A, CD79B	ABC	BCR, CD79A/B mutation	NA	siRNA	[57, 58]
BTK	ABC	BCR, (n.c.i.)	Yes	Ibrutinib	[27, 57]
SYK	ABC	BCR, (n.c.i.)	NA	Fostamatinib (R788)(SYK inhibitor)<br>Entospletinib (GS-9973)	[57, 59-61]
PKCβ	ABC	BCR, CD79A/B mutation,	NA	Enzastaurin, Sotrastaurin/AEB071 (PKCβ inhibitor)	[57, 58, 62]
SRC family	ABC	BCR, (n.c.i.)	NA	Dasatinib, pp2	[57, 63, 64]
PLCγ2	ABC, GCB, type-3 (n.c.i.)	BCR, (n.c.i.)	NA	U73122 (PLC-inhibitor)	[64]
**PI3K/AKT/mTORC Pathway:**					
PI3K/PI3Kδ	ABC, GCB, type-3	BCR, HR, (n.c.i.)	Yes	NVP-BKM120<br>Idelalisib<br>BAY 80-6946	[65-67]
AKT	ABC, GCB, type-3 (n.c.i.)	BCR, HR, MD, (n.c.i.)	Yes	MK-2206, nelfinavir	[68-70]
mTORC1	ABC, GCB, type-3	BCR, (n.c.i.)	Yes	Rapamycin<br>Everolimus	[71-74]

				Temsirolimus<br>CC-223, AZD2014,<br>BEZ235, QL-X-138,<br>PRT062070, OSI-027	
mTORC2	ABC, GCB, type-3 (n.c.i.)	BCR, HR, MD, (n.c.i.)		CC-223, AZD2014,<br>BEZ235, QL-X-138,<br>PRT062070, OSI-027	[73, 75]
**Inhibitor of Apoptosis:**					
BCL2	GCB, (ABC, type-3)	MD, (n.c.i.)	Yes	ABT-737, A263, ABT-199<br>GX15-070 (obatoclax)<br>(BH3-mimetics)	[26, 76-84]
MCL1	ABC, (GCB, type-3)	NA	Yes	GX15-070 (obatoclax)	[76, 77, 80, 85-88]
MCL1<br>(CDK1, CDK2, CDK5, CDK9)	ABC, (GCB, type-3)	MD, (n.c.i.)	NA	Dinaciclib	[89]
BCL-XL	NA	NA	NA	ABT-737, A263, ABT-199, GX15-070 (obatoclax)	[26, 79]
BFL-1	NA	NA	NA	GX15-070 (obatoclax)	[76, 77, 85-87]
XIAP	NA	NA	Yes	Small-molecule XIAP antagonist 1396-12	[90]
Survivin	ABC, GCB, type-3 (n.c.i.)	NA	Yes	YM155	[91-94]
MDM2	ABC, (n.c.i.)	MD, (n.c.i.)	NA	Nutlin-3a	[95]
**B Cell specific Transcription Factors:**					
BCL6	ABC, (GCB, type-3)	BCR, HR, MD, (n.c.i.)	NA	RI-BPI	[96-100]
YY1	ABC, GCB, type-3	MD? (n.c.i.)	NA	*YY1* siRNA	[101-103]
FOXP1	ABC, (n.c.i.)	NA	Yes (n.c.i.)	*FOXP1* siRNA	[104-106]
β-catenin	NA	NA	NA	*FOXP1* siRNA,<br>*β-catenin* siRNA,	[105]
**Stress Proteins:**					
HSP70<br>HSP90α<br>HSP90β	ABC, GCB, type-3 (n.c.i.)	OxPhos (Hsp90α)<br>BCR (Hsp90β)	Yes (n.c.i.)	IPI-504<br>PU-H71	[97, 107-109]
**JAK/STAT Pathways:**					
IL10/IL10R	ABC, GCB, type-3	NA	NA	Anti IL10R blocking antibody	[110, 111]

	(n.c.i.)				
JAK1<br>JAK2	ABC, GCB, type-3	NA	NA	Fedratinib (TG101348)	[111-113]
STAT1	GCB, (ABC, type-3) (n.c.i.)	HR, (n.c.i.)	NA	*STAT1*-siRNA	[112]
STAT3	ABC, (GCB, type-3) (n.c.i.)	NA	NA	Fedratinib (TG101348)	[114-117]
**C-MYC Pathway:**					
c-MYC	ABC, GCB, type-3 (n.c.i.)	MD (n.c.i.)	Yes	*c-MYC* siRNA	[118-121]
BRD4<br>BRD2	ABC, (GCB, type-3?) (n.c.i.)	MD, BCR (n.c.i.)	Yes (n.c.i.)	JQ1, PFI-1, CPI203, I-BET151, OTX015, ARV-825 (PROTAC)	[122-131]
PRPS2	NA	MD, (n.c.i.)	NA	*PRPS2* siRNA	[132]
Aurora A kinase	ABC, GCB, type-3, (n.c.i)	MD, (n.c.i.)	Yes	Alisertib (MLN8237)	[133, 134]
**Epigenetic Modifiers:**					
DNMTs	NA	NA	Yes (n.c.i.)	Decitabine /5-aza-2′-deoxycytidine (DAC/5-AZA),<br>Azacytidine (AZA)	[135]
EZH2	GCB, (n.c.i.)	NA	NA	GSK126, CPI-360	[136-143]
HDACs<br>(HDAC1-4, 6)	GCB, ABC, type-3 (n.c.i.)	NA	Yes (n.c.i.)	Vorinostat<br>Panobinostat<br>LBH589<br>Ricolinostat	[115, 144-146]
Sirtuins<br>(SIRT1)	ABC, GCB (n.c.i.)	NA	Yes (n.c.i.)	Cambinol, Niacinamide	[144, 145, 147]
**ROS-OxPhos-Pathways:**					
PPARγ	ABC, GCB, type-3	OxPhos	NA	T0070907 and GW9662	[148]
γ-glutamyl cysteine synthase (GCS)	ABC, GCB, type-3	OxPhos	NA	*GCS*-siRNA	[148]
fatty acid oxidation program	ABC, GCB, type-3	OxPhos	NA	4-bromocrotonic acid (BrCA)	[148]
**RAS/RAF/MEK/ERK Signaling:**					
ERK1	GCB, type-3	NA	NA	ERK inhibitor no. 76 [3-(2-	[149, 150]

ERK2<br>CHK2	(n.c.i.)			aminoethyl)-5-((4-ethoxyphenyl) methylene)-2,4-thiazolidinedione<br>CHK2 inhibitor II (2-(4-(4-chlorophenoxy) phenyl)-1H-benzimidazole-5-carboxamide hydrate)	
MEK	NA	NA	NA	AZD6244	[151]
**Other Kinases:**					
PIM1<br>PIM2	ABC, GCB, type-3	NA	NA	ETP-39010	[152-154]
JNKs	ABC, (n.c.i.)	(BCR?) NA	NA	SP600125	[155]
CDK1, CDK2, CDK5, CDK7, CDK8, CDK9,	ABC, (GCB, type-3) (n.c.i.)	MD, (n.c.i.)	NA	Dinaciclib<br>Seliciclib (CYC202, R-roscovitine)	[89, 156, 157]
**Mitogenic Signaling:**					
p70S6K/p85S6K	NA	NA	NA	Rapamycin	[158]
CDK1	NA	NA	NA	UCN-01	[158]
KSP	ABC, GCB (n.c.i.)	NA	NA	SB-743921	[159]
**NOTCH Pathway/ DELTEX Family:**					
DTX3L	ABC, GCB, type-3	HR, BCR (n.c.i.)	NA	NA	[160]
NOTCH1<br>NOTCH2	ABC, (GCB?), type-3	HR, (n.c.i.)	NA	NA	[137, 161-163]
**ARTD Family:**					
ARTD8?<br>ARTD9	ABC, GCB, type-3	HR, BCR (n.c.i.)	NA	NA<br>*ARTD9* siRNA	[112, 160, 164, 165]
**PTM Enzymes:**					
Rab geranylgeranyl transferase (prenylation of the oncogenic Rab family)	NA	NA	Yes (n.c.i.)	FTI-277<br>GGTI-298	[166]
**Angiogenesis:**					
VEGF	NA	Stromal II	NA	Bevacizumab (Avastin)	[167-169]
PDGFRβ	NA	NA	NA	Imatinib	[170]

**Metastasisation:**					
CXCR4	GCB, ABC (n.c.i.)	NA	NA	AMD3100	[171-173]
FoxM1	GCB, ABC (n.c.i.)	NA	NA	Thiostrepton	[174]
**Drug Resistance:**					
ABCG2 (Stroma induced drug tolerance)	NA	NA	NA	Fumitremorgin C	[175]
Thioredoxin-1	NA	NA	NA	PX-12	[176]
ALDH1A1	NA	NA	Yes (n.c.i.)	Diethylaminobenzaldehyde (DEAB)	[177]
**Signaling Inhibitors:**					
PDE4B (PDE2,3,7 ?) (inactivates cAMP and abrogates its inhibitory effects in DLBCL)	NA	NA	NA	BAY 60-7550/ cilostamide, cilostazol, siguazodan, milrinone, roflumilast/ papaverine, BRL-54680	[178-180]
**AKT-14-3-3ζ signaling:**					
AKT	NA	NA	NA	Akt inhibitor VIII	[181]
14-3-3ζ	NA	NA	NA	*14-3-3ζ* siRNA	[182]
**Autophagy:**					
General inhibition	NA	NA	NA	Chloroquine/3-methyladenine/Bafilomycin A 1	[65]
**Constitutive activation of DNA damage response (DDR) pathway**					
CHK1/2	ABC, GCB, type-3 (n.c.i.)	NA	NA	PF-0477736 AZD-7762	[183]

DLBCL diffuse large B cell lymphoma, *ABC* activated B cell-like, *GCB* germinal center B cell-like, *OxPhos* oxidative phosphorylation subtype, *BCR* BCR-activated B-cell (antigen) receptor/proliferation subtype**,** *HR* host response signature subtype, *MD* c-MYC-driven signature subtype, *n.c.i.* not completely investigated, *NA* not available, *type-3* unclassified DLBCL-NOS, *NF-κB* nuclear factor-kappa B, *IKK* inhibitor of kappa B kinase, *IKB* inhibitor of kappa B, *MALT1* mucosa-associated lymphoid tissue lymphoma translocation protein 1, *CARD11* caspase recruitment domain family, member 11, *MYD88* myeloid differentiation primary response 88, *UBC13/UEV1A* ubiquitin-conjugating enzyme, *RNF31* ring finger containing protein 31, *NEDD8* neural precursor cell expressed, developmentally down-regulated 8, *IRF4* interferon-regulatory factor 4, *BTK* Bruton's tyrosine kinase, *SYK* spleen tyrosine kinase, *PKCβ*

protein kinase Cβ, *PLCγ2* phospholipase C gamma 2, *mTORC1* mammalian target of rapamycin (mTOR) complex 1, *PI3K* phosphoinositide 3-kinase, *BCL* B-cell lymphoma protein, *MCL1* myeloid cell leukemia 1, *XIAP* X-linked inhibitor of apoptosis, E3 ubiquitin protein ligase, *MDM2* murine double minute E3 ubiquitin ligase, *YY1* Ying Yang 1, *FOXP1* forkhead box P1, *HSPs* heat shock proteins, *IL* interleukin, *JAK* Janus kinase, *STAT* signal transducer and activator of transcription, *BRD* BET bromodomain protein, *PROTAC* proteolysis targeting chimera, *PRPS2* phosphoribosyl-pyrophosphate synthetase 2, *DNMTs* DNA methyltransferases, *EZH2* enhancer of zeste homologue 2, *HDACs* histone deacetylases, *PPAR* peroxisome proliferators activated receptor, *ERK* extracellular signal-regulated kinase, *PIM1* (proviral integration site for the Moloney murine leukemia virus) kinase 1, *CDK* cyclin-dependent kinase, *KSP* kinesin spindle protein, *DTX3L* deltex (DTX)-3-like E3 ubiquitin ligase, *ARTD* ADP-ribosyltransferase diphteria toxin like, *VEGF* vascular endothelial growth factor, *PDGFRβ* platelet-derived growth factor receptor beta, *CXCR4* chemokine (C-X-C motif) receptor 4, *FoxM1* forkhead box M1, *ABCG2* ATP-binding cassette, sub-family G, member 2, *ALDH1A1* aldehyde dehydrogenase 1A1, *PDE* phosphodiesterases

Table S2

Novel single experimental agents in clinical trials in relapsed/refractory DLBCL-NOS

**Inhibitor or Agent**	**Target or Agent mechanism**	**No. of patients**	**Response rate (DLBCL Subtype)**	**Response duration**	**Clinical trials**	**Study**
Pidilizumab<br><br>(after ASCT)	PD-1	66 (24)	ORR: 51%<br>CR: 34%<br>PR: 17%<br>SD: 27%<br>PD: 11%	DOR: 10m<br>16m-PFS: 70%<br>16m-OS: 85%	Phase I/II	[1, 2]<br>Trial-No: NCT00532259
Nivolumab<br><br>(after ASCT)	PD-1	120 (estimated)	NA	NA	Ongoing Phase II	Trial-No: NCT02038933
Daratumumab	CD38	NA	NA	NA	Ongoing Phase II	Trial-No: NCT02413489
Bortezomib	Proteasome (NF-κB)	53 (estimated)	NA	NA	Ongoing Phase II	Trial-No: NCT01965977
IMO-8400 (TLR7/8/9 antagonist)	TLR7/8/9	NA	NA	NA	Ongoing Phase I/II	Trial-No: NCT02252146
Lenalidomide (after ASCT)	CRBN (IRF4) (Immuno-modulatory drug)	52/47	ORR: 29/28%<br>CR/CRu: 10/11%<br>PR: 19/17%<br>SD: 25/26%<br>PD: 38/47%	DOR: 7.9m<br>DOR(CR): 15m<br>PFS(CR): 16.8m<br>PFS(PR): 7.3m<br>OS: NA	Phase II	[184-186]<br>Trial-No: NCT00765245
Lenalidomide	CRBN (IRF4) (Immuno-modulatory drug)	40<br>(GCB: 23, non-GCB: 17)	ABC/type-3:<br>ORR: 52.9%<br>CR: 23.5 %<br>PR: 24 %<br>SD: 0%<br>PD: 40%<br><br>GCB:<br>ORR: 8.7%<br>CR: 4.3 %<br>PR: 24 %<br>SD: 30.4<br>PD: 61%	PFS: 6.2m<br>OS: 13.5m<br><br>PFS: 1.7m<br>OS: 14m	Phase II	[187]
Lenalidomide	CRBN (Aiolos, Ikaros, IRF4) (Immuno-modulatory drug)	51/25<br>(IHC: GCB 23, ABC 28)<br><br>(GEP: GCB 14, ABC 11)	ABC/type-3 (IHC):<br>ORR: 28.6%<br><br>GCB (IHC):<br>ORR: 26.1%<br><br>ABC (GEP):<br>ORR: 45%<br><br>GCB (GEP):<br>ORR: 21 %	ABC/type-3 (IHC):<br>med. PFS: 15.1 wks<br>med. OS: 32.2 wks<br><br>GCB (IHC):<br>med. PFS: 10.1 wks<br>med. OS: 30 wks<br><br>ABC (GEP):<br>med. PFS: 82 wks<br>med. OS: 108 wks	Ongoing phase II/III	[188]<br>Trial-No: NCT01197560

				GCB (GEP):<br>med. PFS: 13.2 wks<br>med. OS: 30 wks		
CC-122	CRBN (Aiolos, Ikaros, IRF4) (Immuno-modulatory drug)	NA	NA	NA	Ongoing phase I	Trial-No: NCT01421524
CC-122	CRBN (Aiolos, Ikaros, IRF4) (Immuno-modulatory drug)	5	ORR: 40%<br>CR: 20%<br>PR: 20%<br>(preliminary data)	NA	Ongoing phase I	[189]
CC-122	CRBN (Aiolos, Ikaros, IRF4) (Immuno-modulatory drug)	21	ORR: 24%<br>CR: 5%<br>PR: 14.5 %<br>(preliminary data)	NA	Ongoing phase I	[56]
Ibrutinib (PCI-32765)	BTK	80 (75)<br>(ABC: 38<br>GCB: 20<br>Type-3: 17)<br><br>125	ABC:<br>ORR: 37%<br>CR: 17 %<br>PR: 24 %<br><br>GCB:<br>ORR: 5.3%	ABC:<br>PFS: 2m<br>OS: 10.3m<br><br>GCB:<br>PFS: 1.3m<br>OS: 3.35m	Phase I/II<br><br>Ongoing phase III/b	[190-192]<br>Trial-No: NCT01325701<br><br>Trial-No: NCT01804686
ACP-196	BTK	NA	NA	NA	Ongoing phase I	Trial-No: NCT02112526
Fostamatinib (R788)	SYK	23	ORR: 22 %<br>CR: 4.4 %<br>PR: 17.6%<br>SD: 17.4%<br>PD: 50%	NA	Phase I/II	[59]<br>Trial-No: NCT00446095
Fostamatinib (R788)	SYK	68	NA	NA	Phase II	Trial-No: NCT01499303
Entospletinib (GS-9973)	SYK	NA	NA	NA	Ongoing phase II	Trial-No: NCT01799889
Navitoclax (ABT-263)	BCL2 family	6	NA	NA	Phase I ongoing phase II	[193]<br>Trial-No: NCT00406809
ABT-199 (GDC-0199)	BCL2	20 (estimated)	NA	NA	Ongoing phase I	[194, 195]<br>Trial-No: NCT01328626
Temsirolimus	mTORC1	27	ORR: 28.1 %	DOR: 2.4m	Phase II	[72]

			CR/CRu: 12.5%<br>PR: 15.6%<br>SD: 18.8%<br>PD: 46.9%	PFS: 2.6m<br>OS: 7.3m<br>3y-OS: 27.1%		Trial-No: NCT00290472
Everolimus (RAD001)	mTORC1	47	ORR: 30%<br>CR/CRu: 0%<br>PR: 14%	NA	Phase II	[71]
CC-223	mTORC1<br>mTORC2	NA	NA	NA	Ongoing phase I/II	[75]<br>Trial-No: NCT01177397
Enzastaurin (LY317615)	PKCβ	55 (40)	ORR: 5.5%<br>CR/CRu: 5.5%	≥2m-PFS: 22%<br>≥4m-PFS: 12%<br>≥20m-PFS: 7.3%	Phase I/II	[196]<br>Trial-No: NCT00042666
Idelalisib (GS-1101, CAL-101)	PI3Kδ	NA	NA	NA	Ongoing phase I<br><br>Ongoing phase II	[66, 197]<br>Trial-No: NCT01796470
Idelalisib (GS-1101, CAL-101)	PI3Kδ	NA	NA	NA	Ongoing phase I	Trial-No: NCT02457598
Bay 80-6946	PI3Kδ	17 (NA) (Preliminary data)	ORR: 11.2%<br>CR/CRu: 5.6%<br>PR: 5.6%<br>SD: 40.3%<br>PD: 46.9%<br>(Preliminary data)	NA	Ongoing phase II	[67]<br>Trial-No: NCT01660451
Copanlisib	PI3Kδ,<br>PI3Kα	NA	NA	NA	Ongoing phase II	Trial-No: NCT02391116
Panobinostat (LBH-589)	HDACs	NA	NA	NA	Ongoing phase I/II	[198]<br>Trial-No: NCT01238692
Ricolinostat (ACY-1215)	HDAC6	NA	NA	NA	Ongoing phase I/II	[146]<br>Trial-No: NCT02091063
Decitabine (DAC)	DMNT1-3	42	NA	NA	Ongoing phase I	Trial-No: NCT00109824
MK-2206	AKT	NA	NA	NA	Ongoing phase II	Trial-No: NCT01481129
BKM-120	PI3K	NA	NA	NA	Ongoing phase II	Trial-No: NCT01693614
CPI-0610	BRD2-4 (BET)	NA	NA	NA	Ongoing phase I	Trial-No: NCT01949883
ISIS-STAT3Rx (Antisense Oligonucleotide Inhibitor of STAT3)	STAT3	NA	NA	NA	Ongoing phase I/II	Trial-No: NCT01563302
Obinutuzumab (GA101) Third-	CD20	25	ORR: 32%<br>BOR: 20%	DOR: 9.8m<br>PFS: 2.7m	Phase II	[199]<br>Trial-No: NCT00517530

generation anti-CD20 mAb						
ABC294640	Sphingosine kinase 2	NA	NA	NA	Ongoing phase I	Trial-No: NCT02229981

DLBCL diffuse large B cell lymphoma, *ABC* activated B cell-like, *GCB* germinal center B cell-like, *ORR* overall response rate, *BOR* best ORR, *PR* partial response, *CR* complete response, *CRu* unconfirmed CR, *SD* stable disease, *PD* progressive disease, *DOR* median duration of response, *OS* median overall survival, *PFS* median progression-free survival, *NA* not available/not applicable, *wks* weeks, *m* months, *y* year, *ASCT* autologous stem-cell transplantation, *PD-1* programmed cell death 1, *NF-κB* nuclear factor-kappa B, *TLR* Toll like receptor, *IRF4* interferon-regulatory factor 4, *BTK* Bruton's tyrosine kinase, *SYK* spleen tyrosine kinase, *PKCβ* protein kinase Cβ, *PLCγ2* phospholipase C gamma 2, *DNMTs* DNA methyltransferases, *PI3K* phosphoinositide 3-kinase, *BCL* B-cell lymphoma protein, *BRD* BET bromodomain protein, *STAT* signal transducer and activator of transcription, *CRBN* cereblon

Table S3:
Experimental clinical studies combining novel experimental agents with conventional (immuno-) chemotherapy (R-CHOP, DHAP) in first-line treatment of newly diagnosed DLBCL

Treatment	Targets	DLBCL-NOS Subtype	Age	No. of patients	Response rate	Response duration	Clinical trial	Study
Bortezomib<br>+<br>CHOP/DA-EPOCH	Proteasome (NF-κB)	ABC	54 (18-78) years	49 (44)	ABC:<br>ORR: 83 %<br>CR: 41.5%<br>PR: 41.5%<br>GCB:<br>ORR: 13 %<br>CR: 6.5%<br>PR: 6.5%	ABC:<br>DOR: 10.2m<br>PFS: 2.8m<br>OS: 10.8m<br>GCB:<br>DOR: 10.2m<br>PFS: 2.8m<br>OS: 3.4m	Phase II	[24]<br>Trial-No: NCT00057902
Bortezomib<br>+<br>EPOCH	Proteasome (NF-κB)	NA	50	NA	NA	NA	Phase II	Trial-No: NCT00054665
Enzastaurin (LY317615)<br>+/-<br>R-CHOP	PKCβ<br>CD20	NA	100	100 (57/43)	R-CHOP/enza:<br>ORR: %<br>CR/CRu: 36%<br>PR: %<br><br>R-CHOP:<br>ORR: %<br>CR/CRu: 26%<br>PR: %	R-CHOP/enza:<br>DOR: 36m<br>1y-PFS: 72%<br>2y-PFS: 59%<br><br>R-CHOP:<br>DOR: 23m<br>1y-PFS: 55%<br>2y-PFS: 49%	Phase II	[200]<br>Trial-No: NCT00451178
Enzastaurin (LY317615)<br>+<br>R-GEMOX	PKCβ<br>CD20	NA	NA	NA	NA	NA	Phase II	Trial-No: NCT00436280
Lenalidomide<br>+<br>Rituximab	CRBN (IRF4)<br>CD20	GCB, ABC, type3, (n.c.i)	62 (43-79) years	17	ORR: 41.5%<br>CR: 35%	DOR: 26.5m<br>2y-PFS: 38%<br>2y-OS: 45%	Phase II	[201]
Lenalidomide<br>+<br>Rituximab		GCB, ABC, type3 (n.c.i)	65 (24-84) years	32	ORR: 28 %<br>CR: 22.2 %<br>PR: 6%<br>SD: 28%<br>PD: 37%	DOR: 10.2m<br>PFS: 2.8m<br>OS: 10.2m	Phase II	[202]<br>Trial-No: NCT00294632
Lenalidomide<br>+<br>R-ICE/BEAM	CRBN (IRF4)<br>CD20	GCB, ABC, type3 (n.c.i)	61.5 (41-75) years	15	ORR: 73% (GCB: 100%)<br>CR: 60%<br>PR: 13%	DOR: 8m	Phase I/II	[203]<br>Trial-No: NCT01241734
Ofatumumab (Arzerra)<br>+<br>DHAP	CD20	NA	NA	NA	NA	NA	Phase III	Trial-No: NCT01014208

Veltuzumab<br>+<br>90Y-Epratuzumab-Tetraxetan<br><br>(Radio-immuno-therapy)	CD20<br>CD22<br><br>Radio-immuno-therapy (radio-labeled anti CD22 antibody, 90Y-epratuzumab tetraxetan)	NA	73<br>(52-84)<br>years	8	ORR: 50%<br>CR: 37.5%<br>PR: 12.5%<br>SD: 12.5%	NA	Phase I	[204]<br>Trial-No: NCT01101581
Azacytidine (AZA)<br>(CC-486)<br>+<br>R-CHOP	DMNTs<br>CD20	NA	65<br>(43-83)<br>years	12	ORR: 92%<br>CR: 92%	DOR: 13m	Phase I	[135]

DLBCL diffuse large B cell lymphoma, *ABC* activated B cell-like, *GCB* germinal center B cell-like, *ORR* overall response rate, *PR* partial response, *CR* complete response, *CRu* unconfirmed CR, *SD* stable disease, *PD* progressive disease, *DOR* median duration of response, *OS* median overall survival, *PFS* median progression-free survival, *NA* not available/not applicable, *m* months, *y* year, *ASCT* autologous stem-cell transplantation, *R-CHOP* rituximab plus cyclophosphamide, doxorubicin, vincristine, and prednisone, *R-GEMOX* rituximab, gemcitabine and oxaliplatin, *DHAP* dexamethasone, cytarabine, and cisplatinum, *R-ICE* rituximab, ifosfamide, carboplatin, and etoposide, *BEAM* carmustine, etoposide, cytarabine, melphalan, *type-3* unclassified DLBCL-NOS, *MMAE* microtubule disrupting agent monomethyl auristatin E, *NF-κB* nuclear factor-kappa B, *IRF4* interferon-regulatory factor 4, *PKCβ* protein kinase Cβ, *CRBN* cereblon

Table S4

Ongoing experimental clinical studies combining novel experimental agents with conventional (immuno-) chemotherapy (R-CHOP, DHAP) in first-line treatment of newly diagnosed DLBCL

Treatment	Targets	Clinical trial	Study
Lenalidomide<br>+<br>DA-EPOCH-R	CRBN (IRF4)<br>CD20	Ongoing Phase I/II	Trial-No:<br>NCT02213913
Lenalidomide<br>+<br>Rituximab	CRBN (IRF4)<br>CD20	Ongoing phase II<br><br>Ongoing phase III	Trial-No:<br>NCT01856192<br>NCT02128061
Lenalidomide<br>+<br>R-ESHAP	CRBN (IRF4)<br>CD20	Ongoing phase I/II	Trial-No:<br>NCT02340936
Lenalidomide<br>+<br>R-CHOP	CRBN (IRF4)<br>CD20	Ongoing phase III	Trial-No:<br>NCT02285062
Ibrutinib<br>+<br>R-CHOP	BTK<br>CD20	Ongoing phase III	[205, 206]<br>Trial-No:<br>NCT01855750
Everolimus<br>(RAD001)<br>+<br>R-CHOP	mTORC1<br>CD20	Ongoing phase I	Trial-No:<br>NCT01334502
Carfilzomib<br>+<br>R-CHOP	Proteasome (NF-κB)<br>CD20	Ongoing Phase I/II	Trial-No:<br>NCT02073097
Bortezomib<br>+<br>R-CHOP	Proteasome (NF-κB)<br>CD20	Ongoing phase II	Trial-No:<br>NCT00931918
Bortezomib<br>+<br>R-CHOP	Proteasome (NF-κB)<br>CD20	Ongoing phase III	[206]<br>Trial-No:<br>NCT01324596
Vorinostat<br>+<br>R-CHOP	HDACs<br>CD20	Ongoing phase I/II	[198]<br>Trial-No:<br>NCT00972478
Panobinostat (LBH-589)<br>+<br>R-CHOP	HDACs<br>CD20	Ongoing phase I/II	[198]<br>Trial-No: NCT01238692
Azacytidine (AZA)<br>(CC-486)<br>+<br>R-CHOP	DMNTs<br>CD20	Ongoing phase I/II	[198]<br>Trial-No:<br>NCT01004991
Azacytidine (AZA)<br>(CC-486)<br>+<br>R-CHOP	DMNTs<br>CD20	Ongoing phase I/II	[198]<br>Trial-No:

DLBCL diffuse large B cell lymphoma, *R-CHOP* rituximab plus cyclophosphamide, doxorubicin, vincristine, and prednisone, *DA-EPOCH-R* etoposide, prednisone, vincristine, cyclophosphamide and doxorubicin plus rituximab, *R-ESHAP* etoposide, methylprednisolone, cytosine arabinoside, and

platinum, *IRF4* interferon response factor 4, *BTK* Brutons' tyrosine kinase, *mTORC1* mammalian target of rapamycin (mTOR) complex 1, *NF-κB* nuclear factor-kappa B, *HDAC* histone deacetylase, *DMNT* DNA methyltransferase, *CRBN* cereblon

Table S5:

Experimental clinical studies combining novel experimental agents with conventional (immuno-) chemotherapy (R-CHOP, DHAP) in second-line treatment of relapsed/refractory DLBCL

Treatment	Targets	Age	No. of patients	Response rate	Response duration	Clinical trials	Study
R-CHOP(-21)	CD20	61 (41-86)<br>61 (19-88)<br>62 (21-92)<br><br>70 (59-80)<br>70 (60-90) years	87<br>422<br>215<br><br>267<br>234	ORR: ≤ 90%<br>CR/CRu: ≤55 %<br>PR: ≤ 30%<br><br>ORR: ≤86 %<br>CR/CRu: ≤75 %<br>PR: ≤10 %	2y-PFS: ≤75%<br>2y-OS: ≤82%<br><br>3y-PFS: ≤62%<br>3y-F/EFS: ≤60%<br>3y-OS: ≤72%	Phase II<br>Phase III	Historical Control cohorts<br>[7, 207, 208]<br><br>[209, 210]
Ibrutinib<br>+<br>R-CHOP	BTK<br>CD20	61.5<br>(22-81)<br>years	23	ORR: 95%<br>CR: 72%<br>PR: 21%	NA	Phase Ib	[205]<br>Trial-No:<br>NCT01569750
Ibrutinib<br>+<br>Rituximab<br>+<br>Bendamustine	BTK<br>CD20	62<br>(23-84)<br>years		ORR: 37%<br>CR: 31%<br>PR: 6%	NA	Phase I/Ib	[211]<br>Trial-No:<br>NCT01479842
Lenalidomide<br>+<br>R-CHOP	CRBN (IRF4)<br>CD20	58<br>(24-77)<br>years	27	ORR: 96%<br>CR/CRu: 74%<br>PR: 22%	2y-PFS: 80%<br>2y-EFS: 70%<br>2y-OS: 90%	Phase Ib	[212]<br>Trial-No:<br>NCT00901615
Lenalidomide<br>+<br>R-CHOP21	CRBN (IRF4)<br>CD20	65<br>(22-87)<br>years	64	ORR: 98%<br>CR: 80%<br>PR: 18%	1y-E/PFS: 70%<br>2y-E/PFS: 59%<br>1y-OS: 90%<br>2y-OS: 78%	Phase II	[207]<br>Trial-No:<br>NCT00670358
Lenalidomide<br>+<br>R-CHOP21	CRBN (IRF4)<br>CD20	69<br>(64-71)<br>years	45	ORR: 92%<br>CR: 86%<br>PR: 6%	2y-PFS: 80%<br>2y-EFS: 70%<br>2y-OS: 90%	Phase II	[213]<br>Trial-No:<br>NCT00907348
Lenalidomide<br>+<br>R-ICE<br>(as a salvage regimen)	CRBN (IRF4)<br>CD20	61.5<br>(41-75)<br>years	15	ORR: 73%<br>CR: 60%<br>PR: 13%	NA	Phase I/II	[203]<br>Trial-No:<br>NCT01241734
CC-122<br>+<br>Obinutuzumab (GA101)	CRBN<br>(Aiolos, Ikaros, IRF4)<br>CD20	64<br>(estimated)	NA	NA	NA	Ongoing phase Ib	[56]<br>Trial-No:<br>NCT02417285
CC-122<br>+<br>Rituximab	CRBN<br>(Aiolos, Ikaros, IRF4)<br>CD20	NA	NA	NA	NA	Ongoing phase Ib	[56]<br>Trial-No:<br>NCT02031419
Epratuzumab<br>+<br>R-CHOP	CD22<br>CD20	58<br>(21-82)<br>years	107 (81)	ORR: 96%<br>CR/CRu: 74%<br>PR: 22%	2y-PFS: 77%<br>2y-EFS: 70%<br>2y-OS: 80%	Phase II	[7]<br>Trial-No:<br>NCT00301821
Epratuzumab<br>+<br>Rituximab	CD22<br>CD20	56<br>(24-78)<br>years	15	ORR: 47%<br>CR/CRu: 33%<br>PR: 22%	DOR: 6 m<br>1y-PFS: 13.5%	Phase II	[214]
Bortezomib	Proteasome	56	40 (35)	ORR: 88%	2y-PFS: 64%	Phase II	[215]

+<br>R-CHOP	(NF-κB)<br>CD20	(20-87)<br>years		CR/CRu: 75%<br>PR: 13%	2y-OS: 70%		Trial-No:<br>NCT00151320
Bortezomib<br>+<br>R-CHOP	Proteasome<br>(NF-κB)<br>CD20	50	NA	NA	NA	Phase II	Trial-No:<br>NCT00379574
Bortezomib<br>+<br>R-CHOP	Proteasome<br>(NF-κB)<br>CD20	170<br>(estimated)	NA	NA	NA	Phase II	Trial-No:<br>NCT01040871
Bortezomib<br>+<br>R-ICE	Proteasome<br>(NF-κB)<br>CD20	NA	NA	NA	NA	Ongoing<br>phase I	Trial-No:<br>NCT01226849
Bortezomib<br>+<br>DICE	Proteasome<br>(NF-κB)	NA	NA	NA	NA	Ongoing<br>phase II	Trial-No:<br>NCT00504751
Bortezomib<br>+<br>R-CHOP	Proteasome<br>(NF-κB)<br>CD20	NA	NA	NA	NA	Ongoing<br>phase IIII	Trial-No:<br>NCT01324596
Bortezomib<br>+<br>R-DHAP<br>With ASCT.	Proteasome<br>(NF-κB)<br>CD20	NA	NA	NA	NA	Ongoing<br>phase II/III	Trial-No:<br>NCT01805557
Carfilzomib<br>+<br>Rituximab<br>+<br>Ifosfamide/Ca<br>rboplatin/<br>Etoposide	Proteasome<br>(NF-κB)<br>CD20	NA	NA	NA	NA	Ongoing<br>Phase I/II	Trial-No:<br>NCT01959698
Everolimus<br>(RAD001)<br>+<br>Rituximab	mTORC1<br>CD20	65<br>(33-87)<br>years	24	ORR: 37.5 %<br>CR: 12.5%<br>PR: 25%<br>SD: 8%<br>PD: 54%	DOR: 8.1 m<br>1y-PFS: 22%<br>1y-OS: 37%	Phase II	[216]<br>Trial-No:<br>NCT00869999
Temsirolimus<br>+<br>R-DHAP	mTORC1<br>CD20	NA	NA	NA	NA	Ongoing<br>phase II	[71, 217, 218]<br>Trial-No:<br>NCT01653067<br>(STORM)
Vorinostat<br>+<br>R-CHOP	HDACs<br>(HDAC<br>(2)3/4)<br>CD20	NA	NA	NA	NA	Ongoing<br>Phase I/II	[198]<br>Trial-No:<br>NCT00667615
Vorinostat<br>+<br>High-Dose<br>Gemcitabine,<br>Busulfan,<br>Melphalan<br>with ASCT	HDACs<br>(HDAC<br>(2)3/4)	44<br>(12-65)<br>years	52 (28)	ORR: 96%<br>CR: 73%	2y-EFS: 61.5%<br>2y-OS: 73%	Phase I	[219]<br>Trial-No:<br>NCT01421173
Alemtuzumab<br>(anti CD52<br>antibody)	CD52<br>CD20	NA	NA	NA	NA	Ongoing<br>phase II	Trial-No:<br>NCT01030900

+ Rituximab + DA-EPOCH							
ABT-199 + Bendamustine + Rituximab	BCL2 CD20	NA	NA	NA	NA	Ongoing phase I/II	Trial-No: NCT01594229
CPI-613 (novel tumor-specific anti-mitochondrial agent) + Bendamustine + Rituximab	Mitochondrial metabolism <br>CD20	NA	NA	NA	NA	Ongoing phase I	[220] Trial-No: NCT02168907

DLBCL diffuse large B cell lymphoma, *ABC* activated B cell-like, *GCB* germinal center B cell-like, *ORR* overall response rate, *PR* partial response, *CR* complete response, *CRu* unconfirmed CR, *SD* stable disease, *PD* progressive disease, *DOR* median duration of response, *OS* median overall survival, *PFS* median progression-free survival, *EFS* median event-free survival, *FFS* median failure-free survival; *NA* not available/not applicable, *m* months, *y* year, *ASCT* autologous stem-cell transplantation, *R-CHOP* rituximab plus cyclophosphamide, doxorubicin, vincristine, and prednisone, *DHAP* dexamethasone, cytarabine, and cisplatinum, *DA-EPOCH-R* etoposide, prednisone, vincristine, cyclophosphamide and doxorubicin plus rituximab, *R-ICE* rituximab, ifosfamide, carboplatin and etoposide, *D-ICE* dexamethasone, ifosfamide, carboplatin, etoposide, *IRF4* interferon response factor 4, *BTK* Brutons' tyrosine kinase, *NF-κB* nuclear factor-kappa B, *mTORC1* mammalian target of rapamycin (mTOR) complex 1, *HDAC* histone deacetylase, *DMNT* DNA methyltransferase, *CRBN* cereblon

Table S6

Ongoing clinical experimental studies with JAK/STAT inhibitors in aggressive B-cell lymphoma including DLBCL and relapsed/refractory DLBCL

Treatment	Targets	Patient characteristics	Relapsed/ refractory DLBCL	Clinical trial	Study
Pacritinib (SB1518) Oral JAK2/ FLT3 inhibitor	JAK2/JAK2(V617F FLT3 kinase (STAT1, STAT3, STAT5)	Patient No.: 5 Age: 18 to 65 y;	No	Completed phase I	[221] Trial-No: NCT00741871
INCB039110 Oral JAK1 inhibitor	JAK1 (STAT1, STAT3)	Patient No.: NA Age: 18 -	Yes	Ongoing phase I	Trial-No: NCT01905813
Ruxolitinib (INCB18424) Oral JAK1/JAK2 inhibitor	JAK1/JAK2 (STAT1, STAT3)	Patient No.: NA Age: 19-	Yes	Ongoing phase II	[222] Trial-No: NCT01431209

DLBCL diffuse large B cell lymphoma, *JAK* Janus kinase, *STAT* signal transducer and activator of transcription, *FLT3* fms-related tyrosine kinase 3

Table S7: Experimental clinical studies evaluating antibody-drug conjugates (ACD) as front-line or second-line treatment in DLBCL (as single agent or in combination)

Treatment	Type of Treatment	Age	No. of DLBCL patients	Response rate	Response duration	Clinical trial	Study
Brentuximab Vedotin	Second-line	62 (17-85) years	50 (49)	ORR: 44%<br>CR: 17%<br>PR: 27%<br>SD: 23%<br>PD: 33%	DOR: 5.8m<br>DOR (CR): 16.6m<br>PFS: 4m<br>OS: NA	Completed phase II	[18, 223]<br>Trial-No: NCT01421667
Brentuximab Vedotin<br>+<br>Rituximab	Second-line	62 (22-78) years	16	ORR: 46%<br>CR: 15%<br>PR: 31%	NA	Completed phase II	[18, 223]<br>Trial-No: NCT01421667
Brentuximab Vedotin<br>+/-<br>Lenalidomide	Second-line	NA	NA	NA	NA	Ongoing Phase 1	Trial-No: NCT02086604
Brentuximab-Vedotin<br>+/-<br>R-CHOP or R-CHP	Front-line	NA	NA	NA	NA	Ongoing phase II	Trial-No: NCT01925612
Inotuzumab ozogamicin (INO)<br>+<br>Rituximab	Second-line	72 (33-85) years	67	ORR: 74%<br>CR: 50%<br>PR: 25%	Relapsed:<br>1y-PFS: 55%<br>2y-PFS: 42%<br>1y-OS: 80%<br>2y-OS: 69%<br>Refractory:<br>2y-PFS: 10%	Completed phase I/II	[20]<br>Trial-No: NCT00299494
Inotuzumab Ozogamicin (CMC-544)<br>+/-<br>Rituximab	Second-line	60 (19-75) years	63	ORR: 40%<br>CR: 21%<br>PR: 26%	1y-PFS: 28.9%<br>2y-PFS: 25.3%	Completed phase II	[224]<br>Trial-No: NCT00867087
Inotuzumab Ozogamicin (CMC-544)<br>+/-<br>R-GEMOX	Second-line	NA	NA	NA	NA	Ongoing phaseI/II	Trial-No: NCT01562990
Inotuzumab Ozogamicin<br>+/-<br>R-CVP	Front-line	NA	154 (estimated)	NA	NA	Ongoing phase II	Trial-No: NCT01679119
Inotuzumab ozogamicin (INO)<br>+<br>Rituximab	Second-line	NA	NA	NA	NA	Ongoing phase II	Trial-No: EudraCT 2008-007802-12
SAR3419	Second-line	66 (41-80) years	5 (39)	Reduced tumors: 76%	NA	Completed Phase I	[11]<br>Trial-No: NCT00549185
SAR3419	Second-line	67 (36-82)<br>70 (37-85)	25<br>16 (week.)<br>9 (optim.)	ORR: 30%<br>Reduced tumors: 64%	8 weeks (NA)	Completed Phase I	[12]

		years					
SAR3419<br>+/-<br>Rituximab	Second-line	NA	50	NA	NA	Completed Phase II	Trial-No: NCT01470456
SAR3419	Second-line	NA	55 (estimated)	NA	NA	Ongoing Phase II	[12]<br>Trial-No: NCT01472887
SGN-CD19A	Second-line	NA	45	ORR: 35%<br>CR: 20%<br>PR: 16%<br><br>Relapsed<br>ORR: 55%<br>CR: 35%<br>PR: 23%<br><br>(preliminary data)	NA	Ongoing Phase I	[225, 226]<br>Trial-No: NCT01786135
MEDI-551 (anti CD19 ADC)<br>+<br>Rituximab<br>+<br>ICE or DHAP	Second-line	NA	NA	NA	NA	Ongoing phase II	Trial-No: NCT01453205
Pinatuzumab Vedotin (DCDT2980S)<br>+<br>Rituximab or Obinutuzumab	Second-line	NA	28	ORR: 43%<br>CR: 18%<br>PR: 25%<br>SD: 14%<br>PD: 43%	DOR: 6m	Completed phase I	[15, 227]<br>Trial-No: NCT01209130 (ROMULUS)
Moxetumomab Pasudotox (HA22, anti CD22 ADC)	Second-line	NA	87 (estimated)	NA	NA	Completed Phase I/II	Trial-No: NCT01030536
Polatuzumab Vedotin (DCDS4501A)<br>+<br>Rituximab	Second-line	67 (20–81) years	27	ORR: 56%<br>CR: 14.8%<br>PR: 37%<br>SD: 37%<br>PD: 14.8%	DOR (CR): 5.2 m<br>DOR (PFS): 5 m	Completed phase I	[15, 227, 228]<br>Trial-No: NCT01290549 (ROMULUS)
Polatuzumab Vedotin (DCDS4501A)<br>+<br>Rituximab<br>+<br>Cyclophosphamide, Doxorubicin and Prednisone	First-line	NA	NA	NA	NA	Ongoing phase I	[228]<br>Trial-No: NCT01992653
Polatuzumab Vedotin (DCDS4501A)<br>+<br>Rituximab	Second-line	NA	NA	NA	NA	Ongoing phase II	Trial-No: NCT01691898
Polatuzumab Vedotin (DCDS4501A)	Second-line	NA	NA	NA	NA	Ongoing phase II	Trial-No: NCT02257567

+<br>Rituximab or Obinutuzumab<br>+<br>Bendamustine							

DLBCL diffuse large B cell lymphoma, *ORR* overall response rate, *PR* partial response, *CR* complete response, *CRu* unconfirmed CR, *SD* stable disease, *PD* progressive disease, *DOR* median duration of response, *OS* median overall survival, *PFS* median progression-free survival, *EFS* median event-free survival, *FFS* median failure-free survival, *NA* not available/not applicable, *m* months, *y* year, *ASCT* autologous stem-cell transplantation, *R-CHOP* rituximab plus cyclophosphamide, doxorubicin, vincristine, and prednisone, DHAP dexamethasone, cytarabine, and cisplatinum, *R-GEMOX* rituximab, gemcitabine and oxaliplatin.

Brentuximab-Vedotin is an affinity-optimized monoclonal anti-CD30 antibody linked to the microtubule disrupting agent monomethyl auristatin E (MMAE). Vedotin is the commercial name for the microtubule disrupting agent monomethyl auristatin E (MMAE), Inotuzumab ozogamicin is an affinity-optimized monoclonal anti-CD22 antibody linked to the DNA damaging toxin N-acetyl-γ-calicheamicin dimethyl hydrazide (CalichDMH). Moxetumomab Pasudotox (HA22/ BL22) is an affinity-optimized monoclonal anti-CD22 antibody linked to a mono-ADP-ribosylating Pseudomonas Exotoxin. SAR3419 is an affinity-optimized monoclonal anti-CD19 antibody linked to the cytotoxine Maytansine. SGN-CD19A is an affinity-optimized monoclonal anti-CD19 antibody linked to the microtubule disrupting agent monomethyl auristatin E (MMAE). MEDI-551 is an affinity-optimized and afucosylated monoclonal anti CD19 antibody with enhanced antibody-dependent cellular cytotoxicity. Pinatuzumab-Vedotin is an affinity-optimized monoclonal anti-CD22 antibody linked to the microtubule disrupting agent monomethyl auristatin E (MMAE). Polatuzumab Vedotin is an affinity-optimized monoclonal anti-CD79B antibody linked to the microtubule disrupting agent monomethyl auristatin E (MMAE).

Table S8:

Experimental clinical studies evaluating anti-CD19 or anti-CD20 Chimeric Antigen Receptor T cells as second-line treatment in relapsed/refractory DLBCL

Inhibitor or Agent	Target or Agent mechanism	No. of patients	Response rate (DLBCL Subtype)	Response duration	Clinical trials	Study
Anti-CD19 CAR T cells	CD19 (T cells expressing anti-CD19 CARs recognize and kill CD19+ target cells)	7	CR/CRu: 57.1%<br>PR: 28.5%<br>SD: 14.3%<br>(preliminary data)	Not yet available	Ongoing phase I	[5, 6]<br>Trial-No: NCT00924326
Anti-CD19 CAR T cells	CD19+ target cells	12	ORR: 50%<br>CR/Cru: 45%<br>(preliminary data)	6m-PFS: 37% (preliminary data)	Ongoing phase II	[229]<br>Trial-No: NCT02030834
Anti-CD19 CAR T cells	CD19+ target cells	NA	NA	NA	Ongoing phase II	Trial-No: NCT02445248
Anti-CD19 CAR T cells	CD19+ target cells	NA	NA	NA	Ongoing phase I	Trial-No: NCT01840566
Anti-CD19 CAR T cells	CD19+ target cells	NA	NA	NA	Ongoing phase I/II	Trial-No: NCT01865617
Anti-CD19 CAR T cells	CD19+ target cells	NA	NA	NA	Ongoing phase I/II	Trial-No: NCT01475058
Anti-CD19 CAR T cells	CD19+ target cells	NA	NA	NA	Ongoing phase I/II	Trial-No: NCT02431988
Anti-CD19 CAR T cells	CD19+ target cells	NA	NA	NA	Ongoing phase I/II	Trial-No: NCT02348216
Anti-CD20 CAR T cells	CD20 (T cells expressing anti-CD19 CARs recognize and kill CD20+ target cells)	NA	NA	NA	Ongoing pilot study	[230]<br>Trial-No: NCT01735604

DLBCL diffuse large B cell lymphoma, *ORR* overall response rate, *PR* partial response, *CR* complete response, *CRu* unconfirmed CR, *SD* stable disease, *PFS* median progression-free survival, *NA* not available, *m* month,

Table S9

Ongoing clinical experimental studies with ARTD/PARP inhibitors in aggressive B-cell lymphoma including DLBCL and relapsed/refractory DLBCL

**Treatment**	**Targets**	**Patient characteristics**	**Relapsed/ refractory DLBCL**	**Clinical trial**	**Study**
Veliparib (ABT-888) + Cyclophosphamide	ARTD1-3 (PIM1?, CDK9?)	Patient No.: NA Age: 18 -	Yes	Ongoing phase I/II	Trial-No: NCT00810966
Veliparib (ABT-888) + Bendamustine + Rituximab	ARTD1-3 (PIM1?, CDK9?) CD20	Patient No.: NA Age: 18 -	Yes	Ongoing phase I/II	Trial-No: NCT01326702
Veliparib (ABT-888) + Cyclophosphamide + Doxorubicin	ARTD1-3 (PIM1?, CDK9?)	Patient No.: NA Age: 18 -	Yes	Ongoing phase I	Trial-No: NCT00740805

DLBCL diffuse large B cell lymphoma, *ARTD* ADP-ribosyltransferase diphteria toxin like, *PARP* poly-ADP-ribose-polymerase, *PIM1* (Proviral Integration site for the Moloney murine leukemia virus) kinase 1, *CDK9* cyclin-dependent kinase 9

Table S10

Pre-clinical multi-targeted combinatorial experimental treatments for DLBCL-NOS

Treatment Combination	Targets	DLBCL subtype(s) where effect was observed	Effects in refractory DLBCL *in vitro* or *in vivo*?	*In vivo* effects in xenograft or B-cell lymphoma mice models	Study
JAKi1/2<br>+<br>MLN120B	STAT3<br>IKKβ/<br>NF-κB	ABC,<br>(n.c.i.)	NA	NA	[28]
Bortezomib/<br>carfilzomib<br>+<br>ABT737/<br>Obatoclax	Proteasome<br>(NF-κB)<br>BCL2	GCB, ABC	Yes;<br>carfilzomib/obatoclax regimen potently induced apoptosis in bortezomib-resistant GCB and ABC cell lines *in vitro*	Obatoclax strongly potentiated carfilzomib-induced growth suppression of GCB tumors in xenograft models	[26, 78]
Bortezomib<br>+<br>Thiostrepton	Proteasome<br>(NF-κB)<br>FoxM1	GCB,<br>(n.c.i.)	NA	NA	[174]
Ibrutinib<br>+<br>Bortezomib/<br>Carfilzomib	BTK<br>Proteasome<br>(NF-κB)	GCB, ABC<br>(BCR signature)	Yes;<br>Ibrutinib/borte-zomib regimen potently induced apoptosis in highly bortezomib-resistant GCB and ABC cell lines *in vitro*	NA	[27, 68]
Ibrutinib<br>+<br>GDC-0980/<br>Everolimus	BTK<br>mTORC1	ABC,<br>(BCR signature)	NA	NA	[68]
Ibrutinib<br>+<br>Idelalisib (GS-1101, CAL-101)/ BKM-120	BTK<br>PI3K isoforms	ABC,<br>(BCR signature)	NA	NA	[68]
Ibrutinib<br>+<br>MK-2206	BTK<br>AKT	ABC,<br>(BCR signature)	NA	NA	[68]
Ibrutinib<br>+<br>PRT-060318	BTK<br>SYK	ABC,<br>(BCR signature)	NA	NA	[68]
Ibrutinib<br>+<br>Navitoclax (ABT-263)/ ABT-199	BTK<br>BCL2	ABC,<br>(BCR signature)	NA	NA	[68]
Ibrutinib<br>+<br>Lenalidomide	BTK<br>IRF4	ABC,<br>(BCR signature)	NA	Lenalidomide synergized strongly with ibrutinib in suppression of the growth of ABC tumors in xenograft models	[52]
Ibrutinib<br>+	BTK<br>mTORC1/2	ABC,<br>(BCR	NA	Ibrutinib synergized strongly with AZD2014 in tumor	[68, 73]

AZD2014		signature)		regression of ABC tumors in xenograft models	
Ibrutinib<br>+<br>NVP-BEZ-235	BTK<br>mTORC1/2<br>PI3K,<br>(DNA-PK)	ABC,<br>(BCR signature)	NA	NA	[68]
Ibrutinib<br>+<br>CPI203 and JQ1	BTK<br>NF-κB<br>BRD2/4<br>(c-Myc?)	ABC,<br>(BCR signature)	NA	Ibrutinib, synergized strongly with BET inhibitors in killing ABC tumors in xenograft models	[122]
Ibrutinib<br>+<br>SP600125	BTK<br>JNKs	ABC,<br>(BCR signature?)<br>(n.c.i.)	NA	NA	[155]
Ibrutinib<br>+<br>OTX015	BTK<br>NF-κB<br>BRD2/4<br>(c-Myc?)	ABC,<br>(BCR signature)	NA	NA	[130]
OTX015<br>+<br>Rituximab,	BRD2/4<br>(c-Myc?)<br>CD20	ABC, GCB, type-3<br>(strongest effect in ABC)<br>(n.c.i.)	NA	NA	[130]
OTX015<br>+<br>lenalidomide	BRD2/4<br>(c-Myc?)<br>CRBN<br>(IRF4)	ABC, GCB, type-3<br>(strongest effect in ABC)<br>(n.c.i.)	NA	NA	[130]
OTX015<br>+<br>Idelalisib	BRD2/4<br>(c-Myc?)<br>PI3Kδ	ABC, GCB, type-3<br>(strongest effect in ABC)<br>(n.c.i.)	NA	NA	[130]
OTX015<br>+<br>Everolimus	BRD2/4<br>(c-Myc?)<br>mTORC1	ABC, GCB, type-3<br>(n.c.i.)	NA	NA	[130]
OTX015<br>+<br>Decitabine	BRD2/4<br>(c-Myc?)<br>DMNTs	ABC, GCB, type-3<br>(n.c.i.)	NA	NA	[130]
OTX015<br>+<br>Vorinostat	BRD2/4<br>(c-Myc?)<br>HDACs	ABC, GCB, type-3<br>(n.c.i.)	NA	NA	[130]
U73122<br>+<br>Enzastaurin/pp2	PLCγ2<br>PKCβ/SRC	ABC, GCB<br>(n.c.i.)	NA	NA	[64]
Ricolinostat (ACY-1215)<br>+<br>Carfilzomib (CFZ)	HDAC6<br>Proteasome<br>(NF-κB)	GCB, ABC<br>(n.c.i.)	NA	NA	[146]
Ricolinostat (ACY-1215)	HDAC6<br>Proteasome	ABC, GCB<br>(n.c.i.)	NA	Combination treatment demonstrated significant	[231]

+ Bortezomib	(NF-κB)			tumor growth delay and prolonged overall survival in xenograft mouse model of DLBCL	
Vorinostat + Carfilzomib (CFZ)	HDACs Proteasome (NF-κB)	GCB, ABC (n.c.i.)	Yes; Vorinostat / CFZ regimen potently induced apoptosis in partially bortezomib-resistant GCB and ABC cell lines *in vitro*	Subeffective vorinostat doses synergized strongly with CFZ in tumor growth suppression and apoptosis of ABC tumors in xenograft models	[25]
Vorinostat + Niacinamide	HDACs Sirtuins	ABC, GCB, type3 (n.c.i.)	Niacinamide /Vorinostat combination showed some effects in patients with relapsed or refractory DLBCL in a phase 1 proof-of-principle clinical trial	Niacinamide /Vorinostat combination treatment synergistically produced remissions in a spontaneous aggressive B-cell lymphoma mouse model expressing Bcl6	[145]
Vorinostat + ABT-737	HDACs BCL2	GCB, (n.c.i.)	NA	NA	[79]
Vorinostat + Enzastaurin	HDACs PKCβ	GCB, ABC (n.c.i.)	NA	NA	[232]
Vorinostat / Panobinostat (LBH-589) + Decitabine	HDACs DMNTs	GCB (strong effects) ABC (weak effects) (n.c.i.)	NA	Vorinostat/ Decitabine combination treatment synergistically inhibited the growth of GCB tumors in xenograft models	[233]
Vorinostat / Panobinostat (LBH-589) + JQ1/ RVX2135	HDACs BRD2-4	NA	NA	Vorinostat / RVX2135 combination treatment synergistically produced remissions in a murine model of aggressive MYC driven B Cell lymphoma	[234]
Panobinostat (LBH-589) + Rapamycin	HDACs (HDAC3/4) mTORC1	GCB, (n.c.i.)	NA	NA	[74]
Panobinostat (LBH-589) + NVP-BEZ235	HDACs (HDAC3/4) mTORC1/2 PI3K (DNA-PK)	ABC, GCB, type3 (incl. BCL/ c-MYC double-hit DLBCL) (n.c.i.)	NA	BEZ235/panobinostat combination treatment synergistically inhibited the growth of GCB tumors in xenograft models and combined treatment increased animal survival compared with single agents.	[235]
ABT-263 + Rapamycin	BCL2 mTORC1	GCB, (n.c.i.)	NA	ABT-263/ Rapamycin combination treatment synergistically induced tumor regression, with GCB tumors	[236]

				showing 100% ORR in xenograft models	
ABT-737<br>+<br>Cyclopamine-KAAD	BCL2<br>SMO	GCB,<br>(ABC? n.c.i.)	NA	NA	[237]
ABT-199<br>+<br>Dinaciclib	BCL2<br><br>CDK1,<br>CDK2,<br>CDK5,<br>CDK9,<br>(MCL1)	GCB,<br>(ABC? n.c.i.)	NA	ABT-199/ dinaciclib combination treatment synergistically induced tumor regression, in xenografts and in a genetically accurate murine model of MYC-BCL2 double-hit lymphoma	[89]
AG490 or<br>STA-21<br>+<br>YM155	STAT3 (?)<br>(STAT1?)<br>Survivin	GCB,<br>(n.c.i.)	NA	NA	[91]
YM155<br>+<br>Rituximab	Survivin<br>CD20	ABC, GCB,<br>(n.c.i.)	NA	YM155/<br>Rituximab combination treatment synergistically induced tumor growth inhibition and tumor regression in GCB and ABC tumors in xenograft models	[93]
CGS-21680<br>(AdR agonist)<br>+<br>Trequinsin	Adenosine A2A receptor,<br><br>Phosphodi-esterases (PDE) 2-4	ABC, GCB,<br>type3<br>(n.c.i.)	NA	NA	[178]
CGS-21680 / Salmeterol (AdR agonists)<br>+<br>Dexamethasone (GR agonist)	Adenosine A2A receptor,<br><br>glucocorticoid receptor (GR)	ABC, GCB,<br>type3<br>(n.c.i.)	NA	NA	[238]
Cyclopamine-KAAD<br>+<br>BAY-11-7082	SMO<br>NF-κB	GCB<br>(ABC? n.c.i.)	NA	NA	[29]
ERK inhibitor 76<br>+<br>CHK2 inhibitor II	ERK1/2<br>CHK2	ABC, GCB,<br>type3<br>(n.c.i.)	NA	Concurrent administration of ERK inhibitor 76 enhanced the antitumour activity of CHK2 inhibitor II in GCB tumors in xenograft models	[149]
Rapamycin<br>+<br>UCN-01	mTORC1<br>CDK1	GCB<br>(n.c.i.)	NA	NA	[158]
Rapamycin<br>+	mTORC1<br>AKT	GCB<br>(n.c.i.)	Yes, rapamycin showed synergistic effects with	NA	[69]

Nelfinavir /MK-2206			nelfinavir or MK-2206 in inhibiting cell viability in rapamycin-resistant GCB cell lines		
Everolimus (RAD001) + NVP-BEZ235	mTORC1/2 PI3K (DNA-PK)	GCB (strong effects) ABC (weak effects) (n.c.i.)	NA	NA	[239]
Temsirolimus + Idelalisib	mTORC1 PI3K	GCB, ABC, (additive effects) (n.c.i.)	NA	NA	[240]
Temsirolimus + Ibrutinib	mTORC1 BTK	ABC, (additive effects) (n.c.i.)	NA	NA	[240]
Temsirolimus + Bortezomib	mTORC1 NF-κB	ABC (additive effects) (antagonistic effects in some GCB cells) (n.c.i.)	NA	NA	[240]
NVP-BKM120 + chloroquine/3-methyladenine/Bafilomycin A 1	PI3K Autophagy	GCB, ABC (n.c.i.)	NA	NA	[65]
Bortezomib + chloroquine	Proteasome (NF-κB) Autophagy	ABC, GCB (n.c.i.)	NA	NA	[241]
RI-BPI + SAHA, TSA, or VPA	BCL6 HDACs	GCB, ABC (n.c.i.)	NA	SAHA strongly potentiated RI-BPI mediated growth suppression of GCB tumors in xenograft models	[97]
RI-BPI + 17-DMAG, PU-H71	BCL6 HSP90	GCB, ABC (n.c.i.)	NA	PU-H71 strongly potentiated RI-BPI mediated growth suppression of GCB tumors in xenograft models	[97]
BL22 (anti-CD22 immunotoxin) + Bryostatin 1	CD22 PKCβII	GCB, ABC? (n.c.i.)	NA	NA	[21]

DLBCL diffuse large B cell lymphoma, *ABC* activated B cell-like, *GCB* germinal center B cell-like, *OxPhos* oxidative phosphorylation subtype, *BCR* BCR-activated B-cell (antigen) receptor/proliferation subtype**,** *HR* host response signature subtype, *MD* c-MYC-driven signature subtype, *n.c.i.* not completely investigated, *NA* not available/not analyzed, *type-3* unclassified DLBCL-NOS, *NF-κB* nuclear factor-

kappa B, *STAT* signal transducer and activator of transcription, *IRF4* interferon response factor 4, *IKK* inhibitor of kappa B kinase, *BRD* BET bromodomain protein, *HDACs* histone deacetylases, *DMNT* DNA methyltransferase, *BTK* Brutons' tyrosine kinase, *SMO* smoothened, frizzled class receptor, *CDK* cyclin-dependent kinase, *MCL1* myeloid cell leukemia 1, *mTORC1* mammalian target of rapamycin (mTOR) complex 1, *PI3K* phosphoinositide 3-kinase, *BCL* B-cell lymphoma protein, *HSP* heat shock protein, *FoxM1* forkhead box M1, *CHK1/2* Chk1 protein kinase 1/2, *PKCβ* protein kinase Cβ, *CRBN* cereblon

Table S11: Dual inhibitors currently evaluated in preclinical and/or clinical studies in DLBCL

Dual Inhibitor	Targets	Manufacturer	DLBCL subtype(s) where effect was observed *in vitro* or *in vivo*	*In vivo* effects in xenograft or B-cell lymphoma mice models	Clinical trial	Study
OSI-027	mTORC1/2	OSI Pharmaceuticals	(n.c.i.)	OSI-027 markedly diminished proliferation and induced apoptosis in a variety of lymphoid cell lines and induced tumor regressions in B-cell lymphoma xenograft models	Completed phase I	[242] Trial-No: NCT00698243
CC-223	mTORC1/2	Celgene Corporation	ABC, GCB, type3 (n.c.i.)	NA	Ongoing phase I Ongoing phase I/II	Trial-No: NCT02031419 NCT01177397 [75]
AZD2014	mTORC1/2	AstraZeneca	ABC, (n.c.i.)	AZD2014 treatment inhibited the growth of ABC tumors in xenograft models and increased animal survival.		[73]
NVP-BEZ235	mTORC1/2 PI3Ks (DNA-PK)	Novartis	ABC, GCB, type3 (incl. double-hit DLBCLs) (n.c.i.)	BEZ235 treatment inhibited the growth of GCB tumors in xenograft models and increased animal survival.		[68, 235, 239, 243]
PRT062070 (Cerdulatinib)	SYK JAK1/3	Portola Pharmaceuticals	GCB, (n.c.i.)	NA	Ongoing phase I	[244] Trial-No: NCT01994382
QL-X-138	BTK MNK		ABC, (n.c.i.)	NA		[245]

DLBCL diffuse large B-cell lymphoma, *ABC* activated B cell-like, *GCB* germinal center B cell-like, *type-3* unclassified DLBCL-NOS, *mTORC1/2* mammalian target of rapamycin (mTOR) complex 1/2, *PI3K* phosphoinositide 3-kinase, *DNA-PK* DNA dependent protein kinase, *SYK* spleen tyrosine kinase, *JAK* Janus kinase, *BTK* Brutons' tyrosine kinase, *MNK* mitogen-activated protein kinase interacting kinase 1 and 2 (MNK1/2), *n.c.i.* not completely investigated, *NA* not available/not analyzed

Table S12

Ongoing experimental clinical multi-targeted studies combining novel experimental agents with or without conventional (immuno-)chemotherapy in first-line treatment of newly diagnosed DLBCL

**Treatment**	**Targets**	**Clinical trial**	**Study**
Sotrastaurin (AEB071)<br>+<br>Everolimus (RAD001)<br>(in patients with CD79-mutant or ABC DLBCL)	PKCβ<br>mTORC1	Ongoing phase Ib/II	Trial-No:<br>NCT01854606

DLBCL diffuse large B-cell lymphoma, *ABC* activated B cell-like, *GCB* germinal center B cell-like, *PKCβ* protein kinase Cβ, *mTORC1/2* mammalian target of rapamycin (mTOR) complex 1/2,

Table S13

Completed experimental clinical multi-targeted studies combining novel experimental agents in relapsed/refractory DLBCL

Inhibitor or Agent	Target or Agent mechanism	No. of patients	Response rate (DLBCL Subtype)	Response duration	Clinical trials	Study
Temsirolimus<br>+<br>Bortezomib	mTORC1<br>Proteasome<br>(NF-κB)	18	ORR: 16.7%<br>CR: 11.1 %<br>PR: 5.5%	DOR: 11.3 m<br>PFS: 2 m	Phase II	[246]<br>Trial-No:<br>NCT01281917
Decitabine<br>+<br>Vorinostat	DNMT1-3<br>HDACs	NA	NA	NA	Completed phase I	Trial-No:<br>NCT00275080
Decitabine<br>(DAC)<br>+/-<br>Valproic acid	DNMT1-3<br>HDACs<br>(HDAC (2)3/4)	NA	NA	NA	Completed phase I	Trial-No:<br>NCT00109824

DLBCL diffuse large B-cell lymphoma, *ORR* overall response rate, *PR* partial response, *CR* complete response, *DOR* median duration of response, *OS* median overall survival, *PFS* median progression-free survival, *NA* not available/not applicable, *m* months, *mTORC1* mammalian target of rapamycin (mTOR) complex 1, *HDACs* histone deacetylases, *DNMTs* DNA methyltransferases, *NF-κB* nuclear factor-kappa B.

Table S14

Ongoing experimental clinical multi-targeted studies combining novel experimental agents with or without conventional (immuno-)chemotherapy in relapsed/refractory DLBCL

**Treatment**	**Targets**	**Results**	**Clinical trial**	**Study**
Decitabine<br>+<br>Vorinostat	DNMT1-3<br>HDACs	NA	Completed phase I	Trial-No:<br>NCT00275080
Decitabine (DAC)<br>+/-<br>Valproic acid	DNMT1-3<br>HDACs<br>(HDAC (2)3/4)	NA	Completed phase I	Trial-No:<br>NCT00109824
Idelalisib<br>+<br>ONO/GS-4059	PI3Kδ<br>BTK		Ongoing phase I	Trial-No:<br>NCT02457598
Lenalidomide<br>+<br>Ibrutinib	CRBN (IRF4)<br>BTK	NA	Ongoing phase I	Trial-No:<br>NCT01955499
Lenalidomide + Ibrutinib<br>+/-<br>Rituximab	CRBN (IRF4)<br>BTK<br>CD20	NA	Ongoing phase I/II	Trial-No:<br>NCT02077166
Lenalidomide + Ibrutinib<br>+<br>DA-EPOCH-R	CRBN (IRF4)<br>BTK<br>CD20	NA	Ongoing phase I/II	Trial-No:<br>NCT02142049
Lenalidomide<br>+<br>Everolimus (RAD001)	CRBN (IRF4)<br>mTORC1	NA	Ongoing phase I/II	Trial-No:<br>NCT01075321
Lenalidomide<br>+<br>Obinutuzumab (GA101)	CRBN (IRF4)<br>CD20	NA	Ongoing phase Ib/II	Trial-No:<br>NCT01582776
CC-122 + CC-223<br>+/-<br>Rituximab	CRBN (Aiolos, Ikaros, IRF4)<br>mTORC1/mTORC2<br>CD20	NA	Ongoing phase Ib	[56]<br>Trial-No:<br>NCT02031419
CC-122 + CC293<br>+/-<br>Rituximab	CRBN (Aiolos, Ikaros, IRF4)<br>BTK<br>CD20	NA	Ongoing phase Ib	[56]<br>Trial-No:<br>NCT02031419
CC-223 + CC293<br>+/-<br>Rituximab	mTORC1/mTORC2<br>BTK<br>CD20	NA	Ongoing phase Ib	[56]<br>Trial-No:<br>NCT02031419
Panobinostat (LBH-589)<br>+<br>Rituximab	HDACs<br>CD20	NA	Ongoing Phase I/II<br>Ongoing Phase II	[198]<br>Trial-No:<br>NCT01238692,<br>NCT01282476
Vorinostat<br>+<br>Azacytidine (AZA)	HDACs<br>(HDAC (2)3/4)<br>DNMT1/2	NA	Ongoing Phase I/II	[198]<br>Trial-No:<br>NCT01120834
Vorinostat<br>+<br>Bortezomib	HDACs<br>(HDAC (2)3/4)<br>Proteasome (NF-κB)	NA	Ongoing Phase II	[198]<br>Trial-No:<br>NCT00703664
Vorinostat<br>+<br>Carfilzomib	HDACs<br>(HDAC (2)3/4)<br>Proteasome (NF-κB)	NA	Ongoing Phase I	Trial-No:<br>NCT01276717

Belinostat (PXD-101)<br>+<br>Carfilzomib	HDACs<br>Proteasome (NF-κB)	NA	Ongoing Phase I	Trial-No:<br>NCT02142530
Idelalisib (GS-1101)<br>+<br>Entospletinib (GS-9973)	PI3K<br>SYK	NA	Ongoing Phase II	[247]<br>Trial-No:<br>NCT01796470
INCB040093<br>(PI3Kδ inhibitor)<br>+<br>INCB039110<br>(JAK1 inhibitor)	PI3K<br>JAK1<br>(STAT1, STAT3)	NA	Ongoing Phase I	Trial-No:<br>NCT01905813
Lenalidomide + Dexamethasone<br>+<br>Rituximab	CRBN (IRF4)<br>CD20<br>GR	NA	Ongoing phase I/II	[248]
Nivolumab<br>+<br>Ipilimumab	PD-1<br>CTLA-4	NA	Ongoing phase I	Trial-No:<br>NCT01592370
Nivolumab<br>+<br>Lirilumab	PD-1<br>KIR	NA	Ongoing phase I	Trial-No:<br>NCT01592370
MPDL3280A<br>(anti-PD-L1 antibody)<br>+<br>Obinutuzumab (GA101)	PD-1<br>CD20<br>(Obinutuzumab is a novel humanized Type II anti-CD20 monoclonal antibody)	NA	Ongoing phase I	Trial-No:<br>NCT02220842
MEDI4736<br>(anti-PD-L1 antibody)<br>+<br>Ibrutinib	PD-1<br>BTK	NA	Ongoing phase I/II	Trial-No:<br>NCT02401048
Veltuzumab<br>+<br>90Y-Epratuzumab- Tetraxetan<br>(Radioimmunotherapy)	CD20<br>CD22<br>+<br>Radioimmunotherapy<br>(radiolabeled anti CD22 antibody, 90Y-epratuzumab tetraxetan)	NA	Ongoing phase II	[204]<br>Trial-No:<br>NCT01101581

DLBCL diffuse large B-cell lymphoma, *DA-EPOCH-R* etoposide, prednisone, vincristine, cyclophosphamide and doxorubicin plus rituximab, *IRF4* interferon-regulatory factor 4, *mTORC1* mammalian target of rapamycin (mTOR) complex 1, *BTK* Bruton's tyrosine kinase, *HDACs* histone deacetylases, *DNMTs* DNA methyltransferases, *NF-κB* nuclear factor-kappa B, *PI3K* phosphoinositide 3-kinase, *SYK* spleen tyrosine kinase, *JAK* Janus kinase, *STAT* signal transducer and activator of transcription, *GR* glucocorticoid receptor, *PD-1* programmed cell death 1, *CTLA4* cytotoxic T-lymphocyte-associated protein 4, *KIR* inwardly rectifying potassium channel, *CRBN* cereblon

References:

1. Armand P, Nagler A, Weller EA, Devine SM, Avigan DE, Chen YB, Kaminski MS, Holland HK, Winter JN, Mason JR *et al*: Disabling immune tolerance by programmed death-1 blockade with pidilizumab after autologous hematopoietic stem-cell transplantation for diffuse large B-cell lymphoma: results of an international phase II trial. *J Clin Oncol* 2013, 31(33):4199-4206.
2. Bryan LJ, Gordon LI: Pidilizumab in the treatment of diffuse large B-cell lymphoma. *Expert Opin Biol Ther* 2014, 14(9):1361-1368.
3. Hartmann S, Hansmann ML: Large B-cell lymphoma rich in PD-1+ T cells: an overlooked subtype of diffuse large B-cell lymphoma? *Am J Clin Pathol* 2014, 142(2):142-143.
4. Ohgami RS, Zhao S, Natkunam Y: Large B-cell lymphomas poor in B cells and rich in PD-1+ T cells can mimic T-cell lymphomas. *Am J Clin Pathol* 2014, 142(2):150-156.
5. Kochenderfer JN, Dudley ME, Kassim SH, Carpenter RO, Yang JC, Phan GQ, Hughes MS, Sherry RM, Feldman S, Spaner D *et al*: Effective Treatment Of Chemotherapy-Refractory Diffuse Large B-Cell Lymphoma With Autologous T Cells Genetically-Engineered To Express An Anti-CD19 Chimeric Antigen Receptor. *Blood* 2013, 122:21.
6. Kochenderfer JN, Dudley ME, Kassim SH, Somerville RP, Carpenter RO, Stetler-Stevenson M, Yang JC, Phan GQ, Hughes MS, Sherry RM *et al*: Chemotherapy-Refractory Diffuse Large B-Cell Lymphoma and Indolent B-Cell Malignancies Can Be Effectively Treated With Autologous T Cells Expressing an Anti-CD19 Chimeric Antigen Receptor. *J Clin Oncol* 2015, 33(6):540-549.
7. Micallef IN, Maurer MJ, Wiseman GA, Nikcevich DA, Kurtin PJ, Cannon MW, Perez DG, Soori GS, Link BK, Habermann TM *et al*: Epratuzumab with rituximab, cyclophosphamide, doxorubicin, vincristine, and prednisone chemotherapy in patients with previously untreated diffuse large B-cell lymphoma. *Blood* 2011, 118(15):4053-4061.
8. Mitrovic Z, Iqbal J, Fu K, Smith LM, Bast M, Greiner TC, Aoun P, Armitage JO, Vose JM, Weisenburger DD *et al*: CD43 expression is associated with inferior survival in the non-germinal centre B-cell subgroup of diffuse large B-cell lymphoma. *Br J Haematol* 2013, 162(1):87-92.
9. Ma XB, Zheng Y, Yuan HP, Jiang J, Wang YP: CD43 expression in diffuse large B-cell lymphoma, not otherwise specified: CD43 is a marker of adverse prognosis. *Hum Pathol* 2015, 46(4):593-599.
10. Rodig SJ, Abramson JS, Pinkus GS, Treon SP, Dorfman DM, Dong HY, Shipp MA, Kutok JL: Heterogeneous CD52 expression among hematologic neoplasms: implications for the use of alemtuzumab (CAMPATH-1H). *Clin Cancer Res* 2006, 12(23):7174-7179.
11. Younes A, Kim S, Romaguera J, Copeland A, Farial Sde C, Kwak LW, Fayad L, Hagemeister F, Fanale M, Neelapu S *et al*: Phase I multidose-escalation study of the anti-CD19 maytansinoid immunoconjugate SAR3419 administered by intravenous infusion every 3 weeks to patients with relapsed/refractory B-cell lymphoma. *J Clin Oncol* 2012, 30(22):2776-2782.
12. Ribrag V, Dupuis J, Tilly H, Morschhauser F, Laine F, Houot R, Haioun C, Copie C, Varga A, Lambert J *et al*: A dose-escalation study of SAR3419, an anti-CD19 antibody maytansinoid conjugate, administered by intravenous infusion once weekly in patients with relapsed/refractory B-cell non-Hodgkin lymphoma. *Clin Cancer Res* 2014, 20(1):213-220.
13. Teicher BA, Chari RV: Antibody conjugate therapeutics: challenges and potential. *Clin Cancer Res* 2011, 17(20):6389-6397.
14. Feld J, Barta SK, Schinke C, Braunschweig I, Zhou Y, Verma AK: Linked-in: design and efficacy of antibody drug conjugates in oncology. *Oncotarget* 2013, 4(3):397-412.
15. Pfeifer M, Zheng B, Erdmann T, Koeppen H, McCord R, Grau M, Staiger A, Chai A, Sandmann T, Madle H *et al*: Anti-CD22 and anti-CD79B antibody drug conjugates are active in different molecular diffuse large B-cell lymphoma subtypes. *Leukemia* 2015, 29(7):1578-1586.
16. Sotomayor EM, Young KH, Younes A: Clinical roundtable monograph: CD30 in lymphoma: its role in biology, diagnostic testing, and targeted therapy. *Clin Adv Hematol Oncol* 2014, 12(4 Suppl 10):1-22.
17. Hu S, Xu-Monette ZY, Balasubramanyam A, Manyam GC, Visco C, Tzankov A, Liu WM, Miranda RN, Zhang L, Montes-Moreno S *et al*: CD30 expression defines a novel subgroup of diffuse large B-cell lymphoma with favorable prognosis and distinct gene expression signature: a report from the International DLBCL Rituximab-CHOP Consortium Program Study. *Blood* 2013, 121(14):2715-2724.
18. A phase 2 study of brentuximab vedotin in patients with relapsed or refractory CD30-positive non-Hodgkin lymphomas: interim results in patients with DLBCL and other B-cell lymphomas. *Clin Adv Hematol Oncol* 2014, 12(2 Suppl 6):3-4.

19. Pore D, Bodo J, Danda A, Yan D, Phillips JG, Lindner D, Hill BT, Smith MR, Hsi ED, Gupta N: Identification of Ezrin-Radixin-Moesin proteins as novel regulators of pathogenic B cell receptor signaling and tumor growth in diffuse large B cell lymphoma. *Leukemia* 2015 Sep;29(9):1857-67
20. Fayad L, Offner F, Smith MR, Verhoef G, Johnson P, Kaufman JL, Rohatiner A, Advani A, Foran J, Hess G *et al*: Safety and clinical activity of a combination therapy comprising two antibody-based targeting agents for the treatment of non-Hodgkin lymphoma: results of a phase I/II study evaluating the immunoconjugate inotuzumab ozogamicin with rituximab. *J Clin Oncol* 2013, 31(5):573-583.
21. Biberacher V, Decker T, Oelsner M, Wagner M, Bogner C, Schmidt B, Kreitman RJ, Peschel C, Pastan I, Meyer Zum Buschenfelde C *et al*: The cytotoxicity of anti-CD22 immunotoxin is enhanced by bryostatin 1 in B-cell lymphomas through CD22 upregulation and PKC-betaII depletion. *Haematologica* 2012, 97(5):771-779.
22. Demosthenous C, Han JJ, Stenson MJ, Maurer MJ, Wellik LE, Link B, Hege K, Dogan A, Sotomayor E, Witzig T *et al*: Translation initiation complex eIF4F is a therapeutic target for dual mTOR kinase inhibitors in non-Hodgkin lymphoma. *Oncotarget* 2015, 6(11):9488-9501.
23. Steinhardt JJ, Peroutka RJ, Mazan-Mamczarz K, Chen Q, Houng S, Robles C, Barth RN, DuBose J, Bruns B, Tesoriero R *et al*: Inhibiting CARD11 translation during BCR activation by targeting the eIF4A RNA helicase. *Blood* 2014, 124(25):3758-3767.
24. Dunleavy K, Pittaluga S, Czuczman MS, Dave SS, Wright G, Grant N, Shovlin M, Jaffe ES, Janik JE, Staudt LM *et al*: Differential efficacy of bortezomib plus chemotherapy within molecular subtypes of diffuse large B-cell lymphoma. *Blood* 2009, 113(24):6069-6076.
25. Dasmahapatra G, Lembersky D, Kramer L, Fisher RI, Friedberg J, Dent P, Grant S: The pan-HDAC inhibitor vorinostat potentiates the activity of the proteasome inhibitor carfilzomib in human DLBCL cells in vitro and in vivo. *Blood* 2010, 115(22):4478-4487.
26. Dasmahapatra G, Lembersky D, Son MP, Patel H, Peterson D, Attkisson E, Fisher RI, Friedberg JW, Dent P, Grant S: Obatoclax interacts synergistically with the irreversible proteasome inhibitor carfilzomib in GC- and ABC-DLBCL cells in vitro and in vivo. *Mol Cancer Ther* 2012, 11(5):1122-1132.
27. Dasmahapatra G, Patel H, Dent P, Fisher RI, Friedberg J, Grant S: The Bruton tyrosine kinase (BTK) inhibitor PCI-32765 synergistically increases proteasome inhibitor activity in diffuse large-B cell lymphoma (DLBCL) and mantle cell lymphoma (MCL) cells sensitive or resistant to bortezomib. *Br J Haematol* 2013, 161(1):43-56.
28. Lam LT, Wright G, Davis RE, Lenz G, Farinha P, Dang L, Chan JW, Rosenwald A, Gascoyne RD, Staudt LM: Cooperative signaling through the signal transducer and activator of transcription 3 and nuclear factor-{kappa}B pathways in subtypes of diffuse large B-cell lymphoma. *Blood* 2008, 111(7):3701-3713.
29. Qu C, Liu Y, Kunkalla K, Singh RR, Blonska M, Lin X, Agarwal NK, Vega F: Trimeric G protein-CARMA1 axis links smoothened, the hedgehog receptor transducer, to NF-kappaB activation in diffuse large B-cell lymphoma. *Blood* 2013, 121(23):4718-4728.
30. Bavi P, Uddin S, Bu R, Ahmed M, Abubaker J, Balde V, Qadri Z, Ajarim D, Al-Dayel F, Hussain AR *et al*: The biological and clinical impact of inhibition of NF-kappaB-initiated apoptosis in diffuse large B cell lymphoma (DLBCL). *J Pathol* 2011, 224(3):355-366.
31. Zhang B, Calado DP, Wang Z, Frohler S, Kochert K, Qian Y, Koralov SB, Schmidt-Supprian M, Sasaki Y, Unitt C *et al*: An oncogenic role for alternative NF-kappaB signaling in DLBCL revealed upon deregulated BCL6 expression. *Cell Rep* 2015, 11(5):715-726.
32. Pham LV, Fu L, Tamayo AT, Bueso-Ramos C, Drakos E, Vega F, Medeiros LJ, Ford RJ: Constitutive BR3 receptor signaling in diffuse, large B-cell lymphomas stabilizes nuclear factor-kappaB-inducing kinase while activating both canonical and alternative nuclear factor-kappaB pathways. *Blood* 2011, 117(1):200-210.
33. Demchenko YN, Brents LA, Li Z, Bergsagel LP, McGee LR, Kuehl MW: Novel inhibitors are cytotoxic for myeloma cells with NFkB inducing kinase-dependent activation of NFkB. *Oncotarget* 2014, 5(12):4554-4566.
34. Lam LT, Davis RE, Pierce J, Hepperle M, Xu Y, Hottelet M, Nong Y, Wen D, Adams J, Dang L *et al*: Small molecule inhibitors of IkappaB kinase are selectively toxic for subgroups of diffuse large B-cell lymphoma defined by gene expression profiling. *Clin Cancer Res* 2005, 11(1):28-40.
35. Lam LT, Davis RE, Ngo VN, Lenz G, Wright G, Xu W, Zhao H, Yu X, Dang L, Staudt LM: Compensatory IKKalpha activation of classical NF-kappaB signaling during IKKbeta inhibition identified by an RNA interference sensitization screen. *Proc Natl Acad Sci U S A* 2008, 105(52):20798-20803.

36. Fabre C, Mimura N, Bobb K, Kong SY, Gorgun G, Cirstea D, Hu Y, Minami J, Ohguchi H, Zhang J *et al*: Dual inhibition of canonical and noncanonical NF-kappaB pathways demonstrates significant antitumor activities in multiple myeloma. *Clin Cancer Res* 2012, 18(17):4669-4681.
37. Bushell KR, Kim Y, Chan FC, Ben-Neriah S, Jenks A, Alcaide M, Fornika D, Grande BM, Arthur S, Gascoyne RD *et al*: Genetic inactivation of TRAF3 in canine and human B-cell lymphoma. *Blood* 2015, 125(6):999-1005.
38. Nogai H, Wenzel SS, Hailfinger S, Grau M, Kaergel E, Seitz V, Wollert-Wulf B, Pfeifer M, Wolf A, Frick M *et al*: IkappaB-zeta controls the constitutive NF-kappaB target gene network and survival of ABC DLBCL. *Blood* 2013, 122(13):2242-2250.
39. Fontan L, Yang C, Kabaleeswaran V, Volpon L, Osborne MJ, Beltran E, Garcia M, Cerchietti L, Shaknovich R, Yang SN *et al*: MALT1 small molecule inhibitors specifically suppress ABC-DLBCL in vitro and in vivo. *Cancer Cell* 2012, 22(6):812-824.
40. Ferch U, Kloo B, Gewies A, Pfander V, Duwel M, Peschel C, Krappmann D, Ruland J: Inhibition of MALT1 protease activity is selectively toxic for activated B cell-like diffuse large B cell lymphoma cells. *J Exp Med* 2009, 206(11):2313-2320.
41. Hailfinger S, Nogai H, Pelzer C, Jaworski M, Cabalzar K, Charton JE, Guzzardi M, Decaillet C, Grau M, Dorken B *et al*: Malt1-dependent RelB cleavage promotes canonical NF-kappaB activation in lymphocytes and lymphoma cell lines. *Proc Natl Acad Sci U S A* 2011, 108(35):14596-14601.
42. Nagel D, Spranger S, Vincendeau M, Grau M, Raffegerst S, Kloo B, Hlahla D, Neuenschwander M, Peter von Kries J, Hadian K *et al*: Pharmacologic inhibition of MALT1 protease by phenothiazines as a therapeutic approach for the treatment of aggressive ABC-DLBCL. *Cancer Cell* 2012, 22(6):825-837.
43. Lenz G, Davis RE, Ngo VN, Lam L, George TC, Wright GW, Dave SS, Zhao H, Xu W, Rosenwald A *et al*: Oncogenic CARD11 mutations in human diffuse large B cell lymphoma. *Science* 2008, 319(5870):1676-1679.
44. Lamason RL, McCully RR, Lew SM, Pomerantz JL: Oncogenic CARD11 mutations induce hyperactive signaling by disrupting autoinhibition by the PKC-responsive inhibitory domain. *Biochemistry* 2010, 49(38):8240-8250.
45. Ngo VN, Young RM, Schmitz R, Jhavar S, Xiao W, Lim KH, Kohlhammer H, Xu W, Yang Y, Zhao H *et al*: Oncogenically active MYD88 mutations in human lymphoma. *Nature* 2010, 470(7332):115-119.
46. Pulvino M, Chen L, Oleksyn D, Li J, Compitello G, Rossi R, Spence S, Balakrishnan V, Jordan C, Poligone B *et al*: Inhibition of COP9-signalosome (CSN) deneddylating activity and tumor growth of diffuse large B-cell lymphomas by doxycycline. *Oncotarget* 2015, 6(17):14796-14813.
47. Pulvino M, Liang Y, Oleksyn D, DeRan M, Van Pelt E, Shapiro J, Sanz I, Chen L, Zhao J: Inhibition of proliferation and survival of diffuse large B-cell lymphoma cells by a small-molecule inhibitor of the ubiquitin-conjugating enzyme Ubc13-Uev1A. *Blood* 2012, 120(8):1668-1677.
48. Yang Y, Schmitz R, Mitala J, Whiting A, Xiao W, Ceribelli M, Wright GW, Zhao H, Xu W, Rosenwald A *et al*: Essential role of the linear ubiquitin chain assembly complex in lymphoma revealed by rare germline polymorphisms. *Cancer Discov* 2014, 4(4):480-493.
49. Milhollen MA, Traore T, Adams-Duffy J, Thomas MP, Berger AJ, Dang L, Dick LR, Garnsey JJ, Koenig E, Langston SP *et al*: MLN4924, a NEDD8-activating enzyme inhibitor, is active in diffuse large B-cell lymphoma models: rationale for treatment of NF-{kappa}B-dependent lymphoma. *Blood* 2010, 116(9):1515-1523.
50. Wang Y, Luo Z, Pan Y, Wang W, Zhou X, Jeong LS, Chu Y, Liu J, Jia L: Targeting protein neddylation with an NEDD8-activating enzyme inhibitor MLN4924 induced apoptosis or senescence in human lymphoma cells. *Cancer Biol Ther* 2015, 16(3):420-429.
51. Zhang LH, Kosek J, Wang M, Heise C, Schafer PH, Chopra R: Lenalidomide efficacy in activated B-cell-like subtype diffuse large B-cell lymphoma is dependent upon IRF4 and cereblon expression. *Br J Haematol* 2013, 160(4):487-502.
52. Yang Y, Shaffer AL, 3rd, Emre NC, Ceribelli M, Zhang M, Wright G, Xiao W, Powell J, Platig J, Kohlhammer H *et al*: Exploiting synthetic lethality for the therapy of ABC diffuse large B cell lymphoma. *Cancer Cell* 2012, 21(6):723-737.
53. Hagner PR, Man HW, Fontanillo C, Wang M, Couto S, Breider M, Bjorklund C, Havens CG, Lu G, Rychak E *et al*: CC-122, a pleiotropic pathway modifier, mimics an interferon response and has antitumor activity in DLBCL. *Blood* 2015, 126(6):779-789.
54. Chamberlain PP, Lopez-Girona A, Miller K, Carmel G, Pagarigan B, Chie-Leon B, Rychak E, Corral LG, Ren YJ, Wang M *et al*: Structure of the human Cereblon-DDB1-lenalidomide complex reveals basis for responsiveness to thalidomide analogs. *Nature structural & molecular biology* 2014, 21(9):803-809.

55. Lu G, Middleton RE, Sun H, Naniong M, Ott CJ, Mitsiades CS, Wong KK, Bradner JE, Kaelin WG, Jr.: The myeloma drug lenalidomide promotes the cereblon-dependent destruction of Ikaros proteins. *Science* 2014, 343(6168):305-309.
56. Ribrag V, Damian S, Gharibo M, Gironella M, Santoro A, Rasco DW, Edenfield W, Wei X, James A, Hagner PR *et al*: CC-122 Degrades the Lymphoid Transcription Factor Aiolos (IKZF3) by Modulating Cereblon and Shows Clinical Activity in a Phase Ib Study of Subjects With Relapsed or Refractory Non-Hodgkin's Lymphoma and Multiple Myeloma. Presented at the ASH 56th Annual Meeting; San Francisco, CA USA Dec 5-9, 2014; 2014.
57. Davis RE, Ngo VN, Lenz G, Tolar P, Young RM, Romesser PB, Kohlhammer H, Lamy L, Zhao H, Yang Y *et al*: Chronic active B-cell-receptor signalling in diffuse large B-cell lymphoma. *Nature* 2010, 463(7277):88-92.
58. Naylor TL, Tang H, Ratsch BA, Enns A, Loo A, Chen L, Lenz P, Waters NJ, Schuler W, Dorken B *et al*: Protein kinase C inhibitor sotrastaurin selectively inhibits the growth of CD79 mutant diffuse large B-cell lymphomas. *Cancer Res* 2011, 71(7):2643-2653.
59. Friedberg JW, Sharman J, Sweetenham J, Johnston PB, Vose JM, Lacasce A, Schaefer-Cutillo J, De Vos S, Sinha R, Leonard JP *et al*: Inhibition of Syk with fostamatinib disodium has significant clinical activity in non-Hodgkin lymphoma and chronic lymphocytic leukemia. *Blood* 2010, 115(13):2578-2585.
60. Chen L, Monti S, Juszczynski P, Daley J, Chen W, Witzig TE, Habermann TM, Kutok JL, Shipp MA: SYK-dependent tonic B-cell receptor signaling is a rational treatment target in diffuse large B-cell lymphoma. *Blood* 2008, 111(4):2230-2237.
61. Chen L, Monti S, Juszczynski P, Ouyang J, Chapuy B, Neuberg D, Doench JG, Bogusz AM, Habermann TM, Dogan A *et al*: SYK inhibition modulates distinct PI3K/AKT- dependent survival pathways and cholesterol biosynthesis in diffuse large B cell lymphomas. *Cancer Cell* 2013, 23(6):826-838.
62. Cosenza M, Civallero M, Grisendi G, Marcheselli L, Roat E, Bari A, Sacchi S: Combination of low doses of enzastaurin and lenalidomide has synergistic activity in B-non-Hodgkin lymphoma cell lines. *Ann Hematol* 2012, 91(10):1613-1622.
63. Yang C, Lu P, Lee FY, Chadburn A, Barrientos JC, Leonard JP, Ye F, Zhang D, Knowles DM, Wang YL: Tyrosine kinase inhibition in diffuse large B-cell lymphoma: molecular basis for antitumor activity and drug resistance of dasatinib. *Leukemia* 2008, 22(9):1755-1766.
64. Huynh MQ, Gossmann J, Gattenloehner S, Klapper W, Wacker HH, Ramaswamy A, Bittner A, Kaiser U, Neubauer A: Expression and pro-survival function of phospholipase Cgamma2 in diffuse large B-cell lymphoma. *Leuk Lymphoma* 2015, 56(4):1088-1095.
65. Zang C, Eucker J, Liu H, Coordes A, Lenarz M, Possinger K, Scholz CW: Inhibition of pan-class I phosphatidyl-inositol-3-kinase by NVP-BKM120 effectively blocks proliferation and induces cell death in diffuse large B-cell lymphoma. *Leuk Lymphoma* 2014, 55(2):425-434.
66. Kahl B, Byrd JC, Flinn IW, Wagner-Johnston ND, Spurgeon SE, Benson DM, Furman RR, Brown JR, Coutre SE, Lannutti BJ *et al*: Clinical Safety and Activity In a Phase 1 Study of CAL-101, An Isoform-Selective Inhibitor of Phosphatidylinositol 3-Kinase P110{delta}, In Patients with Relapsed or Refractory Non-Hodgkin Lymphoma. *Blood, ASH Annual Meeting Abstracts* 2010, 116(21):741.
67. Dreyling M, Morschhauser F, Bron D, Bouabdallah K, Vitolo U, Linton K, Van Den Neste E, Mappa S, Giurescu M, Childs BH *et al*: Preliminary Results Of a Phase II Study Of Single Agent Bay 80-6946, a Novel PI3K Inhibitor, In Patients With Relapsed/Refractory, Indolent Or Aggressive Lymphoma. *Blood* 2013, 122:21.
68. Mathews Griner LA, Guha R, Shinn P, Young RM, Keller JM, Liu D, Goldlust IS, Yasgar A, McKnight C, Boxer MB *et al*: High-throughput combinatorial screening identifies drugs that cooperate with ibrutinib to kill activated B-cell-like diffuse large B-cell lymphoma cells. *Proc Natl Acad Sci U S A* 2014, 111(6):2349-2354.
69. Petrich AM, Leshchenko V, Kuo PY, Xia B, Thirukonda VK, Ulahannan N, Gordon S, Fazzari MJ, Ye BH, Sparano JA *et al*: Akt inhibitors MK-2206 and nelfinavir overcome mTOR inhibitor resistance in diffuse large B-cell lymphoma. *Clin Cancer Res* 2012, 18(9):2534-2544.
70. Devlin JR, Hannan KM, Ng PY, Bywater MJ, Shortt J, Cullinane C, McArthur GA, Johnstone RW, Hannan RD, Pearson RB: AKT signalling is required for ribosomal RNA synthesis and progression of Emu-Myc B-cell lymphoma in vivo. *Febs J* 2013, 280(21):5307-5316.
71. Witzig TE, Reeder CB, LaPlant BR, Gupta M, Johnston PB, Micallef IN, Porrata LF, Ansell SM, Colgan JP, Jacobsen ED *et al*: A phase II trial of the oral mTOR inhibitor everolimus in relapsed aggressive lymphoma. *Leukemia* 2011, 25(2):341-347.

72. Smith SM, van Besien K, Karrison T, Dancey J, McLaughlin P, Younes A, Smith S, Stiff P, Lester E, Modi S *et al*: Temsirolimus has activity in non-mantle cell non-Hodgkin's lymphoma subtypes: The University of Chicago phase II consortium. *J Clin Oncol* 2010, 28(31):4740-4746.
73. Ezell SA, Mayo M, Bihani T, Tepsuporn S, Wang S, Passino M, Grosskurth SE, Collins M, Parmentier J, Reimer C *et al*: Synergistic induction of apoptosis by combination of BTK and dual mTORC1/2 inhibitors in diffuse large B cell lymphoma. *Oncotarget* 2014, 5(13):4990-5001.
74. Gupta M, Ansell SM, Novak AJ, Kumar S, Kaufmann SH, Witzig TE: Inhibition of histone deacetylase overcomes rapamycin-mediated resistance in diffuse large B-cell lymphoma by inhibiting Akt signaling through mTORC2. *Blood* 2009, 114(14):2926-2935.
75. Mortensen DS, Fultz KE, Xu S, Xu W, Packard G, Khambatta G, Gamez JC, Leisten J, Zhao J, Apuy J *et al*: CC-223, a Potent and Selective Inhibitor of mTOR Kinase: In Vitro and In Vivo Characterization. *Mol Cancer Ther* 2015, 14(6):1295-1305.
76. Iqbal J, Meyer PN, Smith LM, Johnson NA, Vose JM, Greiner TC, Connors JM, Staudt LM, Rimsza L, Jaffe E *et al*: BCL2 predicts survival in germinal center B-cell-like diffuse large B-cell lymphoma treated with CHOP-like therapy and rituximab. *Clin Cancer Res* 2011, 17(24):7785-7795.
77. Mounier N, Briere J, Gisselbrecht C, Emile JF, Lederlin P, Sebban C, Berger F, Bosly A, Morel P, Tilly H *et al*: Rituximab plus CHOP (R-CHOP) overcomes bcl-2--associated resistance to chemotherapy in elderly patients with diffuse large B-cell lymphoma (DLBCL). *Blood* 2003, 101(11):4279-4284.
78. Paoluzzi L, Gonen M, Bhagat G, Furman RR, Gardner JR, Scotto L, Gueorguiev VD, Heaney ML, Manova K, O'Connor OA: The BH3-only mimetic ABT-737 synergizes the antineoplastic activity of proteasome inhibitors in lymphoid malignancies. *Blood* 2008, 112(7):2906-2916.
79. Thompson RC, Vardinogiannis I, Gilmore TD: The sensitivity of diffuse large B-cell lymphoma cell lines to histone deacetylase inhibitor-induced apoptosis is modulated by BCL-2 family protein activity. *PLoS One* 2013, 8(5):e62822.
80. Billard C: BH3 mimetics: status of the field and new developments. *Mol Cancer Ther* 2013, 12(9):1691-1700.
81. Oltersdorf T, Elmore SW, Shoemaker AR, Armstrong RC, Augeri DJ, Belli BA, Bruncko M, Deckwerth TL, Dinges J, Hajduk PJ *et al*: An inhibitor of Bcl-2 family proteins induces regression of solid tumours. *Nature* 2005, 435(7042):677-681.
82. Tse C, Shoemaker AR, Adickes J, Anderson MG, Chen J, Jin S, Johnson EF, Marsh KC, Mitten MJ, Nimmer P *et al*: ABT-263: a potent and orally bioavailable Bcl-2 family inhibitor. *Cancer Res* 2008, 68(9):3421-3428.
83. Souers AJ, Leverson JD, Boghaert ER, Ackler SL, Catron ND, Chen J, Dayton BD, Ding H, Enschede SH, Fairbrother WJ *et al*: ABT-199, a potent and selective BCL-2 inhibitor, achieves antitumor activity while sparing platelets. *Nat Med* 2013, 19(2):202-208.
84. Vandenberg CJ, Cory S: ABT-199, a new Bcl-2-specific BH3 mimetic, has in vivo efficacy against aggressive Myc-driven mouse lymphomas without provoking thrombocytopenia. *Blood* 2013, 121(12):2285-2288.
85. Iqbal J, Neppalli VT, Wright G, Dave BJ, Horsman DE, Rosenwald A, Lynch J, Hans CP, Weisenburger DD, Greiner TC *et al*: BCL2 expression is a prognostic marker for the activated B-cell-like type of diffuse large B-cell lymphoma. *J Clin Oncol* 2006, 24(6):961-968.
86. Wenzel SS, Grau M, Mavis C, Hailfinger S, Wolf A, Madle H, Deeb G, Dorken B, Thome M, Lenz P *et al*: MCL1 is deregulated in subgroups of diffuse large B-cell lymphoma. *Leukemia* 2013, 27(6):1381-1390.
87. Yecies D, Carlson NE, Deng J, Letai A: Acquired resistance to ABT-737 in lymphoma cells that up-regulate MCL-1 and BFL-1. *Blood* 2010, 115(16):3304-3313.
88. Shore GC, Viallet J: Modulating the bcl-2 family of apoptosis suppressors for potential therapeutic benefit in cancer. *Hematology Am Soc Hematol Educ Program* 2005:226-230.
89. Li L, Pongtornpipat P, Tiutan T, Kendrick SL, Park S, Persky DO, Rimsza LM, Puvvada SD, Schatz JH: Synergistic induction of apoptosis in high-risk DLBCL by BCL2 inhibition with ABT-199 combined with pharmacologic loss of MCL1. *Leukemia* 2015, 29(8):1702-1712.
90. Cillessen SA, Reed JC, Welsh K, Pinilla C, Houghten R, Hooijberg E, Deurhof J, Castricum KC, Kortman P, Hess CJ *et al*: Small-molecule XIAP antagonist restores caspase-9 mediated apoptosis in XIAP-positive diffuse large B-cell lymphoma cells. *Blood* 2008, 111(1):369-375.
91. Kaneko N, Kita A, Yamanaka K, Mori M: Combination of YM155, a survivin suppressant with a STAT3 inhibitor: a new strategy to treat diffuse large B-cell lymphoma. *Leuk Res* 2013, 37(9):1156-1161.

92. Kita A, Nakahara T, Yamanaka K, Nakano K, Nakata M, Mori M, Kaneko N, Koutoku H, Izumisawa N, Sasamata M: Antitumor effects of YM155, a novel survivin suppressant, against human aggressive non-Hodgkin lymphoma. *Leuk Res* 2011, 35(6):787-792.
93. Kaneko N, Mitsuoka K, Amino N, Yamanaka K, Kita A, Mori M, Miyoshi S, Kuromitsu S: Combination of YM155, a survivin suppressant, with bendamustine and rituximab: a new combination therapy to treat relapsed/refractory diffuse large B-cell lymphoma. *Clin Cancer Res* 2014, 20(7):1814-1822.
94. Cheson BD, Bartlett NL, Vose JM, Lopez-Hernandez A, Seiz AL, Keating AT, Shamsili S, Papadopoulos KP: A phase II study of the survivin suppressant YM155 in patients with refractory diffuse large B-cell lymphoma. *Cancer* 2012, 118(12):3128-3134.
95. Drakos E, Singh RR, Rassidakis GZ, Schlette E, Li J, Claret FX, Ford RJ, Jr., Vega F, Medeiros LJ: Activation of the p53 pathway by the MDM2 inhibitor nutlin-3a overcomes BCL2 overexpression in a preclinical model of diffuse large B-cell lymphoma associated with t(14;18)(q32;q21). *Leukemia* 2011, 25(5):856-867.
96. Cerchietti LC, Ghetu AF, Zhu X, Da Silva GF, Zhong S, Matthews M, Bunting KL, Polo JM, Fares C, Arrowsmith CH *et al*: A small-molecule inhibitor of BCL6 kills DLBCL cells in vitro and in vivo. *Cancer Cell* 2010, 17(4):400-411.
97. Cerchietti LC, Hatzi K, Caldas-Lopes E, Yang SN, Figueroa ME, Morin RD, Hirst M, Mendez L, Shaknovich R, Cole PA *et al*: BCL6 repression of EP300 in human diffuse large B cell lymphoma cells provides a basis for rational combinatorial therapy. *J Clin Invest* 2010.
98. Cerchietti LC, Yang SN, Shaknovich R, Hatzi K, Polo JM, Chadburn A, Dowdy SF, Melnick A: A peptomimetic inhibitor of BCL6 with potent antilymphoma effects in vitro and in vivo. *Blood* 2009, 113(15):3397-3405.
99. Juszczynski P, Chen L, O'Donnell E, Polo JM, Ranuncolo SM, Dalla-Favera R, Melnick A, Shipp MA: BCL6 modulates tonic BCR signaling in diffuse large B-cell lymphomas by repressing the SYK phosphatase, PTPROt. *Blood* 2009, 114(26):5315-5321.
100. Polo JM, Juszczynski P, Monti S, Cerchietti L, Ye K, Greally JM, Shipp M, Melnick A: Transcriptional signature with differential expression of BCL6 target genes accurately identifies BCL6-dependent diffuse large B cell lymphomas. *Proc Natl Acad Sci U S A* 2007, 104(9):3207-3212.
101. Castellano G, Torrisi E, Ligresti G, Nicoletti F, Malaponte G, Traval S, McCubrey JA, Canevari S, Libra M: Yin Yang 1 overexpression in diffuse large B-cell lymphoma is associated with B-cell transformation and tumor progression. *Cell Cycle* 2010, 9(3):557-563.
102. Green MR, Monti S, Dalla-Favera R, Pasqualucci L, Walsh NC, Schmidt-Supprian M, Kutok JL, Rodig SJ, Neuberg DS, Rajewsky K *et al*: Signatures of murine B-cell development implicate Yy1 as a regulator of the germinal center-specific program. *Proc Natl Acad Sci U S A* 2011, 108(7):2873-2878.
103. Ramkumar C, Cui H, Kong Y, Jones SN, Gerstein RM, Zhang H: Smurf2 suppresses B-cell proliferation and lymphomagenesis by mediating ubiquitination and degradation of YY1. *Nat Commun* 2013, 4:2598.
104. Wong KK, Gascoyne DM, Brown PJ, Soilleux EJ, Snell C, Chen H, Lyne L, Lawrie CH, Gascoyne RD, Pedersen LM *et al*: Reciprocal expression of the endocytic protein HIP1R and its repressor FOXP1 predicts outcome in R-CHOP-treated diffuse large B-cell lymphoma patients. *Leukemia* 2014, 28(2):362-372.
105. Walker MP, Stopford CM, Cederlund M, Fang F, Jahn C, Rabinowitz AD, Goldfarb D, Graham DM, Yan F, Deal AM *et al*: FOXP1 potentiates Wnt/beta-catenin signaling in diffuse large B cell lymphoma. *Science signaling* 2015, 8(362):ra12.
106. van Keimpema M, Gruneberg LJ, Mokry M, van Boxtel R, Koster J, Coffer PJ, Pals ST, Spaargaren M: FOXP1 directly represses transcription of proapoptotic genes and cooperates with NF-kappaB to promote survival of human B cells. *Blood* 2014, 124(23):3431-3440.
107. Shringarpure R, Catley L, Bhole D, Burger R, Podar K, Tai YT, Kessler B, Galardy P, Ploegh H, Tassone P *et al*: Gene expression analysis of B-lymphoma cells resistant and sensitive to bortezomib. *Br J Haematol* 2006, 134(2):145-156.
108. Abramson JS, Chen W, Juszczynski P, Takahashi H, Neuberg D, Kutok JL, Takeyama K, Shipp MA: The heat shock protein 90 inhibitor IPI-504 induces apoptosis of AKT-dependent diffuse large B-cell lymphomas. *Br J Haematol* 2009, 144(3):358-366.
109. Cerchietti LC, Lopes EC, Yang SN, Hatzi K, Bunting KL, Tsikitas LA, Mallik A, Robles AI, Walling J, Varticovski L *et al*: A purine scaffold Hsp90 inhibitor destabilizes BCL-6 and has specific antitumor activity in BCL-6-dependent B cell lymphomas. *Nat Med* 2009, 15(12):1369-1376.
110. Beguelin W, Sawh S, Chambwe N, Chun Chan F, Jiang Y, Choo JW, Scott DW, Chalmers A, Geng H, Tsikitas L *et al*: IL10 receptor is a novel therapeutic target in DLBCLs. *Leukemia* 2015.

111. Gupta M, Han JJ, Stenson M, Maurer M, Wellik L, Hu G, Ziesmer S, Dogan A, Witzig TE: Elevated serum IL-10 levels in diffuse large B-cell lymphoma: a mechanism of aberrant JAK2 activation. *Blood* 2012, 119(12):2844-2853.
112. Camicia R, Bachmann SB, Winkler HC, Beer M, Tinguely M, Haralambieva E, Hassa PO: BAL1/ARTD9 represses the anti-proliferative and pro-apoptotic IFNgamma-STAT1-IRF1-p53 axis in diffuse large B-cell lymphoma. *J Cell Sci* 2013, 126(Pt 9):1969-1980.
113. Witzig TE, Price-Troska TL, Stenson MJ, Gupta M: Lack of JAK2 activating non-synonymous mutations in diffuse large B-cell tumors: JAK2 deregulation still unexplained. *Leuk Lymphoma* 2013, 54(2):397-399.
114. Ding BB, Yu JJ, Yu RY, Mendez LM, Shaknovich R, Zhang Y, Cattoretti G, Ye BH: Constitutively activated STAT3 promotes cell proliferation and survival in the activated B-cell subtype of diffuse large B-cell lymphomas. *Blood* 2008, 111(3):1515-1523.
115. Gupta M, Han JJ, Stenson M, Wellik L, Witzig TE: Regulation of STAT3 by histone deacetylase-3 in diffuse large B-cell lymphoma: implications for therapy. *Leukemia* 2012, 26(6):1356-1364.
116. Huang X, Meng B, Iqbal J, Ding BB, Perry AM, Cao W, Smith LM, Bi C, Jiang C, Greiner TC *et al*: Activation of the STAT3 signaling pathway is associated with poor survival in diffuse large B-cell lymphoma treated with R-CHOP. *J Clin Oncol* 2013, 31(36):4520-4528.
117. Hu G, Witzig TE, Gupta M: A novel missense (M206K) STAT3 mutation in diffuse large B cell lymphoma deregulates STAT3 signaling. *PLoS One* 2013, 8(7):e67851.
118. Johnson NA, Slack GW, Savage KJ, Connors JM, Ben-Neriah S, Rogic S, Scott DW, Tan KL, Steidl C, Sehn LH *et al*: Concurrent expression of MYC and BCL2 in diffuse large B-cell lymphoma treated with rituximab plus cyclophosphamide, doxorubicin, vincristine, and prednisone. *J Clin Oncol* 2012, 30(28):3452-3459.
119. Green TM, Young KH, Visco C, Xu-Monette ZY, Orazi A, Go RS, Nielsen O, Gadeberg OV, Mourits-Andersen T, Frederiksen M *et al*: Immunohistochemical double-hit score is a strong predictor of outcome in patients with diffuse large B-cell lymphoma treated with rituximab plus cyclophosphamide, doxorubicin, vincristine, and prednisone. *J Clin Oncol* 2012, 30(28):3460-3467.
120. Perry AM, Alvarado-Bernal Y, Laurini JA, Smith LM, Slack GW, Tan KL, Sehn LH, Fu K, Aoun P, Greiner TC *et al*: MYC and BCL2 protein expression predicts survival in patients with diffuse large B-cell lymphoma treated with rituximab. *Br J Haematol* 2014, 165(3):382-391.
121. Hu S, Xu-Monette ZY, Tzankov A, Green T, Wu L, Balasubramanyam A, Liu WM, Visco C, Li Y, Miranda RN *et al*: MYC/BCL2 protein coexpression contributes to the inferior survival of activated B-cell subtype of diffuse large B-cell lymphoma and demonstrates high-risk gene expression signatures: a report from The International DLBCL Rituximab-CHOP Consortium Program. *Blood* 2013, 121(20):4021-4031; quiz 4250.
122. Ceribelli M, Kelly PN, Shaffer AL, Wright GW, Xiao W, Yang Y, Mathews Griner LA, Guha R, Shinn P, Keller JM *et al*: Blockade of oncogenic IkappaB kinase activity in diffuse large B-cell lymphoma by bromodomain and extraterminal domain protein inhibitors. *Proc Natl Acad Sci U S A* 2014, 111(31):11365-11370.
123. Chapuy B, McKeown MR, Lin CY, Monti S, Roemer MG, Qi J, Rahl PB, Sun HH, Yeda KT, Doench JG *et al*: Discovery and characterization of super-enhancer-associated dependencies in diffuse large B cell lymphoma. *Cancer Cell* 2013, 24(6):777-790.
124. Emadali A, Rousseaux S, Bruder-Costa J, Rome C, Duley S, Hamaidia S, Betton P, Debernardi A, Leroux D, Bernay B *et al*: Identification of a novel BET bromodomain inhibitor-sensitive, gene regulatory circuit that controls Rituximab response and tumour growth in aggressive lymphoid cancers. *EMBO Mol Med* 2013, 5(8):1180-1195.
125. Mertz JA, Conery AR, Bryant BM, Sandy P, Balasubramanian S, Mele DA, Bergeron L, Sims RJ, 3rd: Targeting MYC dependence in cancer by inhibiting BET bromodomains. *Proc Natl Acad Sci U S A* 2011, 108(40):16669-16674.
126. Ott CJ, Kopp N, Bird L, Paranal RM, Qi J, Bowman T, Rodig SJ, Kung AL, Bradner JE, Weinstock DM: BET bromodomain inhibition targets both c-Myc and IL7R in high-risk acute lymphoblastic leukemia. *Blood* 2012, 120(14):2843-2852.
127. Picaud S, Da Costa D, Thanasopoulou A, Filippakopoulos P, Fish PV, Philpott M, Fedorov O, Brennan P, Bunnage ME, Owen DR *et al*: PFI-1, a highly selective protein interaction inhibitor, targeting BET Bromodomains. *Cancer Res* 2013, 73(11):3336-3346.
128. Lenburg ME, Sinha A, Faller DV, Denis GV: Tumor-specific and proliferation-specific gene expression typifies murine transgenic B cell lymphomagenesis. *J Biol Chem* 2007, 282(7):4803-4811.

129. Chaidos A, Caputo V, Gouvedenou K, Liu B, Marigo I, Chaudhry MS, Rotolo A, Tough DF, Smithers NN, Bassil AK *et al*: Potent antimyeloma activity of the novel bromodomain inhibitors I-BET151 and I-BET762. *Blood* 2014, 123(5):697-705.
130. Boi M, Gaudio E, Bonetti P, Kwee I, Bernasconi E, Tarantelli C, Rinaldi A, Testoni M, Cascione L, Ponzoni M *et al*: The BET Bromodomain Inhibitor OTX015 Affects Pathogenetic Pathways in Preclinical B-cell Tumor Models and Synergizes with Targeted Drugs. *Clin Cancer Res* 2015, 21(7):1628-1638.
131. Lu J, Qian Y, Altieri M, Dong H, Wang J, Raina K, Hines J, Winkler JD, Crew AP, Coleman K *et al*: Hijacking the E3 Ubiquitin Ligase Cereblon to Efficiently Target BRD4. *Chem Biol* 2015, 22(6):755-763.
132. Cunningham JT, Moreno MV, Lodi A, Ronen SM, Ruggero D: Protein and nucleotide biosynthesis are coupled by a single rate-limiting enzyme, PRPS2, to drive cancer. *Cell* 2014, 157(5):1088-1103.
133. Friedberg JW, Mahadevan D, Cebula E, Persky D, Lossos I, Agarwal AB, Jung J, Burack R, Zhou X, Leonard EJ *et al*: Phase II study of alisertib, a selective Aurora A kinase inhibitor, in relapsed and refractory aggressive B- and T-cell non-Hodgkin lymphomas. *Journal of clinical oncology : official journal of the American Society of Clinical Oncology* 2014, 32(1):44-50.
134. Mahadevan D, Morales C, Cooke LS, Manziello A, Mount DW, Persky DO, Fisher RI, Miller TP, Qi W: Alisertib added to rituximab and vincristine is synthetic lethal and potentially curative in mice with aggressive DLBCL co-overexpressing MYC and BCL2. *PLoS One* 2014, 9(6):e95184.
135. Clozel T, Yang S, Elstrom RL, Tam W, Martin P, Kormaksson M, Banerjee S, Vasanthakumar A, Culjkovic B, Scott DW *et al*: Mechanism-based epigenetic chemosensitization therapy of diffuse large B-cell lymphoma. *Cancer Discov* 2013, 3(9):1002-1019.
136. Berg T, Thoene S, Yap D, Wee T, Schoeler N, Rosten P, Lim E, Bilenky M, Mungall AJ, Oellerich T *et al*: A transgenic mouse model demonstrating the oncogenic role of mutations in the polycomb-group gene EZH2 in lymphomagenesis. *Blood* 2014, 123(25):3914-3924.
137. Lohr JG, Stojanov P, Lawrence MS, Auclair D, Chapuy B, Sougnez C, Cruz-Gordillo P, Knoechel B, Asmann YW, Slager SL *et al*: Discovery and prioritization of somatic mutations in diffuse large B-cell lymphoma (DLBCL) by whole-exome sequencing. *Proc Natl Acad Sci U S A* 2012, 109(10):3879-3884.
138. McCabe MT, Ott HM, Ganji G, Korenchuk S, Thompson C, Van Aller GS, Liu Y, Graves AP, Della Pietra A, 3rd, Diaz E *et al*: EZH2 inhibition as a therapeutic strategy for lymphoma with EZH2-activating mutations. *Nature* 2012, 492(7427):108-112.
139. Morin RD, Johnson NA, Severson TM, Mungall AJ, An J, Goya R, Paul JE, Boyle M, Woolcock BW, Kuchenbauer F *et al*: Somatic mutations altering EZH2 (Tyr641) in follicular and diffuse large B-cell lymphomas of germinal-center origin. *Nat Genet* 2010, 42(2):181-185.
140. Velichutina I, Shaknovich R, Geng H, Johnson NA, Gascoyne RD, Melnick AM, Elemento O: EZH2-mediated epigenetic silencing in germinal center B cells contributes to proliferation and lymphomagenesis. *Blood* 2010, 116(24):5247-5255.
141. Beguelin W, Popovic R, Teater M, Jiang Y, Bunting KL, Rosen M, Shen H, Yang SN, Wang L, Ezponda T *et al*: EZH2 is required for germinal center formation and somatic EZH2 mutations promote lymphoid transformation. *Cancer Cell* 2013, 23(5):677-692.
142. Knutson SK, Kawano S, Minoshima Y, Warholic NM, Huang KC, Xiao Y, Kadowaki T, Uesugi M, Kuznetsov G, Kumar N *et al*: Selective inhibition of EZH2 by EPZ-6438 leads to potent antitumor activity in EZH2-mutant non-Hodgkin lymphoma. *Mol Cancer Ther* 2014, 13(4):842-854.
143. Bradley WD, Arora S, Busby J, Balasubramanian S, Gehling VS, Nasveschuk CG, Vaswani RG, Yuan CC, Hatton C, Zhao F *et al*: EZH2 inhibitor efficacy in non-Hodgkin's lymphoma does not require suppression of H3K27 monomethylation. *Chem Biol* 2014, 21(11):1463-1475.
144. Bereshchenko OR, Gu W, Dalla-Favera R: Acetylation inactivates the transcriptional repressor BCL6. *Nat Genet* 2002, 32(4):606-613.
145. Amengual JE, Clark-Garvey S, Kalac M, Scotto L, Marchi E, Neylon E, Johannet P, Wei Y, Zain J, O'Connor OA: Sirtuin and pan-class I/II deacetylase (DAC) inhibition is synergistic in preclinical models and clinical studies of lymphoma. *Blood* 2013, 122(12):2104-2113.
146. Dasmahapatra G, Patel H, Friedberg J, Quayle SN, Jones SS, Grant S: In vitro and in vivo interactions between the HDAC6 inhibitor ricolinostat (ACY1215) and the irreversible proteasome inhibitor carfilzomib in non-Hodgkin lymphoma cells. *Mol Cancer Ther* 2014, 13(12):2886-2897.
147. Jang KY, Hwang SH, Kwon KS, Kim KR, Choi HN, Lee NR, Kwak JY, Park BH, Park HS, Chung MJ *et al*: SIRT1 expression is associated with poor prognosis of diffuse large B-cell lymphoma. *Am J Surg Pathol* 2008, 32(10):1523-1531.

148. Caro P, Kishan AU, Norberg E, Stanley IA, Chapuy B, Ficarro SB, Polak K, Tondera D, Gounarides J, Yin H *et al*: Metabolic signatures uncover distinct targets in molecular subsets of diffuse large B cell lymphoma. *Cancer Cell* 2012, 22(4):547-560.
149. Dai B, Zhao XF, Mazan-Mamczarz K, Hagner P, Corl S, Bahassi el M, Lu S, Stambrook PJ, Shapiro P, Gartenhaus RB: Functional and molecular interactions between ERK and CHK2 in diffuse large B-cell lymphoma. *Nat Commun* 2011, 2:402.
150. Dai B, Zhao XF, Hagner P, Shapiro P, Mazan-Mamczarz K, Zhao S, Natkunam Y, Gartenhaus RB: Extracellular signal-regulated kinase positively regulates the oncogenic activity of MCT-1 in diffuse large B-cell lymphoma. *Cancer Res* 2009, 69(19):7835-7843.
151. Bhalla S, Evens AM, Dai B, Prachand S, Gordon LI, Gartenhaus RB: The novel anti-MEK small molecule AZD6244 induces BIM-dependent and AKT-independent apoptosis in diffuse large B-cell lymphoma. *Blood* 2011, 118(4):1052-1061.
152. Baron BW, Anastasi J, Hyjek EM, Bies J, Reddy PL, Dong J, Joseph L, Thirman MJ, Wroblewski K, Wolff L *et al*: PIM1 gene cooperates with human BCL6 gene to promote the development of lymphomas. *Proc Natl Acad Sci U S A* 2012, 109(15):5735-5739.
153. Gomez-Abad C, Pisonero H, Blanco-Aparicio C, Roncador G, Gonzalez-Menchen A, Martinez-Climent JA, Mata E, Rodriguez ME, Munoz-Gonzalez G, Sanchez-Beato M *et al*: PIM2 inhibition as a rational therapeutic approach in B-cell lymphoma. *Blood* 2011, 118(20):5517-5527.
154. Brault L, Menter T, Obermann EC, Knapp S, Thommen S, Schwaller J, Tzankov A: PIM kinases are progression markers and emerging therapeutic targets in diffuse large B-cell lymphoma. *British journal of cancer* 2012, 107(3):491-500.
155. Schmid CA, Robinson MD, Scheifinger NA, Muller S, Cogliatti S, Tzankov A, Muller A: DUSP4 deficiency caused by promoter hypermethylation drives JNK signaling and tumor cell survival in diffuse large B cell lymphoma. *The Journal of experimental medicine* 2015, 212(5):775-792.
156. Gregory GP, Hogg SJ, Kats LM, Vidacs E, Baker AJ, Gilan O, Lefebure M, Martin BP, Dawson MA, Johnstone RW *et al*: CDK9 inhibition by dinaciclib potently suppresses Mcl-1 to induce durable apoptotic responses in aggressive MYC-driven B-cell lymphoma in vivo. *Leukemia* 2015, 29(6):1437-1441.
157. Lacrima K, Rinaldi A, Vignati S, Martin V, Tibiletti MG, Gaidano G, Catapano CV, Bertoni F: Cyclin-dependent kinase inhibitor seliciclib shows in vitro activity in diffuse large B-cell lymphomas. *Leuk Lymphoma* 2007, 48(1):158-167.
158. Zhao MY, Auerbach A, D'Costa AM, Rapoport AP, Burger AM, Sausville EA, Stass SA, Jiang F, Sands AM, Aguilera N *et al*: Phospho-p70S6K/p85S6K and cdc2/cdk1 are novel targets for diffuse large B-cell lymphoma combination therapy. *Clin Cancer Res* 2009, 15(5):1708-1720.
159. Bongero D, Paoluzzi L, Marchi E, Zullo KM, Neisa R, Mao Y, Escandon R, Wood K, O'Connor OA: The Novel Kinesin Spindle Protein (KSP) Inhibitor SB-743921 Exhibits Marked Activity in In Vivo and In Vitro Models of Aggressive Large B-Cell Lymphoma. *Leuk Lymphoma* 2015:1-25.
160. Juszczynski P, Kutok JL, Li C, Mitra J, Aguiar RC, Shipp MA: BAL1 and BBAP are regulated by a gamma interferon-responsive bidirectional promoter and are overexpressed in diffuse large B-cell lymphomas with a prominent inflammatory infiltrate. *Mol Cell Biol* 2006, 26(14):5348-5359.
161. Masuda S, Kumano K, Suzuki T, Tomita T, Iwatsubo T, Natsugari H, Tojo A, Shibutani M, Mitsumori K, Hanazono Y *et al*: Dual antitumor mechanisms of Notch signaling inhibitor in a T-cell acute lymphoblastic leukemia xenograft model. *Cancer Sci* 2009, 100(12):2444-2450.
162. Monti S, Savage KJ, Kutok JL, Feuerhake F, Kurtin P, Mihm M, Wu B, Pasqualucci L, Neuberg D, Aguiar RC *et al*: Molecular profiling of diffuse large B-cell lymphoma identifies robust subtypes including one characterized by host inflammatory response. *Blood* 2005, 105(5):1851-1861.
163. Lee SY, Kumano K, Nakazaki K, Sanada M, Matsumoto A, Yamamoto G, Nannya Y, Suzuki R, Ota S, Ota Y *et al*: Gain-of-function mutations and copy number increases of Notch2 in diffuse large B-cell lymphoma. *Cancer Sci* 2009, 100(5):920-926.
164. Cho SH, Ahn AK, Bhargava P, Lee CH, Eischen CM, McGuinness O, Boothby M: Glycolytic rate and lymphomagenesis depend on PARP14, an ADP ribosyltransferase of the B aggressive lymphoma (BAL) family. *Proc Natl Acad Sci U S A* 2011, 108(38):15972-15977.
165. Aguiar RC, Yakushijin Y, Kharbanda S, Salgia R, Fletcher JA, Shipp MA: BAL is a novel risk-related gene in diffuse large B-cell lymphomas that enhances cellular migration. *Blood* 2000, 96(13):4328-4334.
166. Ageberg M, Rydstrom K, Linden O, Linderoth J, Jerkeman M, Drott K: Inhibition of geranylgeranylation mediates sensitivity to CHOP-induced cell death of DLBCL cell lines. *Exp Cell Res* 2011, 317(8):1179-1191.

167. Stopeck AT, Unger JM, Rimsza LM, Bellamy WT, Iannone M, Persky DO, Leblanc M, Fisher RI, Miller TP: A phase II trial of single agent bevacizumab in patients with relapsed, aggressive non-Hodgkin lymphoma: Southwest oncology group study S0108. *Leuk Lymphoma* 2009, 50(5):728-735.
168. Stopeck AT, Unger JM, Rimsza LM, LeBlanc M, Farnsworth B, Iannone M, Glenn MJ, Fisher RI, Miller TP: A phase 2 trial of standard-dose cyclophosphamide, doxorubicin, vincristine, prednisone (CHOP) and rituximab plus bevacizumab for patients with newly diagnosed diffuse large B-cell non-Hodgkin lymphoma: SWOG 0515. *Blood* 2012, 120(6):1210-1217.
169. Seymour JF, Pfreundschuh M, Trneny M, Sehn LH, Catalano J, Csinady E, Moore N, Coiffier B: R-CHOP with or without bevacizumab in patients with previously untreated diffuse large B-cell lymphoma: final MAIN study outcomes. *Haematologica* 2014, 99(8):1343-1349.
170. Ruan J, Luo M, Wang C, Fan L, Yang SN, Cardenas M, Geng H, Leonard JP, Melnick A, Cerchietti L *et al*: Imatinib disrupts lymphoma angiogenesis by targeting vascular pericytes. *Blood* 2013, 121(26):5192-5202.
171. Shin HC, Seo J, Kang BW, Moon JH, Chae YS, Lee SJ, Lee YJ, Han S, Seo SK, Kim JG *et al*: Clinical significance of nuclear factor kappaB and chemokine receptor CXCR4 expression in patients with diffuse large B-cell lymphoma who received rituximab-based therapy. *Korean J Intern Med* 2014, 29(6):785-792.
172. Chen J, Xu-Monette ZY, Deng L, Shen Q, Manyam GC, Martinez-Lopez A, Zhang L, Montes-Moreno S, Visco C, Tzankov A *et al*: Dysregulated CXCR4 expression promotes lymphoma cell survival and independently predicts disease progression in germinal center B-cell-like diffuse large B-cell lymphoma. *Oncotarget* 2015, 6(8):5597-5614.
173. Moreno MJ, Bosch R, Dieguez-Gonzalez R, Novelli S, Mozos A, Gallardo A, Pavon MA, Cespedes MV, Granena A, Alcoceba M *et al*: CXCR4 expression enhances diffuse large B cell lymphoma dissemination and decreases patient survival. *J Pathol* 2015, 235(3):445-455.
174. Uddin S, Hussain AR, Ahmed M, Siddiqui K, Al-Dayel F, Bavi P, Al-Kuraya KS: Overexpression of FoxM1 offers a promising therapeutic target in diffuse large B-cell lymphoma. *Haematologica* 2012, 97(7):1092-1100.
175. Singh RR, Kunkalla K, Qu C, Schlette E, Neelapu SS, Samaniego F, Vega F: ABCG2 is a direct transcriptional target of hedgehog signaling and involved in stroma-induced drug tolerance in diffuse large B-cell lymphoma. *Oncogene* 2011, 30(49):4874-4886.
176. Li C, Thompson MA, Tamayo AT, Zuo Z, Lee J, Vega F, Ford RJ, Pham LV: Over-expression of Thioredoxin-1 mediates growth, survival, and chemoresistance and is a druggable target in diffuse large B-cell lymphoma. *Oncotarget* 2012, 3(3):314-326.
177. Song YH, Zhong MZ, Gan PP, Yi PY, Tang YH, Liu YP, Jiang JQ, Li L: ALDH1A1 mediates resistance of diffuse large B cell lymphoma to the CHOP regimen. *Tumour Biol* 2014, 35(12):11809-11817.
178. Rickles RJ, Pierce LT, Giordano TP, 3rd, Tam WF, McMillin DW, Delmore J, Laubach JP, Borisy AA, Richardson PG, Lee MS: Adenosine A2A receptor agonists and PDE inhibitors: a synergistic multitarget mechanism discovered through systematic combination screening in B-cell malignancies. *Blood* 2010, 116(4):593-602.
179. Kim SW, Rai D, McKeller MR, Aguiar RC: Rational combined targeting of phosphodiesterase 4B and SYK in DLBCL. *Blood* 2009, 113(24):6153-6160.
180. Smith PG, Wang F, Wilkinson KN, Savage KJ, Klein U, Neuberg DS, Bollag G, Shipp MA, Aguiar RC: The phosphodiesterase PDE4B limits cAMP-associated PI3K/AKT-dependent apoptosis in diffuse large B-cell lymphoma. *Blood* 2005, 105(1):308-316.
181. Maxwell SA, Cherry EM, Bayless KJ: Akt, 14-3-3zeta, and vimentin mediate a drug-resistant invasive phenotype in diffuse large B-cell lymphoma. *Leuk Lymphoma* 2011, 52(5):849-864.
182. Maxwell SA, Li Z, Jaye D, Ballard S, Ferrell J, Fu H: 14-3-3zeta mediates resistance of diffuse large B cell lymphoma to an anthracycline-based chemotherapeutic regimen. *J Biol Chem* 2009, 284(33):22379-22389.
183. Derenzini E, Agostinelli C, Imbrogno E, Iacobucci I, Casadei B, Brighenti E, Righi S, Fuligni F, Ghelli Luserna Di Rora A, Ferrari A *et al*: Constitutive activation of the DNA damage response pathway as a novel therapeutic target in diffuse large B-cell lymphoma. *Oncotarget* 2015, 6(9):6553-6569.
184. Witzig TE, Vose JM, Zinzani PL, Reeder CB, Buckstein R, Polikoff JA, Bouabdallah R, Haioun C, Tilly H, Guo P *et al*: An international phase II trial of single-agent lenalidomide for relapsed or refractory aggressive B-cell non-Hodgkin's lymphoma. *Ann Oncol* 2011, 22(7):1622-1627.
185. Wiernik PH, Lossos IS, Tuscano JM, Justice G, Vose JM, Cole CE, Lam W, McBride K, Wride K, Pietronigro D *et al*: Lenalidomide monotherapy in relapsed or refractory aggressive non-Hodgkin's lymphoma. *J Clin Oncol* 2008, 26(30):4952-4957.

186. Vose JM, Habermann TM, Czuczman MS, Zinzani PL, Reeder CB, Tuscano JM, Lossos IS, Li J, Pietronigro D, Witzig TE: Single-agent lenalidomide is active in patients with relapsed or refractory aggressive non-Hodgkin lymphoma who received prior stem cell transplantation. *Br J Haematol* 2013, 162(5):639-647.
187. Hernandez-Ilizaliturri FJ, Deeb G, Zinzani PL, Pileri SA, Malik F, Macon WR, Goy A, Witzig TE, Czuczman MS: Higher response to lenalidomide in relapsed/refractory diffuse large B-cell lymphoma in nongerminal center B-cell-like than in germinal center B-cell-like phenotype. *Cancer* 2011, 117(22):5058-5066.
188. Czuczman M, Davies A, Linton KM, Wagner-Johnston N, Gascoyne RD, Eberhard DA, Salles G, Witzig TE, Zinzani PL, Wright GW *et al*: Phase 2/3 Multicenter, Randomized Study Comparing the Efficacy and Safety of Lenalidomide Versus Investigator's Choice in Relapsed/Refractory DLBCL. *Presented at the ASH 56th Annual Meeting; San Francisco, CA USA Dec 5-9, 2014; Blood: 124 (21), December 6, 2014;* 2014.
189. Rasco DW, Gandhi AK, James A, Li S, O'Mara E, Chopra R, DiMartino JF, Shih K: A First In Human Dose Escalation Study Of CC-122, A First-In-Class Pleiotropic Pathway Modulator™ (PPM) Compound In Subjects With Relapsed Or Refractory Solid Tumors, Multiple Myeloma and Non-Hodgkin's Lymphoma. *Presented at the ASH 55th Annual Meeting; Dec 7-10, 2013; New Orleans, LA USA* 2013.
190. Schwarzbich MA, Witzens-Harig M: Ibrutinib. *Recent Results Cancer Res* 2014, 201:259-267.
191. Wilson WH, Gerecitano JF, Goy A, Kenkre VP, Barr PM, Blum KA, Shustov AR, Advani R, Lih J, Williams M *et al*: The Bruton's tyrosine kinase (BTK) inhibitor, ibrutinib (PCI-32765), has preferential activity in the ABC subtype of relapsed/refractory de novo diffuse large B-cell lymphoma (DLBCL): interim results of a multicenter, open-label, phase 2 study. *Blood; ASH Annual Meeting Abstracts* 2012, 120(686).
192. Wilson WH, Young RM, Schmitz R, Yang Y, Pittaluga S, Wright G, Lih CJ, Williams PM, Shaffer AL, Gerecitano J *et al*: Targeting B cell receptor signaling with ibrutinib in diffuse large B cell lymphoma. *Nat Med* 2015, 21(8):922-926.
193. Wilson WH, O'Connor OA, Czuczman MS, LaCasce AS, Gerecitano JF, Leonard JP, Tulpule A, Dunleavy K, Xiong H, Chiu YL *et al*: Navitoclax, a targeted high-affinity inhibitor of BCL-2, in lymphoid malignancies: a phase 1 dose-escalation study of safety, pharmacokinetics, pharmacodynamics, and antitumour activity. *Lancet Oncol* 2010, 11(12):1149-1159.
194. Davids MS, Seymour JF, Gerecitano JF, Kahl BS, Pagel JM, Wierda WG, Anderson M, Rudersdorf NK, Gressick LA, Montalvo NP *et al*: Updated results of a phase I first-in-human study of the BCL-2 inhibitor ABT-199 (GDC-0199) in patients with relapsed/refractory non-Hodgkin lymphoma (NHL). *J Clin Oncol* 2013, 31:15.
195. Davids MS, Seymour JF, Gerecitano JF, Kahl BS, Pagel JM, Wierda WG, Anderson M, Rudersdorf NK, Gressick LA, Montalvo NP *et al*: Phase I study of ABT-199 (GDC-0199) in patients with relapsed/refractory (R/R) non-Hodgkin lymphoma (NHL): responses observed in diffuse large B-cell (DLBCL) and follicular lymphoma (FL) at higher cohort doses. *J Clin Oncol J Clin Oncol* 2014, 32(55):(suppl; abstr 8522).
196. Robertson MJ, Kahl BS, Vose JM, de Vos S, Laughlin M, Flynn PJ, Rowland K, Cruz JC, Goldberg SL, Musib L *et al*: Phase II study of enzastaurin, a protein kinase C beta inhibitor, in patients with relapsed or refractory diffuse large B-cell lymphoma. *J Clin Oncol* 2007, 25(13):1741-1746.
197. Young RM, Staudt LM: Targeting pathological B cell receptor signalling in lymphoid malignancies. *Nat Rev Drug Discov* 2013, 12(3):229-243.
198. Cerchietti L, Leonard JP: Targeting the epigenome and other new strategies in diffuse large B-cell lymphoma: beyond R-CHOP. *Hematology Am Soc Hematol Educ Program* 2013, 2013:591-595.
199. Morschhauser FA, Cartron G, Thieblemont C, Solal-Celigny P, Haioun C, Bouabdallah R, Feugier P, Bouabdallah K, Asikanius E, Lei G *et al*: Obinutuzumab (GA101) monotherapy in relapsed/refractory diffuse large b-cell lymphoma or mantle-cell lymphoma: results from the phase II GAUGUIN study. *J Clin Oncol* 2013, 31(23):2912-2919.
200. Hainsworth JD, Arrowsmith ER, McCleod M, Hsi ED, Hamid O, Shi P, Lin BK, Fayad LE: An open-label, randomized, phase 2 Study of R-CHOP plus enzastaurin versus R-CHOP in the first-line treatment of patients with intermediate- or high-riskdiffuse large B-cell lymphoma. *Leuk Lymphoma* 2015:1-11.
201. Ivanov V, Coso D, Chetaille B, Esterni B, Olive D, Aurran-Schleinitz T, Schiano JM, Stoppa AM, Broussais-Guillaumot F, Blaise D *et al*: Efficacy and safety of lenalinomide combined with rituximab in patients with relapsed/refractory diffuse large B-cell lymphoma. *Leuk Lymphoma* 2014, 55(11):2508-2513.

202. Wang M, Fowler N, Wagner-Bartak N, Feng L, Romaguera J, Neelapu SS, Hagemeister F, Fanale M, Oki Y, Pro B *et al*: Oral lenalidomide with rituximab in relapsed or refractory diffuse large cell, follicular and transformed lymphoma: a phase II clinical trial. *Leukemia* 2013, 27(9):1902-1909.
203. Feldman T, Mato AR, Chow KF, Protomastro EA, Yannotti KM, Bhattacharyya P, Yang X, Donato ML, Rowley SD, Carini C *et al*: Addition of lenalidomide to rituximab, ifosfamide, carboplatin, etoposide (RICER) in first-relapse/primary refractory diffuse large B-cell lymphoma. *Br J Haematol* 2014, 166(1):77-83.
204. Witzig TE, Tomblyn MB, Misleh JG, Kio EA, Sharkey RM, Wegener WA, Goldenberg DM: Anti-CD22 90Y-epratuzumab tetraxetan combined with anti-CD20 veltuzumab: a phase I study in patients with relapsed/refractory, aggressive non-Hodgkin lymphoma. *Haematologica* 2014, 99(11):1738-1745.
205. Younes A, Thieblemont C, Morschhauser F, Flinn I, Friedberg JW, Amorim S, Hivert B, Westin J, Vermeulen J, Bandyopadhyay N *et al*: Combination of ibrutinib with rituximab, cyclophosphamide, doxorubicin, vincristine, and prednisone (R-CHOP) for treatment-naive patients with CD20-positive B-cell non-Hodgkin lymphoma: a non-randomised, phase 1b study. *The Lancet Oncology* 2014, 15(9):1019-1026.
206. Roschewski M, Staudt LM, Wilson WH: Diffuse large B-cell lymphoma-treatment approaches in the molecular era. *Nat Rev Clin Oncol* 2014, 11(1):12-23.
207. Nowakowski GS, LaPlant B, Macon WR, Reeder CB, Foran JM, Nelson GD, Thompson CA, Rivera CE, Inwards DJ, Micallef IN *et al*: Lenalidomide Combined With R-CHOP Overcomes Negative Prognostic Impact of Non-Germinal Center B-Cell Phenotype in Newly Diagnosed Diffuse Large B-Cell Lymphoma: A Phase II Study. *J Clin Oncol* 2014.
208. Cunningham D, Hawkes EA, Jack A, Qian W, Smith P, Mouncey P, Pocock C, Ardeshna KM, Radford JA, McMillan A *et al*: Rituximab plus cyclophosphamide, doxorubicin, vincristine, and prednisolone in patients with newly diagnosed diffuse large B-cell non-Hodgkin lymphoma: a phase 3 comparison of dose intensification with 14-day versus 21-day cycles. *Lancet* 2013, 381(9880):1817-1826.
209. Delarue R, Tilly H, Mounier N, Petrella T, Salles G, Thieblemont C, Bologna S, Ghesquieres H, Hacini M, Fruchart C *et al*: Dose-dense rituximab-CHOP compared with standard rituximab-CHOP in elderly patients with diffuse large B-cell lymphoma (the LNH03-6B study): a randomised phase 3 trial. *The Lancet Oncology* 2013, 14(6):525-533.
210. Habermann TM, Weller EA, Morrison VA, Gascoyne RD, Cassileth PA, Cohn JB, Dakhil SR, Woda B, Fisher RI, Peterson BA *et al*: Rituximab-CHOP versus CHOP alone or with maintenance rituximab in older patients with diffuse large B-cell lymphoma. *J Clin Oncol* 2006, 24(19):3121-3127.
211. Maddocks K, Christian B, Jaglowski S, Flynn J, Jones JA, Porcu P, Wei L, Jenkins C, Lozanski G, Byrd JC *et al*: A phase 1/1b study of rituximab, bendamustine, and ibrutinib in patients with untreated and relapsed/refractory non-Hodgkin lymphoma. *Blood* 2015, 125(2):242-248.
212. Tilly H, Morschhauser F, Salles G, Casasnovas RO, Feugier P, Molina TJ, Jardin F, Terriou L, Haioun C, Coiffier B: Phase 1b study of lenalidomide in combination with rituximab-CHOP (R2-CHOP) in patients with B-cell lymphoma. *Leukemia* 2013, 27(1):252-255.
213. Vitolo U, Chiappella A, Franceschetti S, Carella AM, Baldi I, Inghirami G, Spina M, Pavone V, Ladetto M, Liberati AM *et al*: Lenalidomide plus R-CHOP21 in elderly patients with untreated diffuse large B-cell lymphoma: results of the REAL07 open-label, multicentre, phase 2 trial. *Lancet Oncol* 2014, 15(7):730-737.
214. Strauss SJ, Morschhauser F, Rech J, Repp R, Solal-Celigny P, Zinzani PL, Engert A, Coiffier B, Hoelzer DF, Wegener WA *et al*: Multicenter phase II trial of immunotherapy with the humanized anti-CD22 antibody, epratuzumab, in combination with rituximab, in refractory or recurrent non-Hodgkin's lymphoma. *J Clin Oncol* 2006, 24(24):3880-3886.
215. Ruan J, Martin P, Furman RR, Lee SM, Cheung K, Vose JM, Lacasce A, Morrison J, Elstrom R, Ely S *et al*: Bortezomib plus CHOP-rituximab for previously untreated diffuse large B-cell lymphoma and mantle cell lymphoma. *J Clin Oncol* 2011, 29(6):690-697.
216. Barnes JA, Jacobsen E, Feng Y, Freedman A, Hochberg EP, LaCasce AS, Armand P, Joyce R, Sohani AR, Rodig SJ *et al*: Everolimus in combination with rituximab induces complete responses in heavily pretreated diffuse large B-cell lymphoma. *Haematologica* 2013, 98(4):615-619.
217. Johnston PB, Inwards DJ, Colgan JP, Laplant BR, Kabat BF, Habermann TM, Micallef IN, Porrata LF, Ansell SM, Reeder CB *et al*: A Phase II trial of the oral mTOR inhibitor everolimus in relapsed Hodgkin lymphoma. *Am J Hematol* 2010, 85(5):320-324.
218. Witzens-Harig M, Memmer ML, Dreyling M, Hess G: A phase I/II trial to evaluate the safety, feasibility and activity of salvage therapy consisting of the mTOR inhibitor Temsirolimus added to standard

therapy of Rituximab and DHAP for the treatment of patients with relapsed or refractory diffuse large cell B-Cell lymphoma - the STORM trial. *BMC Cancer* 2013, 13:308.
219. Nieto Y, Valdez BC, Thall PF, Ahmed S, Jones RB, Hosing C, Popat U, Shpall EJ, Qazilbash M, Gulbis A *et al*: Vorinostat Combined with High-Dose Gemcitabine, Busulfan and Melphalan with Autologous Stem-Cell Transplantation in Patients with Refractory Lymphomas. *Biol Blood Marrow Transplant* 2015, Jun 11. [Epub ahead of print]
220. Pardee TS, Lee K, Luddy J, Maturo C, Rodriguez R, Isom S, Miller LD, Stadelman KM, Levitan D, Hurd D *et al*: A phase I study of the first-in-class antimitochondrial metabolism agent, CPI-613, in patients with advanced hematologic malignancies. *Clin Cancer Res* 2014, 20(20):5255-5264.
221. Younes A, Romaguera J, Fanale M, McLaughlin P, Hagemeister F, Copeland A, Neelapu S, Kwak L, Shah J, de Castro Faria S *et al*: Phase I study of a novel oral Janus kinase 2 inhibitor, SB1518, in patients with relapsed lymphoma: evidence of clinical and biologic activity in multiple lymphoma subtypes. *J Clin Oncol* 2012, 30(33):4161-4167.
222. Rosenthal A, Mesa RA: Janus kinase inhibitors for the treatment of myeloproliferative neoplasms. *Expert Opin Pharmacother* 2014, 15(9):1265-1276.
223. Jacobsen ED, Sharman JP, Oki Y, Advani RH, Winter JN, Bello CM, Spitzer G, Palanca-Wessels MC, Kennedy DA, Levine P *et al*: Brentuximab vedotin demonstrates objective responses in a phase 2 study of relapsed/refractory DLBCL with variable CD30 expression. *Blood* 2015, 125(9):1394-1402.
224. Wagner-Johnston ND, Goy A, Rodriguez MA, Ehmann WC, Hamlin PA, Radford J, Thieblemont C, Suh C, Sweetenham J, Huang Y *et al*: A Phase 2 Study of Inotuzumab Ozogamicin and Rituximab, Followed by Autologous Stem Cell Transplantation in Patients with Relapsed/Refractory Diffuse Large B-Cell Lymphoma. *Leuk Lymphoma* 2015:1-27.
225. Albertson TM, Sandalic L, Zhao B, Kostic A, Law C: SGN-CD19A: A novel Anti-CD19 antibody drug conjugate. *Cancer Res* 2014, 74(19 Suppl).
226. Forero-Torres A, Moskowitz C, Advani RH, Shah BD, Kostic A, Albertson TM, Sandalic L, Zhao B, Law C: Interim analysis of a phase 1, open-label, dose-escalation study of SGN-CD19A in patients with relapsed or refractory B-lineage non-Hodgkin lymphoma (NHL). ASCO Annual Meeting. Oral Abstract Session. *J Clin Oncol* 2014, 32:(suppl; abstr 8505).
227. Preliminary Results of a Phase II Randomized Study (ROMULUS) of Polatuzumab Vedotin or Pinatuzumab Vedotin Plus Rituximab in Patients With Relapsed/Refractory Non-Hodgkin Lymphoma (NHL). *Clin Adv Hematol Oncol* 2014, 12(8 Suppl 16):15-18.
228. Palanca-Wessels MC, Czuczman M, Salles G, Assouline S, Sehn LH, Flinn I, Patel MR, Sangha R, Hagenbeek A, Advani R *et al*: Safety and activity of the anti-CD79B antibody-drug conjugate polatuzumab vedotin in relapsed or refractory B-cell non-Hodgkin lymphoma and chronic lymphocytic leukaemia: a phase 1 study. *The Lancet Oncology* 2015, 16(6):704-715.
229. Schuster SJ, Svoboda J, Nasta S, Porter DL, Mato A, Shah GD, Landsburg DJ, Chong EA, Lacey SF, Melenhorst JJ *et al*: Phase IIa Trial of Chimeric Antigen Receptor Modified T Cells Directed Against CD19 (CTL019) in Patients with Relapsed or Refractory CD19+ Lymphomas, American Society of Clinical Oncology Annual Meeting 1 June 2015: Abstract 8516. *J Clin Oncol* 2015, 33:(suppl; abstr 8516).
230. Wang Y, Zhang WY, Han QW, Liu Y, Dai HR, Guo YL, Bo J, Fan H, Zhang Y, Zhang YJ *et al*: Effective response and delayed toxicities of refractory advanced diffuse large B-cell lymphoma treated by CD20-directed chimeric antigen receptor-modified T cells. *Clin Immunol* 2014, 155(2):160-175.
231. Amengual JE, Johannet PM, Lombardo M, Zullo KM, Hoehn D, Bhagat G, Scotto L, Jirau-Serrano X, Radeski D, Heinen J *et al*: Dual targeting of protein degradation pathways with the selective HDAC6 inhibitor, ACY-1215, and bortezomib is synergistic in lymphoma. *Clin Cancer Res* 2015, Jun 26. [Epub ahead of print]
232. Bodo J, Sedlak J, Maciejewski JP, Almasan A, Hsi ED: HDAC inhibitors potentiate the apoptotic effect of enzastaurin in lymphoma cells. *Apoptosis* 2011, 16(9):914-923.
233. Kalac M, Scotto L, Marchi E, Amengual J, Seshan VE, Bhagat G, Ulahannan N, Leshchenko VV, Temkin AM, Parekh S *et al*: HDAC inhibitors and decitabine are highly synergistic and associated with unique gene-expression and epigenetic profiles in models of DLBCL. *Blood* 2011, 118(20):5506-5516.
234. Bhadury J, Nilsson LM, Veppil Muralidharan S, Green LC, Li Z, Gesner EM, Hansen HC, Keller UB, McLure KG, Nilsson JA: BET and HDAC inhibitors induce similar genes and biological effects and synergize to kill in Myc-induced murine lymphoma. *Proc Natl Acad Sci U S A* 2014, 111(26):E2721-2730.

235. Rahmani M, Aust MM, Benson EC, Wallace L, Friedberg J, Grant S: PI3K/mTOR inhibition markedly potentiates HDAC inhibitor activity in NHL cells through BIM- and MCL-1-dependent mechanisms in vitro and in vivo. *Clin Cancer Res* 2014, 20(18):4849-4860.
236. Ackler S, Xiao Y, Mitten MJ, Foster K, Oleksijew A, Refici M, Schlessinger S, Wang B, Chemburkar SR, Bauch J *et al*: ABT-263 and rapamycin act cooperatively to kill lymphoma cells in vitro and in vivo. *Mol Cancer Ther* 2008, 7(10):3265-3274.
237. Kunkalla K, Liu Y, Qu C, Leventaki V, Agarwal NK, Singh RR, Vega F: Functional inhibition of BCL2 is needed to increase the susceptibility to apoptosis to SMO inhibitors in diffuse large B-cell lymphoma of germinal center subtype. *Ann Hematol* 2013, 92(6):777-787.
238. Rickles RJ, Tam WF, Giordano TP, 3rd, Pierce LT, Farwell M, McMillin DW, Necheva A, Crowe D, Chen M, Avery W *et al*: Adenosine A2A and beta-2 adrenergic receptor agonists: novel selective and synergistic multiple myeloma targets discovered through systematic combination screening. *Mol Cancer Ther* 2012, 11(7):1432-1442.
239. Zang C, Eucker J, Liu H, Muller A, Possinger K, Scholz CW: Concurrent inhibition of PI3-kinase and mTOR induces cell death in diffuse large B cell lymphomas, a mechanism involving down regulation of Mcl-1. *Cancer Lett* 2013, 339(2):288-297.
240. Zoellner AK, Bayerl S, Hutter G, Zimmermann Y, Hiddemann W, Dreyling M: Temsirolimus inhibits cell growth in combination with inhibitors of the B-cell receptor pathway. *Leuk Lymphoma* 2015, Aug 3:1-8. [Epub ahead of print]
241. Jia L, Gopinathan G, Sukumar JT, Gribben JG: Blocking autophagy prevents bortezomib-induced NF-kappaB activation by reducing I-kappaBalpha degradation in lymphoma cells. *PLoS One* 2012, 7(2):e32584.
242. Gupta M, Hendrickson AE, Yun SS, Han JJ, Schneider PA, Koh BD, Stenson MJ, Wellik LE, Shing JC, Peterson KL *et al*: Dual mTORC1/mTORC2 inhibition diminishes Akt activation and induces Puma-dependent apoptosis in lymphoid malignancies. *Blood* 2012, 119(2):476-487.
243. Shortt J, Martin BP, Newbold A, Hannan KM, Devlin JR, Baker AJ, Ralli R, Cullinane C, Schmitt CA, Reimann M *et al*: Combined inhibition of PI3K-related DNA damage response kinases and mTORC1 induces apoptosis in MYC-driven B-cell lymphomas. *Blood* 2013, 121(15):2964-2974.
244. Coffey G, Betz A, DeGuzman F, Pak Y, Inagaki M, Baker DC, Hollenbach SJ, Pandey A, Sinha U: The novel kinase inhibitor PRT062070 (Cerdulatinib) demonstrates efficacy in models of autoimmunity and B-cell cancer. *J Pharmacol Exp Ther* 2014, 351(3):538-548.
245. Wu H, Hu C, Wang A, Weisberg EL, Chen Y, Yun CH, Wang W, Liu Y, Liu X, Tian B *et al*: Discovery of a BTK/MNK dual inhibitor for lymphoma and leukemia. *Leukemia* 2015.
246. Fenske TS, Shah NM, Kim KM, Saha S, Zhang C, Baim AE, Farnen JP, Onitilo AA, Blank JH, Ahuja H *et al*: A phase 2 study of weekly temsirolimus and bortezomib for relapsed or refractory B-cell non-Hodgkin lymphoma: A Wisconsin Oncology Network study. *Cancer* 2015 Oct 1;121(19):3465-71
247. Burke RT, Meadows S, Loriaux MM, Currie KS, Mitchell SA, Maciejewski P, Clarke AS, Dipaolo JA, Druker BJ, Lannutti BJ *et al*: A potential therapeutic strategy for chronic lymphocytic leukemia by combining Idelalisib and GS-9973, a novel spleen tyrosine kinase (Syk) inhibitor. *Oncotarget* 2014, 5(4):908-915.
248. Ivanov V, Tabouret E, Chuto G, Chetaille B, Fezoui H, Coso D, Rey J, Aurran-Schleinitz T, Schiano JM, Stoppa AM *et al*: Rituximab-lenalidomide-dexamethasone induces complete and durable remission in relapsed refractory diffuse large B-cell non-Hodgkin lymphoma. *Leuk Lymphoma* 2010, 51(9):1758-1760.
